# Checklist of British and Irish Hymenoptera - Braconidae

**DOI:** 10.3897/BDJ.4.e8151

**Published:** 2016-04-21

**Authors:** Gavin R. Broad, Mark R. Shaw, H. Charles J. Godfray

**Affiliations:** ‡ The Natural History Museum, London, United Kingdom; § National Museums of Scotland, Edinburgh, United Kingdom; | University of Oxford, Oxford, United Kingdom

**Keywords:** Britain, Ireland, fauna, Ichneumonoidea

## Abstract

**Background:**

The checklist of British and Irish Braconidae is revised, based in large part on the collections of the National Museums of Scotland, Edinburgh, and the Natural History Museum, London. Distribution records are provided at the country level together with extensive synonymy and bibliography.

**New information:**

Of the 1,338 species regarded as valid, presumed native and certainly identified, 83 are here recorded for the first time from the British Isles. One new synonym is established (*Dyscritus suffolciensis* Morley, 1933 = *Syntretus splendidus* (Marshall, 1887) **syn. nov.**)

## Introduction

The Braconidae is one of two families of the superfamily Ichneumonoidea, along with the Ichneumonidae. Given the size of each family in Britain (over 1,300 braconids and 2,500 ichneumonids) we are publishing the two checklists separately. This is one part of a series of papers revising the British and Irish list of Hymenoptera, that started with Broad and Livermore (2014a), Broad and Livermore (2014b) and Liston et al. (2014). For the background and rationale behind these British Hymenoptera checklists see Broad (2014). The bases for the taxonomy are Fauna Europaea and Taxapad (Yu et al. 2012) (braconid data for both compiled by Kees van Achterberg) but we have not relied on these sources for data on species occurrence in Britain. Rather, we have relied mostly on the primary literature and collections, especially NMS and BMNH. Indeed, large parts of the checklist rely on original work, identifying specimens in these collections.

Much of the synonymy adopted here is equivalent to the German list (Belokobylskij et al. 2003) but where there are taxonomic differences of opinion, usually van Achterberg’s interpretation has been followed, largely for consistency with Fauna Europaea, but also because of van Achterberg’s experience with many groups of braconids. The biggest departure from these sources is in the generic classification of Microgastrinae, for which we largely follow Mason (1981).

We reference all additions to and deletions from the British list since Huddleston (1978) and record country-level distribution within the British Isles, but regarding Ireland as one. We made much use of O'Connor et al. (1999) for Irish records. Because the braconid literature is extensive and scattered we also provide many taxonomic references. The braconid section of the 1978 British checklist (Huddleston 1978) repeats many of Shenefelt’s (e.g. Shenefelt 1973, Shenefelt 1974) mistakes and these are rectified here.

Figs 1, 2, 3, 4 illustrate a tiny part of the morphological and biological diversity of Braconidae. Shaw and Huddleston (1991) provide an introduction to the varied biology of this family of parasitoid wasps.

## Materials and methods

For a more detailed description of the background and rationale to the Hymenoptera checklist, see Broad (2014). We have tried to account for every name on the 1978 (Huddleston 1978) checklist and have referenced all additions to and deletions from that list. We provide rather extensive synonymy and citations because if you do not have access to the Taxapad database (Yu et al. 2012) it can be very difficult to trace the fate of names in the voluminous and scattered literature. Conventions and abbreviations are listed below.

[***species***] taxon deleted from the British and Irish list

BMNH Natural History Museum, London

NMS National Museums of Scotland, Edinburgh

# known introductions occurring only under artificial conditions

? status (including uncertain synonymy) or identification in the British Isles uncertain

misident. has been misidentified as this name

nom. dub. *nomen dubium*, a name of doubtful status

nom. ob. *nomen oblitum*, ‘forgotten name’, does not have priority over a younger name

nom. nov. *nomen novum*, a replacement name

nom. nud. *nomen nudum*, an unavailable name, with no type specimen

preocc. name preoccupied (junior homonym)

stat. rev. *status revocatus*, revived status (e.g., raised from synonymy)

unavailable name unavailable under provisions of the ICZN code

var. variety, only available as a valid name under certain provisions of the ICZN code

Word document and spreadsheet versions of the checklist are available in the supplementary materials. Future updates to the British and Irish list will be incorporated in an online version of the checklist at Hymenoptera of the British Isles.

## Checklists

### Agathidinae

#### Agathidinae

Haliday, 1833

##### Notes

Except for *Agathis* and ‘*Bassus*’ species (i.e. including *Lytopylus* and *Therophilus*), distribution and synonymic data from Nixon (1986).

#### Agathidini

Nees, 1814

BASSINI Nees, 1812 invalid
EUMICRODINI Förster, 1863
BASSINI Förster, 1869 preocc.
MICRODINI Ashmead, 1900
MESOCOELINI Viereck, 1918
ANEUROBRACONINI Fahringer, 1936
EARININI Sharkey, 1992

#### Agathis

Latreille, 1804

AENIGMOSTOMUS Ashmead, 1900
METRIOSOMA Szépligeti, 1902
BAEOGNATHA Kokujev, 1903 synonymy by Achterberg (2011)
LISSAGATHIS Cameron, 1911
RHAMPHAGATHIS Tobias, 1962

##### Notes

Distribution and synonymic data from Nixon (1986) and Simbolotti and Achterberg (1999).

Species of *Agathis* excluded from the British and Irish list:

[*malvacearum* Latreille, 1805; syn. *panzeri* (Jurine, 1807, *Ichneumon*); *metzneriae* Muesebeck, 1967] Listed by Huddleston (1978); probably refers to *varipes*.

#### Agathis anglica

Marshall, 1885

longicauda Kokujev, 1895 preocc.
Agathis anglica ?*marshalli* Fahringer, 1937
albanica Fischer, 1957
syriaca Fischer, 1957
caucasica Tobias, 1963
taiwanensis Chou & Sharkey, 1989

##### Distribution

England, Wales

#### Agathis assimilis

Kokujev, 1895

propinqua Kokujev, 1895
jakowlewi Kokujev, 1895
sibirica Telenga, 1933
anchisiades Nixon, 1986

##### Distribution

Scotland

##### Notes

added by Nixon (1986)

#### Agathis breviseta

Nees, 1812

achterbergi Nixon, 1986

##### Distribution

England, Scotland, Ireland

##### Notes

Simbolotti and Achterberg (1999) synonymised *Agathis achterbergi* Nixon, 1986 under *breviseta* but it is listed as a valid species in Taxapad (Yu et al. 2012).

#### Agathis fuscipennis

(Zetterstedt, 1838)

Microgaster fuscipennis Zetterstedt, 1838
breviseta misident.
rostrata misident.
glabricula Thomson, 1895
schmiedeknechti Kokujev, 1895
annulata Fahringer, 1937
meridionellae Fischer, 1957
albicostellae Fischer, 1966
artemesiana Fischer, 1966

##### Distribution

England, Ireland

#### Agathis griseifrons

Thomson, 1895

laticarpa Telenga, 1955

##### Distribution

England, Ireland

#### Agathis lugubris

(Förster, 1863)

Cenostomus lugubris Förster, 1863
minuta Niezabitowski, 1910

##### Distribution

England, Scotland, Wales, Ireland

##### Notes

added by Nixon (1986)

#### Agathis montana

Shestakov, 1932

zaykovi Nixon, 1986

##### Distribution

England

##### Notes

added by Simbolotti and Achterberg (1999)

#### Agathis nigra

Nees, 1812

testaceipes Fischer, 1957
kasachstanica Tobias, 1963
nixoni Belokobylskij & Jervis, 1998

##### Distribution

England

#### Agathis rufipalpis

Nees, 1812

##### Distribution

Ireland

#### Agathis tibialis

Nees, 1814

genualis Marshall, 1898

##### Distribution

England

##### Notes

NMS, det. van Achterberg, added here

#### Agathis varipes

Thomson, 1895

simulatrix Kokujev, 1895
rufipes Ivanov, 1899
dissimilis Shestakov, 1928
rufilabialis Fahringer, 1937
glabricollis Telenga, 1957
serratulae Tobias, 1963
lederi Fischer, 1968
ariadne Nixon, 1986

##### Distribution

England

##### Notes

added by Nixon (1986)

#### Bassus

Fabricius, 1804

MICRODUS Nees, 1814
DIPLOZON Haliday, 1833
EURYZONA Haliday, 1838 nom. nud.
EUMICRODUS Förster, 1863
HEMIOGASTER Enderlein, 1920 synonymy by Achterberg and Long (2010)

##### Notes

Following molecular phylogenetic analysis of Agathidinae (Sharkey et al. 2006), the genus *Bassuss.l.* was recognised as being a polyphyletic assemblage. Species are now being described in or reassigned to *Lytopylus*, *Thermophilus* and the non-British *Camptothlipsis* (Sharkey et al. 2009, Stevens et al. 2010, Stevens et al. 2011, Achterberg and Long 2010, Achterberg 2011) but very few European species have been formally transferred. The species on the British list have therefore been reassigned here on the bases of recent generic keys (e.g. Sharkey et al. 2009) in anticipation of future taxonomy. Whereas *Bassuss. s.* is now restricted to a small group of species (only one in Britain), *Therophilus* remains large and probably para- or polyphyletic. Distribution and synonymic data for *Bassus*, *Lytopylus* and *Therophilus* from Nixon (1986) and Simbolotti and Achterberg (1992).

#### Bassus calculator

(Fabricius, 1798)

Ichneumon calculator Fabricius, 1798
abscissus (Ratzeburg, 1844, *Microdus*)

##### Distribution

England

#### Earinus

Wesmael, 1837

Earinus Förster, 1863

#### Earinus elator

(Fabricius, 1804)

Banchus elator Fabricius, 1804
nitidulus (Nees, 1814, *Microdus*)
thoracicus (Nees, 1834, *Microdus*)
major (Fonscolombe, 1846, *Agathis*)
pilosus Tobias, 1960

##### Distribution

England, Scotland, Ireland

#### Earinus gloriatorius

(Panzer, 1809)

Bassus gloriatorius Panzer, 1809
gloriator (Nees, 1812, *Microdus*)
ochropes (Curtis, 1829, *Microdus*) nom. nud.
affinis (Wesmael, 1837, *Microdus*)
delusor (Wesmael, 1837, *Microdus*)
tuberculatus (Wesmael, 1837, *Microdus*)
varicoxis (Wesmael, 1837, *Microdus*)
niger (Zetterstedt, 1838, *Microgaster*)
bicingulatus (Thomson, 1895, *Agathis*)
ochropes Lyle, 1920
ruficoxis Fahringer, 1937

##### Distribution

England, Scotland, Ireland, Isle of Man

#### Earinus transversus

Lyle, 1920

##### Distribution

England

##### Notes

distribution data from Shaw (2005)

#### Lytopylus

Förster, 1863

AEROPHILUS Szépligeti, 1902 synonymy by Sharkey et al. (2006)
NEOMICRODUS Szépligeti, 1908
AEROPHILOPSIS Viereck, 1913
AEROPHILINA Enderlein, 1920
IOXIA Enderlein, 1920
HORMAGATHIS Brues, 1926
OBESOMICRODUS Papp, 1971
FACILAGATHIS van Achterberg & Chen, 2004

##### Notes

See comments under *Bassus*; generic synonymy from Sharkey et al. (2009).

#### Lytopylus rufipes

(Nees, 1814)

Microdus rufipes Nees, 1814
germanicus (Enderlein, 1904, *Braunsia*)
diversus Muesebeck, 1933
amurensis (Shestakov, 1940, *Microdus*)

##### Distribution

England

#### Therophilus

Wesmael, 1837

ORGILONEURA Ashmead, 1900
AGATHIELLA Szépligeti, 1902
AEROPHILIODES Strand, 1911

##### Notes

See comments under *Bassus*; generic synonymy from Sharkey et al. (2009).

Species excluded from the British and Irish list:

[*brevicaudis* (Reinhard, 1867, *Microdus*)] Listed as a ‘species inquirendae’ by Nixon (1986) but included as a German species by Simbolotti and Achterberg (1992). Transferred to *Bassus* by Simbolotti and Achterberg (1999) but not listed as a species of *Therophilus* by Sharkey and Stoelb (2012).

[*nugax* (Reinhard, 1867, *Microdus*); syn. *rufiventris* (Abdinbekova, 1975, *Microdus*)] Listed by Huddleston (1978) but no mention of British specimens by Nixon (1986) or Simbolotti and Achterberg (1992).

#### Therophilus arcuatus

(Reinhard, 1867)

Microdus arcuatus Reinhard, 1867

##### Notes

Taken out of synonymy with conspicuus by Simbolotti and Achterberg (1992).

#### Therophilus cingulipes

(Nees, 1812)

Microdus cingulipes Nees, 1812
nantouensis Chou & Sharkey, 1989

##### Distribution

England, Ireland

#### Therophilus clausthalianus

(Ratzeburg, 1844)

Ichneumon clausthalianus Ratzeburg, 1844

##### Distribution

England, Ireland

#### Therophilus conspicuus

(Wesmael, 1837)

Microdus conspicuus Wesmael, 1837
zonatus (Marshall, 1885, *Earinus*)
carpocapsae Cushman, 1915
angustatus (Telenga, 1955, *Microdus*)
variabilis Chou & Sharkey, 1989

##### Distribution

England, Ireland

#### Therophilus tegularis

(Thomson, 1895)

Agathis tegularis Thomson, 1895

##### Distribution

England

##### Notes

added by Simbolotti and Achterberg (1992)

#### Therophilus tumidulus

(Nees, 1812)

Microdus tumidulus Nees, 1812
intermedius (Ivanov, 1899, *Eumicrodus*)
annae (Enderlein, 1908, *Microdus*)
aino (Watanabe, 1937, *Microdus*)
ruficoxis (Fahringer, 1937, *Microdus*)
rufus (Fahringer, 1938, *Microdus*)
bicolor (Shestakov, 1940, *Microdus*) preocc.
victoris (Telenga, 1955, *Microdus*)
shestakovi (Shenefelt, 1970, *Agathis*)
anuphrievi (Tobias, 1986, *Microdus*)

##### Distribution

England, Scotland, Ireland

#### Therophilus

Wesmael, 1837

##### Notes

Species *incertae sedis* within Agathidinae; Sharkey and Stoelb (2012) defined *Therophilus* as a monophyletic genus and listed the included species, but several species currently classified in *Therophilus* are of uncertain generic placement.

#### Therophilus dimidiator

(Nees, 1834)

Microdus dimidiator Nees, 1834
cingulator (Ratzeburg, 1852, *Microdus*)
laticinctus (Cresson, 1873, *Microdus*)
ocellanae (Richardson, 1913, *Microdus*)

##### Distribution

England

##### Notes

Listed by Huddleston (1978) but no mention of British specimens by Nixon (1986), who states, however, that the traditional interpretation of the species is probably correct. No British or Irish specimens were seen by Simbolotti and Achterberg (1992) but van Achterberg has identified an English specimen in BMNH as *Therophilus dimidiator*.

#### Therophilus epinotiae

(van Achterberg, 1992)

Bassus epinotiae van Achterberg, 1992

##### Distribution

England

##### Notes

added by Simbolotti and Achterberg (1992)

#### Therophilus linguarius

(Nees, 1812)

Microdus linguarius Nees, 1812
minor (Enderlein, 1908, *Microdus*)
kaszabi (Papp, 1967, *Vipio*)

##### Distribution

England

#### Therophilus mediator

(Nees, 1814)

Microdus mediator Nees, 1814
lugubrator (Ratzeburg, 1852, *Microdus*)

##### Distribution

England

##### Notes

Transferred from *Agathis* by Simbolotti and Achterberg (1999).

#### Therophilus pumilus

(Ratzeburg, 1844)

Microdus pumilus Ratzeburg, 1844

##### Distribution

England

##### Notes

NMS, det. Shaw, added here; listed by Huddleston (1978) but no mention of British specimens by Nixon (1986) or Simbolotti and Achterberg (1992). Transferred from *Agathis* by Simbolotti and Achterberg (1999) but listed as a species of *Agathis* in Taxapad (Yu et al. 2012).

#### Therophilus rugulosus

(Nees, 1834)

Microdus rugulosus Nees, 1834
compeditus (Vollenhoven, 1878, *Microdus*)
punctatus (Abdinbekova, 1975, *Microdus*)

##### Distribution

England, Ireland

### Alysiinae

#### Alysiinae

Leach, 1815

#### Alysiini

Leach, 1815

ALLOEINI Ashmead, 1900

#### Adelurola

Strand, 1928

ADELURA Förster, 1863 preocc.
NEOCARPA Fischer, 1966

#### Adelurola florimela

(Haliday, 1838)

Alysia florimela Haliday, 1838
multiarticulata (Marshall, 1898, *Phaenocarpa*)
pentapleuroides (Fischer, 1971, *Dapsilarthra*) unavailable

##### Distribution

England, Scotland, Ireland, Isle of Man

#### Alloea

Haliday, 1833

DIASPASTA Förster, 1863
LAMADATHA Cameron, 1900

#### Alloea contracta

Haliday, 1833

testaceipes (Cameron, 1900, *Lamadatha*)

##### Distribution

England, Scotland, Wales, Ireland

#### Alloea lonchopterae

Fischer, 1966

##### Distribution

England, Isle of Man

##### Notes

Added by Godfray and Bland (2011) English record from specimen in BMNH.

#### Alysia

Latreille, 1804

#### Alysia

Latreille, 1804

CECHENUS Illiger, 1807
BASSUS Nees, 1812
GONIARCHA Förster, 1863
STROPHAEA Förster, 1863

##### Notes

Much of the taxonomy and distribution from Wharton (1986).

Species of Alysia (Alysia) excluded from the British and Irish list:

[*cingulata* Nees, 1834 nom. dub.]

#### Alysia (Alysia) alticola

(Ashmead, 1890)

Pentapleura alticola Ashmead, 1890
soror Marshall, 1894

##### Distribution

England, Scotland, Wales, Isle of Man

#### Alysia (Alysia) frigida

Haliday, 1838

##### Distribution

England

##### Notes

added by Godfray and Achterberg (2015)

#### Alysia (Alysia) incongrua

Nees, 1834

##### Distribution

England, Scotland, Ireland

#### Alysia (Alysia) lucia

Haliday, 1838

diversiceps Fischer, 1967

##### Distribution

Scotland

#### Alysia (Alysia) lucicola

Haliday, 1838

##### Distribution

England, Scotland, Ireland

#### Alysia (Alysia) luciella

Stelfox, 1941

##### Distribution

Ireland

#### Alysia (Alysia) manducator

(Panzer, 1799)

Ichneumon manducator Panzer, 1799
haematopa (Gmelin, 1790, *Ichneumon*) preocc.
stercoraria Latreille, 1805
apicalis Curtis, 1826
similis Curtis, 1826
stercorator Lamarck, 1835
curtungula Thomson, 1895
bucephala Marshall, 1898
manducatrix Schulz, 1906

##### Distribution

England, Scotland, Wales, Ireland, Isle of Man

#### Alysia (Alysia) truncator

(Nees, 1812)

Bassus truncator Nees, 1812

##### Distribution

England, Scotland, Ireland

#### Anarcha

Förster, 1863

##### Notes

Much of the taxonomy and distribution from Wharton (1988b).

Species of Alysia (Anarcha) excluded from the British and Irish list:

[*similis* (Nees, 1812, *Bassus*) nom. dub.]

#### Alysia (Anarcha) atra

Haliday, 1838

##### Distribution

Ireland

#### Alysia (Anarcha) fuscipennis

Haliday, 1838

obscuripes Thomson, 1895

##### Distribution

England, Scotland, Ireland

#### Alysia (Anarcha) mandibulator

(Nees, 1812)

Bassus mandibulator Nees, 1812
loripes Haliday, 1838
mandibulatrix Schulz, 1906

##### Distribution

England, Ireland

#### Alysia (Anarcha) rufidens

Nees, 1834

puncticollis Thomson, 1895

##### Distribution

England, Ireland

#### Alysia (Anarcha) sophia

Haliday, 1835

##### Distribution

Ireland

#### Alysia (Anarcha) subaperta

Thomson, 1895

similis misident.

##### Distribution

England

##### Notes

added by Wharton (1988b)

#### Alysia (Anarcha) thapsina

Wharton, 1988

##### Distribution

Ireland

##### Notes

added by Wharton (1988b)

#### Alysia (Anarcha) tipulae

(Scopoli, 1763)

Ichneumon tipulae Scopoli, 1763
abdominator (Nees, 1814, *Bassus*)
notabilis (Förster, 1863, *Anarcha*)

##### Distribution

England, Scotland, Ireland

#### Alysia (Anarcha) umbrata

Stelfox, 1941

##### Distribution

Ireland

#### Anisocyrta

Förster, 1863

#### Anisocyrta perdita

(Haliday, 1838)

Alysia perdita Haliday, 1838

##### Distribution

Scotland

#### Aphaereta

Förster, 1863

TRICHESIA Provancher, 1880
TRINARIA Provancher, 1886
APHAERETE Dalla Torre, 1898
ATOPANDRIUM Graham, 1952
TRISYNALDIS Fischer, 1958

##### Notes

*Atopandrium* was synonymised under *Aphaereta* by Achterberg (1995) but treated as a valid genus again in Taxapad (Yu et al. 2012).

#### Aphaereta debilitata

Morley, 1933

loripenne (Graham, 1952, *Atopandrium*)
confluctum (Fischer, 1958, *Trisynaldis*)

##### Distribution

England, Wales

#### Aphaereta falcigera

Graham, 1960

##### Distribution

England, Ireland

#### Aphaereta major

(Thomson, 1895)

Alysia major Thomson, 1895
major Marshall, 1898 preocc.

##### Distribution

England, Scotland, Ireland

#### Aphaereta minuta

(Nees, 1811)

Stephanus minutus Nees, 1811
cephalotes (Haliday, 1833, *Alysia*)
fuscipes (Nees, 1834, *Alysia*)
confluens (Ratzeburg, 1844, *Alysia*)
stigmaticalis (Thomson, 1895, *Alysia*)
inepta Morley, 1933

##### Distribution

England, Scotland, Ireland

#### Aphaereta pallipes

(Say, 1829)

Alysia pallipes Say, 1829
auripes (Provancher, 1881, *Trichesia*)
pilicornis (Provancher, 1886, *Trinaria*)
californica Ashmead, 1889
muscae Ashmead, 1889
oscinidis Ashmead, 1889
pallidipes (Dalla Torre, 1898, *Aphaerete*)
delosa Viereck, 1905
subtricarinata Viereck, 1905
pegomyiae Brues, 1907
sarcophagae Gahan, 1914

##### Distribution

England

##### Notes

added by Shaw (1983)

#### Aphaereta tenuicornis

Nixon, 1939

##### Distribution

England, Wales, Ireland

#### Asobara

Förster, 1863

SPANISTA Förster, 1863

#### Asobara tabida

(Nees, 1834)

Alysia tabida Nees, 1834
anomala (Thomson, 1895, *Alysia*)
crenulata (Fahringer, 1935, *Phaenocarpa*)

##### Distribution

England, Ireland

#### Aspilota

Förster, 1863

DIPIESTA Förster, 1863
EUSYNALDIS Zaykov & Fischer, 1982
SYNALDIS misident.

##### Notes

Some distribution data from Stelfox and Graham (1951a). Some species treated as belonging to *Dinotrema* by other authors have been included in *Aspilota* in Taxapad (Yu et al. 2012) and there is clearly much work to be done in allocating species to the current generic concepts (see Wharton 1985). The late T. Munk was preparing a revision of the European species of *Aspilotas. l.* (see note under *Dinotrema*).

#### Aspilota acutidentata

(Fischer, 1970)

Synaldis acutidentata Fischer, 1970

##### Distribution

England, Scotland

##### Notes

NMS, BMNH, det. Munk, added here; identified by T. Munk as a species of *Dinotrema*.

#### Aspilota anaphoretica

Fischer, 1973

##### Distribution

England, Scotland, Ireland

##### Notes

NMS, BMNH, det. Munk, added here

#### Aspilota blasii

Fischer, 1973

##### Distribution

England

##### Notes

BMNH, det. Munk, added here

#### Aspilota compressiventris

Stelfox & Graham, 1951

##### Distribution

England

#### Aspilota curta

Marshall, 1895

##### Distribution

England

#### Aspilota daemon

Stelfox and Graham, 1948

##### Distribution

England, Scotland, Ireland

#### Aspilota delicata

Fischer, 1973

##### Distribution

England, Scotland, Ireland

##### Notes

BMNH, det. Munk, added here

#### Aspilota efoveolata

Thomson, 1895

pneumatica Fischer, 1973

##### Distribution

England, Scotland, Wales

##### Notes

NMS, BMNH, det. Munk, added here

#### Aspilota flagellaris

Fischer, 1973

##### Distribution

England, Scotland, Ireland

##### Notes

BMNH, det. Munk, added here

#### Aspilota fuscicornis

(Haliday, 1838)

Alysia fuscicornis Haliday, 1838
minuta (Nees, 1812, *Bassus*)
exile (Ruthe, 1859, *Orthostigma*)
dilatata (Thomson, 1895, *Alysia*)

##### Distribution

England, Scotland, Ireland

#### Aspilota globipes

(Fischer, 1962)

Synaldis globipes Fischer, 1962

##### Distribution

England, Scotland

##### Notes

NMS, BMNH, det. Munk, added here

#### Aspilota imparidens

Fischer, 1974

##### Distribution

England, Scotland, Wales

##### Notes

NMS, BMNH, det. Munk, added here

#### Aspilota insolita

(Tobias, 1962)

Orthostigma insolita Tobias, 1962

##### Distribution

England, Ireland

##### Notes

BMNH, det. Munk, added here

#### Aspilota intermediana

Fischer, 1975

##### Distribution

England

##### Notes

added by Notton (1991)

#### Aspilota iocosipecta

Fischer, 1974

##### Distribution

England, Scotland, Ireland

##### Notes

NMS, BMNH, det. Munk, added here

#### Aspilota macrops

Stelfox & Graham, 1951

##### Distribution

England, Ireland

#### Aspilota nidicola

Hedqvist, 1972

##### Distribution

England

##### Notes

BMNH, det. Munk, added here

#### Aspilota pillerensis

Fischer, 1973

##### Distribution

England

##### Notes

BMNH, det. Munk, added here

#### Aspilota ruficornis

(Nees, 1834)

Alysia ruficornis Nees, 1834

##### Distribution

England, Scotland, Wales, Ireland

#### Aspilota stenogaster

Stelfox & Graham, 1951

##### Distribution

England

#### Aspilota tetragona

Fischer, 1976

##### Distribution

England, Scotland, Ireland

##### Notes

NMS, BMNH, det. Munk, added here

#### Aspilota vernalis

Stelfox & Graham, 1951

##### Distribution

England, Scotland, Ireland

#### Chasmodon

Haliday, 1838

#### Chasmodon apterus

(Nees, 1812)

Bassus apterus Nees, 1812

##### Distribution

England, Scotland, Wales, Ireland, Isle of Man

#### Cratospila

Förster, 1863

HEDYLUS Marshall, 1891; synonymy by Papp (2009b)

#### Cratospila circe

(Haliday, 1838)

Alysia circe Haliday, 1838
habilis (Marshall, 1891, *Hedylus*); synonymy by Papp (2009b)
annellata (Thomson, 1895, *Alysia*)

##### Distribution

England, Ireland

#### Dapsilarthra

Förster, 1863

##### Notes

*Dapsilarthra* has been used in a very broad sense to include *Adelurola*, *Mesocrina*, *Heterolexis* and *Grammospila*, and in a more narrow sense to include the last two genera. Achterberg (2014) is followed here in according each generic rank.

#### Dapsilarthra apii

(Curtis, 1826)

Alysia apii Curtis, 1826
laevipectus (Thomson, 1895, *Alysia*)
americana (Brues, 1907, *Orthostigma*)

##### Distribution

England, Scotland, Ireland

#### Dapsilarthra sylvia

(Haliday, 1839)

Alysia sylvia Haliday, 1839
carpathica van Achterberg, 1983; synonymy by Achterberg (1997)

##### Distribution

England, Scotland, Ireland

#### Dinotrema

Förster, 1863

##### Notes

Wharton (1980) summarises the arguments against recognition of the genus *Synaldis*; Achterberg (1988b) likewise notes that the genus is defined only by the absence of fore wing vein 2-SR, which is known to be intraspecifically variable (Wharton 1980). Despite this, various authors attach great taxonomic weight to this venational character and maintain *Synaldis* as a valid genus (as reflected by the classification in Yu et al. 2012). Species that would be classified in *Synaldis* (*acutidentata*, *concolor*, *distracta* and *globipes*) are listed here in *Aspilota* and *Dinotrema*, according to Fauna Europaea. Some distribution data from Stelfox and Graham (1951b), Stelfox and Graham (1951a) and Achterberg (1988a). The late T. Munk was revising the European species of *Aspilotas.l.* and gave much helpful advice on the generic placements of the species occurring in Britain and Ireland, some of which is unpublished and will therefore differ from the generic combinations found in, e.g. Belokobylskij et al. (2003) and Taxapad (Yu et al. 2012). Munk also advised that although *Aspilota* is a well-defined genus, *Dinotrema* is not defined by any apomorphies and will be split up.

Species excluded from the British and Irish list:

[*pusillum* (Nees, 1812, *Bassus*)] T. Munk (pers. comm.) regarded this as an unidentified species; the type has been destroyed.

#### Dinotrema

Förster, 1863

ASPILOTA misident.
SYNALDIS Förster, 1863
COLOBOMA Förster, 1863
SPANOMERIS Förster, 1863
SCOTIONEURUS Provancher, 1886
PTERUSA Fischer, 1958; synonymy by Achterberg and Vikberg (2014)
EUDINOSTIGMA Tobias, 1986; synonymy by Wharton (2002)

#### Dinotrema (Dinotrema) aluum

(Stelfox & Graham, 1950)

Aspilota alua Stelfox & Graham, 1950
alva misspelling

##### Distribution

Ireland

#### Dinotrema (Dinotrema) alysiae

Munk & Peris-Felipo, 2013

##### Distribution

England

##### Notes

added by Munk et al. (2013)

#### Dinotrema (Dinotrema) areolatum

(Stelfox & Graham, 1950)

Aspilota areolata Stelfox & Graham, 1950

##### Distribution

England, Scotland

#### Dinotrema (Dinotrema) brevicorne

(Nees, 1814)

Bassus brevicornis Nees, 1814

##### Distribution

England

#### Dinotrema (Dinotrema) brevissimicorne

(Stelfox & Graham, 1948)

Aspilota brevissimicornis Stelfox & Graham, 1948

##### Distribution

Scotland, Ireland

#### Dinotrema (Dinotrema) compressum

(Haliday, 1838)

Alysia compressa Haliday, 1838

##### Distribution

England

#### Dinotrema (Dinotrema) concinnum

(Haliday, 1838)

Alysia concinna Haliday, 1838
maximum (Fischer, 1962, *Synaldis*)
tyrrhena (Masi, 1933, *Aspilota*)

##### Distribution

England, Ireland

##### Notes

*Synaldis maximum* Fischer, 1962 has been treated as a junior synonym by König (1972) and subsequent authors but T. Munk (pers. comm.) regarded this as a valid species of *Dinotrema* that may be found to occur in Britain.

#### Dinotrema (Dinotrema) concolor

(Nees, 1812)

Bassus concolor Nees, 1812
distractum (Ruthe, 1859, *Orthostigma*) unavailable

##### Distribution

England, Ireland

#### Dinotrema (Dinotrema) crassicosta

(Thomson, 1895)

Alysia crassicosta Thomson, 1895

##### Distribution

Scotland

##### Notes

NMS, det. Munk, added here

#### Dinotrema (Dinotrema) cratocera

(Thomson, 1895)

Alysia cratocera Thomson, 1895

##### Distribution

Scotland

##### Notes

BMNH, det. Munk, added here

#### Dinotrema (Dinotrema) denticulatum

(Stelfox & Graham, 1951)

Aspilota denticulata Stelfox & Graham, 1951

##### Distribution

Ireland

#### Dinotrema (Dinotrema) dimidiatum

(Thomson, 1895)

Alysia dimidiata Thomson, 1895

##### Distribution

England

##### Notes

BMNH, det. Munk, added here

#### Dinotrema (Dinotrema) distractum

(Nees, 1834)

Alysia distracta Nees, 1834

#### Dinotrema (Dinotrema) divisum

(Stelfox & Graham, 1950)

Aspilota divisa Stelfox & Graham, 1950
aureliae (Fischer, 1973, *Aspilota*); tentative synonymy by Papp (2004b)

##### Distribution

England, Scotland, Ireland

#### Dinotrema (Dinotrema) erythropum

Förster, 1863

praecipuum (Marshall, 1895, *Aspilota*)

##### Distribution

England, Wales, Ireland

#### Dinotrema (Dinotrema) falsificum

(Stelfox & Graham, 1950)

Aspilota falsifica Stelfox & Graham, 1950

##### Distribution

Scotland, Ireland

#### Dinotrema (Dinotrema) glabrum

(Stelfox & Graham, 1951)

Aspilota glabra Stelfox & Graham, 1951
venustum (Tobias, 1962, *Aspilota*)

##### Distribution

England, Scotland, Ireland

#### Dinotrema (Dinotrema) insidiatrix

(Marshall, 1895)

Aspilota insidiatrix Marshall, 1895

##### Distribution

England

#### Dinotrema (Dinotrema) insignis

(Stelfox & Graham, 1950)

Aspilota insignis Stelfox & Graham, 1950

##### Distribution

England

#### Dinotrema (Dinotrema) iuxtanaeviam

(Fischer, 1980)

Aspilota iuxtanaeviam Fischer, 1978

##### Distribution

England

##### Notes

added by Fischer (1980)

#### Dinotrema (Dinotrema) jaculans

(Haliday, 1838)

Alysia jaculans Haliday, 1838

##### Distribution

England, Scotland, Ireland

#### Dinotrema (Dinotrema) latistigma

(Fischer, 1962)

Synaldis latistigma Fischer, 1962

##### Distribution

England

##### Notes

NMS, det. Munk, added here; treated as a species of *Eudinostigma* in Fauna Europaea and Taxapad (Yu et al. 2012).

#### Dinotrema (Dinotrema) lineola

(Thomson, 1895)

Alysia lineola Thomson, 1895

##### Distribution

England

#### Dinotrema (Dinotrema) liosoma

(Stelfox & Graham,1951)

Aspilota liosoma Stelfox & Graham,1951
caudatum (Thomson, 1895, *Aspilota*) preocc.

#### Dinotrema (Dinotrema) mesocaudatum

van Achterberg, 1988

##### Distribution

England

##### Notes

added by Achterberg (1988b)

#### Dinotrema (Dinotrema) microcera

(Thomson, 1895)

Alysia microcera Thomson, 1895

##### Distribution

England, Scotland, Wales, Ireland

##### Notes

BMNH, det. Munk, added here

#### Dinotrema (Dinotrema) necrophilum

(Hedqvist, 1972)

Aspilota necrophila Hedqvist, 1972

##### Distribution

England

##### Notes

Added by Disney and Munk (2005) and transferred from *Aspilota*.

#### Dinotrema (Dinotrema) nervosum

(Haliday, 1833)

Alysia nervosa Haliday, 1833

##### Distribution

England, Scotland, Ireland

#### Dinotrema (Dinotrema) pulvinatum

(Stelfox & Graham, 1949)

Aspilota pulvinata Stelfox & Graham, 1949

##### Distribution

England, Scotland, Wales, Ireland

##### Notes

Treated as a species of *Eudinostigma* in Fauna Europaea and Taxapad (Yu et al. 2012).

#### Dinotrema (Dinotrema) ruficollis

(Stelfox & Graham, 1950)

Aspilota ruficollis Stelfox & Graham, 1950

##### Distribution

England, Ireland

##### Notes

Distribution data from Achterberg (1988b).

#### Dinotrema (Dinotrema) semicompressum

(Stelfox & Graham, 1949)

Aspilota semicompressa Stelfox & Graham, 1949
parapunctatum (Fischer, 1976, *Aspilota*); synonymy by Papp (2004b)

##### Distribution

England, Scotland, Ireland

#### Dinotrema (Dinotrema) sphaerimembre

(Fischer, 1973)

Aspilota sphaerimembris Fischer, 1973

##### Distribution

Scotland

##### Notes

NMS, det. Munk, added here

#### Dinotrema (Dinotrema) sternaulicum

(Fischer, 1973)

Aspilota sternaulica Fischer, 1973

##### Distribution

England, Scotland

##### Notes

NMS, det. Munk, added here

#### Dinotrema (Dinotrema) tauricum

(Telenga, 1935)

Aspilota taurica Telenga, 1935

##### Distribution

England

##### Notes

added by Achterberg (1988b)

#### Dinotrema (Dinotrema) vesparum

(Stelfox, 1943)

Aspilota vesparum Stelfox, 1943

##### Distribution

England, Scotland

#### Dinotrema (Dinotrema) vituperatum

(Fischer, 1974)

Aspilota vituperata Fischer, 1974

##### Distribution

England, Scotland

##### Notes

NMS, det. Munk, added here

#### Leptotrema

van Achterberg, 1988

##### Notes

Described as a separate genus by Achterberg (1988b), who gives distribution data for the one included species; treated as a subgenus of *Dinotrema* by Wharton (2002).

#### Dinotrema (Leptotrema) dentifemur

(Stelfox, 1943)

Aspilota dentifemur Stelfox, 1943

##### Distribution

England, Scotland, Ireland

#### Prosapha

Förster, 1863

#### Dinotrema (Prosapha) speculum

(Haliday, 1838)

Alysia speculum Haliday, 1838
venustum (Haliday, 1838, *Alysia*)

##### Distribution

England, Ireland

#### Grammospila

Förster, 1863

PARAORTHOSTIGMA Königsmann, 1972

#### Grammospila isabella

(Haliday, 1838)

Alysia isabella Haliday, 1838

##### Distribution

England, Scotland

#### Grammospila rufiventris

(Nees, 1812)

Bassus rufiventris Nees, 1812
flaviventris (Haliday, 1838, *Alysia*)
ochrogaster (Szépligeti, 1898, *Phaenocarpa*)
fuscula Griffiths, 1968

##### Distribution

England, Scotland, Ireland, Isle of Man

##### Notes

*Grammospila fuscula* (Griffiths, 1968, *Dapsilarthra*) removed from synonymy by Achterberg (2014)​.

#### Heterolexis

Förster, 1863

#### Heterolexis balteata

(Thomson, 1895)

Alysia balteata Thomson, 1895

##### Distribution

England, Scotland, Wales, Ireland

#### Heterolexis dictynna

(Marshall, 1895)

Adelura dictynna Marshall, 1895

##### Distribution

England, Scotland, Ireland

#### Idiasta

Förster, 1863

EUPHAENOCARPA Tobias, 1975

#### Idiasta dichrocera

Königsmann, 1960

##### Distribution

England

##### Notes

NMS, det. Shaw, added here

#### Idiasta maritima

(Haliday, 1838)

Alysia maritima Haliday, 1838

##### Distribution

England, Scotland, Ireland

#### Idiasta nephele

(Haliday, 1838)

Alysia nephele Haliday, 1838

##### Distribution

England, Scotland

#### Mesocrina

Förster, 1863

PSEUDOMESOCRINA Königsmann, 1959

#### Mesocrina indagatrix

Förster, 1863

venatrix Marshall, 1895

##### Distribution

England

#### Orthostigma

Ratzeburg, 1844

DELOCARPA Förster, 1863
ISCHNOCARPA Förster, 1863

#### Orthostigma cratospilum

(Thompson, 1895)

Alysia cratospila Thompson, 1895

##### Distribution

England

##### Notes

added by Godfray and Achterberg (2015)

#### Orthostigma longicorne

Königsmann, 1969

##### Distribution

Ireland

#### Orthostigma maculipes

(Haliday, 1838)

Alysia maculipes Haliday, 1838

##### Distribution

England, Ireland

#### Orthostigma pumilum

(Nees, 1834)

Alysia pumila Nees, 1834
flavipes (Ratzeburg, 1844, *Aphidius*)
fulvipes Rondani, 1876 nom. nud.
brunnipes Ratzeburg, 1852
bruneipes Dalla Torre, 1898

##### Distribution

England, Ireland

#### Panerema

Förster, 1863

##### Notes

Distribution data from Achterberg (1988b).

#### Panerema fulvicornis

(Haliday, 1838)

Alysia fulvicornis Haliday, 1838

##### Distribution

Wales, Ireland

#### Panerema inops

Förster, 1863

##### Distribution

England, Wales, Ireland

#### Pentapleura

Förster, 1863

OPISENDEA Förster, 1863
GNATHOSPILA Fischer, 1966

#### Pentapleura angustula

(Haliday, 1838)

Alysia angustula Haliday, 1838
laevipleuris (Tobias, 1962, *Aspilota*)

##### Distribution

England, Scotland, Ireland

#### Pentapleura fuliginosa

(Haliday, 1838)

Alysia fuliginosa Haliday, 1838
carinata (Thomson, 1895, *Alysia*)

##### Distribution

England, Wales, Ireland

#### Pentapleura pumilio

(Nees, 1812)

Bassus pumilio Nees, 1812
triticaphis (Fitch, 1861, *Toxares*)
mesocrinoides Goidanich, 1936

##### Distribution

England, Scotland, Wales, Ireland

#### Phaenocarpa

Förster, 1863

##### Notes

Some taxonomic and distribution data from Achterberg (1997).

#### Homophyla

Förster, 1863

##### Notes

species of Phaenocarpa (Homophyla) excluded from the British and Irish list:

[*pegomyiae* Marshall, 1898] Listed as British by Lyle (1933) but we have not seen any British or Irish specimens.

#### Phaenocarpa (Homophyla) pullata

(Haliday, 1838)

Alysia pullata Haliday, 1838

##### Distribution

England, Ireland

#### Phaenocarpa

Förster, 1863

IDIOLEXIS Förster, 1863; synonymy by Wharton (2002)
MESOTHESIS Förster, 1863
SATHRA Förster, 1863
ASYNAPHES Provancher, 1886
KAHLIA Ashmead, 1900
HOLCALYSIA Cameron, 1905
STIRALYSIA Cameron, 1910
RHOPALONEURA Stelfox, 1941

#### Phaenocarpa (Phaenocarpa) canaliculata

Stelfox, 1941

##### Distribution

England, Ireland

#### Phaenocarpa (Phaenocarpa) conspurcator

(Haliday, 1838)

Alysia conspurcator Haliday, 1838
arctica (Thomson, 1895, *Alysia*)
tatrica Niezabitowski, 1910
remota Papp, 1981

##### Distribution

England, Scotland, Wales, Ireland

#### Phaenocarpa (Phaenocarpa) eugenia

(Haliday, 1838)

Alysia eugenia Haliday, 1838
pectoralis (Zetterstedt, 1838, *Alysia*)
orbicularis Gurasashvilli, 1983

##### Distribution

England, Scotland, Ireland

#### Phaenocarpa (Phaenocarpa) eunice

(Haliday, 1838)

Alysia eunice Haliday, 1838
nimia Stelfox, 1941

##### Distribution

England, Wales, Ireland

#### Phaenocarpa (Phaenocarpa) flavipes

(Haliday, 1838)

Alysia flavipes Haliday, 1838

##### Distribution

England, Ireland

#### Phaenocarpa (Phaenocarpa) frequentator

(Zetterstedt, 1838)

Alysia frequentator Zetterstedt, 1838
frequentatrix (Schulz, 1906, *Alysia*)

##### Distribution

Wales

##### Notes

added by Godfray and Achterberg (2015)

#### Phaenocarpa (Phaenocarpa) galatea

(Haliday, 1838)

Alysia galatea Haliday, 1838

##### Distribution

Scotland, Ireland

#### Phaenocarpa (Phaenocarpa) helophilae

van Achterberg, 1998

##### Distribution

England

##### Notes

added by Achterberg (1998)

#### Phaenocarpa (Phaenocarpa) livida

(Haliday, 1838)

Alysia livida Haliday, 1838
debilis (Förster, 1863, *Sathra*)

##### Distribution

England, Ireland

#### Phaenocarpa (Phaenocarpa) luteipes

Stelfox, 1950

##### Distribution

Ireland

#### Phaenocarpa (Phaenocarpa) maria

(Haliday, 1838)

Alysia maria Haliday, 1838

##### Distribution

England, Ireland

#### Phaenocarpa (Phaenocarpa) nina

(Haliday, 1838)

Alysia nina Haliday, 1838

##### Distribution

Scotland

##### Notes

Not listed in Huddleston (1978), although described from Scottish material (Achterberg 1998).

#### Phaenocarpa (Phaenocarpa) notabilis

Stelfox, 1944

##### Distribution

Scotland, Ireland

#### Phaenocarpa (Phaenocarpa) picinervis

(Haliday, 1838)

Alysia picinervis Haliday, 1838
americana Ashmead, 1889

##### Distribution

England, Scotland, Ireland

#### Phaenocarpa (Phaenocarpa) pratellae

(Curtis, 1826)

Alysia pratellae Curtis, 1826
piceator (Zetterstedt, 1838, *Alysia*)
psalliotae Telenga, 1935; synonymy by Achterberg (2014)

##### Distribution

England, Scotland

#### Phaenocarpa (Phaenocarpa) punctigera

(Haliday, 1838)

Alysia punctigera Haliday, 1838

##### Distribution

England, Ireland

##### Notes

Listed as a species of *Idiolexis* in Fauna Europaea.

#### Phaenocarpa (Phaenocarpa) ruficeps

(Nees, 1812)

Bassus ruficeps Nees, 1812
testacea (Nees, 1812, *Bassus*)
gracilis (Curtis, 1826, *Alysia*)
pallida (Curtis, 1826, *Alysia*)
agricolator (Zetterstedt, 1838, *Alysia*)
oculator (Ratzeburg, 1848, *Alysia*)
rubriceps (Provancher, 1883, *Alysia*) preocc.
rubricepes Provancher, 1888
testaceipes (Cameron, 1905, *Holcalysia*)
divergens Fischer, 1975
fervida Fischer, 1975
incerta Fischer, 1975
meritoria Papp, 1981
ferga Papp, 1982

##### Distribution

England, Scotland, Wales, Ireland, Isle of Man

#### Phaenocarpa (Phaenocarpa) tacita

Stelfox, 1941

caucasica Gurasashvili, 1983 preocc.
caucasicola Tobias, 1986

##### Distribution

Ireland

#### Phaenocarpa (Phaenocarpa) trisulcata

Stelfox, 1950

##### Distribution

Ireland

#### Syncrasis

Förster, 1863

PHAENOLYTA Förster, 1863

#### Syncrasis fucicola

(Haliday, 1838)

Alysia fucicola Haliday, 1838

##### Distribution

Ireland

#### Syncrasis halidayi

(Förster, 1863)

Phaenolyta halidayi Förster, 1863
fuscipes preocc., unavailable

##### Distribution

England, Ireland

#### Tanycarpa

Förster, 1863

ACROBELA Förster, 1863; synonymy by Wharton (2002)
EPICLISTA Förster, 1863
HYPOSTROPHA Förster, 1863

#### Tanycarpa bicolor

(Nees, 1812)

Bassus bicolor Nees, 1812
ancilla (Haliday, 1838, *Alysia*)

##### Distribution

England, Ireland

#### Tanycarpa gracilicornis

(Nees, 1812)

Bassus gracilicornis Nees, 1812

##### Distribution

Ireland

#### Tanycarpa mitis

Stelfox, 1941

##### Distribution

England, Ireland

#### Tanycarpa punctata

van Achterberg, 1976

#### Tanycarpa rufinotata

(Haliday, 1838)

Alysia rufinotata Haliday, 1838
carinata (Förster, 1863, *Acrobela*)
erythrogaster (Förster, 1863, *Epiclista*)
foersteri (Shenefelt, 1974, *Alysia*)

##### Distribution

England, Ireland

#### Trachyusa

Ruthe, 1854

COSMIOCARPA Förster, 1863

##### Notes

Distribution data from Achterberg and O'Connor (1990) and the collections of NMS.

#### Trachyusa aurora

(Haliday, 1838)

Alysia aurora Haliday, 1838
nigriceps Ruthe, 1854

##### Distribution

England, Ireland

#### Trachyusa nigrothoracica

van Achterberg & O’Connor, 1990

##### Distribution

England, Ireland

##### Notes

added by Achterberg and O'Connor (1990)

#### Dacnusini

Förster, 1863

##### Notes

The classification of the Dacnusini was revised by Griffiths (Griffiths 1964, Griffiths 1967a, Griffiths 1967b, Griffiths 1967c, Griffiths 1968a, Griffiths 1968b) in a pioneering treatment that was one of the first applications of explicit phylogenetic methods in taxonomy. It was also unusual in being based on extensive reared material. Griffiths’ classification is followed here although one of the consequences of his strict adherence to phylogenetic principles is that *Chorebus* and *Dacnusa* are very large genera. Also, as Griffiths realised, the definition of *Exotela* is unsatisfactory as it lacks clear apomorphic characters. This group of insects had previously been revised by Nixon (Nixon 1943, Nixon 1944, Nixon 1945, Nixon 1946, Nixon 1948, Nixon 1949, Nixon 1954), who recognised a greater number of genera, and some authors (Tobias 1986, Tobias 1998, Perepechayenko 2000, Fischer et al. 2004) have resurrected Nixon’s genera or used them as subgenera. This seems a retrograde step given Griffiths’ clear statements of phylogenetic hypotheses and is not used here. In both *Chorebus* and *Dacnusa*, Griffiths defined a series of species groups that with further research should form the basis of better classifications of these genera. Griffiths (1964) gives a key to genera and Wharton (1997) is also very helpful. The *Coelinius* genus group (*Coelinius*, *Trachionus*, *Epimicta*, *Aristelix*, *Laotris*, *Sarops* and *Synelix*) is generally agreed to be monophyletic and was not revised at species level by Nixon or Griffiths. Distribution data from Griffiths’ and Nixon’s revisions (loc. cit.) and NMS.

#### Amyras

Nixon, 1943

#### Amyras clandestina

(Haliday, 1839)

Alysia clandestina Haliday, 1839
quadridentata (Thomson, 1895, *Dacnusa*)

##### Distribution

Ireland

#### Aristelix

Nixon, 1943

#### Aristelix phaenicura

(Haliday, 1839)

Alysia phaenicura Haliday, 1839
phoenicura misspelling

##### Distribution

England, Ireland

#### Chaenusa

Haliday, 1839

CHOREBIDEA Viereck, 1914
CHOREBIDEA Nixon, 1943 preocc.
CHOREBIDELLA Riegel, 1947

##### Notes

No recent revision of this genus.

#### Chaenusa conjungens

(Nees, 1811)

Bracon conjungens Nees, 1811
conjugens misspelling

##### Distribution

England, Scotland, Ireland

#### Chaenusa elongata

Stelfox, 1957

##### Distribution

Ireland

#### Chaenusa limoniadum

(Marshall, 1896)

Chorebus limoniadum Marshall, 1896

##### Distribution

England

##### Notes

Transferred from *Chorebus* by Perepechayenko (2000).

#### Chaenusa lymphata

(Haliday, 1839)

Alysia lymphata Haliday, 1839

##### Distribution

England, Ireland

##### Notes

Transferred from *Chorebus* by Achterberg (1997).

#### Chaenusa naiadum

(Haliday, 1839)

Alysia naiadum Haliday, 1839
naiadum (Curtis, 1837, *Chorebus*) nom. nud.

##### Distribution

England, Scotland, Ireland

#### Chaenusa nereidum

(Haliday, 1839)

Alysia nereidum Haliday, 1839
nereidum (Curtis, 1837, *Chorebus*) nom. nud.

##### Distribution

England, Ireland

#### Chaenusa opaca

Stelfox, 1957

##### Distribution

Ireland

#### Chorebus

Haliday, 1833

AMETRIA Förster, 1863
GYROCAMPA Förster, 1863
PHAENOLEXIS Förster, 1863
STIPHROCERA Förster, 1863
DIPLUSIA Ruthe, 1882
ETRIPTES Nixon, 1943
PARAGYROCAMPA Tobias, 1962

#### Chorebus abaris

(Nixon, 1943)

Dacnusa abaris Nixon, 1943

##### Distribution

England, Scotland

#### Chorebus abnormiceps

(Nixon, 1943)

Dacnusa abnormiceps Nixon, 1943
quadriceps (Nixon, 1941, *Dacnusa*) preocc.

##### Distribution

England

#### Chorebus agraules

(Nixon, 1945)

Dacnusa agraules Nixon, 1945

##### Distribution

England

#### Chorebus albipes

(Haliday, 1839)

Alysia albipes Haliday, 1839

##### Distribution

England, Scotland, Ireland

#### Chorebus alecto

(Morley, 1924)

Rhizarcha alecto Morley, 1924
turissa (Nixon, 1937, *Dacnusa*)

##### Distribution

England, Scotland, Ireland

#### Chorebus alua

(Nixon, 1944)

Dacnusa alua Nixon, 1944

##### Distribution

Ireland

#### Chorebus amasis

(Nixon, 1945)

Dacnusa amasis Nixon, 1945

##### Distribution

England

#### Chorebus ampliator

(Nees, 1834)

Alysia ampliator Nees, 1834
nigricornis (Förster, 1863, *Stiphrocera*)

##### Distribution

England, Scotland, Ireland

#### Chorebus anasellus

(Stelfox, 1952)

Dacnusa anasella Stelfox, 1952

##### Distribution

Scotland, Ireland

#### Chorebus angelicae

(Nixon, 1945)

Dacnusa angelicae Nixon, 1945

##### Distribution

England

#### Chorebus anita

(Nixon, 1943)

Dacnusa anita Nixon, 1943

##### Distribution

England, Scotland

#### Chorebus aphantus

(Marshall, 1895)

Dacnusa aphanta Marshall, 1895

##### Distribution

England, Scotland, Wales, Ireland

#### Chorebus apollyon

(Morley, 1924)

Dacnusa apollyon Morley, 1924

##### Distribution

England, Ireland

#### Chorebus ares

(Nixon, 1944)

Dacnusa ares Nixon, 1944

##### Distribution

England

#### Chorebus armida

(Nixon, 1945)

Dacnusa armida Nixon, 1945

##### Distribution

England, Scotland, Wales

#### Chorebus artemisiellus

Griffiths, 1968

##### Distribution

England

#### Chorebus asramenes

(Nixon, 1943)

Dacnusa asramenes Nixon, 1943

##### Distribution

England, Ireland

#### Chorebus avesta

(Nixon, 1944)

Dacnusa avesta Nixon, 1944

##### Distribution

England, Scotland

#### Chorebus bathyzonus

(Marshall, 1895)

Dacnusa bathyzona Marshall, 1895
ornatus (Telenga, 1935, *Dacnusa*)

##### Distribution

England, Ireland

#### Chorebus bensoni

(Nixon, 1943)

Dacnusa bensoni Nixon, 1943

##### Distribution

England, Scotland

#### Chorebus bres

(Nixon, 1944)

Dacnusa bres Nixon, 1944

##### Distribution

England, Ireland

#### Chorebus brevicornis

(Thomson, 1895)

Dacnusa brevicornis Thomson, 1895
chrysippe (Nixon, 1944, *Dacnusa*)
ea (Nixon, 1944, *Dacnusa*)

##### Distribution

England, Scotland, Ireland

#### Chorebus caelebs

(Nixon, 1944)

Dacnusa caelebs Nixon, 1944

##### Distribution

England

#### Chorebus calthae

Griffiths, 1967

##### Distribution

England

#### Chorebus cambricus

Griffiths, 1968

##### Distribution

Wales

#### Chorebus cinctus

(Haliday, 1839)

Alysia cincta Haliday, 1839
castaneiventris (Thomson, 1895, *Dacnusa*)

##### Distribution

England, Ireland

#### Chorebus coxator

(Thomson, 1895)

Dacnusa coxator Thomson, 1895

##### Distribution

England, Scotland, Wales

#### Chorebus crassipes

(Stelfox, 1954)

Dacnusa crassipes Stelfox, 1954

##### Distribution

Scotland, Ireland

#### Chorebus credne

(Nixon, 1944)

Dacnusa credne Nixon, 1944

##### Distribution

England, Scotland, Ireland

#### Chorebus crenulatus

(Thomson, 1895)

Dacnusa crenulata Thomson, 1895
elegantulus (Nixon, 1937, *Dacnusa*)

##### Distribution

England, Scotland, Ireland

#### Chorebus crocale

(Nixon, 1945)

Dacnusa crocale Nixon, 1945

##### Distribution

England

#### Chorebus cubocephalus

(Telenga, 1934)

Rhizarcha cubocephala Telenga, 1934
cyclops (Nixon, 1937, *Dacnusa*); synonymy by Tobias (1986)

##### Distribution

Ireland

#### Chorebus cylindricus

(Telenga, 1934)

Dacnusa cylindrica Telenga, 1934
cybele (Nixon, 1937, *Dacnusa*); synonymy by Tobias (1986)

##### Distribution

England, Scotland

#### Chorebus cytherea

(Nixon, 1937)

Dacnusa cytherea Nixon, 1937
calliope (Nixon, 1944, *Dacnusa*)
tesmia (Nixon, 1944, *Dacnusa*)

##### Distribution

England, Scotland, Ireland

#### Chorebus dagda

(Nixon, 1943)

Dacnusa dagda Nixon, 1943

##### Distribution

England

#### Chorebus daimenes

(Nixon, 1945)

Dacnusa daimenes Nixon, 1945

##### Distribution

England, Scotland

#### Chorebus deione

(Nixon, 1944)

Dacnusa deione Nixon, 1944

##### Distribution

England, Scotland

#### Chorebus didas

(Nixon, 1944)

Dacnusa didas Nixon, 1944

##### Distribution

England, Scotland

#### Chorebus difficilis

Griffiths, 1968

##### Distribution

England

#### Chorebus diremtus

(Nees, 1834)

Alysia diremta Nees, 1834
diremptus (Haliday, 1839, *Alysia*)

##### Distribution

England, Ireland

#### Chorebus dirona

(Nixon, 1945)

Dacnusa dirona Nixon, 1945

##### Distribution

England, Ireland

#### Chorebus enephes

(Nixon, 1945)

Dacnusa enephes Nixon, 1945

##### Distribution

England

#### Chorebus eros

(Nixon, 1937)

Dacnusa eros Nixon, 1937

##### Distribution

England, Scotland, Ireland

#### Chorebus esbelta

(Nixon, 1937)

Dacnusa esbelta Nixon, 1937

##### Distribution

England, Scotland, Ireland

#### Chorebus euryale

(Nixon, 1944)

Dacnusa euryale Nixon, 1944

##### Distribution

England

#### Chorebus fallaciosae

Griffiths, 1967

##### Distribution

England

#### Chorebus fallax

(Nixon, 1937)

Dacnusa fallax Nixon, 1937

##### Distribution

England, Scotland, Ireland

#### Chorebus flavipes

(Goureau, 1851)

Dacnusa flavipes Goureau, 1851
raissa (Nixon, 1937, *Dacnusa*)

##### Distribution

England, Scotland, Ireland

#### Chorebus fordi

(Nixon, 1954)

Dacnusa fordi Nixon, 1954

##### Distribution

England

#### Chorebus foveolus

(Haliday, 1839)

Alysia foveola Haliday, 1839

##### Distribution

England, Scotland, Wales, Ireland

#### Chorebus fuscipennis

(Nixon, 1937)

Dacnusa fuscipennis Nixon, 1937

##### Distribution

England

#### Chorebus gedanensis

(Ratzeburg, 1852)

Alysia gedanensis Ratzeburg, 1852
anguligena (Nixon, 1937, *Dacnusa*)

##### Distribution

England

#### Chorebus glaber

(Nixon, 1944)

Dacnusa glabra Nixon, 1944

##### Distribution

England, Ireland

#### Chorebus glabriculus

(Thomson, 1895)

Dacnusa glabricula Thomson, 1895
cortipalpis (Nixon, 1937, *Dacnusa*)

##### Distribution

England, Scotland, Ireland

#### Chorebus hilaris

Griffiths, 1967

##### Distribution

England

#### Chorebus hirtigena

Stelfox, 1957

##### Distribution

Ireland

#### Chorebus humeralis

Griffiths, 1968

##### Distribution

Ireland

#### Chorebus incertus

(Goureau, 1851)

Dacnusa incerta Goureau, 1851

##### Distribution

England

#### Chorebus iphias

(Nixon, 1943)

Dacnusa iphias Nixon, 1943

##### Distribution

England

#### Chorebus kama

(Nixon, 1945)

Dacnusa kama Nixon, 1945

##### Distribution

England

#### Chorebus lanigerus

(Stelfox, 1957)

Gyrocampa lanigera Stelfox, 1957

##### Distribution

Ireland

#### Chorebus lar

(Morley, 1924)

Dacnusa lar Morley, 1924
innanus (Nixon, 1943, *Dacnusa*)

##### Distribution

England

#### Chorebus larides

(Nixon, 1944)

Dacnusa larides Nixon, 1944

##### Distribution

England, Ireland

#### Chorebus lateralis

(Haliday, 1839)

Alysia lateralis Haliday, 1839
fuscula (Haliday, 1839, *Alysia*)
albicoxa (Thomson, 1895, *Dacnusa*)

##### Distribution

England, Scotland, Wales, Ireland

#### Chorebus leptogaster

(Haliday, 1839)

Alysia leptogaster Haliday, 1839
naenia (Morley, 1924, *Dacnusa*)
dinae (Burghele, 1960, *Dacnusa*)

##### Distribution

England, Ireland

#### Chorebus longicornis

(Nees, 1811)

Bracon longicornis Nees, 1811
affinis (Nees, 1812, *Bassus*)

##### Distribution

England, Scotland, Ireland

##### Notes

Synonymy first established in Shenefelt (1974) but overlooked until O'Connor et al. (1999).

#### Chorebus lugubris

(Nixon, 1937)

Dacnusa lugubris Nixon, 1937

##### Distribution

England, Ireland

#### Chorebus luzulae

Griffiths, 1966

##### Distribution

Scotland

##### Notes

added by Godfray and Bland (2011)

#### Chorebus lychnidis

Griffiths, 1967

##### Distribution

England

#### Chorebus maculigastra

Shenefelt, 1974

maculata (Nixon, 1944, *Dacnusa*) preocc.

##### Distribution

England, Ireland

#### Chorebus merellus

(Nixon, 1937)

Dacnusa merella Nixon, 1937

##### Distribution

England, Scotland, Ireland

#### Chorebus merion

(Nixon, 1945)

Dacnusa merion Nixon, 1945

##### Distribution

England

#### Chorebus miodes

(Nixon, 1949)

Gyrocampa miodes Nixon, 1949

##### Distribution

England, Ireland

#### Chorebus misellus

(Marshall, 1895)

Dacnusa misella Marshall, 1895

##### Distribution

England

#### Chorebus mitrus

(Nixon, 1945)

Dacnusa mitra Nixon, 1945

##### Distribution

England, Scotland, Ireland

#### Chorebus nanus

(Nixon, 1943)

Dacnusa nana Nixon, 1943

##### Distribution

England

#### Chorebus navicularis

(Nees, 1812)

Bassus navicularis Nees, 1812

##### Distribution

England

#### Chorebus nerissus

(Nixon, 1937)

Dacnusa nerissa Nixon, 1937

##### Distribution

England

#### Chorebus nigriscaposus

(Nixon, 1949)

Gyrocampa nigriscaposa Nixon, 1949
propodealis (Nixon, 1949, *Gyrocampa*)

##### Distribution

Ireland

#### Chorebus ninella

(Nixon, 1945)

Dacnusa ninella Nixon, 1945

##### Distribution

England, Scotland, Ireland

#### Chorebus nobilis

Griffiths, 1968

##### Distribution

England, Ireland

#### Chorebus nomia

(Nixon, 1937)

Dacnusa nomia Nixon, 1937

##### Distribution

England, Ireland

#### Chorebus nydia

(Nixon, 1937)

Dacnusa nydia Nixon, 1937

##### Distribution

England, Scotland, Ireland

#### Chorebus orbiculatae

Griffiths, 1967

##### Distribution

England, Ireland

#### Chorebus ovalis

(Marshall, 1896)

Dacnusa ovalis Marshall, 1896

##### Distribution

England, Ireland

#### Chorebus parvungula

(Thomson, 1895)

Dacnusa parvungula Thomson, 1895
acco (Nixon, 1943, *Dacnusa*)

##### Distribution

England, Scotland, Ireland

#### Chorebus perkinsi

(Nixon, 1944)

Dacnusa perkinsi Nixon, 1944

##### Distribution

England

#### Chorebus petiolatus

(Nees, 1834)

Alysia petiolata Nees, 1834

##### Distribution

England, Scotland, Ireland

#### Chorebus phaedra

(Nixon, 1937)

Dacnusa phaedra Nixon, 1937

##### Distribution

England, Scotland

#### Chorebus pimpinellae

Griffiths, 1967

##### Distribution

England

#### Chorebus pione

(Nixon, 1944)

Dacnusa pione Nixon, 1944

##### Distribution

England

#### Chorebus poemyzae

Griffiths, 1968

##### Distribution

England, Scotland

#### Chorebus posticus

(Haliday, 1839)

Alysia postica Haliday, 1839
gracilis (Nees, 1834, *Alysia*); synonymy by Achterberg (1997)
egregia (Marshall, 1895, *Dacnusa*)
dentatus (Tobias, 1962, *Dacnusa*); synonymy by Tobias (1986)

##### Distribution

England, Scotland, Ireland

#### Chorebus pulverosus

(Haliday, 1839)

Alysia pulverosa Haliday, 1839
marsyas (Nixon, 1937, *Dacnusa*)

##### Distribution

England, Ireland

#### Chorebus punctum

(Goureau, 1851)

Dacnusa punctum Goureau, 1851

##### Distribution

England, Scotland, Ireland

#### Chorebus resa

(Nixon, 1937)

Dacnusa resa Nixon, 1937

##### Distribution

England

#### Chorebus rhanis

(Nixon, 1943)

Dacnusa rhanis Nixon, 1943

##### Distribution

Scotland

#### Chorebus risilis

(Nixon, 1949)

Gyrocampa risilis Nixon, 1949

##### Distribution

Ireland

#### Chorebus rondanii

(Giard, 1904)

Dacnusa rondanii Giard, 1904
galbus (Nixon, 1944, *Dacnusa*)

##### Distribution

England

#### Chorebus rotundiventris

(Thomson, 1895)

Dacnusa rotundiventris Thomson, 1895

##### Distribution

Ireland

#### Chorebus rousseaui

(Schulz, 1907)

Dacnusa rousseaui Schulz, 1907

##### Notes

The status of this taxon needs further research.

#### Chorebus ruficollis

(Stelfox, 1957)

Gyrocampa ruficollis Stelfox, 1957

##### Distribution

Ireland

#### Chorebus rufimarginatus

(Stelfox, 1954)

Dacnusa rufimarginata Stelfox, 1954

##### Distribution

Ireland

#### Chorebus scabiosae

Griffiths, 1967

##### Distribution

England

#### Chorebus scabrifossa

Stelfox, 1957

##### Distribution

Ireland

#### Chorebus selene

(Nixon, 1937)

Dacnusa selene Nixon, 1937

##### Distribution

England, Scotland

#### Chorebus senilis

(Nees, 1812)

Bassus senilis Nees, 1812
tomentosus (Thomson, 1895, *Dacnusa*)
nemesis (Morley, 1924, *Dacnusa*)

##### Distribution

England, Scotland, Wales, Ireland

#### Chorebus serus

(Nixon, 1937)

Dacnusa sera Nixon, 1937

##### Distribution

England

#### Chorebus siniffa

(Nixon, 1937)

Dacnusa siniffa Nixon, 1937

##### Distribution

England, Ireland

#### Chorebus solstitialis

(Stelfox, 1952)

Dacnusa solstitialis Stelfox, 1952

##### Distribution

England

#### Chorebus spenceri

Griffiths, 1964

##### Distribution

England, Scotland

#### Chorebus striola

Stelfox, 1957

##### Distribution

Ireland

#### Chorebus sylvestris

Griffiths, 1967

##### Distribution

England, Scotland, Ireland

#### Chorebus talaris

(Haliday, 1839)

Alysia talaris Haliday, 1839

##### Distribution

England, Scotland, Ireland

#### Chorebus tamiris

(Nixon, 1943)

Dacnusa tamiris Nixon, 1943

##### Distribution

England

#### Chorebus tamsi

(Nixon, 1944)

Dacnusa tamsi Nixon, 1944

##### Distribution

England

#### Chorebus tanis

(Nixon, 1945)

Dacnusa tanis Nixon, 1945

##### Distribution

England

#### Chorebus tenellae

Griffiths, 1967

##### Distribution

Scotland, Ireland

##### Notes

added by Griffiths (1984)

#### Chorebus thecla

(Nixon, 1943)

Dacnusa thecla Nixon, 1943

##### Distribution

England

#### Chorebus thisbe

(Nixon, 1937)

Dacnusa thisbe Nixon, 1937

##### Distribution

England

#### Chorebus thusa

(Nixon, 1937)

Dacnusa thusa Nixon, 1937

##### Distribution

England, Ireland

#### Chorebus transversus

(Nixon, 1954)

Dacnusa transversa Nixon, 1954

##### Distribution

England, Scotland, Ireland

#### Chorebus trilobomyzae

Griffiths, 1968

##### Distribution

England, Scotland

#### Chorebus uliginosus

(Haliday, 1839)

Alysia uliginosa Haliday, 1839
thienemanni (Ruschka, 1913, *Gyrocampa*)

##### Distribution

England, Ireland

#### Chorebus uma

(Nixon, 1944)

Dacnusa uma Nixon, 1944

##### Distribution

England

#### Chorebus varuna

(Nixon, 1945)

Dacnusa varuna Nixon, 1945

##### Distribution

England, Scotland

#### Chorebus vernalis

Griffiths, 1968

##### Distribution

England

#### Chorebus vitripennis

Griffiths, 1968

##### Distribution

Scotland, Wales, Ireland

#### Coelinidea

Viereck, 1913

LEPTON Zetterstedt, 1838 preocc.
ERICOELINIUS Viereck, 1913
FISCHERASTRIOLUS Perepechayenko, 1999

##### Notes

There is no recent treatment of this genus; generic synonymy follows Achterberg (2014).

#### Coelinidea elegans

(Curtis, 1829)

Chaenon elegans Curtis, 1829
brevicornis (Curtis, 1829, *Chaenon*)
cingulatus (Curtis, 1829, *Chaenon*)
rufinotatus (Curtis, 1829, *Chaenon*)
similis (Curtis, 1829, *Chaenon*)

##### Distribution

England, Ireland

#### Coelinidea fuliginosus

(Curtis, 1829)

Chaenon fuliginosus Curtis, 1829

##### Distribution

England

#### Coelinidea gracilis

(Curtis, 1829)

Chaenon gracilis Curtis, 1829
attenuator (Zetterstedt, 1838, *Lepton*)

##### Distribution

England, Scotland, Ireland

#### Coelinidea niger

(Nees, 1811)

Stephanus niger Nees, 1811
affinis (Curtis, 1829, *Chaenon*)
nigricans (Westwood, 1835, *Chaenon*)
olivieri (Guérin-Ménéville, 1842, *Alysia*)

##### Distribution

England, Ireland

#### Coelinidea obscurus

(Curtis, 1829)

Chaenon obscurus Curtis, 1829

##### Distribution

England

#### Coelinidea podagricus

(Haliday, 1839)

Alysia podagrica Haliday, 1839

##### Distribution

Ireland

#### Coelinidea ruficollis

(Herrich-Schäffer, 1838)

Coelinius ruficollis Herrich-Schäffer, 1838
procerus (Haliday, 1839, *Alysia*)

##### Distribution

England

#### Coelinidea viduus

(Curtis, 1829)

Chaenon viduus Curtis, 1829
ater (Curtis, 1837, *Chaenon*) nom. nud.

##### Distribution

England, Ireland

#### Coelinius

Nees, 1818

CHAENON Curtis, 1829
COPISURA Schiødte,1837
COPIDURA Förster,1862

##### Notes

Griffiths (1964) proposed that *Polemochartus* and *Coelinidea* should be included as subgenera of *Coelinius*. Wharton and Austin (1991), Wharton (1994) and Kula (2008) supported the concept of an enlarged *Coelinius* but, pointing to intermediate taxa in the Oriental Region, argued against the retention of subgenera. Achterberg (2014) retains the three genera which is current European usage and is followed here pending a modern review of the group.

#### Coelinius parvulus

(Nees, 1811)

Chaenon parvulus Nees, 1811
Coelinius parvulus ?*cultriformis* (Latreille, 1802, *Ichneumon*)
circulator (Gravenhorst, 1807, *Ichneumon*) preocc.
anceps (Curtis, 1829, *Chaenon*)
rimator (Schiødte, 1837, *Copisura*)
bicarinatus Herrich-Schäffer, 1838
flexuosus Herrich-Schäffer, 1838
bicolor Maréchal, 1938

##### Distribution

England, Scotland, Ireland, Isle of Man

#### Coloneura

Förster, 1863

ISOMERISTA Förster, 1863
TRISISA Förster, 1863
MERITES Nixon, 1943
PRIAPSIS Nixon, 1943

#### Coloneura dice

(Nixon, 1943)

Priapsis dice Nixon, 1943

##### Distribution

England

#### Coloneura stylata

Förster, 1863

exilis (Förster, 1863, *Trisisa*)
oligomera (Förster, 1863, *Isomerista*)
taras (Nixon, 1943, *Merites*)

##### Distribution

England, Ireland

#### Dacnusa

Haliday, 1833

AGONIA Förster, 1863
APHANTA Förster, 1863
BRACHYSTROPHA Förster, 1863
LIPOSCIA Förster, 1863
PACHYSEMA Förster, 1863
RHIZARCHA Förster, 1863
TANYSTROPHA Förster, 1863
RADIOLARIA Provancher, 1886
COLONEURELLA van Achterberg, 1976

#### Dacnusa abdita

Haliday, 1838

incidens Thomson, 1895
lepida Marshall, 1896

##### Distribution

England, Scotland, Wales, Ireland

#### Dacnusa adducta

(Haliday, 1839)

Alysia adducta Haliday, 1839
abducta misspelling

##### Distribution

England, Scotland, Ireland

##### Notes

Based on its unusual venation, *adducta* is often placed in the monotypic genus *Agonia*; Griffiths’ (Griffiths 1964) argument that it is a derived species of *Dacnusa* is followed here.

#### Dacnusa alticeps

Nixon, 1937

##### Distribution

England, Scotland

#### Dacnusa aquilegiae

Marshall, 1896

##### Distribution

England, Scotland

#### Dacnusa areolaris

(Nees, 1811)

Bracon areolaris Nees, 1811
lysias Goureau, 1851

##### Distribution

England, Scotland, Wales, Ireland

#### Dacnusa astarte

(Nixon, 1948)

Rhizarcha astarte Nixon, 1948

##### Distribution

England

#### Dacnusa aterrima

Thomson, 1895

##### Distribution

England

#### Dacnusa confinis

Ruthe, 1859

minuta (Curtis, 1826, *Alysia*)

##### Distribution

England, Wales, Ireland

##### Notes

*Alysia minuta* was listed as a species of *Alysia* by Shenefelt (1974) and Huddleston (1978) although a junior homonym of *Alysia minuta* Nees, 1812; Wharton (1986) showed it to belong to *Dacnusa* and van Achterberg (in litt.) concluded that it is conspecific with *confinis*.

#### Dacnusa delphinii

Griffiths, 1967

##### Distribution

England

#### Dacnusa discolor

(Förster, 1863)

Liposcia discolor Förster, 1863
cercides (Nixon, 1954, *Pachysema*)

##### Distribution

England, Scotland, Ireland

#### Dacnusa dryas

(Nixon, 1948)

Rhizarcha dryas Nixon, 1948

##### Distribution

England

#### Dacnusa ergeteles

(Nixon, 1954)

Pachysema ergeteles Nixon, 1954

##### Distribution

Ireland

#### Dacnusa euphrasiella

Griffiths, 1984

##### Distribution

Ireland

##### Notes

added by Griffiths (1984)

#### Dacnusa evadne

Nixon, 1937

##### Distribution

England, Scotland, Ireland

#### Dacnusa faeroeensis

(Roman, 1917)

Rhizarcha faeroeensis Roman, 1917
lestes Nixon, 1937

##### Distribution

England, Scotland, Wales, Ireland

#### Dacnusa fasciata

Stelfox, 1954

##### Distribution

Ireland

#### Dacnusa hospita

(Förster, 1863)

Aphanta hospita Förster, 1863

##### Distribution

England, Scotland, Ireland

#### Dacnusa laeta

(Nixon, 1954)

Pachysema laeta Nixon, 1954

##### Distribution

England, Scotland, Ireland

#### Dacnusa laevipectus

Thomson, 1895

nox (Morley, 1924, *Rhizarcha*)

##### Distribution

England, Scotland, Wales, Ireland

#### Dacnusa lissos

(Nixon, 1954)

Pachysema lissos Nixon, 1954

##### Distribution

England

#### Dacnusa longiradialis

Nixon, 1937

##### Distribution

England, Scotland, Ireland

#### Dacnusa lugens

(Haliday, 1839)

Alysia lugens Haliday, 1839

##### Distribution

England, Ireland

#### Dacnusa macrospila

(Haliday, 1839)

Alysia macrospila Haliday, 1839

##### Distribution

England, Scotland, Ireland

#### Dacnusa maculipes

Thomson, 1895

##### Distribution

England, Scotland, Wales, Ireland

#### Dacnusa maxima

(Fischer, 1961)

Pachysema maxima Fischer, 1961

##### Distribution

Wales

#### Dacnusa melicerta

(Nixon, 1954)

Pachysema melicerta Nixon, 1954
fumipes Tobias, 1998

##### Distribution

England, Scotland, Ireland

#### Dacnusa merope

(Nixon, 1948)

Rhizarcha merope Nixon, 1948

##### Distribution

England

#### Dacnusa metula

(Nixon, 1954)

Pachysema metula Nixon, 1954

##### Distribution

England, Scotland, Ireland

#### Dacnusa monticola

(Förster, 1863)

Brachystropha monticola Förster, 1863
mutia (Nixon, 1948, *Rhizarcha*)
coracina Stelfox, 1957

##### Distribution

Ireland

#### Dacnusa nigrella

Griffiths, 1967

##### Distribution

Scotland

##### Notes

added by Godfray and Bland (2011)

#### Dacnusa nigropygmaea

Stelfox, 1954

##### Distribution

Ireland

#### Dacnusa obesa

Stelfox, 1954

##### Distribution

Scotland, Ireland

#### Dacnusa ocyroe

Nixon, 1937

##### Distribution

England, Scotland, Wales, Ireland

#### Dacnusa plantaginis

Griffiths, 1967

discolor misident.

##### Distribution

England, Scotland, Wales, Ireland

#### Dacnusa pubescens

(Curtis, 1826)

Alysia pubescens Curtis, 1826
exserens (Nees, 1834, *Alysia*)

##### Distribution

England, Scotland, Ireland

#### Dacnusa sibirica

Telenga, 1934

##### Distribution

England, Scotland, Wales, Ireland

#### Dacnusa comis

(Nixon, 1954)

Pachysema comis Nixon, 1954

#### Dacnusa soma

(Nixon, 1948)

Rhizarcha soma Nixon, 1948

##### Distribution

Scotland, Ireland

#### Dacnusa stramineipes

(Haliday, 1839)

Alysia stramineipes Haliday, 1839
haemorrhoa (Förster, 1863, *Tanystropha*)
longicauda Thomson, 1895

##### Distribution

England, Scotland, Ireland

##### Notes

*Dacnusa longicauda* was synonymised by Nixon (1937) although this was overlooked by Huddleston (1978).

#### Dacnusa tarsalis

Thomson, 1895

nitetis (Nixon, 1948, *Rhizarcha*)

##### Distribution

England, Scotland, Wales, Ireland

#### Dacnusa temula

(Haliday, 1839)

Alysia temula Haliday, 1839

##### Distribution

England, Scotland, Ireland

#### Dacnusa veronicae

Griffiths, 1967

##### Distribution

England, Scotland

#### Epimicta

Förster, 1863

#### Epimicta marginalis

(Haliday, 1839)

Alysia marginalis Haliday, 1839

##### Distribution

England, Ireland

#### Exotela

Förster, 1863

MESORA Förster, 1863
TOXELEA Nixon, 1943
ANTRUSA Nixon, 1943

##### Notes

Griffiths’ (Griffiths 1964, Griffiths 1967a, Griffiths 1967b, Griffiths 1967c, Griffiths 1968a, Griffiths 1968b) concept of *Exotela* is followed here; the most plesiomorphic species (*flavicoxa*, *interstitialis*, *melanocera* and *vaenia*) are often placed in *Antrusa* (e.g. Achterberg 2014).

#### Exotela cyclogaster

Förster, 1863

bellina (Nixon, 1937, *Dacnusa*)
umbellina (Nixon, 1954, *Toxelea*)
sonchina Griffiths, 1967

##### Distribution

England, Scotland, Wales, Ireland

##### Notes

Griffiths (1967c) divided *cyclogaster* into three subspecies, nominate *cyclogaster*, *umbellina* and *sonchina*. Tobias (1986) later treated these as separate species although Godfray (1984) argued that *umbellina* and *cyclogaster* are host race variants. Pending further research they are treated here as a single taxon.

#### Exotela dives

(Nixon, 1954)

Toxelea dives Nixon, 1954

##### Distribution

Ireland

#### Exotela flavicoxa

(Thomson, 1895)

Dacnusa flavicoxa Thomson, 1895

##### Distribution

England, Scotland, Ireland

#### Exotela gilvipes

(Haliday, 1839)

Alysia gilvipes Haliday, 1839
albilabris (Thomson, 1895, *Dacnusa*)

##### Distribution

England, Scotland, Ireland

#### Exotela hera

(Nixon, 1937)

Dacnusa hera Nixon, 1937

##### Distribution

England, Scotland, Isle of Man

#### Exotela interstitialis

(Thomson, 1895)

Dacnusa interstitialis Thomson, 1895
mamertes (Nixon, 1943, *Dacnusa*)

##### Notes

Placed in *Chorebus* by Tobias and Jakimavicius (1973) and in *Antrusa* by Papp (2007); needs further research.

#### Exotela lonicerae

Griffiths, 1967

##### Distribution

England, Scotland

#### Exotela melanocera

(Thomson, 1895)

Dacnusa melanocera Thomson, 1895
persimilis (Nixon, 1954, *Antrusa*)

##### Distribution

England, Ireland

#### Exotela phryne

(Nixon, 1954)

Toxelea phryne Nixon, 1954

##### Distribution

England, Scotland

#### Exotela spinifer

(Nixon, 1954)

Toxelea spinifer Nixon, 1954

##### Distribution

England, Scotland, Ireland

#### Exotela sulcata

(Tobias, 1962)

Pachysema sulcata Tobias, 1962

##### Distribution

England, Scotland, Ireland

#### Exotela vaenia

(Nixon, 1954)

Antrusa vaenia Nixon, 1954

##### Distribution

England

#### Exotela viciae

Griffiths, 1984

##### Distribution

England

##### Notes

added by Griffiths (1984)

#### Laotris

Nixon, 1943

#### Laotris striatula

(Haliday, 1839)

Alysia striatula Haliday, 1839

##### Distribution

England, Scotland, Ireland

#### Polemochartus

Nees, 1818

#### Polemochartus liparae

(Giraud, 1863)

Polemon liparae Giraud, 1863

##### Distribution

England

#### Polemochartus melas

(Giraud, 1863)

Polemon melas Giraud, 1863

##### Distribution

England

##### Notes

added by Shaw and Jennings (2008)

#### Protodacnusa

Griffiths, 1964

#### Protodacnusa litoralis

Griffiths, 1964

##### Distribution

Ireland

#### Protodacnusa tristis

(Nees, 1834)

Alysia tristis Nees, 1834
ampliator (Haliday, 1839, *Alysia*) preocc.
longistigma (Telenga, 1935, *Dacnusa*)

##### Distribution

England, Ireland

#### Sarops

Nixon, 1942

#### Sarops rea

Nixon, 1942

##### Distribution

England

#### Synelix

Förster, 1863

ECTILIS Nixon, 1943

#### Synelix semirugosa

(Haliday, 1839)

Alysia semirugosa Haliday, 1839
agnata Förster, 1863
amaurosomae (Telenga, 1935, *Dacnusa*)

##### Distribution

England, Scotland, Wales, Ireland

#### Tates

Nixon, 1943

#### Tates heterocera

(Thomson, 1895)

Dacnusa heterocera Thomson, 1895

##### Distribution

England, Scotland

#### Trachionus

Haliday, 1833

#### Trachionus

Haliday, 1833

AENONE Curtis, 1837 preocc.
AENONE Haliday, 1838 preocc., nom. nud.
OENONE Haliday, 1839 preocc.
SYMPHYA Förster, 1863
ANARMUS Ruthe, 1882

##### Notes

Generic synonymy follows Achterberg (1997).

#### Trachionus (Trachionus) mandibularis

(Nees, 1816)

Sigalphus mandibularis Nees, 1816

##### Distribution

England, Wales, Ireland

#### Planiricus

Perepechayenko, 2000

#### Trachionus (Planiricus) hians

(Nees, 1816)

Sigalphus hians Nees, 1816

##### Distribution

England, Scotland, Ireland

#### Trachionus (Planiricus) ringens

(Haliday, 1839)

Alysia ringens Haliday, 1839

##### Distribution

England, Scotland, Ireland

### Aphidiinae

#### Aphidiinae

Haliday, 1833

##### Notes

In the older literature (and some very recent literature) often treated as a separate family (i.e. Aphidiidae) within Ichneumonoidea. Some distribution data from Mackauer (1961), Starý (1978) and Baker (2013).

#### Aphidiini

Haliday, 1833

#### Adialytus

Förster, 1863

#### Adialytus ambiguus

(Haliday, 1834)

Aphidius ambiguus Haliday, 1834
diminuens (Nees, 1834, *Aphidius*)
Adialytus ambiguus ?*exiguus* (Haliday, 1834, *Aphidius*)
delhiensis (Subba Rao & Sharma, 1960, *Aphidius*)
arvicola (Starý, 1961, *Lysiphlebus*)
mackaueri (Starý, 1961, *Lysiphlebus*)
crocinus (Mackauer, 1962, *Lysiphlebus*)

##### Distribution

Scotland, Wales

##### Notes

The type of *exiguus* is lost (Achterberg 1997).

#### Adialytus salicaphis

(Fitch, 1855)

Trioxys salicaphis Fitch, 1855
populaphis (Fitch, 1855, *Trioxys*)
tenuis Förster, 1863
salicaphidis (Ashmead, 1889, *Lipolexis*)
laticephalus (Telenga, 1953, *Aphidius*)

##### Distribution

England, Wales

##### Notes

added by Baker and Broad (2009)

#### Aphidius

Nees, 1818

INCUBUS Schrank, 1802 nom. ob.
THERACMION Holmgren, 1872
EUAPHIDIUS Mackauer, 1961

##### Notes

doubtfully placed species of *Aphidius*:

[*constrictus* (Nees, 1811, *Bracon*)] Included as a British species by Lyle (1933) on the basis of Szépligeti’s (Szépligeti 1904) Palaearctic catalogue; the type is lost and the species has not been interpreted by recent authors.

[*dimidiatus* Curtis, 1831]

[*pallidinotus* Haliday, 1834 nom. nud.]

[*pseudoplatanus* Curtis, 1837; *constrictus* misident.] There have been no references to this species other than catalogue listings (Smith 1853, Marshall 1872). Although proposed (Curtis 1837) as a name for the species called *Aphidius constrictus* by Haliday, it has not been interpreted by recent authors.

[*viminalis* Haliday, 1834 nom. nud.]

#### Aphidius absinthii

Marshall, 1896

##### Distribution

England

##### Notes

Treated as a valid species of *Aphidius* by Rakhshani et al. (2011) rather than a synonym of *asteris*.

#### Aphidius aquilus

Mackauer, 1961

sicarius Mackauer, 1961
callipterinellae (Takada, 1966, *Lysaphidus*)

##### Distribution

England, Wales

#### Aphidius asteris

Haliday, 1834

melanocephalus (Nees, 1811, *Bracon*)
lutescens Haliday, 1834
commodus Gahan, 1926
artemisiae Ivanov, 1927

##### Distribution

England, Wales

#### Aphidius avenae

Haliday, 1834

picipes (Nees, 1811, *Bracon*) suppressed
crithmi Marshall, 1896
granarius Marshall, 1896
pascuorum Marshall, 1896
hungaricus (Györfi, 1958, *Lysiphlebus*)
caraganae Starý, 1963

##### Distribution

England, Wales, Ireland

#### Aphidius cingulatus

Ruthe, 1859

arcticus (Holmgren, 1872, *Theracmion*)
gregarius Marshall, 1872
lachni Ashmead, 1889
pterocommae Ashmead, 1889
pterocommae Marshall, 1896 preocc.
luzhetzki Telenga, 1958

##### Distribution

England, Wales

#### Aphidius colemani

Viereck, 1912

huebrichi Brèthes, 1913
platensis Brèthes, 1913
porteri Brèthes, 1915
aphidiphilus Benoit, 1955
leroyi Benoit, 1955
transcaspicus Telenga, 1958

##### Notes

#Introduced into greenhouses for biocontrol (Starý 1975).

#### Aphidius eadyi

Starý, Gonzalez & Hall, 1980

urticae misident.

##### Distribution

England

##### Notes

added by Müller et al. (1999)

#### Aphidius eglanteriae

Haliday, 1834

#### Aphidius ervi

Haliday, 1834

infirmus (Nees, 1811, *Bracon*)
ulmi Marshall, 1896
medicaginis Marshall, 1898
fumipennis Györfi, 1958
nigrescens Mackauer, 1962
mirotarsi Starý, 1963

##### Distribution

England, Wales, Ireland

#### Aphidius fumatus

Haliday, 1834

#### Aphidius funebris

Mackauer, 1961

cirsii Ivanov, 1925 preocc.
bispinosus Telenga, 1958
eriophori Mackauer, 1967

##### Distribution

England, Wales

##### Notes

added by Pennacchio (1989)

#### Aphidius hortensis

Marshall, 1896

berberidis Smith, 1944

##### Distribution

England, Wales

#### Aphidius matricariae

Haliday, 1834

arundinis Haliday, 1834
cirsii Haliday, 1834 preocc.
phorodontis Ashmead, 1889
chrysanthemi Marshall, 1896
lychnidis Marshall, 1896
polygoni Marshall, 1896
affinis Quilis, 1931
baudysi Quilis, 1931
discrytus Quilis, 1931
merceti Quilis, 1931
obscuriformis Quilis, 1931
valentinus Quilis, 1931
renominatus Hincks, 1943
nigriteleus Smith, 1944

##### Distribution

England, Wales, Ireland

#### Aphidius microlophii

Pennacchio & Tremblay, 1987

##### Distribution

England, Wales

##### Notes

Added by Pennacchio (1989); also recorded by Müller et al. (1999).

#### Aphidius rhopalosiphi

de Stefani-Perez, 1902

equiseticola Starý, 1963
poacearum Starý, 1963

##### Distribution

England

#### Aphidius ribis

Haliday, 1834

ribaphidis (Ashmead, 1889, *Lysiphlebus*)
scabiosae Marshall, 1896
ribis Ashmead, 1898 preocc.

##### Distribution

England

#### Aphidius rosae

Haliday, 1833

aphidum (Linnaeus, 1758, *Ichneumon*) nom. ob.
aphidator (Thunberg, 1824, *Ichneumon*) nom. ob.
rosae Curtis, 1831 nom. nud.
protaeus Wesmael, 1835
rosarum Nees, 1834
xanthostoma Bouché, 1834
protaeus Wesmael, 1835
cancellatus Buckton, 1876

##### Distribution

England, Wales, Ireland

#### Aphidius rubi

Starý, 1962

##### Distribution

England

##### Notes

Added by Müller et al. (1999); Belshaw (appendix in Müller et al. 1999) demonstrates that this species should be treated as separate from *urticae*.

#### Aphidius salicis

Haliday, 1834

restrictus Nees, 1834
duodecimarticulatus Ratzeburg, 1852
dauci Marshall, 1896

##### Distribution

England, Wales

#### Aphidius setiger

(Mackauer, 1961)

Euaphidius setiger Mackauer, 1961
aceri Ivanov, 1925

##### Distribution

Wales

##### Notes

added by Baker and Broad (2009)

#### Aphidius sonchi

Marshall, 1896

##### Distribution

England, Wales

#### Aphidius tanacetarius

Mackauer, 1962

tanaceti Curtis, 1837 nom. nud.
tanaceticola Starý, 1963

##### Notes

Belokobylskij et al. (2003) treat *tanacetarius* as the valid name for *tanaceti*, which is a nomen nudum. This species has not been listed as British since its original description (Curtis 1837) and Smith’s (Smith 1853) catalogue. Pungerl (1986) treated *tanacetarius* as a valid species but did not see any British or Irish material.

#### Aphidius urticae

Haliday, 1834

euphorbiae Marshall, 1896
longulus Marshall, 1896
lonicerae Marshall, 1896
silenes Marshall, 1896
goidanichi Quilis, 1932
ivanovae Telenga, 1958
rubi Starý, 1962
silvaticus Starý, 1962
aulacorthi Starý, 1963

##### Distribution

England

#### Aphidius uzbekistanicus

Luzhetski, 1960

urticae misident.; Powell (1982)
Aphidius uzbekistanicus ?*beltrani* Quilis, 1931
Aphidius uzbekistanicus ?*indivisus* Quilis, 1931
Aphidius uzbekistanicus ?*macropterus* Quilis, 1931
Aphidius uzbekistanicus ?*pailloti* Quilis, 1931
impressus Mackauer, 1965

##### Distribution

England

##### Notes

*Aphidius beltrani* is a possible senior synonym of *uzbekistanicus* (Starý 1973).

#### Betuloxys

Mackauer, 1960

#### Betuloxys compressicornis

(Ruthe, 1859)

Trioxys compressicornis Ruthe, 1859
testaceus (Stelfox, 1948, *Trioxys*)

##### Distribution

England, Wales, Ireland

#### Binodoxys

Mackauer, 1960

MISAPHIDUS Rondani, 1848 nom. ob.

##### Notes

In Belokobylskij et al. (2003), van Achterberg listed these species under the genus name *Misaphidus* but, according to van Achterberg (pers. comm.), *Misaphidus* Rondani, 1848 *s.l.* (type-species: *Misaphidus crudelis* Rondani, 1848 (= *Aphidius centaureae* Haliday, 1833)) is a nomen oblitum according to Article 23.9 of ICZN.

#### Binodoxys acalephae

(Marshall, 1896)

Aphidius acalephae Marshall, 1896
rietscheli (Mackauer, 1959, *Trioxys*)
urticae (Mackauer, 1959, *Trioxys*)

##### Distribution

England

#### Binodoxys angelicae

(Haliday, 1833)

Aphidius angelicae Haliday, 1833
placidus (Gautier, 1922, *Trioxys*)
boscai (Quilis, 1931, *Trioxys*)
fumariae (Quilis, 1931, *Trioxys*)
granatensis (Quilis, 1931, *Trioxys*)
obscuriformis (Quilis, 1931, *Trioxys*)
amoplanus (Quilis, 1934, *Trioxys*)
mediterraneus (Mackauer, 1960, *Trioxys*)
wollastonii (Cabrera, 1962, *Trioxys*) nom. nud.
sikkimensis (Raychaudhuri, Samanta, Pramanik, Tamili & Sarkar, 1990, *Trioxys*)

##### Distribution

Wales

#### Binodoxys brevicornis

(Haliday, 1833)

Aphidius brevicornis Haliday, 1833
minutus (Haliday, 1833, *Aphidius*)

##### Distribution

Wales, Ireland

#### Binodoxys centaureae

(Haliday, 1833)

Aphidius centaureae Haliday, 1833
crudelis (Rondani, 1848, *Misaphidus*)
orientalis (Starý & Schlinger, 1967, *Trioxys*)
uroleucon Takada & Rishi, 1980

##### Distribution

England, Scotland, Wales, Ireland

#### Binodoxys heraclei

(Haliday, 1833)

Aphidius heraclei Haliday, 1833
obsoletus (Wesmael, 1835, *Aphidius*)
variegator (Szépligeti, 1898, *Trioxys*)

##### Distribution

Wales

#### Binodoxys letifer

(Haliday, 1833)

Aphidius letifer Haliday, 1833

##### Distribution

Ireland

#### Diaeretellus

Starý, 1960

#### Diaeretellus ephippium

(Haliday, 1834)

Aphidius ephippium Haliday, 1834

##### Distribution

England, Ireland

#### Diaeretiella

Starý, 1960

#### Diaeretiella rapae

(McIntosh, 1855)

Aphidius rapae McIntosh, 1855
vulgaris (Bouché, 1834, *Aphidius*)
rapae (Curtis, 1860, *Aphidius*) preocc.
chenopodii (Förster, 1867, *Diaeretus*) nom. nud.
halticae (Rondani, 1877, *Misaphidus*)
piceus (Cresson, 1879, *Trioxys*)
chenopodiaphidis (Ashmead, 1889, *Lipolexis*)
ferruginipes (Ashmead, 1890, *Diaeretus*) nom. nud.
brassicae (Marshall, 1896, *Aphidius*)

##### Distribution

England, Wales

#### Diaeretus

Förster, 1863

#### Diaeretus leucopterus

(Haliday, 1834)

Aphidius leucopterus Haliday, 1834
exspectatus (Gautier & Bonnamour, 1936, *Aphidius*)

##### Distribution

England, Wales

#### Harkeria

Cameron, 1900

PARAMONOCTONUS Starý, 1959

#### Harkeria rufa

Cameron, 1900

##### Distribution

England

##### Notes

Distribution from Achterberg (1989), who elevated *Harkeria* from a subgenus of *Monoctonus*.

#### Lipolexis

Förster, 1863

GYNOCRYPTUS Quilis, 1931

#### Lipolexis gracilis

Förster, 1863

palpator (Gautier & Bonnamour, 1931, *Aphidius*)
pieltaini (Quilis, 1931, *Gynocryptus*)
chinensis Chen, 1980

##### Distribution

England

##### Notes

BMNH, det. Torrance & Broad, added here

#### Lysaphidus

Smith, 1944

#### Lysaphidus schimitscheki

Starý, 1960

##### Distribution

England

#### Lysaphidus

Förster, 1863

#### Lysiphlebus

Förster, 1863

PLATYCYPHUS Mackauer, 1960

#### Lysiphlebus (Lysiphlebus) dissolutus

(Nees, 1811)

Bracon dissolutus Nees, 1811
macrocornis Mackauer, 1960

##### Distribution

England

#### Phlebus

Starý, 1975

APHIDARIA Provancher, 1888 preocc.

#### Lysiphlebus (Phlebus) confusus

Tremblay & Eady, 1978

##### Distribution

England, Wales

##### Notes

Added here on the basis of specimens in BMNH, identified by R.D. Eady as *ambiguus* sensu Mackauer and Starý nec Haliday, later described by Tremblay and Eady (1978) as a separate species, *confusus*, the true *ambiguus* belonging in *Adialytus*.

#### Lysiphlebus (Phlebus) fabarum

(Marshall, 1896)

Aphidius fabarum Marshall, 1896
aphidiperda (Rondani, 1877, *Misaphidus*)
monilicornis (Thomson, 1895, *Aphidius*)
cardui (Marshall, 1896, *Aphidius*)
aurantii (Pierantoni, 1907, *Aphidius*)
polygoni (Ivanov, 1927, *Aphidius*) preocc.
gomezi (Quilis, 1930, *Aphidius*)
janinii (Quilis, 1930, *Aphidius*)
inermis Quilis, 1931
innovatus Quilis, 1931
moroderi Quilis, 1931
ivanovi Mackauer, 1967

##### Distribution

England, Wales

#### Monoctonia

Starý, 1962

#### Monoctonia vesicarii

Tremblay, 1991

##### Distribution

England

##### Notes

added by Rakhshani et al. (2015)

#### Monoctonus

Haliday, 1833

#### Falciconus

Mackauer, 1961

#### Monoctonus (Falciconus) pseudoplatani

(Marshall, 1896)

Aphidius pseudoplatani Marshall, 1896

##### Distribution

England, Wales

#### Monoctonus

Haliday, 1833

#### Monoctonus (Monoctonus) caricis

(Haliday, 1833)

Aphidius caricis Haliday, 1833

##### Distribution

England, Wales

#### Monoctonus (Monoctonus) cerasi

(Marshall, 1896)

Aphidius cerasi Marshall, 1896

##### Distribution

England

#### Monoctonus (Monoctonus) crepidis

(Haliday, 1834)

Aphidius crepidis Haliday, 1834
tuberculatus (Wesmael, 1835, *Aphidius*)
paludum Marshall, 1896

##### Distribution

England, Wales

#### Monoctonus (Monoctonus) ligustri

van Achterberg, 1989

##### Distribution

England, Wales

##### Notes

added by Achterberg (1989)

#### Monoctonus (Monoctonus) nervosus

(Haliday, 1833)

Aphidius nervosus Haliday, 1833
paulensis (Ashmead, 1902, *Aphidius*)
secundus Viereck, 1915
biroi Györfi, 1958
breviantennalis Starý, 1959

#### Paralipsis

Förster, 1863

Myrmecobosca Maneval, 1940

#### Paralipsis enervis

(Nees, 1834)

Aphidius enervis Nees, 1834
mandibularis (Maneval, 1940, *Myrmecobosca*)
linnei (Hincks, 1949, *Myrmecobosca*)

##### Distribution

England, Wales

#### Pauesia

Quilis Pérez, 1931

#### Paraphidius

Starý,1958

##### Notes

species of Pauesia (Paraphidius) excluded from the British and Irish list:

[*silvestris* (Starý, 1960, *Paraphidius*)] added by Enobakhare (2001) but we do not know where Enobokhare’s material is deposited and the identification needs to be checked.

#### Pauesia (Paraphidius) abietis

(Marshall, 1896)

Aphidius abietis Marshall, 1896

##### Distribution

England

#### Pauesia (Paraphidius) cupressobii

(Starý, 1960)

Paraphidius cupressobii Starý, 1960

##### Distribution

Scotland

##### Notes

BMNH, det. Gärdenfors, added here

#### Pauesia (Paraphidius) juniperorum

(Starý, 1960)

Paraphidius juniperorum Starý, 1960

##### Distribution

England, Scotland

#### Pauesia (Paraphidius) pini

(Haliday, 1834)

Aphidius pini Haliday, 1834
planistipes (Nees,1834, *Aphidius*)
varia (Nees, 1834, *Aphidius*)
panzerii (Rondani, 1848, *Aphidius*)
lachnivorus (Ashmead, 1906, *Aphidius*)

##### Distribution

England, Scotland, Wales, Ireland

#### Pauesia

Quilis Pérez, 1931

#### Pauesia (Pauesia) infulata

(Haliday, 1834)

Aphidius infulatus Haliday, 1834
albiflagellaris (Starý, 1960, *Paraphidius*)

##### Distribution

Scotland

#### Pauesia (Pauesia) laricis

(Haliday, 1834)

Aphidius laricis Haliday, 1834

##### Distribution

Wales

#### Pauesia (Pauesia) picta

(Haliday, 1834)

Aphidius pictus Haliday, 1834

##### Distribution

Wales

#### Pauesia (Pauesia) unilachni

(Gahan, 1926)

Aphidius unilachni Gahan, 1926
albuferensis Quilis, 1931
praevisus (Gautier & Bonnamour, 1936, *Aphidius*)
basilewskyi (Benoit, 1955, *Trioxys*)

##### Distribution

England, Wales

##### Notes

added by Belshaw and Quicke (1997)

#### Trioxys

Haliday, 1833

#### Pectoxys

Mackauer, 1960

#### Trioxys (Pectoxys) macroceratus

Mackauer, 1960

#### Trioxys

Haliday, 1833

APHIDILEO Rondani, 1877
NEVROPENES Provancher, 1886
BIOXYS Starý & Schlinger, 1967

#### Trioxys (Trioxys) auctus

(Haliday, 1833)

Aphidius auctus Haliday, 1833
flaviceps Szépligeti, 1898

##### Distribution

Ireland

#### Trioxys (Trioxys) betulae

Marshall, 1896

solani Ivanov, 1925
hincksi Mackauer, 1960

##### Distribution

England, Wales

#### Trioxys (Trioxys) cirsii

(Curtis, 1831)

Aphidius cirsii Curtis, 1831
aceris (Haliday, 1833, *Aphidius*)

##### Distribution

England, Wales, Ireland

#### Trioxys (Trioxys) curvicaudus

Mackauer, 1967

##### Distribution

England, Wales

##### Notes

added by Starý (1978)

#### Trioxys (Trioxys) falcatus

Mackauer, 1959

##### Distribution

Wales

##### Notes

added by Baker and Broad (2009)

#### Trioxys (Trioxys) ibis

Mackauer, 1961

#### Trioxys (Trioxys) pallidus

(Haliday, 1833)

Aphidius pallidus Haliday, 1833
resolutus (Nees, 1834, *Aphidius*)
callipteri (Marshall, 1896, *Aphidius*)
pulcher Gautier & Bonnamour, 1924

##### Distribution

England, Wales

#### Trioxys (Trioxys) tenuicaudus

Starý, 1978

##### Distribution

Wales

##### Notes

added by Baker and Broad (2013)

#### Ephedrini

Mackauer, 1961

#### Ephedrus

Haliday, 1833

##### Notes

Synonymy and distribution data from Gärdenfors (1986).

#### Breviephedrus

Gärdenfors,1986

#### Ephedrus (Breviephedrus) brevis

Stelfox, 1941

picticornis Stelfox, 1941
niger Stelfox, 1941 nom. nud.

##### Distribution

England, Scotland, Ireland

#### Ephedrus

Haliday, 1833

ELASSUS Wesmael, 1835

#### Ephedrus (Ephedrus) cerasicola

Starý, 1962

##### Distribution

England

##### Notes

added by Van Veen et al. (2008)

#### Ephedrus (Ephedrus) helleni

Mackauer, 1968

salicicola Takada, 1968

##### Distribution

Scotland

##### Notes

added by Baker and Broad (2013)

#### Ephedrus (Ephedrus) lacertosus

(Haliday, 1833)

Aphidius lacertosus Haliday, 1833
muesebecki Smith, 1944

##### Distribution

England, Scotland, Ireland

#### Ephedrus (Ephedrus) laevicollis

(Thomson, 1895)

Aphidius laevicollis Thomson, 1895
brevicornis (Nees, 1834, *Aphidius*) preocc.
minor Stelfox, 1941
salicicola Takada, 1968

##### Distribution

Wales, Ireland

#### Ephedrus (Ephedrus) niger

Gautier, Bonnamour & Gaumont, 1929

Ephedrus (Ephedrus) niger ?*aphidivora* (Rondani, 1848, *Alysia*); synonymy by Papp (1996a)
campestris Starý, 1962

##### Distribution

England

##### Notes

added by Van Veen et al. (2008)

#### Ephedrus (Ephedrus) persicae

Froggat, 1904

nevadensis Baker, 1909
nitidus Gahan, 1917
vidali Quilis, 1931
interstitialis Watanabe, 1941
pulchellus Stelfox, 1941
impressus Granger, 1949
holmani Starý, 1958
palaestinensis Mackauer, 1959

##### Distribution

England, Ireland

#### Ephedrus (Ephedrus) plagiator

(Nees, 1811)

Bracon plagiator Nees, 1811
parcicornis (Nees, 1834, *Aphidius*)
japonicus Ashmead, 1906
homostigma Fahringer, 1934

##### Distribution

England, Scotland, Wales, Ireland

#### Lysephedrus

Starý, 1958

#### Ephedrus (Lysephedrus) validus

(Haliday, 1833)

Aphidius validus Haliday, 1833

##### Distribution

England, Ireland

#### Toxares

Haliday, 1840

TRIONYX Haliday, 1833 preocc.
TERONYX Haldeman, 1842

#### Toxares deltiger

(Haliday, 1833)

Aphidius deltiger Haliday, 1833
flaveolus (Györfi, 1958, *Ephedrus*)

##### Distribution

England, Ireland

#### Praini

Mackauer, 1961

#### Areopraon

Mackauer, 1959

MESOPRAON Starý, 1981; synonymy by Tomanovic et al. (2006)

#### Areopraon lepelleyi

(Waterston, 1926)

Praon lepelleyi Waterston, 1926

##### Distribution

England

#### Areopraon silvestre

(Starý, 1971)

Praon silvestre Starý, 1971

##### Distribution

Wales

##### Notes

Added by Baker and Broad (2013); transferred to *Areopraon* by Tomanovic et al. (2006).

#### Dyscritulus

Hincks, 1943

DYSCRITUS Marshall, 1896 preocc.

##### Notes

*Dyscritus suffolciensis* Morley, 1933 is listed by default as a species of *Dyscritulus* in Taxapad (Yu et al. 2012) as, although Starý (1959) recognised it as belonging to Euphorinae, its identity had not yet been established. Here it is synonymised under *Syntretus splendidus* (Marshall) q.v.

#### Dyscritulus planiceps

(Marshall, 1896)

Dyscritus planiceps Marshall, 1896

##### Distribution

England, Wales

#### Dyscritulus pygmaeus

Mackauer, 1961

#### Praon

Haliday, 1833

ACHORISTUS Ratzeburg, 1852
APHIDARIA Provancher, 1886
PARAPRAON Starý, 1983

##### Notes

species of *Praon* excluded from the British and Irish list:

[*simulans* (Provancher, 1886, *Aphidaria*); syn. *aguti* Smith, 1944] A Nearctic species, listed by Kloet and Hincks (1945) in error.

#### Praon abjectum

(Haliday, 1833)

Aphidius abjectum Haliday, 1833
aphidiiforme (Ratzeburg, 1852, *Bracon*)
peregrinum Ruthe, 1859

##### Distribution

England, Ireland

#### Praon absinthii

Bignell, 1894

##### Notes

Erroneously listed in Taxapad as a junior synonym of *flavinode*; see, for example, Kavallieratos et al. (2005).

#### Praon barbatum

Mackauer, 1967

##### Distribution

England

##### Notes

BMNH, det. Torrance & Broad, added here

#### Praon bicolor

Mackauer, 1959

##### Distribution

Wales

##### Notes

added by Baker and Broad (2009)

#### Praon cavariellae

Starý, 1971

##### Distribution

Scotland, Wales

##### Notes

added by Baker and Broad (2013)

#### Praon dorsale

(Haliday, 1833)

Aphidius dorsale Haliday, 1833
discolor (Nees, 1834, *Blacus*)
collare Förster, 1867 nom. nud.

##### Distribution

England, Wales, Ireland

#### Praon exsoletum

(Nees, 1811)

Bracon exsoletum Nees, 1811
palitans Muesebeck, 1956

#### Praon flavinode

(Haliday, 1833)

Aphidius flavinode Haliday, 1833
emacerator (Nees, 1834, *Blacus*)
glabrum Starý & Schlinger, 1967

##### Distribution

England, Wales

#### Praon gallicum

Starý, 1971

##### Distribution

England

##### Notes

added by Traugott et al. (2008)

#### Praon longicorne

(Marshall, 1896)

Aphidius longicorne Marshall, 1896
grossum Starý, 1971

##### Distribution

Wales

#### Praon necans

Mackauer, 1959

nympheae Subba Rao, Sarup & Sharma, 1963

##### Distribution

Wales

##### Notes

added by Baker and Broad (2013)

#### Praon spinosum

Mackauer, 1959

##### Distribution

Wales

##### Notes

added by Baker and Broad (2009)

#### Praon volucre

(Haliday, 1833)

Aphidius volucre Haliday, 1833
angulator (Nees, 1834, *Blacus*)
aphidivorum (Ratzeburg, 1844, *Aphidius*)
pruni Ivanov, 1925
breve Fahringer, 1935
mongolicum Watanabe, 1949
myzophagum Mackauer, 1959

##### Distribution

England, Wales, Ireland

#### Praon yomenae

Takada, 1968

##### Distribution

Wales

##### Notes

added by Baker and Broad (2013)

### Brachistinae

#### Brachistinae

Förster, 1863

CALYPTINAE Marshall, 1872

##### Notes

Following Belshaw and Quicke (2002), Achterberg (2003a) and Belokobylskij et al. (2003) we treat the Brachistinae as a subfamily separate from Helconinae. Sharanowski et al. (2011) expanded the limits of the Brachistinae by including the tribe Diospilini and the former subfamily Blacinae (as well as the extralimital Brulleiini). The tribes employed here are those that have been used in previous classifications under the former subfamilies, but the tribal classification remains essentially untested.

#### Blacini

Förster, 1863

##### Notes

Generally considered to be a distinct subfamily but Sharanowski et al. (2011) found that the blacines nested within the Brachistinae in their molecular phylogenetic analyses. We think it likely that these results will be upheld with further phylogenetic work and follow Sharanowski et al.’s recommended changes to the subfamily classification. Distribution and taxonomic data from Achterberg (1988a), with additional references given. Note that Tobias (1986) records B. (Neoblacus) koenigi Fischer, 1967 from England but there is no evidence that this species occurs here.

#### Blacometeorus

Tobias, 1976

#### Blacometeorus brevicauda

(Hellén, 1958)

Diospilus brevicauda Hellén, 1958

##### Distribution

England

##### Notes

added by Achterberg (1988a); Shaw (1996b)

#### Blacometeorus pusillus

(Hellén, 1958)

Diospilus pusillus Hellén, 1958

##### Distribution

England

##### Notes

added by Shaw (1996b)

#### Blacus

Nees, 1818

#### Blacus

Nees, 1818

GONIOCORMUS Förster, 1863
MIOCOLUS Förster, 1863

#### Blacus (Blacus) errans

(Nees, 1811)

Bracon errans Nees, 1811
vagans Ruthe, 1861

##### Distribution

England

#### Blacus (Blacus) exilis

(Nees, 1811)

Bracon exilis Nees, 1811
lactucaphis (Fitch, 1855, *Aphidius*)
pallipes (Förster, 1863, *Miocolus*)
pallidipes (Dalla Torre, 1898, *Miocolus*)
nanus Ashmead, 1905 nom. nud.
propallipes Shenefelt, 1969
intermedius Janzon, 1975

##### Distribution

England, Ireland

#### Blacus (Blacus) filicornis

Haeselbarth, 1973

##### Distribution

England, Ireland

#### Blacus (Blacus) forticornis

Haeselbarth, 1973

##### Distribution

England

#### Blacus (Blacus) hastatus

Haliday, 1835

terebrator Ruthe, 1861

##### Distribution

England, Scotland, Ireland

#### Blacus (Blacus) humilis

(Nees, 1811)

Bracon humilis Nees, 1811
trivialis Haliday, 1835
wesmaeli Ruthe, 1861

##### Distribution

England, Wales, Ireland

#### Blacus (Blacus) instabilis

Ruthe, 1861

petiolatus Haeselbarth, 1973 nom. nud.

##### Distribution

England, Scotland, Ireland

#### Blacus (Blacus) leptostigma

Ruthe, 1861

##### Distribution

Ireland

#### Blacus (Blacus) longipennis

(Gravenhorst, 1809)

Ophion longipenne Gravenhorst, 1809
dubius Ruthe, 1861

##### Distribution

England, Scotland, Ireland

#### Blacus (Blacus) nigricornis

Haeselbarth, 1973

##### Distribution

England, Scotland, Ireland

#### Blacus (Blacus) paganus

Haliday, 1835

brevicornis Ruthe, 1861

##### Distribution

Ireland

#### Blacus (Blacus) stelfoxi

Haeselbarth, 1973

##### Distribution

England

##### Notes

The holotype was collected in Sandhurst, Kent (Haeselbarth 1973) but not listed for Britain or Ireland by Achterberg (1988a).

#### Ganychorus

Haliday, 1835

#### Blacus (Ganychorus) ambulans

Haliday, 1835

##### Distribution

Ireland

#### Blacus (Ganychorus) armatulus

Ruthe, 1861

##### Distribution

England

#### Blacus (Ganychorus) diversicornis

Nees, 1834

compar Ruthe, 1861

##### Distribution

England, Ireland

#### Blacus (Ganychorus) macropterus

Haeselbarth, 1973

##### Distribution

England, Scotland

##### Notes

Described as a subspecies of *ambulans* by Haeselbarth (1973), but not included by Huddleston (1978), where it should have been listed as a synonym. Treated as a separate species by Achterberg (1997).

#### Blacus (Ganychorus) maculipes

Wesmael, 1835

##### Distribution

England, Scotland, Ireland

#### Blacus (Ganychorus) nitidus

Haeselbarth, 1973

petiolatus Tobias, 1976

##### Distribution

England

#### Blacus (Ganychorus) pallipes

Haliday, 1835

barynoti misident.
tuberculatus Wesmael, 1835
florus Goureau, 1851

##### Distribution

England, Ireland

##### Notes

Included, as *barynoti* (Boudier, 1834) (a junior synonym of *Pygostolus sticticus*), as a doubtfully placed species of *Blacus* by Huddleston (1978).

#### Blacus (Ganychorus) ruficornis

(Nees, 1811)

Bracon ruficornis Nees, 1811
bisstigmatus (Say, 1836, *Microgaster*)
tipulator (Zetterstedt, 1838, *Bracon*)
cerealis (Curtis, 1860, *Dacnusa*)
pallidipes (Costa, 1885, *Dinocampus*)
dentatus Hellén, 1958

##### Distribution

England, Scotland, Wales, Ireland

#### Blacus (Ganychorus) strictus

Stelfox, 1941

strictus (Curtis, 1837, *Ganychorus*) nom. nud.

##### Distribution

Scotland, Ireland

#### Blacus (Ganychorus) tripudians

Haliday, 1835

##### Distribution

England, Ireland

#### Hysterobolus

Viereck, 1913

#### Blacus (Hysterobolus) mamillanus

Ruthe, 1861

aptenodytes Marshall, 1889

##### Distribution

England

#### Brachistini

Förster, 1863

#### Eubazus

Nees, 1814

#### Aliolus

Say, 1836

#### Eubazus (Aliolus) lepidus

(Haliday, 1835)

Helcon lepidus Haliday, 1835
hofferi (Šnoflák, 1953, *Triaspis*); synonymy by Achterberg (2003a)
kusarensis (Abdinbekova, 1969, *Allodorus*); synonymy by Achterberg (2003a)

##### Distribution

England

#### Allodorus

Förster, 1863

#### Eubazus (Allodorus) convexope

van Achterberg, 2000

##### Distribution

Scotland

##### Notes

added by Achterberg (2000)

#### Eubazus (Allodorus) semirugosus

(Nees, 1816)

Sigalphus semirugosus Nees, 1816
rufipes (Herrich-Schäffer, 1838, *Eubadizon*)
tuberculator (Zetterstedt, 1838, *Bracon*)
curculionum (Hartig, 1847, *Sigalphus*)
atricornis (Ratzeburg, 1848, *Brachistes*)
mucronatus (Thomson, 1892, *Calyptus*)
truncatus (Thomson, 1892, *Calyptus*)
glabratus (Fahringer, 1941, *Calyptus*)
arete (Fahringer, 1944, *Calyptus*)

##### Distribution

England, Scotland

#### Eubazus (Allodorus) tricoloripes

van Achterberg, 2000

##### Distribution

England

##### Notes

added by Achterberg (2000)

#### Brachistes

Wesmael, 1835

#### Eubazus (Brachistes) fasciatus

(Nees, 1816)

Sigalphus fasciatus Nees, 1816
fuscipalpis (Wesmael, 1835, *Brachistes*)

##### Distribution

England, Ireland

#### Eubazus (Brachistes) minutus

(Ratzeburg, 1848)

Brachistes minutus Ratzeburg, 1848

##### Distribution

England

#### Eubazus (Brachistes) ruficoxis

(Wesmael, 1835)

Brachistes ruficoxis Wesmael, 1835
politus (Ratzeburg, 1852, *Brachistes*); synonymy by van Achterberg in Belokobylskij et al. (2003)
byctisci (Watanabe, 1933, *Calyptus*)
ruficornis misspelling

##### Distribution

England

#### Eubazus (Brachistes) segmentatus

(Marshall, 1889)

Calyptus segmentatus Marshall, 1889

##### Distribution

England

#### Eubazus (Brachistes) semicastaneus

(Marshall, 1893)

Calyptus semicastaneus Marshall, 1893

##### Distribution

England

#### Eubazus (Brachistes) tibialis

(Haliday, 1835)

Helcon tibialis Haliday, 1835
uncigenus (Wesmael, 1835, *Brachistes*)

##### Distribution

England, Scotland, Ireland

#### Calyptus

Haliday, 1835

#### Eubazus (Calyptus) macrocephalus

Nees, 1812

synchitae (Hedqvist, 1956, *Eubadizon*)
ratzeburgi (Fischer, 1962, *Eubadizon*); synonymy by van Achterberg in Belokobylskij et al. (2003)
xiphydriae Tobias, 1986; synonymy by van Achterberg in Belokobylskij et al. (2003)

##### Distribution

England

#### Eubazus (Calyptus) sigalphoides

(Marshall, 1889)

Calyptus sigalphoides Marshall, 1889

##### Distribution

England

#### Eubazus

Nees, 1814

Eubadizon Nees, 1834
Eubadizus Nees, 1834

#### Eubazus (Eubazus) flavipes

(Haliday, 1835)

Helcon flavipes Haliday, 1835
laevis (Herrich-Schäffer, 1838, *Eubadizon*)

##### Distribution

England, Ireland

#### Eubazus (Eubazus) longicauda

(Curtis, 1832)

Zele longicauda Curtis, 1832

##### Distribution

England

##### Notes

Listed as a species of *Zele* (=*Homolobus*) in Huddleston (1978). Will be synonymised under another species of *Eubazus* (van Achterberg, pers. comm.).

#### Eubazus (Eubazus) pallipes

Nees, 1814

coxalis (Nees, 1834, *Eubadizon*)
semistriatus (Haliday, 1835, *Helcon*) unavailable
americanus (Cresson, 1872, *Eubadizon*)
pallipede misspelling
pallidipes misspelling
pollipes misspelling

##### Distribution

England

#### Foersteria

Szépligeti, 1896

#### Foersteria (Foersteria) laeviuscula

Szépligeti, 1896

##### Distribution

England, Scotland

##### Notes

NMS, det. van Achterberg, added here

#### Foersteria (Foersteria) puber

(Haliday, 1835)

Helcon puber Haliday, 1835
opaca (Reinhard, 1867, *Calyptus*)
flavipes Szépligeti, 1896
talitzkii Tobias, 1961

##### Distribution

England, Ireland

#### Schizoprymnus

Förster, 1863

##### Notes

species of *Schizoprymnus* excluded from the British and Irish list:

[*nigripes* (Thomson, 1892, *Sigalphus*)] Seems to have been recorded in error by Kloet and Hincks (1945), perpetuated by Huddleston (1978).

#### Schizoprymnus ambiguus

(Nees, 1816)

Sigalphus ambiguus Nees, 1816

##### Distribution

England, Ireland

#### Schizoprymnus collaris

(Thomson, 1874)

Sigalphus collaris Thomson, 1874

##### Distribution

England

##### Notes

Added by Notton et al. (2014); listed as a species of *Triaspis* by Yu et al. (2012) but transferred back to *Schizoprymnus* by Notton et al. (2014).

#### Schizoprymnus obscurus

(Nees, 1816)

Sigalphus obscurus Nees, 1816

##### Distribution

England

#### Triaspis

Haliday, 1838

MUIRIELLA Fullaway, 1919

##### Notes

species of *Triaspis* excluded from the British and Irish list:

[*striola* (Thomson, 1874, *Sigalphus*)] Listed as a doubtfully British species in Huddleston (1978); the only record is of a tentative identification by Nixon (Hussey 1952).

#### Triaspis aciculata

(Ratzeburg, 1848)

Sigalphus aciculatus Ratzeburg, 1848

##### Distribution

England

##### Notes

BMNH, NMS, det. van Achterberg, added here; although synonymised with *obscurella* by van Achterberg (in Belokobylskij et al. 2003) this is listed as a separate species in Fauna Europaea. There are English specimens identified by van Achterberg as *aciculata* in NMS and BMNH.

#### Triaspis caledonica

(Marshall, 1888)

Sigalphus caledonicus Marshall, 1888

##### Distribution

Scotland

#### Triaspis caudata

(Nees, 1816)

Sigalphus caudatus Nees, 1816
gracilis (Herrich-Schäffer, 1840, *Sigalphus*)
australis (Szépligeti, 1901, *Sigalphus*)
arctica Hellén, 1958

##### Distribution

England, Ireland

#### Triaspis flavipalpis

(Wesmael, 1835)

Sigalphus flavipalpis Wesmael, 1835

##### Distribution

England

#### Triaspis floricola

(Wesmael, 1835)

Sigalphus floricola Wesmael, 1835
minima Snoflák, 1953

##### Distribution

England, Ireland

#### Triaspis luteipes

(Thomson, 1874)

Sigalphus luteipes Thomson, 1874

##### Distribution

England

#### Triaspis obscurella

(Nees, 1816)

Sigalphus obscurellus Nees, 1816
simulator (Szépligeti, 1901, *Sigalphus*)

##### Distribution

England

#### Triaspis pallipes

(Nees, 1816)

Sigalphus pallipes Nees, 1816
fulvipes (Haliday, 1835, *Helcon*)
fagi (Ratzeburg, 1852, *Brachistes*)
similis (Szépligeti, 1901, *Sigalphus*) preocc.
pallidipes misspelling

##### Distribution

England, Ireland, Isle of Man

##### Notes

some distribution data from Shaw and Askew (1976)

#### Triaspis podlussanyi

Papp, 1998

##### Distribution

England

##### Notes

added by Shaw & Mendel (in prep.)

#### Triaspis striatula

(Nees, 1816)

Sigalphus striatulus Nees, 1816

##### Distribution

England, Ireland

#### Triaspis thoracica

(Curtis, 1860)

Sigalphus thoracicus Curtis, 1860
gibberosa (Szépligeti, 1901, *Sigalphus*); synonymy by Papp (2004b)
rugosa (Szépligeti, 1901, *Sigalphus*); synonymy by Papp (2004b)

##### Distribution

England

#### Diospilini

Förster, 1863

#### Aspicolpus

Wesmael, 1838

ASPIDOCOLPUS Agassiz, 1846

#### Aspicolpus clipealis

(Tobias, 1967)

Aspidocolpus clipealis Tobias, 1967

##### Distribution

England

##### Notes

NMS, det. van Achterberg, added here; tentative identification; included here as a certain generic record of an *Aspicolpus* species in Britain.

#### Aspigonus

Wesmael, 1835

ASPIDOGONUS Agassiz, 1846

#### Aspigonus flavicornis

(Nees, 1834)

Bracon flavicornis Nees, 1834
diversicornis Wesmael, 1835; synonymy by van Achterberg in Belokobylskij et al. (2003)

##### Distribution

England, Wales

#### Diospilus

Haliday, 1833

BAEACIS Förster, 1878; synonymy by Achterberg (2014)
ALLOCHROMUS Marshall, 190

#### Diospilus abietis

(Ratzeburg, 1844)

Aspigonus abietis Ratzeburg, 1844

#### Diospilus capito

(Nees, 1834)

Bracon capito Nees, 1834
filator (Nees, 1834, *Bracon*) preocc.
fuscipes (Wesmael, 1835, *Taphaeus*)

##### Distribution

England, Ireland

#### Diospilus dispar

(Nees, 1811)

Bracon dispar Nees, 1811
ephippium (Nees, 1834, *Bracon*)

##### Distribution

England

#### Diospilus inflexus

Reinhard, 1862

ovatus Marshall, 1889

##### Distribution

England

#### Diospilus intermedius

(Förster, 1878)

Baeacis intermedia Förster, 1878

##### Distribution

England

##### Notes

BMNH, det. van Achterberg, added here

#### Diospilus morosus

Reinhard, 1862

##### Distribution

England

#### Diospilus nigricornis

(Wesmael, 1835)

Taphaeus nigricornis Wesmael, 1835
affinis (Wesmael, 1835, *Taphaeus*)
rufipes Reinhard, 1862

##### Distribution

England

##### Notes

Listed as *Taphaeus affinis* and *T. nigricornis* in Huddleston (1978).

#### Diospilus oleraceus

Haliday, 1833

conformis (Wesmael, 1835, *Taphaeus*)
ruficornis Szépligeti, 1896

##### Distribution

England, Ireland

#### Diospilus productus

Marshall, 1894

##### Distribution

England

#### Taphaeus

Wesmael, 1835

Anostenus Förster, 1863
Anastenus Dalla Torre, 1898

##### Notes

The systematic position of Taphaeus is uncertain. Treated as a genus of Blacinae in Fauna Europaea.

#### Taphaeus hiator

(Thunberg, 1824)

Ichneumon hiator Thunberg, 1824
irregularis Wesmael, 1835
speculator (Haliday, 1835, *Helcon*)
polydrusi (Gahan, 1916, *Diospilus*)

##### Distribution

England, Scotland, Ireland

#### Vadumasonium

Kammerer, 2006

VADUM Mason, 1987 preocc.

#### Vadumasonium vardyorum

van Achterberg & Broad, 2013

##### Distribution

England

##### Notes

added by Achterberg and Broad (2013)

#### Dyscoletini

van Achterberg, 1984

##### Notes

This is generally considered to be a tribe of Blacinae; we list the Dyscoletini as a tribe of Brachistinae because we follow Sharanowski et al. (2011) in including the Blacinae within Brachistinae.

#### Dyscoletes

Haliday, 1840

DYSCOLUS Haliday, 1836 preocc.
DISCOLUS misspelling
MICROCENTRUS Szépligeti, 1904 preocc.
ELACHISTOCENTRUM Schulz, 1911

#### Dyscoletes lancifer

(Haliday, 1836)

Dyscolus lancifer Haliday, 1836
similis (Szépligeti, 1896, *Discoletes*)

##### Distribution

England, Scotland

### Braconinae

#### Braconinae

Nees, 1812

##### Notes

Tribal classification follows Belshaw et al. (2001) and Quicke (pers. comm.). For genera other than *Bracon* and *Coeloides*, Shaw (1994) and Shaw and Quicke (1999) give distribution data.

#### Aphrastobraconini

Ashmead, 1900

#### Atanycolus

Förster, 1862

##### Notes

species excluded from the British and Irish list by Shaw and Quicke (1999):

[*denigrator* (Linnaeus, 1758, *Ichneumon*); syn. *incertus* (Sulzer, 1776, *Ichneumon*); *heteropus* (Thomson, 1892, *Bracon*) *albiscutis* Telenga, 1936]

#### Cyanopterus

Haliday, 1835

##### Notes

species excluded from the British and Irish list by Shaw and Quicke (1999):

[*flavator* (Fabricius, 1793, *Ichneumon*); syn. *flavulator* (Ratzeburg, 1844, *Bracon*); *longipalpis* (Thomson, 1892, *Bracon*); *barcinonensis* (Marshall, 1897, *Coeloides*)]

#### Pseudovipio

Szépligeti, 1896

#### Pseudovipio guttiventris

(Thomson, 1892)

Bracon guttiventris Thomson, 1892
variegatus (Boheman, 1853, *Agathis*) preocc.
biroi Szépligeti, 1896

##### Distribution

England

##### Notes

Listed as *Glyptomorpha variegata* in Huddleston (1978).

#### Vipio

Latreille, 1804

##### Notes

species excluded from the British and Irish list by Shaw and Quicke (1999):

[*terrefactor* (Villers, 1789, *Ichneumon*); syn. *improvisus* Kokujev, 1898; *interpellator* Kokujev, 1898; *neesii* Kokujev, 1898] The only record was due to a misidentification of *Pseudovipio guttiventris* (Shaw and Quicke 1999).

#### Braconini

Nees, 1812

#### Baryproctus

Ashmead, 1900

Barycryptus Hoffmeyer, 1932

#### Baryproctus barypus

(Marshall, 1885)

Bracon barypus Marshall, 1885
hungaricus Szépligeti, 1901
caucasicus Telenga, 1936
apti Györfi, 1953

##### Distribution

England

##### Notes

See Shaw (1994) and Jennings (2008) for recent records.

#### Bracon

Fabricius, 1804

##### Notes

Comprehensive taxonomic revisions of *Bracon* are lacking for all but a few species groups. Published works differ substantially in their treatments of valid species, synonyms and subgeneric placements (compare, for example, the differing treatments of Tobias (1986), Papp (1974), Papp (1996a), and Shenefelt (1978)) so the taxonomy in Taxapad (Yu et al. 2012) has, for the most part, been used as a default classification.

#### Bracon

Fabricius, 1804

BRACO Wesmael, 1838
BRACHON Agassiz, 1846
TROPIDOBRACON Ashmead, 1900
MICROBRACON Ashmead, 1890
AMICOPLIDEA Ashmead, 1900
MACRODYCTIUM Ashmead, 1900
LIOBRACON Nason, 1905 preocc.
LORENZOA de Stefani-Perez, 1909
SELIODUS Brèthes, 1909
KULCZYNSKIA Niezabitowski, 1910
BRAZON Schulz, 1911
STRIOBRACON Fahringer, 1927
CHIVINIA Shestakov, 1932
EUTROPOBRACON Ayyar, 1928

#### Bracon (Bracon) alutaceus

Szépligeti, 1901

pygmaeus Niezabitowski, 1910; synonymy by Papp (2008a)
polonicus Fahringer, 1927; synonymy by Papp (2008a)
pallidalatus Tobias, 1957

##### Distribution

England, Scotland

##### Notes

NMS, det. Papp, added here

#### Bracon (Bracon) flavipes

Nees, 1834

##### Distribution

England

#### Bracon (Bracon) fulvipes

Nees, 1834

carinatus Szépligeti, 1901
marshalli Vayssière, 1902 preocc.
glabratus Fahringer, 1927
apionis Strand, 1928
sylvanus Greese, 1928
kiritshenkoi Telenga, 1936

##### Distribution

England, Scotland, Wales, Ireland, Isle of Man

#### Bracon (Bracon) intercessor

Nees, 1834

laetus (Wesmael, 1838, *Braco*); synonymy by Papp (2012), as a 'var' of *intercessor*
lativentris Thomson, 1892 preocc.
fulvus Szépligeti, 1896
universitatis Dalla Torre, 1898
adjectus Szépligeti, 1901
bisinuatus Szépligeti, 1901
dubiosus Szépligeti, 1901
duplicatus Szépligeti, 1901
fallaciosus Szépligeti, 1901
elegans Szépligeti, 1901
mixtus Szépligeti, 1901
mundus Szépligeti, 1901
nigropictus Szépligeti, 1901
nitidiusculus Szépligeti, 1901
rufiscapus Szépligeti, 1901
subtilis Szépligeti, 1901
suspectus Szépligeti, 1901
vigilax Kokujev, 1912
maidli Fahringer, 1925
asiaticus Fahringer, 1927 preocc.
concolor Fahringer, 1927 preocc.
major Fahringer, 1927
concorellus Strand, 1928
megasomides Strand, 1928
rhynchiti Greese, 1928
kachetinus Telenga, 1933
kansensis Fahringer, 1934 preocc.
maslovskii Telenga, 1936
segregatus Telenga, 1936

##### Distribution

England, Scotland

#### Bracon (Bracon) leptus

Marshall, 1897

centaureae Szépligeti, 1901
rufipedator Szépligeti, 1901
rufipalpis Szépligeti, 1901]

##### Distribution

England

##### Notes

Listed as British in Fauna Europaea, probably because it was described by Marshall, but the first published British record was by Jennings (2012).

#### Bracon (Bracon) longicollis

(Wesmael, 1838)

Braco longicollis Wesmael, 1838
fraudator Marshall, 1885
brevicauda Thomson, 1892 preocc.
crassicauda Thomson, 1892
pseudowesmaeli Strand, 1928
wesmaeli Fahringer, 1927 preocc.

##### Distribution

England, Scotland, Wales, Isle of Man

##### Notes

spme distribution data from Papp (1999c)

#### Bracon (Bracon) luteator

Spinola, 1808

nigripedator Nees, 1834
filicauda Costa, 1888
hypopygialis Szépligeti, 1901
intermedius Szépligeti, 1901
pilosulus Szépligeti, 1901

##### Distribution

England

##### Notes

added by Fulmek (1968) (omitted by Huddleston 1978)

#### Bracon (Bracon) nigratus

(Wesmael, 1838)

Braco nigratus Wesmael, 1838

##### Distribution

England, Ireland

#### Bracon (Bracon) pectoralis

(Wesmael, 1838)

Braco pectoralis Wesmael, 1838
ochrosus Szépligeti, 1896
sulphurator Szépligeti, 1896
unicolor Szépligeti, 1896
fumigatus Szépligeti, 1901; synonymy by Papp (2008a)

##### Distribution

England

#### Bracon (Bracon) rugulosus

Szépligeti, 1901

depressiusculus Szépligeti, 1901
neglectus Szépligeti, 1904
spurnensis Hincks, 1951

##### Distribution

England, Scotland, Wales

##### Notes

According to Taxapad (Yu et al. 2012), *rugulosus* Szépligeti is preoccupied by *Bracon rugulosus* Nees, 1811 (now classified as a species of *Aleiodes*, in Rogadinae). Omitted by Huddleston (1978).

#### Bracon (Bracon) scutellaris

(Wesmael, 1838)

Braco scutellaris Wesmael, 1838

##### Distribution

England, Scotland, Ireland

#### Bracon (Bracon) speerschneideri

Schmiedeknecht, 1897

##### Distribution

England

##### Notes

added by Papp (1999a)

#### Bracon (Bracon) subrugosus

Szépligeti, 1901

sulcatulus Szépligeti, 1896
subglaber Szépligeti, 1901
quinquemaculatus Szépligeti, 1901
trypetanus Fahringer, 1927; synonymy by Papp (2008a)
tauricus Telenga, 1936

##### Distribution

England

##### Notes

added by Papp (2008a)

#### Bracon (Bracon) trucidator

Marshall, 1888

minutator misident.
bilineatus Thomson, 1892
hilaris Marshall, 1897
pannonicus Szépligeti, 1901
marshalli Telenga, 1936 preocc.

##### Distribution

England

##### Notes

BMNH, NMS, added here; previously confused under *minutator* (van Achterberg, pers. comm.). Both species are present in the BMNH and NMS collections, det. Papp and van Achterberg.

#### Bracon (Bracon) variegator

Spinola, 1808

melanosoma Szépligeti, 1901
micros Szépligeti, 1901
nanulus Szépligeti, 1901
lineatellae (Fischer, 1968, *Habrobracon*); synonymy by Papp (2008b)

##### Distribution

England, Scotland, Ireland

#### Glabrobracon

Fahringer, 1927

##### Notes

Some taxonomic and distribution data from Papp (1999a), Papp (1999c), Papp (2000).

Species of Bracon (Glabrobracon) excluded from the British and Irish list:

[*coniferarum* Fahringer, 1928] Should not have been listed by Huddleston (1978) as it was recorded as British only on the basis of Carr’s Staffordshire lists (see Perkins 1953, Fitton et al. 1978, Shaw 2003a).

#### Bracon (Glabrobracon) abbreviator

Nees, 1834

abscissor Nees, 1834; synonymy by Papp (2008a)
oestmaeli (Wesmael, 1838, *Braco*); synonymy by Papp (2008a)
regularis (Wesmael, 1838, *Braco*); synonymy by Papp (2008a)
eutrephes Marshall, 1897
rufigaster Szépligeti, 1901; synonymy by Papp (2008a)
rufiventris Telenga, 1936 preocc.
minimula Strand, 1928
minimus Fahringer, 1927 preocc.

##### Distribution

England

#### Bracon (Glabrobracon) admotus

Papp, 2000

##### Distribution

England

##### Notes

BMNH, det. Papp, added here

#### Bracon (Glabrobracon) albion

Papp, 1999

##### Distribution

England, Scotland

##### Notes

added by Papp (1999c)

#### Bracon (Glabrobracon) arcuatus

Thomson, 1892

##### Distribution

England, Scotland

##### Notes

Added by Papp (2000); listed by Kloet and Hincks (1945) (but not by Huddleston 1978), although it is not clear on what basis.

#### Bracon (Glabrobracon) atrator

Nees, 1834

longicauda Thomson, 1892

##### Distribution

England, Scotland, Ireland

#### Bracon (Glabrobracon) caudatus

Ratzeburg, 1848

##### Distribution

England

#### Bracon (Glabrobracon) claripennis

Thomson, 1892

##### Distribution

England

##### Notes

added by Papp (2000)

#### Bracon (Glabrobracon) colpophorus

(Wesmael, 1838)

Braco colpophorus Wesmael, 1838
mokrzeckii Niezabitowski, 1927; synonymy by Papp (1997)

##### Distribution

England

#### Bracon (Glabrobracon) conjugellae

Bengtsson, 1924

bengtssoni Fahringer, 1928 preocc.
minor Fahringer, 1928 preocc.
nanana Strand, 1928

##### Distribution

England, Scotland

##### Notes

NMS, det. Papp, added here

#### Bracon (Glabrobracon) curticaudis

Szépligeti, 1901

##### Distribution

Scotland

##### Notes

Added by Papp (2008a) and removed from synonymy with *terebella*.

#### Bracon (Glabrobracon) delibator

Haliday, 1833

anthracinus Nees, 1834; synonymy by Achterberg (1997)
breviseta Fahringer, 1935

##### Distribution

England, Scotland, Ireland

#### Bracon (Glabrobracon) fuscicoxis

(Wesmael, 1838)

Braco fuscicoxis Wesmael, 1838
levicarinatus Niezabitowski, 1910

##### Distribution

England

#### Bracon (Glabrobracon) glaphyrus

Marshall, 1897

##### Distribution

England

##### Notes

Faure (1924) mentions English material but *glaphyrus* was omitted by Huddleston (1978).

#### Bracon (Glabrobracon) guttator

Panzer, 1804

##### Distribution

England

##### Notes

Omitted by Huddleston (1978); recorded as British by Fulmek (1968), although we are not sure how reliably; English material in NMS.

#### Bracon (Glabrobracon) immutator

Nees, 1834

efoveolatus Thomson, 1892
hemirugosus Szépligeti, 1901
flavicoxanus Strand, 1928
marshalli Fahringer, 1927 preocc.
romani Fahringer, 1927 preocc.
uplandiae Strand, 1928
austriacus Fahringer, 1936
nigripalpis Telenga, 1936

##### Distribution

England, Scotland

#### Bracon (Glabrobracon) instabilis

Marshall, 1897

##### Distribution

England

#### Bracon (Glabrobracon) kopelkei

Papp, 2000

##### Distribution

England

##### Notes

BMNH, det. Papp, added here

#### Bracon (Glabrobracon) longulus

Thomson, 1892

##### Distribution

England, Scotland

##### Notes

NMS, det. Papp, added here

#### Bracon (Glabrobracon) marshalli

Szépligeti, 1901

obscurator misident.

##### Distribution

England, Scotland

##### Notes

Added by Papp (2000); listed by Kloet and Hincks (1945) (but not by Huddleston 1978), although it is not clear on what basis.

#### Bracon (Glabrobracon) minutator

(Fabricius, 1798)

Ichneumon minutator Fabricius, 1798
thalassinus Schmiedeknecht, 1897; synonymy by Papp (1999a)
tener Szépligeti, 1904
abscissoris Strand, 1928
flavipalpula Strand, 1928
flavipalpis Fahringer, 1928 preocc.
rufiventris Fahringer, 1928 preocc.
notatus Telenga, 1936
unicolor Telenga, 1936 preocc.

##### Distribution

England, Ireland

##### Notes

Braconminutator auct. is trucidator Marshall (van Achterberg, pers. comm.).

#### Bracon (Glabrobracon) momphae

Papp, 1999

##### Distribution

England, Scotland

##### Notes

added by Papp (1999c)

#### Bracon (Glabrobracon) nigricollis

(Wesmael, 1838)

Braco nigricollis Wesmael, 1838
brunneomaculatus Schütze & Roman, 1931

##### Distribution

England

##### Notes

NMS, det. Papp, added here

#### Bracon (Glabrobracon) obscurator

Nees, 1811

kotulai Niezabitowski, 1910
nigripes Fahringer, 1928 preocc.
rytrensis Fahringer, 1928 preocc.
rytronis Strand, 1928
zaleszczykiensis Strand, 1928

##### Distribution

England, Scotland, Wales

#### Bracon (Glabrobracon) otiosus

Marshall, 1885

macrurus Thomson, 1892; synonymy by Papp (1999c)
explorator Szépligeti, 1904 preocc.; synonymy by Papp (1999c)
pumilionis Roman, 1928; synonymy by Papp (1999c)

##### Distribution

England

##### Notes

Treated as a synonym of *bipartitus* (=*variator*) by Belokobylskij et al. (2003).

#### Bracon (Glabrobracon) pallicarpus

Thomson, 1892

##### Distribution

England

##### Notes

added by Papp (2000)

#### Bracon (Glabrobracon) parvicornis

Thomson, 1892

carbonarius Szépligeti, 1901 preocc.
aterrimus Telenga, 1936 preocc.

##### Distribution

England

##### Notes

NMS, det. Papp, added here

#### Bracon (Glabrobracon) parvulus

Wesmael, 1838

fumipennis Thomson, 1892
fuscipennis Thomson, 1892 preocc.
thomsoni Marshall, 1897

##### Distribution

England, Scotland, Wales

##### Notes

NMS, det. Papp, added here

#### Bracon (Glabrobracon) pineti

Thomson, 1892

##### Distribution

England

##### Notes

NMS, det. Papp, added here

#### Bracon (Glabrobracon) praecox

(Wesmael, 1838)

Braco praecox Wesmael, 1838
biorrhizae Fahringer, 1928
elongatus Dutu-Lacatusu, 1956 preocc.

##### Distribution

Scotland

##### Notes

NMS, det. Papp, added here

#### Bracon (Glabrobracon) ratzeburgii

Dalla Torre, 1898

longicaudis Ratzeburg, 1852 preocc.

##### Distribution

England

#### Bracon (Glabrobracon) terebella

(Wesmael, 1838)

Braco terebella Wesmael, 1838
breviterebris Fahringer, 1928
miaricola Strand, 1928
wesmaeli Fahringer, 1928 preocc.
unicolor Telenga, 1936 preocc.

##### Distribution

England

#### Bracon (Glabrobracon) variator

Nees, 1811

bipartitus (Wesmael, 1838, *Braco*)
maculiger (Wesmael, 1838, *Braco*)
collinus Szépligeti, 1901
breviventris Szépligeti, 1901
rytrensis Niezabitowski, 1910
sueciensis Fahringer, 1927
dimidiatus Fahringer, 1928 preocc.
nigerrimus Fahringer, 1928
rytrensis Fahringer, 1928 preocc.
rytrocola Strand, 1928
chinensis Fahringer, 1929 preocc.
caucasicus Telenga, 1936
collaris Telenga, 1936 preocc.
meridionalis Telenga, 1936
ornatulus Telenga, 1936
rytrensis Telenga, 1936 preocc.
turcmenus Telenga, 1936
asiaticus Telenga, 1949 preocc.

##### Distribution

England, Scotland, Wales, Ireland

##### Notes

*Braco bipartitus* is listed as a subspecies of *variator* in Taxapad (Yu et al. 2012). Listed as a British species by Huddleston (1978) but this actually refers to *otiosus*.

#### Bracon (Glabrobracon) xanthogaster

Nees, 1834

breviseta Hedwig, 1961 preocc.

##### Distribution

England

##### Notes

NMS, det. Papp, added here

#### Habrobracon

Ashmead, 1895

#### Bracon (Habrobracon) concolorans

Marshall, 1900

concolor Thomson, 1892 preocc.
nigricans (Szépligeti, 1901, *Habrobracon*); synonymy by Papp (2008b)
mongolicus (Telenga, 1936, *Habrobracon*)

##### Distribution

England, Scotland, Ireland

##### Notes

Added by Papp (2008b) and removed from synonymy with *stabilis*.

#### Bracon (Habrobracon) crassicornis

Thomson, 1894

flavosignatus (Tobias, 1957, *Habrobracon*); synonymy by Papp (2008b)

##### Distribution

England, Scotland

##### Notes

added by Papp (2008b)

#### Bracon (Habrobracon) hebetor

Say, 1836

dorsator Say, 1836
brevicornis (Wesmael, 1838, *Braco*)
juglandis Ashmead, 1889
brunneus (Szépligeti, 1901, *Habrobracon*)
vernalis (Szépligeti, 1901, *Habrobracon*)
beneficientior (Viereck, 1911, *Habrobracon*)
plotnicovi (Bogoljubov, 1914, *Habrobracon*)
breviantennatus de Stefani, 1919
serinopae (Cherian, 1929, *Microbracon*)
tortricidarum (Goidanich, 1934, *Habrobracon*)
pectinophorae (Watanabe, 1935, *Habrobracon*)
asiaticus (Telenga, 1936, *Habrobracon*)
flavus (Telenga, 1936, *Habrobracon*)
turkestanicus (Telenga, 1936, *Habrobracon*)
lozinskii (Bogacev, 1939, *Habrobracon*)

##### Distribution

England

##### Notes

Mainly an indoors species in Britain, attacking pests of stored products; *brevicornis* been variously treated as a valid species or as a synonym of *hebetor*, being listed under the latter in Taxapad (Yu et al. 2012) and by Papp (2008b). Both *brevicornis* and *hebetor* have been recorded as British.

#### Bracon (Habrobracon) stabilis

(Wesmael, 1838)

Braco stabilis Wesmael, 1838
opacus Stelfox, 1953 preocc.

##### Distribution

England, Ireland

#### Lucobracon

Fahringer, 1927

##### Notes

species of Bracon (Lucobracon) excluded from the British and Irish list:

[*strobilorum* Ratzeburg, 1848] England and Ireland are listed under the distribution in Shenefelt (1978), and Britain in Fauna Europaea, but we have been unable to trace any British or Irish literature records or specimens. In Belokobylskij et al. (2003) and in Taxapad (Yu et al. 2012) this is listed as a species of *Coeloides* but Papp (1992) had established that this species had been mis-interpreted and is in fact a species of Bracon (Lucobracon). Swiss specimens identified as *strobilorum* by Papp in NMS and BMNH belong in *Bracon*.

#### Bracon (Lucobracon) brachycerus

Thomson, 1892

kudsiricus Papp, 1965

##### Distribution

England

##### Notes

NMS, det. Papp, added here

#### Bracon (Lucobracon) crassungula

Thomson, 1892

##### Distribution

England

##### Notes

NMS, BMNH, det. Papp, added here

#### Bracon (Lucobracon) erraticus

(Wesmael, 1838)

Braco erraticus Wesmael, 1838
superciliosus (Wesmael, 1838, *Braco*)
erythrostictus Marshall, 1885
exarator Marshall, 1885
praetermissus Marshall, 1885
vectensis Marshall, 1885; synonymy by Papp (1999c)
foveola Thomson, 1892
aestivalis Szépligeti, 1901; synonymy by Papp (2005b)
confinis Szépligeti, 1901
congruus Szépligeti, 1901 preocc.
similis Szépligeti, 1901
ventricosus Szépligeti, 1901
secernendus Schulz, 1906
lagodechianus Telenga, 1936
maculatus Telenga, 1936
planiceps Telenga, 1936
talitzkii Telenga, 1936
transcaspicus Telenga, 1936
hades Papp, 1965

##### Distribution

England, Scotland, Ireland, Isle of Man

##### Notes

Although *pratermissus* was synonymised with *Bracon erraticus* by Papp (1999c), this has not been followed by Belokobylskij et al. (2003).

#### Bracon (Lucobracon) flagellaris

Thomson, 1892

facialis Thomson, 1892 preocc.
thomsonii Dalla Torre, 1898 preocc.
dallatorrei Szépligeti, 1901

##### Distribution

England

##### Notes

BMNH, det. Papp, added here

#### Bracon (Lucobracon) grandiceps

Thomson, 1892

gallicus Thomson, 1892

##### Distribution

Scotland

##### Notes

NMS, BMNH, det. Papp, added here

#### Bracon (Lucobracon) guttiger

(Wesmael, 1838)

Braco guttiger Wesmael, 1838
fasciatus Fahringer, 1927

##### Distribution

England, Scotland

#### Bracon (Lucobracon) hylobii

Ratzeburg, 1848

bruchorum Fahringer, 1934

##### Distribution

England, Scotland

#### Bracon (Lucobracon) larvicida

(Wesmael, 1838)

Braco larvicida Wesmael, 1838
crassiusculus Szépligeti, 1901
romani Fahringer, 1927 preocc.
szepligetii Fahringer, 1927 preocc.
fahringeriensis Strand, 1928
pseudoromani Strand, 1928

##### Distribution

England

#### Bracon (Lucobracon) nigriventris

Wesmael, 1838

Braco nigriventris Wesmael, 1838
subornatus Szépligeti, 1901; synonymy by Papp (2008a)
biroi Fahringer, 1927
minor Fahringer, 1927
albanicus Telenga, 1936
laticeps Telenga, 1936
lencoranus Telenga, 1936
persimilis Telenga, 1936
turolus Papp, 1984

##### Distribution

England

##### Notes

NMS, det. Papp, added here

#### Bracon (Lucobracon) sphaerocephalus

Szépligeti, 1901

globiceps Szépligeti, 1901; synonymy by Papp (2005b)

##### Distribution

England, Scotland

##### Notes

NMS, det. Papp, added here

#### Bracon (Lucobracon) thuringiacus

Schmiedeknecht, 1897

schmiedeknechti Fahringer, 1927 preocc.
blankenburgiae Strand, 1928

##### Distribution

England

##### Notes

added by Papp (1999a)

#### Bracon (Lucobracon) tornator

Marshall, 1885

aequalis Thomson, 1892

##### Distribution

England, Scotland, Wales

##### Notes

some distribution data from Papp (1997)

#### Bracon (Lucobracon) triangularis

Nees, 1834

##### Distribution

England, Ireland

#### Orthobracon

Fahringer, 1927

##### Notes

species of Bracon (Orthobracon) excluded from the British and Irish list:

[*tenuicornis* (Wesmael, 1838, *Braco*)] Probably not a valid British or Irish species now that B. (Lucobracon) erythrostictus has been taken out of synonymy (Papp 1999c).

#### Bracon (Orthobracon) discoideus

(Wesmael, 1838)

Braco discoideus Wesmael, 1838
opionus Fahringer, 1928
sculpturatus Fahringer, 1928
sculpturifera Strand, 1928

##### Distribution

England, Scotland, Ireland

#### Bracon (Orthobracon) epitriptus

Marshall, 1885

pallidipes Szépligeti, 1896; synonymy by Papp (2008a)
melanogaster Szépligeti, 1901

##### Distribution

England, Scotland, Wales, Ireland

##### Notes

some distribution data from Papp (1999c)

#### Bracon (Orthobracon) exhilarator

Nees, 1834

satanas (Wesmael, 1838, *Braco*)
tibialis Zetterstedt, 1838
striolatus Thomson, 1892
marshalli Fahringer, 1927 preocc,
polonicus Fahringer, 1927 preocc.
polonicella Strand, 1928
varicoloris Strand, 1928

##### Distribution

England, Scotland, Wales, Isle of Man

#### Bracon (Orthobracon) filicornis

Thomson, 1892

##### Distribution

Scotland

##### Notes

NMS, det. Papp, added here

#### Bracon (Orthobracon) laevigatissimus

Dalla Torre, 1898

laevigatus Ratzeburg, 1852 preocc.

##### Distribution

England

##### Notes

Omitted by Huddleston (1978); listed as British by various authors dating back to Marshall (1885).

#### Bracon (Orthobracon) mediator

Nees, 1834

##### Distribution

England, Scotland, Ireland

#### Bracon (Orthobracon) ochropus

Nees, 1834

marshalli Fahringer, 1927 preocc.
neesi Fahringer, 1927
ochropodis Strand, 1928

##### Distribution

England, Scotland

##### Notes

NMS, det. Papp, added here

#### Bracon (Orthobracon) orbus

Papp, 1981

##### Distribution

England

##### Notes

NMS, det. Papp, added here

#### Bracon (Orthobracon) picticornis

Wesmael, 1838

gallarum Ratzeburg, 1852
versicolor Szépligeti, 1901
ratzeburgensis Strand, 1928
laevigatus Fahringer, 1927 preocc.

##### Distribution

England, Ireland

#### Bracon (Orthobracon) procerus

Papp, 1965

##### Distribution

England

##### Notes

NMS, det. Papp, added here

#### Bracon (Orthobracon) pulcher

Bengtsson, 1924

bengtssoni Fahringer, 1927

##### Distribution

England

##### Notes

NMS, det. Papp, added here

#### Bracon (Orthobracon) roberti

(Wesmael, 1838)

Braco roberti Wesmael, 1838

##### Distribution

England, Ireland

#### Bracon (Orthobracon) romani

Fahringer, 1927

##### Distribution

England, Scotland, Wales, Ireland

##### Notes

NMS, det. Papp, added here

#### Bracon (Orthobracon) subcylindricus

(Wesmael, 1838)

Braco subcylindricus Wesmael, 1838
niger (Vojnovskaja-Krieger, 1929, *Baryproctus*)

##### Distribution

England

#### Bracon (Orthobracon) subsinuatus

Szépligeti, 1901

##### Distribution

England, Scotland, Ireland

##### Notes

Added by Papp (2008a). Removed from synonymy with *epitriptus* by Papp (2008a), who treats both species as belonging to the subgenus *Glabrobracon*, contrary to their placement in Taxapad (Yu et al. 2012).

#### Bracon (Orthobracon) titubans

(Wesmael, 1838)

Braco titubans Wesmael, 1838
tarsator Thomson, 1892
terebrator Szépligeti, 1901

##### Distribution

England

##### Notes

NMS, BMNH, det. Papp, added here

#### Bracon (Orthobracon) virgatus

Marshall, 1897

lineifer van Achterberg, 1988; synonymy by Papp (1999c)

##### Distribution

England, Scotland

##### Notes

some distribution data from Shaw and Bailey (1991)

#### Osculobracon

Papp, 2008

#### Bracon (Osculobracon) osculator

Nees, 1811

bisignatus (Wesmael, 1838, *Braco*)
degenerator Marshall, 1885
minutus Szépligeti, 1901
temporalis Telenga, 1936
venustus Telenga, 1936

##### Distribution

England, Scotland, Wales, Ireland

#### Pigeria

van Achterberg, 1985

##### Notes

Described as a separate genus but Quicke and Sharkey (1989) suggested that Pigeria could be treated as a subgenus of Bracon, which was followed by Papp (1998).

#### Bracon (Pigeria) piger

(Wesmael, 1838)

Braco piger Wesmael, 1838
rotundatus Szépligeti, 1901; synonymy by Papp (2008a)
rotundulus Szépligeti, 1904

##### Distribution

England, Ireland

##### Notes

*Bracon semiluteus* Walker, 1874 is not a synonym of *piger* (contra Morley (1913) and subsequent authors) but is a junior synonym of another species of *Bracon* (Papp, in prep.).

#### Bracon (Pigeria) wolschrijni

(van Achterberg, 1985)

Pigeria wolschrijni van Achterberg, 1985

##### Distribution

Wales

##### Notes

NMS, det. van Achterberg, added here

#### Rostrobracon

Tobias, 1957

##### Notes

species of Bracon (Rostrobracon) excluded from the British and Irish list:

[*urinator* (Fabricius, 1798, *Ichneumon*); syn. *cuspidator* (Rossi, 1792, *Ichneumon*); *comptus* Marshall, 1897] Marshall (1900) mentions an English specimen but this seems unlikely in view of its present southern European range.

#### Coeloidini

Tobias, 1957

#### Coeloides

Wesmael, 1838

SYNTOMOMELUS Kokujev, 1902
HABROBRACONIDEA Viereck, 1912
COELOIDINA Viereck, 1921
CEROBRACON Viereck, 1926

##### Notes

Distribution data from Shaw (2000b).

#### Coeloides abdominalis

(Zetterstedt, 1838)

Bracon abdominalis Zetterstedt, 1838

##### Distribution

England

#### Coeloides filiformis

Ratzeburg, 1852

melanurus Ivanov, 1896

##### Distribution

England

##### Notes

added by Shaw (2000b)

#### Coeloides melanostigma

Strand, 1918

sordidator misident.
stigmaticus Hellén, 1928

##### Distribution

England

##### Notes

Added by Shaw (2000b); according to Haeselbarth (in Belokobylskij et al. 2003), the name *sordidator* (Ratzeburg, 1844, *Bracon*) probably does not belong in *Coeloides*, so the species usually referred to as *sordidator* takes the name *melanostigma*.

#### Coeloides melanotus

Wesmael, 1838

flavus Ivanov, 1896
maculatus Ivanov, 1896

##### Distribution

England, Wales

#### Coeloides scolyticida

Wesmael, 1838

initiatellus (Ratzeburg, 1848, *Bracon*)

##### Distribution

England

### Cardiochilinae

#### Cardiochilinae

Ashmead, 1900

##### Notes

no British or Irish species

#### Cardiochiles

Nees, 1819

Ditherus Cameron, 1902

##### Notes

species of *Cardiochiles* excluded from the British and Irish list:

[*saltator* (Fabricius, 1781, *Ichneumon*); syn. *brachialis* Rondani, 1877; *katkowi* Kokujev, 1895; *fumipennis* Szépligeti, 1901; *sibiricus* Telenga, 1955] not British, recorded in error (Shaw and Huddleston 1991)

### Cenocoeliinae

#### Cenocoeliinae

Szépligeti, 1901

##### Notes

Distribution data from Shaw (1999).

#### Cenocoelius

Haliday, 1840

LACCOPHRYS Förster, 1863
PROMACHUS Cresson, 1887 preocc.
CAENOCOELIUS Marshall, 1894
POSTPROMACHUS Maes, 1999

#### Cenocoelius aartseni

(van Achterberg, 1994)

Promachus aartseni van Achterberg, 1994

##### Distribution

England

##### Notes

added by Shaw (1999)

#### Cenocoelius analis

(Nees, 1834)

Bracon analis Nees, 1834
flavifrons Haliday, 1840
cephalotes (Ratzeburg, 1848, *Opius*) preocc.
magdalini (Förster, 1863, *Laccophrys*)
hungaricus Kiss, 1927

##### Distribution

England, Ireland

#### Lestricus

Reinhard, 1865

#### Lestricus secalis

(Linnaeus, 1758)

Ichneumon secalis Linnaeus, 1758
agricolator (Linnaeus, 1767, *Ichneumon*)
rubriceps (Ratzeburg, 1844, *Alysia*)
femorator (Tobias, 1973, *Cenocoelius*)

##### Distribution

Scotland

##### Notes

NMS, det. Shaw, added here; removed from the British list by Shaw (1999) but recently found in Scotland.

### Charmontinae

#### Charmontinae

van Achterberg, 1979

#### Charmon

Haliday, 1833

PROVANCHERIA Ashmead, 1900
CYCLOCORMUS Cameron, 1911
EUBADIZON misident.

#### Charmon cruentatus

Haliday, 1833

pectoralis (Nees, 1834, *Eubadizon*)
pleuralis (Cresson, 1872, *Eubadizon*)
luteus (Cameron, 1911, *Cyclocormus*)
Charmon cruentatus ?*brevicauda* (Hellén, 1958, *Eubadizon*)

##### Distribution

England, Scotland, Wales, Ireland, Isle of Man

##### Notes

Although Achterberg (1979) treated *brevicauda* as a ‘form’ of *cruentatus*, the possibility of a separate species requires investigation.

#### Charmon extensor

(Linnaeus, 1758)

Ichneumon extensor Linnaeus, 1758
gracilis (Provancher, 1880, *Eubadizon*)
hungaricus (Kiss, 1927, *Calyptus*)
striatus (Shestakov, 1940, *Eubadizon*)

##### Distribution

England, Scotland, Ireland

### Cheloninae

#### Cheloninae

Förster, 1863

ADELIINAE Viereck, 1918
ACAELIINAE Viereck, 1918
ACOELIINAE Viereck, 1918

##### Notes

The genus *Adelius* and related extralimital genera have usually been treated as comprising a separate subfamily, Adeliinae. Recent phylogenetic studies (Belshaw et al. 2000, Belshaw and Quicke 2002) have placed the adeliines within the Cheloninae, as sister taxon to *Phanerotoma*.

#### Chelonini

Förster, 1863

#### Ascogaster

Wesmael, 1835

CASCOGASTER Baker, 1926
LEPTODREPANA Shaw, 1983; synonymy by Achterberg (1990)

##### Notes

Distribution and synonymic data taken from Huddleston (1984).

Species of *Ascogster* excluded from the British and Irish list:

[*bicarinata* (Herrich-Schäffer, 1838, *Chelonus*); syn. *mlokossewitschi* Kokujev, 1895; syn. *rufiventris* Telenga, 1941; preocc.] Included by Huddleston (1978) but no British specimens were seen by Huddleston (1984).

[*similis* (Nees, 1816, *Chelonus*)] The type is lost and the species is unplaceable Huddleston (1984).

#### Ascogaster abdominator

(Dahlbom, 1833)

Chelonus abdominator Dahlbom, 1833
instabilis Wesmael, 1835
fulviventris Curtis, 1837
femoralis (Herrich-Schäffer, 1838, *Chelonus*)
rufiventris (Herrich-Schäffer, 1838, *Chelonus*)
pallida Ruthe, 1855

##### Distribution

England, Scotland, Ireland

#### Ascogaster albitarsus

Reinhard, 1867

similis (Herrich-Schäffer, 1838, *Chelonus*)
leptopus Thomson, 1874

##### Distribution

Scotland, Ireland

##### Notes

added by Huddleston (1984)

#### Ascogaster annularis

(Nees, 1816)

Sigalphus annularis Nees, 1816

##### Distribution

England

#### Ascogaster armata

Wesmael, 1835

pulchella (Curtis, 1829, *Chelonus*) nom. nud.
esenbeckii Curtis, 1837
luteicornis (Herrich-Schäffer, 1838, *Chelonus*)

##### Distribution

England, Wales

#### Ascogaster bidentula

Wesmael, 1835

scabriuscula (Zetterstedt, 1838, *Sigalphus*)
multiarticulata (Ratzeburg, 1852, *Chelonus*)
gibbiscuta Thomson, 1874
fuscipennis Thomson, 1892
atamiensis Ashmead, 1906

##### Distribution

England, Scotland, Wales, Ireland

##### Notes

added by Huddleston (1984)

#### Ascogaster brevicornis

Wesmael, 1835

monilicornis (Herrich-Schäffer, 1838, *Chelonus*)

##### Distribution

England, Ireland

##### Notes

added by Huddleston (1984)

#### Ascogaster canifrons

Wesmael, 1835

graniger Thomson, 1892
zernyana Fahringer, 1925

##### Distribution

England, Ireland

#### Ascogaster consobrina

Curtis, 1837

##### Distribution

England, Scotland, Ireland

#### Ascogaster dentifer

Tobias, 1976

##### Distribution

England

##### Notes

added by Huddleston (1984)

#### Ascogaster dispar

Fahringer, 1934

spinifer Tobias, 1964
kozlovi Tobias, 1972

##### Distribution

England

##### Notes

added by Huddleston (1984)

#### Ascogaster gonocephala

Wesmael, 1835

##### Distribution

England

##### Notes

added by Huddleston (1984)

#### Ascogaster grahami

Huddleston, 1984

##### Distribution

England

##### Notes

added by Huddleston (1984)

#### Ascogaster klugii

(Nees, 1816)

Sigalphus klugii Nees, 1816
ruficeps Wesmael, 1835
neesii Reinhard, 1867

##### Distribution

England

##### Notes

added by Huddleston (1984)

#### Ascogaster quadridentata

Wesmael, 1835

pallidicornis Curtis, 1837
impressa (Herrich-Schäffer, 1838, *Chelonus*)
quadridens (Herrich-Schäffer, 1838, *Chelonus*)
cynipum Thomson, 1892
nigricornis Thomson, 1892
egregia Kokujev, 1895
nigrator (Szépligeti, 1896, *Chelonus*)
carpocapsae (Viereck, 1909, *Chelonus*)
epinotiae Watanabe, 1937

##### Distribution

England

#### Ascogaster rufidens

Wesmael, 1835

rufipes (Herrich-Schäffer, 1838, *Chelonus*) preocc.
laevigator (Ratzeburg, 1852, *Chelonus*)

##### Distribution

England, Ireland

#### Ascogaster rufipes

(Latreille, 1809)

Sigalphus rufipes Latreille, 1809
elegans (Nees, 1816, *Sigalphus*)
fasciata (Dahlbom, 1833, *Chelonus*)
pallipes (Herrich-Schäffer, 1838, *Chelonus*)
rubripes (Lucas, 1849, *Chelonus*)
rugosula (Goureau, 1861, *Chelonus*)
ratzeburgii Marshall, 1885
arisanica Sonan, 1932
nigribasis Fahringer, 1934
soror Telenga, 1941

##### Distribution

England, Ireland

#### Ascogaster varipes

Wesmael, 1835

atriceps (Ratzeburg, 1844, *Chelonus*)
tersa Reinhard, 1867
cavifrons Thomson, 1874
sternalis Thomson, 1874
catula (Marshall, 1885, *Chelonus*); synonymy by Papp (1996c)
jaroslawensis Kokujev, 1895

##### Distribution

England, Scotland, Ireland

#### Chelonus

Jurine, 1801

#### Chelonus

Jurine, 1801

Anomala von Block, 1799 nom. ob.
DAVISANIA La Munyon, 1877
ARICHELONUS Viereck, 1913
MEGACHELONUS Baker, 1926

#### Chelonus (Chelonus) acuminatus

Herrich-Schäffer, 1838

##### Distribution

England

##### Notes

NMS, det. Huddleston, added here

#### Chelonus (Chelonus) annulatus

(Nees, 1816)

Sigalphus annulatus Nees, 1816
maculatus Szépligeti, 1896

##### Distribution

England, Scotland, Ireland

#### Chelonus (Chelonus) asiaticus

Telenga, 1941

##### Distribution

England, Ireland

##### Notes

BMNH, det. Lozan, added here. The name is a junior homonym of *Chelonus asiaticus* Fahringer, 1932.

#### Chelonus (Chelonus) canescens

Wesmael, 1835

##### Distribution

England

#### Chelonus (Chelonus) carbonator

Marshall, 1885

asiaticus Fahringer, 1932

##### Distribution

England

#### Chelonus (Chelonus) corvulus

Marshall, 1885

suturatus Szépligeti, 1898

##### Distribution

England, Scotland

#### Chelonus (Chelonus) cylindrus

(Klug, 1816)

Sigalphus cylindrus Klug, 1816
variabilis Herrich-Schäffer, 1838
macrocerus Thomson, 1874
speculator Marshall, 1885
ebeninus Fahringer, 1934

##### Distribution

England

##### Notes

*Chelonus speculator* was listed as a synonym of *oculator* by Huddleston (1978).

#### Chelonus (Chelonus) decorus

Marshall, 1885

clavipes Curtis, 1837 nom. nud.
clavipes Fahringer, 1934
szepligetii Fahringer, 1934

##### Distribution

England

#### Chelonus (Chelonus) inanitus

(Linnaeus, 1767)

Cynips inanita Linnaeus, 1767
binarius (Fourcroy, 1785, *Ichneumon*)
atomos (Rossi, 1790, *Ichneumon*)

##### Distribution

England, Ireland

#### Chelonus (Chelonus) obscuratus

Herrich-Schäffer, 1838

intermedius Thomson, 1874

##### Distribution

England

##### Notes

NMS, det. Huddleston, added here

#### Chelonus (Chelonus) oculator

(Fabricius, 1785)

Ichneumon oculator Fabricius, 1785
integer (von Block, 1799, *Anomala*)
mutabilis (Nees, 1816, *Sigalphus*)
oculatus (Nees, 1816, *Sigalphus*)

##### Distribution

England, Scotland

#### Chelonus (Chelonus) pusio

Marshall, 1885

##### Distribution

England

#### Chelonus (Chelonus) scabrator

(Fabricius, 1793)

Ichneumon scabrator Fabricius, 1793
scaber (Nees, 1816, *Sigalphus*)
buccatus Thomson, 1874

##### Distribution

England

#### Chelonus (Chelonus) submuticus

Wesmael, 1835

luteipes Thomson, 1874; synonymy by Papp (1995b)

##### Distribution

England

#### Chelonus (Chelonus) wesmaelii

Curtis, 1837

zimini Tobias, 1972

##### Distribution

England

#### Microchelonus

Szépligeti, 1908

CHELONELLA Szépligeti, 1908
NEOCHELONELLA Hincks, 1943

##### Notes

Treated as a valid genus by Papp (1996c) and Belokobylskij et al. (2003), sometimes treated as a straight synonym of *Chelonus* (Achterberg and Polaszek 1996), here treated as a subgenus of *Chelonus*, following Taxapad (Yu et al. 2012).

#### Chelonus (Microchelonus) atripes

Thomson, 1874

cunctator (Papp, 1971, *Microchelonus*)
kamtshaticus (Tobias, 1986, *Microchelonus*)

##### Distribution

England

##### Notes

NMS, BMNH, det. Huddleston and Lozan, added here

#### Chelonus (Microchelonus) basalis

Curtis, 1837

##### Distribution

England, Scotland

#### Chelonus (Microchelonus) binus

(Tobias, 1995)

Microchelonus binus Tobias, 1995

##### Distribution

England

##### Notes

BMNH, det. Lozan, added here

#### Chelonus (Microchelonus) contractus

(Nees, 1816)

Sigalphus contractus Nees, 1816
compressiscapus Szépligeti, 1898; synonymy by Papp (1996c)

##### Distribution

England, Scotland, Wales, Ireland

##### Notes

some distribution data from Tobias and Shaw (2005)

#### Chelonus (Microchelonus) depressus

Thomson, 1874

##### Distribution

England, Scotland, Wales

##### Notes

NMS, BMNH, det. Huddleston and Lozan, added here

#### Chelonus (Microchelonus) exilis

Marshall, 1885

excavatus Tobias, 1972; synonymy by Papp (1995b)

##### Distribution

England

#### Chelonus (Microchelonus) fenestratus

(Nees, 1816)

Sigalphus fenestratus Nees, 1816
dispar Marshall, 1885
bimaculatus Ivanov, 1899 preocc.

##### Distribution

England

##### Notes

Synonymised under *contractus* by Papp (1995b) but treated as a separate species by Tobias and Lozan (2003) and listed as such in Taxapad (Yu et al. 2012).

#### Chelonus (Microchelonus) fumipennis

(Tobias, 1986)

Microchelonus fumipennis Tobias, 1986

##### Notes

BMNH, det. Lozan, added here; specimen lacking locality data, Billups coll.

#### Chelonus (Microchelonus) latrunculus

Marshall, 1885

Chelonus (Microchelonus) latrunculus ?*parcicornis* Herrich-Schäffer, 1838
thomsonii Dalla Torre, 1898
polonicus Fahringer, 1934
rectus (Papp, 1971, *Microchelonus*)

##### Distribution

England, Scotland

##### Notes

Although *parcicornis* would have priority, Papp (1995b) states that the type is lost and its identity unverifiable.

#### Chelonus (Microchelonus) lugubris

Wesmael, 1835

##### Distribution

England, Ireland

##### Notes

NMS, BMNH, det. Huddleston and Lozan, added here

#### Chelonus (Microchelonus) microphtalmus

Wesmael, 1838

dilatus Papp, 1971

##### Distribution

England

##### Notes

NMS, det. Huddleston, added here

#### Chelonus (Microchelonus) miscellae

(Tobias & Shaw, 2005)

Microchelonus miscellae Tobias & Shaw, 2005

##### Distribution

England

##### Notes

added by Tobias and Shaw (2005)

#### Chelonus (Microchelonus) retusus

(Nees, 1816)

Sigalphus retusus Nees, 1816
emarginatus Herrich-Schäffer, 1838
subemarginatus Herrich-Schäffer, 1838
caudatus Thomson, 1874
pamiricus Vojnovskaja-Krieger, 1931

##### Distribution

England

##### Notes

BMNH, det. Lozan, added here

#### Chelonus (Microchelonus) risorius

Reinhard, 1867

fissus Szépligeti, 1900 preocc.
fissuralis (Tobias, 1964, *Neochelonella*)
magnifissus (Tobias, 1986, *Microchelonus*)

##### Distribution

England

#### Chelonus (Microchelonus) sulcatus

Jurine, 1807

##### Distribution

England

#### Parachelonus

Tobias, 1995

#### Chelonus (Parachelonus) gravenhorstii

(Nees, 1816)

Sigalphus gravenhorstii Nees, 1816
maculator Dahlbom, 1833
eurytheca Wesmael, 1838
adjaricus Tobias, 1976
tricolor Tobias, 1976

##### Distribution

England

#### Chelonus (Parachelonus) pellucens

(Nees, 1816)

Sigalphus pellucens Nees, 1816
nitens Reinhard, 1867
alboannulatus Szépligeti, 1896
pulchricornis Szépligeti, 1898
varimaculatus Tobias, 1986
austriacus Fahringer, 1934

##### Distribution

England

##### Notes

added by Papp (2004c)

#### Stylochelonus

Hellén, 1958

#### Chelonus (Stylochelonus) pedator

Dahlbom, 1833

secutor (Marshall, 1885, *Chelonus*)

##### Distribution

England, Scotland

#### Chelonus (Stylochelonus) pusillus

(Szépligeti, 1908)

Stylochelonus pusillus Szépligeti, 1908
furtivus (Tobias, 1986, *Microchelonus*)
tuberculiventris (Tobias, 1986, *Microchelonus*)

##### Distribution

England

##### Notes

BMNH, det. Lozan, added here

#### Phanerotomini

Baker, 1926

#### Adelius

Haliday, 1833

ACAELIUS Haliday, 1834
ACOELIUS Haliday, 1835
PLEIOMERUS Wesmael, 1837
ANOMOPTERUS Rohwer, 1914
MYRIOLA Shestakov, 1932

#### Adelius erythronotus

(Förster, 1851)

Acoelius erythronotus Förster, 1851
pyrrhia (Beirne, 1945, *Acoelius*)
flavus (Tobias, 1966, *Acoelius*)

##### Distribution

England, Ireland

##### Notes

*Adelius pyrrhia* was listed as a valid species by O'Connor et al. (1999) but as a synonym of *erythronotus* by Belokobylskij et al. (2003).

#### Adelius germanus

(Haliday, 1834)

Acaelius germanus Haliday, 1834

##### Distribution

England, Ireland

#### Adelius subfasciatus

Haliday, 1833

minutissimus (Zetterstedt, 1840, *Bracon*)
parvulus (Förster, 1851, *Acoelius*)

##### Distribution

England, Wales, Ireland

##### Notes

some distribution data from Shaw and Askew (1976)

#### Adelius viator

(Förster, 1851)

Acoelius viator Förster, 1851

##### Distribution

England

#### Phanerotoma

Wesmael, 1838

#### Phanerotoma

Wesmael, 1838

PHANEROGASTER Wesmael, 1838 unavailable
SULYDUS Du Buysson, 1897
ICHNEUTIPTERUS Vachal, 1907; synonymy by Achterberg (1990)
NEOPHANEROTOMA Szépligeti, 1908
NEOACAMPSIS Szépligeti, 1914; synonymy by Achterberg (1990)
PHANEROTOMINA Shestakov, 1930

##### Notes

species of Phanerotoma (Phanerotoma) excluded from the British and Irish list:

[*planifrons* (Nees, 1816, *Sigalphus*); syn. *blanda* Fahringer, 1934; *bicolor* Snoflák, 1958 preocc.; *asini* Llopis Minguez, 1968; *snoflaki* Shenefelt, 1973] Prior to van Achterberg’s (Achterberg 1990) revision, *dentata* had requently been misidentified as *planifrons*. All of the British specimens identified as *planifrons* in the BMNH are in fact *dentata*.

#### Phanerotoma (Phanerotoma) acuminata

Szépligeti, 1908

##### Distribution

England

##### Notes

added by Achterberg (1990)

#### Phanerotoma (Phanerotoma) dentata

(Panzer, 1805)

Chelonus dentatus Panzer, 1805
dentator (Nees, 1816, *Sigalphus*)
rendilea Fahringer, 1934
minor Šnoflák, 1951; synonymy by Achterberg (1990)

##### Distribution

England

##### Notes

Huddleston (1978) listed *Sigalphus rufescens* Latreille, 1809 as a junior synonym of *dentata* but this is now considered to be a separate species, not occurring in Britain or Ireland (Achterberg 1990), with historical records being misidentifications.

#### Phanerotoma (Phanerotoma) leucobasis

Kriechbaumer, 1894

ocularis Kohl, 1906
ornatulopsis de Saeger, 1942
desertorum Hedwig, 1957; synonymy by Achterberg (1990)
flavitestacea Fischer, 1939; synonymy by Achterberg (1990)
caboverdensis Hedqvist,1965

##### Distribution

England

##### Notes

Added by van Achterberg (1990). *Phanerotoma rjabovi* Vojnovskaja-Krieger, 1929 and *media* Shestakov, 1930, synonymised under *leucobasis* by Achterberg (1990), are now considered to be junior synonyms of *fracta* Kokujev, 1903 (Belokobylskij 2000b).

#### Bracotritoma

Csiki, 1909

TRITOMA Szépligeti, 1908 preocc.; synonymy by Achterberg (1990)
SZEPLIGETIA Schulz, 1911; synonymy by Achterberg (1990)
TRITOMIOS Strand, 1921; synonymy by Achterberg (1990)
UNICA Šnoflák, 1951

#### Phanerotoma (Bracotritoma) bilinea

Lyle, 1924

gregori Šnoflák, 1951

##### Distribution

England

#### Phanerotoma (Bracotritoma) tritoma

(Marshall, 1898)

Chelonus tritomus Marshall, 1898
antennalis Šnoflák, 1951; synonymy by Achterberg (1990)

##### Distribution

England, Wales, Ireland

### Doryctinae

#### Doryctinae

Förster, 1863

##### Notes

Tribal classification follows Belokobylskij (1992), but note that recent work suggests that none of the major doryctine tribes are actually monophyletic (Belokobylskij et al. 2004). Generic synonyms for the most part follow Belokobylskij et al. (2004).

#### Doryctini

Förster, 1863

#### Dendrosoter

Wesmael, 1838

EURYBOLUS Ratzeburg, 1848
CAENOPACHYS Förster, 1863

##### Notes

Although Mancini et al. (2003) argued that *Caenopachys* should be treated as a separate genus, Gebiola et al. (2015) presented molecular phylogenetic evidence that that ‘*Caenopachys*’ species (including *hartigii* in Britain) are nested within *Dendrosoter*.

#### Dendrosoter hartigii

(Ratzeburg, 1848)

Bracon hartigii Ratzeburg, 1848
flaviventris Förster, 1878
caenopachoides Ruschka, 1925; synonymy by Gebiola et al. (2015)
hartigi misspelling

##### Distribution

England

#### Dendrosoter middendorffi

(Ratzeburg, 1848)

Bracon middendorffi Ratzeburg, 1848
schimitscheki Fahringer, 1941

##### Distribution

England

##### Notes

NMS, det. Shaw, added in Fauna Europaea

#### Dendrosoter protuberans

(Nees, 1834)

Bracon protuberans Nees, 1834
insignis Förster, 1878

##### Distribution

England

#### Doryctes

Haliday, 1836

ISCHIOGONUS Wesmael, 1838
Neodoryctes Szépligeti, 1914
UDAMOLCUS Enderlein, 1920
PRISTODORYCTES Kieffer, 1921
Paradoryctes Granger, 1949
PLYCTES Fischer, 1970

#### Doryctes heydenii

Reinhard, 1865

##### Distribution

England

#### Doryctes leucogaster

(Nees, 1834)

Bracon leucogaster Nees, 1834

##### Notes

#Probably occurred only in imported timber. Its British status appears to depend solely on specimens reared in July 1908 from Austrian oak imported to a timber yard near Millwall docks (Elliot and Morley 1911).

#### Doryctes obliteratus

(Nees, 1834)

Bracon obliteratus Nees, 1834
mutillator misident.
tabidus (Haliday, 1836, *Rogas*)
brachyurus Marshall, 1888
strigatus Kokujev, 1900
petrovskii Kokujev, 1902

##### Distribution

England

##### Notes

Regarded as a synonym of *striatellus* by Belokobylskij et al. (2003).

#### Doryctes pomarius

Reinhard, 1865

schimitscheki Fahringer, 1931

##### Distribution

England

#### Doryctes rossicus

Telenga, 1941

##### Distribution

England

##### Notes

NMS, det. Shaw, added in Fauna Europaea

#### Doryctes striatellus

(Nees, 1834)

Bracon striatellus Nees, 1834
maculipes Curtis, 1837 nom. nud.
disparator (Ratzeburg, 1844, *Bracon*)
rex Marshall, 1897
striatelloides Strand, 1918
yogoi Watanabe, 1954
ambigua Kokujev, 1900
notatus Kokujev, 1900

##### Distribution

England

#### Doryctes undulatus

(Ratzeburg, 1852)

Bracon undulatus Ratzeburg, 1852

##### Distribution

England

#### Gildoria

Hedqvist, 1974

##### Notes

Removed from synonymy with *Dendrosotinus* Telenga, 1941 by Achterberg (2003b).

#### Gildoria similis

(Bouček, 1955)

Dendrosotinus similis Bouček, 1955

##### Distribution

England

##### Notes

added by Shaw (1998)

#### Ontsira

Cameron, 1900

DORYCTODES Hellén, 1927

##### Notes

Zaldívar-Riverón et al. (2008a) did not recover *Ontsira* as monophyletic and consequently some authors (e.g. Achterberg 2014) prefer to treat these species as belonging to *Doryctodes*.

#### Ontsira antica

(Wollaston, 1858)

Clinocentrus anticus Wollaston, 1858
gallica (Reinhard, 1865, *Doryctes*)
truncorum (Goureau, 1866, *Bracon*)
incerta (Ashmead, 1888, *Doryctes*)
caudalis (Hellén, 1957, *Oncophanes*)

##### Distribution

Scotland

#### Ontsira ignea

(Ratzeburg, 1852)

Bracon igneus Ratzeburg, 1852

##### Distribution

England

##### Notes

NMS, det. Shaw and van Achterberg, added here

#### Ontsira imperator

(Haliday, 1836)

Rogas imperator Haliday, 1836
zonata (Wesmael, 1838, *Ischiogonus*)
praecisa (Ratzeburg, 1852, *Bracon*)
cingulata (Provancher, 1880, *Syngaster*)
dubia (Kokujev, 1900, *Doryctes*)
iranica (Telenga, 1941, *Doryctodes*)
niger (Hedwig, 1957, *Coeloides*)

##### Distribution

England, Ireland

#### Rhaconotus

Ruthe, 1854

HEDYSOMUS Förster, 1863
HORMIOPTERUS Giraud, 1869
RHADINOGASTER Szépligeti, 1908
EURYPHRYMNUS Cameron, 1910
RHACONOTINUS Hedqvist, 1965

#### Rhaconotus aciculatus

Ruthe, 1854

cerdai Docavo Alberti, 1960
major Tobias, 1964

##### Distribution

England

##### Notes

added by Shaw (1998)

#### Wachsmannia

Szépligeti, 1900

##### Notes

Belokobylskij (1992) synonymised *Wachsmannia* under *Ontsira* but van Achterberg (1995) disagreed and synonymised *Wachsmannia* under *Hypodoryctes* Kokujev, 1900. Van Achterberg (pers. comm. and in Fauna Europaea) now regards *Wachsmannia* as a separate genus again.

#### Wachsmannia spathiiformis

(Ratzeburg, 1848)

Bracon spathiiformis Ratzeburg, 1848
maculipennis Szépligeti, 1900; synonymy by Achterberg (1995)
obliteratus misident.

##### Distribution

England

#### Ecphylini

Hellén, 1957

#### Ecphylus

Förster, 1863

TERENUSA Marshall, 1885
PARAECPHYLUS Ashmead, 1900
SACTOPUS Ashmead, 1900

##### Notes

Other species are treated as synonyms of *silesiacus* by Belokobylskij et al. (2003) but van Achterberg (in Belokobylskij et al. 2003) states that there is biological evidence for several species.

#### Ecphylus eccoptogastri

(Ratzeburg, 1848)

Bracon eccoptogastri Ratzeburg, 1848

#### Ecphylus hylesini

(Ratzeburg, 1852)

Bracon hylesini Ratzeburg, 1852

##### Distribution

England

##### Notes

NMS, det. Shaw and van Achterberg, added in Fauna Europaea

#### Ecphylus pinicola

Hedqvist, 1967

##### Distribution

England, Scotland

##### Notes

NMS, det. Shaw and van Achterberg, added in Fauna Europaea

#### Ecphylus silesiacus

(Ratzeburg, 1848)

Bracon silesiacus Ratzeburg, 1848
minutissimus (Ratzeburg, 1848, *Bracon*) preocc.

##### Distribution

England

##### Notes

NMS, det. Shaw and van Achterberg, added in Fauna Europaea; Shaw (1998) recorded *silesiacus* as British but was using the name in the broader sense (i.e. =*eccoptogastri*, =*hylesini*, =*pinicola*). Listed in Huddleston (1978) as *eccoptogastri*.

#### Hecabolini

Förster, 1863

#### Hecabolus

Curtis, 1834

ANISOPELMA Wesmael, 1838

#### Hecabolus sulcatus

Curtis, 1834

belgicus (Wesmael, 1838, *Anisopelma*)

##### Distribution

England, Wales

#### Monolexis

Förster, 1863

##### Notes

Species of *Monolexis* excluded from the British and Irish list:

[*fuscicornis* Förster, 1863; syn. *lycti* (Cresson, 1880, *Anisopelma*); *minimus* (Cresson, 1880, *Anisopelma*); *utilis* (Cresson, 1880, *Anisopelma*); *doderoi* (Mantero, 1910, *Hecabolus*); *lavagnei* Picard, 1913; *atis* Nixon, 1943] Seems to have been included on the British list only on the basis of specimens emerging from Oak (*Quercus*) timber imported from the USA (e.g. Laing 1928).

#### Heterospilus

Haliday, 1836

SYNODUS misident.
TELEBOLUS Marshall, 1888
KAREBA Cameron, 1905
ANOCATOSTIGMA Enderlein, 1920
HARPAGOLACCUS Enderlein, 1920
LITUANIA Jakimavičius, 1968

##### Notes

species of *Heterospilus* excluded from the British and Irish list:

[*caesus* (Nees, 1834, *Bracon*) misident.] Excluded by Shaw and Huddleston (1991), see also Shaw (1997).

#### Heterospilus ater

Fischer, 1960

##### Distribution

England

##### Notes

added by Shaw (1998)

#### Heterospilus fuscexilis

Shaw, 1997

##### Distribution

England

##### Notes

added by Shaw (1997)

#### Spathiini

Förster, 1863

#### Spathius

Nees, 1818

STENOPHASMUS Smith, 1859
EUSPATHIUS Förster, 1863
PSEUDOSPATHIUS Szépligeti, 1902
RHACOSPATHIUS Cameron, 1905

#### Spathius brevicaudis

Ratzeburg, 1844

##### Distribution

England, Wales

##### Notes

NMS, det. Shaw, added in Fauna Europaea

#### Spathius exarator

(Linnaeus, 1758)

Ichneumon exarator Linnaeus, 1758
formicatus (Linnaeus, 1767, *Ichneumon*)
mutillarius (Fabricius, 1775, *Ichneumon*)
mystacatus (Schrank, 1781, *Ichneumon*)
affinis (Fabricius, 1793, *Ichneumon*)
immaturus (Gravenhorst, 1807, *Ichneumon*)
clavatus (Panzer, 1809, *Cryptus*)
affinator (Thunberg, 1824, *Ichneumon*)
attenuator (Thunberg, 1824, *Ichneumon*)
formicator (Thunberg, 1824, *Ichneumon*) preocc.
mutillator (Thunberg, 1824, *Ichneumon*)
exannulatus Ratzeburg, 1848
ferrugatus Goureau, 1866
strandi Fahringer, 1930
breviterebrantus Dutu-Lacatusu, 1956

##### Distribution

England, Scotland, Wales, Ireland

#### Spathius pedestris

Wesmael, 1838

apterus Wollaston, 1858
maderi Fahringer, 1930
hirtus Hedqvist, 1976

##### Distribution

England

##### Notes

Recorded as new to Britain by Perkins and Nixon (1939). Huddleston (1978) listed this as a species in need of confirmation, although, being wingless, it is straightforward to identify. There are additional British specimens in BMNH, World Museum Liverpool, Manchester Museum and Leeds City Museum.

#### Spathius phymatodis

Fischer, 1966

##### Distribution

England

##### Notes

NMS, det. Shaw, added in Fauna Europaea

#### Spathius rubidus

(Rossius, 1794)

Ichneumon rubidus Rossius, 1794
umbrator (Thunberg, 1824, *Ichneumon*) preocc,
rugosus Ratzeburg, 1848
sculpturatus Hellén, 1927
depressus Hedqvist, 1976
bimaculatus Telenga, 1941

##### Distribution

England, Scotland, Ireland

#### Spathius umbratus

(Fabricius, 1798)

Ichneumon umbratus Fabricius, 1798
erythrocephalus Wesmael, 1838
curvicaudis Ratzeburg, 1844

##### Distribution

England

##### Notes

*Spathius curvicaudis* was recorded as new to Britain by Shaw (1988a) but synonymised under *erythrocephalus* by Belokobylskij and Samartsev (2014) and Achterberg (2014), who also synonymyised *erythrocephalus* under *umbratus*.

### Euphorinae

#### Euphorinae

Förster, 1863

##### Notes

The tribal and generic classification follows Stigenberg et al. (2015).

#### Centistini

Čapek, 1970

#### Allurus

Förster, 1863

#### Allurus lituratus

(Haliday, 1835)

Leiophron lituratus Haliday, 1835

##### Distribution

Ireland

##### Notes

Synonymised under *muricatus* by Achterberg (1985), later removed from synonymy by Achterberg (1997), with a key to the species.

#### Allurus muricatus

(Haliday, 1833)

Ancylus muricatus Haliday, 1833
armatus (Wesmael, 1835, *Leiophron*)
niger (Lyle, 1926, *Leiophron*)

##### Distribution

Scotland, Ireland

#### Centistes

Haliday, 1835

#### Ancylocentrus

Förster, 1863

##### Notes

species of Centistes (Ancylocentrus) excluded from the British and Irish list:

[*subsulcatus* (Thomson, 1895, *Leiophron*)] Not included in Huddleston (1978) but listed as British in Taxapad (Yu et al. 2012) as it was recorded by Kerrich (1932), as a tentative identification (by Roman), which has not been corroborated.

#### Centistes (Ancylocentrus) ater

(Nees, 1834)

Leiophron ater Nees, 1834
excrucians (Haliday, 1835, *Leiophron*)
lativalvis (Jakimavičius, 1972, *Allurus*)

##### Distribution

England, Ireland

#### Centistes (Ancylocentrus) collaris

(Thomson, 1895)

Leiophron collaris Thomson, 1895

##### Distribution

England, Ireland

##### Notes

BMNH, det. Mason, Broad, added here

#### Centistes (Ancylocentrus) edentatus

(Haliday, 1835)

Leiophron edentatus Haliday, 1835

##### Distribution

England, Ireland

#### Centistes (Ancylocentrus) nasutus

(Wesmael, 1838)

Eubadizon nasutus Wesmael, 1838
saxo (Reinhard, 1862, *Leiophron*)

##### Distribution

England

##### Notes

some distribution data from Luff (1976a)

#### Centistes

Haliday, 1835

ANCYLUS Haliday, 1833
ANCYLLUS Haldeman, 1842
EUPHORIDEA Ashmead, 1900
LIOSIGALPHUS Ashmead, 1900

#### Centistes (Centistes) cuspidatus

(Haliday, 1833)

Ancylus cuspidatus Haliday, 1833
lucidator (Nees, 1834, *Bracon*)

##### Distribution

England, Ireland

#### Centistes (Centistes) fuscipes

(Nees, 1834)

Bracon fuscipes Nees, 1834
fuscipes (Wesmael, 1835, *Leiophron*)

##### Distribution

England

#### Syrrhizus

Förster, 1863

#### Centistes (Syrrhizus) delusorius

(Förster, 1863)

Syrrhizus delusorius Förster, 1863

##### Distribution

England, Scotland

#### Cosmophorini

Muesebeck & Walkley, 1951

#### Cosmophorus

Ratzeburg, 1848

COSMOPHORINUS Viereck, 1925

#### Cosmophorus cembrae

Ruschka, 1925

##### Distribution

England

##### Notes

added by Shaw (1989)

#### Ropalophorus

Curtis, 1837

RHOPALOPHORUS Blanchard, 1840 emendation, preocc.
CORYNOPHORE Blanchard, 1845
EUSTALOCERUS Förster, 1863

#### Ropalophorus clavicornis

(Wesmael, 1835)

Microctonus clavicornis Wesmael, 1835
wisconsinensis Shenefelt, 1960; synonymy by Yang et al. (2003)

##### Distribution

England

#### Dinocampini

Shaw, 1985

#### Dinocampus

Förster, 1863

#### Dinocampus coccinellae

(Schrank, 1802)

Ichneumon coccinellae Schrank, 1802
terminatus (Nees, 1812, *Bracon*)
sculptus (Cresson, 1872, *Euphorus*)
americanus (Riley, 1888, *Centistes*)

##### Distribution

England, Scotland, Wales, Ireland

#### Euphorini

Förster, 1863

#### Leiophron

Nees, 1818

##### Notes

Distribution and much taxonomic data from Richards (1967), New (1970) and Loan (1974).

#### Euphorus

Nees, 1834

##### Notes

Treated as a subgenus of *Leiophron* by Belokobylskij (2000b).

#### Leiophron (Euphorus) duploclaviventris

Shenefelt, 1969

claviventris (Ruthe, 1856, *Microctonus*) preocc.
ruthei Loan, 1974

##### Distribution

England

#### Leiophron (Euphorus) pallidistigma

(Curtis, 1833)

Leiophron pallidistigma Curtis, 1833
pallicornis (Nees, 1834, *Euphorus*)
claviventris (Wesmael, 1835, *Microctonus*)
intacta (Haliday, 1835, *Leiophron*)
parvula (Ruthe, 1856, *Microctonus*)

##### Distribution

England, Ireland

#### Leiophron (Euphorus) similis

(Curtis, 1833)

Leiophron similis Curtis, 1833
basalis (Curtis, 1833, *Leiophron*)

##### Distribution

England, Wales, Ireland

##### Notes

Listed twice, under *Microctonus* and *Leiophron*, by Huddleston (1978).

#### Leiophron

Nees, 1818

Liophron Förster, 1863

#### Leiophron (Leiophron) apicalis

Haliday, 1833

apicalis Curtis, 1833 preocc.
ornata (Marshall, 1887, *Euphorus*)

##### Distribution

England, Ireland, Isle of Man

##### Notes

The type of *Euphorus ornatus* is lost but Loan (1974) suggested, on the basis of possible type material (collected in England), that *ornata* may be a synonym of *apicalis*, which has been formally proposed by Papp (2003).

#### Leiophron (Leiophron) fascipennis

(Ruthe, 1856)

Microctonus fascipennis Ruthe, 1856
fasciipennis misspelling
aciculata Belokobylskij, 1993

##### Distribution

England

#### Leiophron (Leiophron) fulvipes

Curtis, 1833

##### Distribution

England

#### Leiophron (Leiophron) heterocordyli

Richards, 1967

##### Distribution

England

#### Peristenus

Förster, 1863

##### Notes

species of *Peristenus* excluded from the British and Irish list:

[*brevicornis* (Herrich-Schäffer, 1838, *Perilitus*)] Listed by Loan (1974) as incertae sedis within *Peristenus* or *Leiophron*, by Huddleston (1978) as a doubtfully placed species of *Leiophron* and by van Achterberg (in Belokobylskij et al. 2003) as a species of *Peristenus*. We cannot trace any references to this species occurring in Britain or Ireland.

[*duplobrevicornis* (Shenefelt, 1969, *Leiophron*); syn. *brevicornis* (Ruthe, 1856, *Microctonus*) preocc.] According to Loan (1974), the type of *brevicornis* (Ruthe) is lost and the species cannot be identified with certainty. Recorded as British by Marshall (1872). Belokobylskij et al. (2003) list it as a species of *Peristenus* (=*Leiophron* according to Belokobylskij).

[*mitis* (Haliday, 1833, *Leiophron*)] According to van Achterberg (in Belokobylskij et al. 2003) the type is lost and was probably a deformed specimen anyway.

#### Peristenus accinctus

(Haliday, 1835)

Leiophron accinctus Haliday, 1835
laeviventris (Ruthe, 1856, *Microctonus*)
intermedius (Ruthe, 1856, *Microctonus*)

#### Peristenus antennalis

(Hincks, 1943) preocc.

Leiophron antennalis Hincks, 1943
picipes (Haliday, 1835, *Leiophron*) preocc.

##### Distribution

Ireland

##### Notes

Added by Achterberg (1997). Hincks’s was a replacement name for Haliday’s use of the name *picipes* for a species that is distinct from *picipes* (Curtis). Preoccupied by *Leiophron antennalis* Watanabe, 1937 (now classified in Centistes (Ancylocentrus)).

#### Peristenus facialis

(Thomson, 1892)

Euphorus facialis Thomson, 1892
fascialis misspelling
microcerus (Thomson, 1892, *Euphorus*)

##### Distribution

England, Wales

#### Peristenus grandiceps

(Thomson, 1892)

Euphorus grandiceps Thomson, 1892

##### Distribution

England, Ireland

##### Notes

Not listed by Huddleston (1978), although it was taken out of synonymy with *orchesiae* by Loan (1974).

#### Peristenus malatus

Loan, 1976

##### Distribution

Scotland

##### Notes

distribution data from Loan (1976)

#### Peristenus nitidus

(Curtis, 1833)

Leiophron nitidus Curtis, 1833

##### Distribution

England

#### Peristenus orchesiae

(Curtis, 1833)

Leiophron orchesiae Curtis, 1833
rufibarbis (Curtis, 1837, *Leiophron*)

##### Distribution

England

#### Peristenus orthotyli

(Richards, 1967)

Leiophron orthotyli Richards, 1967

##### Distribution

England

#### Peristenus pallipes

(Curtis, 1833)

Leiophron pallipes Curtis, 1833
barbiger (Wesmael, 1835, *Microctonus*)

##### Distribution

England, Scotland, Ireland, Isle of Man

##### Notes

Van Achterberg has identified a Scottish specimen as *barbiger*, currently listed as a junior synonym of *pallipes*.

#### Peristenus pallipes

(Herrich-Schäffer, 1838) preocc.

Perilitus pallipes Herrich-Schäffer, 1838
mellipes (Cresson, 1872, *Euphorus*)
punctatus (Provancher, 1883, *Microctonus*)
tuberculifer (Marshall, 1887, *Euphorus*)
nocturnus (Viereck, 1905, *Brachistes*)

#### Peristenus picipes

(Curtis, 1833)

Leiophron picipes Curtis, 1833
coactus (Marshall, 1887, *Euphorus*)

##### Distribution

England, Ireland

#### Helorimorphini

Schmiedeknecht, 1907

#### Chrysopophthorus

Goidanich, 1948

#### Chrysopophthorus hungaricus

(Kiss, 1927)

Helorimorpha hungaricus Kiss, 1927
chrysopimaginis Goidanich, 1948
elegans Tobias, 1961

##### Distribution

England

##### Notes

added by Shaw (1996a)

#### Wesmaelia

Förster, 1863

#### Wesmaelia petiolata

(Wollaston, 1858)

Euphorus petiolatus Wollaston, 1858
pendula Förster, 1863
cremasta Marshall, 1872
americana Myers, 1917
asiatica Shestakov, 1932

##### Distribution

England

#### Myiocephalini

Chen & van Achterberg, 1997

LOXOCEPHALINI Shaw, 1985 invalid

#### Myiocephalus

Marshall, 1897

LOXOCEPHALUS Förster, 1863 preocc.
SPILOMMA Morley, 1909

#### Myiocephalus boops

(Wesmael, 1835)

Microctonus boops Wesmael, 1835
longipes (Förster, 1863, *Loxocephalus*)
laticeps (Provancher, 1886, *Gamosecus*)
falconivibrans (Morley, 1909, *Spilomma*)
hedini (Fahringer, 1930, *Aphidius*)

##### Distribution

England, Ireland

#### Neoneurini

Bengtsson, 1918

ELASMOSOMINI Viereck, 1918

##### Notes

Treated as a separate subfamily in many works (e.g. Shaw and Huddleston 1991, Achterberg 1993b) but recent phylogenetic studies have shown the neoneurines to be nested within the Euphorinae (e.g. Belshaw and Quicke 2002, Dowton et al. 2002).

#### Elasmosoma

Ruthe, 1858

PARAMIRAX Ashmead, 1895 unavailable

#### Elasmosoma berolinense

Ruthe, 1858

##### Distribution

England

##### Notes

added by Shaw (2009)

#### Neoneurus

Haliday, 1838

ECCLITES Förster, 1863
SIXIA Vollenhoven, 1867; synonymy by Achterberg (1997)

#### Neoneurus auctus

(Thomson, 1895)

Elasmosoma aucta Thomson, 1895
halidaii Marshall, 1897
bistigmaticus (Morley, 1909, *Euphorus*)

##### Distribution

England, Scotland

#### Perilitini

Förster, 1863

MICROCTONINI Shaw, 1985

#### Microctonus

Wesmael, 1835

GAMOSECUS Provancher, 1880

##### Notes

Haeselbarth (2008) described many new species in Perilitus (Microctonus) but as *Microctonus* was raised to generic rank by Stigenberg et al. (2015), these are effectively new combinations in *Microctonus*. Synonymy follows Haeselbarth (2008).

#### Microctonus aciculatus

(Haeselbarth, 2008)

Perilitus aciculatus Haeselbarth, 2008

##### Distribution

Ireland

##### Notes

added by Haeselbarth (2008)

#### Microctonus aethiops

Nees, 1834

spurius Ruthe, 1856
Microctonus aethiops ?*brevispina* (Thomson, 1892, *Euphorus*)
aethiopoides Loan, 1975

##### Distribution

England, Ireland

#### Microctonus alticae

(Haeselbarth, 2008)

Perilitus alticae Haeselbarth, 2008

##### Distribution

England

##### Notes

added by Haeselbarth (2008)

#### Microctonus aphthonae

(Haeselbarth, 2008)

Perilitus aphthonae Haeselbarth, 2008

##### Distribution

England, Ireland

##### Notes

added by Haeselbarth (2008)

#### Microctonus apiophaga

Loan, 1974

#### Microctonus areolatus

(Thomson, 1892)

Perilitus areolatus Thomson, 1892

##### Distribution

England

#### Microctonus belokobylskiji

(Haeselbarth, 2008)

Perilitus belokobylskiji Haeselbarth, 2008

##### Distribution

Ireland

##### Notes

added by Haeselbarth (2008)

#### Microctonus brassicae

(Haeselbarth, 2008)

Perilitus brassicae Haeselbarth, 2008

##### Distribution

England

##### Notes

added by Haeselbarth (2008)

#### Microctonus brevicollis

(Haliday, 1835)

Perilitus brevicollis Haliday, 1835

##### Distribution

England, Ireland

#### Microctonus cerealium

(Haliday, 1835)

Perilitus cerealium Haliday, 1835
secalis (Haliday, 1833, *Perilitus*) unavailable

##### Distribution

England, Ireland

#### Microctonus colesi

Drea, 1968

##### Distribution

Scotland, Ireland

##### Notes

added by Haeselbarth (2008)

#### Microctonus consuetor

(Nees, 1834)

Perilitus consuetor Nees, 1834

##### Distribution

Scotland

##### Notes

added by Haeselbarth (2008)

#### Microctonus debilis

(Wollaston, 1858)

Perilitus debilis Wollaston, 1858
Microctonus debilis ?*gracilipes* (Thomson, 1892, *Perilitus*)

##### Distribution

Ireland

##### Notes

added by Haeselbarth (2008)

#### Microctonus fagi

(Haeselbarth, 2008)

Perilitus fagi Haeselbarth, 2008

##### Distribution

England, Ireland

##### Notes

Added by Haeselbarth (2008); specimens tentatively associated with the type material.

#### Microctonus fittkaui

(Haeselbarth, 2008)

Perilitus fittkaui Haeselbarth, 2008

##### Distribution

England

##### Notes

added by Haeselbarth (2008)

#### Microctonus flaviventris

(Thomson, 1892)

Perilitus flaviventris Thomson, 1892
areolatus (Thomson, 1892, *Perilitus*)

##### Distribution

Ireland

##### Notes

added by Haeselbarth (2008)

#### Microctonus haszprunari

(Haeselbarth, 2008)

Perilitus haszprunari Haeselbarth, 2008

##### Distribution

Ireland

##### Notes

added by Haeselbarth (2008)

#### Microctonus lipari

Čapek & Starý, 1995

##### Distribution

England

##### Notes

added by Haeselbarth (2008)

#### Microctonus melanopus

Ruthe, 1856

##### Distribution

England, Scotland, Ireland

##### Notes

added by Haeselbarth (2008)

#### Microctonus parcicornis

Ruthe, 1856

##### Distribution

England

#### Microctonus perforatus

(Haeselbarth, 2008)

Perilitus perforatus Haeselbarth, 2008

##### Distribution

England

##### Notes

added by Haeselbarth (2008)

#### Microctonus podargae

(Haeselbarth, 2008)

Perilitus podargae Haeselbarth, 2008

##### Distribution

England

##### Notes

added by Haeselbarth (2008)

#### Microctonus retusus

Ruthe, 1856

lancearius Ruthe, 1856
caudatus (Thomson, 1892, *Perilitus*)

##### Distribution

England, Ireland

##### Notes

Added by Luff (1976b); overlooked by Huddleston (1978) and O'Connor et al. (1999).

#### Microctonus silvularis

(Haeselbarth, 2008)

Perilitus silvularis Haeselbarth, 2008

##### Distribution

Scotland, Ireland

##### Notes

added by Haeselbarth (2008)

#### Microctonus stenocari

(Haeselbarth, 2008)

Perilitus stenocari Haeselbarth, 2008

##### Distribution

Scotland

##### Notes

added by Haeselbarth (2008)

#### Microctonus strophosomi

(Haeselbarth, 2008)

Perilitus strophosomi Haeselbarth, 2008

##### Distribution

Scotland, Ireland

##### Notes

added by Haeselbarth (2008)

#### Microctonus thyellae

(Haeselbarth, 2008)

Perilitus thyellae Haeselbarth, 2008

##### Distribution

England, Scotland

##### Notes

added by Haeselbarth (2008)

#### Perilitus

Nees, 1818

##### Notes

species of *Perilitus* excluded from the British and Irish list:

[*falciger* (Ruthe, 1856, *Microctonus*)] Richards (1960) states that it is not known with certainty from Britain.

#### Perilitus areolaris

Gerdin & Hedqvist, 1985

##### Distribution

England

##### Notes

added by Haeselbarth (1999)

#### Perilitus dubius

(Wesmael, 1838)

Microctonus dubius Wesmael, 1838
rutilus Herrich-Schäffer, 1838 preocc.

##### Distribution

England

#### Perilitus foveolatus

Reinhard, 1862

sicheli Giard, 1895; synonymy by Haeselbarth (1999)

##### Distribution

England, Wales

#### Perilitus marci

Haeselbarth, 1999

##### Distribution

England

##### Notes

added by Haeselbarth (1999)

#### Perilitus rutilus

(Nees, 1811)

Bracon rutilus Nees, 1811
luteus Herrich-Schäffer, 1838
ruralis Herrich-Schäffer, 1838
strenuus Marshall, 1887; synonymy by Haeselbarth (1999)
pyri (Viereck, 1917, *Dinocampus*)
tuberculus Zaykov, 1981; synonymy by Haeselbarth (1999)

##### Distribution

England, Ireland

#### Rilipertus

Haeselbarth, 1996

#### Rilipertus intricatus

(Ruthe, 1859)

Microctonus intricatus Ruthe, 1859
borealis (Thomson, 1892, *Perilitus*); synonymy by Haeselbarth (1996)

##### Distribution

Scotland, Ireland

#### Spathicopis

van Achterberg, 1977

#### flavocephala

van Achterberg, 1977

##### Distribution

England

##### Notes

NMS, det. van Achterberg, added here

#### Pygostolini

Belokobylskij, 2000

#### Pygostolus

Haliday, 1833

##### Notes

Distribution data from Achterberg (1992a) and BMNH.

#### Pygostolus falcatus

(Nees, 1834)

Leiophron falcatus Nees, 1834
testaceus misident.

##### Distribution

England, Ireland

#### Pygostolus multiarticulatus

(Ratzeburg, 1852)

Blacus multiarticulatus Ratzeburg, 1852
falcatus (Wesmael, 1838, *Blacus*) preocc.

##### Distribution

England

#### Pygostolus otiorhynchi

(Boudier, 1834)

Bracon otiorhynchi Boudier, 1834

##### Distribution

England, Scotland

##### Notes

Achterberg (1992a) separated *otiorhynchi* from *falcatus* but Belokobylskij et al. (2003), without comment, treated the two names as synonymous again. This is not followed here as *otiorhynchi* and *falcatus* seem to be distinct species.

#### Pygostolus sticticus

(Fabricius, 1798)

Ichneumon sticticus Fabricius, 1798
testaceus (Fallén, 1813, *Bassus*) preocc.
sticticator (Thunberg, 1824, *Ichneumon*)
barynoti (Boudier, 1834, *Bracon*)
gigas (Wesmael, 1835, *Blacus*)

##### Distribution

England, Scotland, Ireland, Isle of Man

#### Syntretini

Shaw, 1985

#### Syntretus

Förster, 1863

FALCOSYNTRETUS Tobias, 1965
PARASYNTRETUS Belokobylskij, 1993

##### Notes

Distribution and synonymic data from Achterberg and Haeselbarth (2003).

Species of *Syntretus* excluded from the British and Irish list:

[*parvicornis* (Ruthe, 1862, *Microctonus*)] Not listed as British or Irish by Achterberg and Haeselbarth (2003).

#### Syntretus breviradialis

van Achterberg & Haeselbarth, 2003

##### Distribution

England

##### Notes

added by Broad (2009)

#### Syntretus conterminus

(Nees, 1834)

Perilitus conterminus Nees, 1834

##### Distribution

England, Scotland, Ireland

#### Syntretus elegans

(Ruthe, 1856)

Microctonus elegans Ruthe, 1856
transsylvanicus (Kiss, 1927, *Perilitus*)

##### Distribution

Ireland

##### Notes

added by Achterberg and Haeselbarth (2003)

#### Syntretus flevo

van Achterberg & Haeselbarth, 2003

##### Distribution

England, Ireland

##### Notes

added by Achterberg and Haeselbarth (2003)

#### Syntretus fuscicoxis

van Achterberg & Haeselbarth, 2003

##### Distribution

England, Ireland

##### Notes

added by Achterberg and Haeselbarth (2003)

#### Syntretus fuscivalvis

van Achterberg & Haeselbarth, 2003

##### Distribution

England, Scotland, Ireland

##### Notes

added by Achterberg and Haeselbarth (2003)

#### Syntretus idalius

(Haliday, 1833)

Perilitus idalius Haliday, 1833
vernalis (Wesmael, 1835, *Microctonus*)
cultus (Marshall, 1887, *Microctonus*)

##### Distribution

England, Scotland, Ireland

#### Syntretus ocularis

van Achterberg & Haeselbarth, 2003

##### Distribution

England, Wales, Scotland

##### Notes

added by Achterberg and Haeselbarth (2003)

#### Syntretus politus

(Ruthe, 1856)

Microctonus politus Ruthe, 1856
cynthius (Curtis, 1837, *Microctonus*) nom. nud.
cynthius Lyle, 1927

##### Distribution

England, Ireland

#### Syntretus pusio

(Marshall, 1898)

Microctonus pusio Marshall, 1898

##### Distribution

England, Ireland

#### Syntretus splendidus

(Marshall, 1887)

Microctonus splendidus Marshall, 1887
testaceus (Capron, 1887, *Microctonus*)
suffolciensis (Morley, 1933, *Dyscritus*) **new synonymy**
niger Tobias, 1976

##### Distribution

England, Scotland, Ireland

##### Notes

The female holotype of *Dyscritus suffolciensis* Morley in BMNH, described as an aphidiine, has remained uninterpreted, although Starý (1959) recognised that it is a euphorine. It is a normal specimen of *Syntretus splendidus*.

#### Syntretus taegeri

van Achterberg & Haeselbarth, 2003

##### Distribution

England

##### Notes

added by Achterberg and Haeselbarth (2003)

#### Syntretus xanthocephalus

(Marshall, 1887)

Microctonus xanthocephalus Marshall, 1887
tempestivus (Curtis, 1837, *Microctonus*) nom. nud.
lyctaea Cole, 1959
lyctae misspelling

##### Distribution

England, Scotland, Ireland

#### Syntretus zuijleni

van Achterberg & Haeselbarth, 2003

##### Distribution

England, Scotland, Ireland

##### Notes

added by Achterberg and Haeselbarth (2003)

#### Townesilitini

Shaw, 1985

#### Streblocera

Westwood, 1833

##### Notes

*Cosmophoridia* Hedqvist, 1955 and *Eutanycerus*, usually considered synonymous with *Streblocera*, were regarded as valid subgenera by Chen and Achterberg (1997).

#### Streblocera

Westwood, 1833

LECYTHODELLA Enderlein, 1912

#### Streblocera (Streblocera) fulviceps

Westwood, 1833

##### Distribution

England

#### Streblocera (Streblocera) longiscapha

Westwood, 1882

##### Distribution

England

#### Eutanycerus

Förster, 1863

VILLOCERA Chen & van Achterberg, 1997; synonymy by Belokobylskij (2000a)

#### Streblocera (Eutanycerus) macroscapa

(Ruthe, 1856)

Microctonus macroscapus Ruthe, 1856
halidayana (Förster, 1863, *Eutanycerus*)

##### Distribution

England

#### Townesilitus

Haeselbarth & Loan, 1983

#### Townesilitus aemulus

(Ruthe, 1856)

Microctonus aemulus Ruthe, 1856
punctifrontis (Watanabe, 1955, *Microctonus*)

##### Distribution

England, Wales, Ireland

##### Notes

added by Haeselbarth (1988)

#### Townesilitus bicolor

(Wesmael, 1835)

Microctonus bicolor Wesmael, 1835
breviradialis (Tobias, 1976, *Microctonus*)

##### Distribution

England, Wales, Ireland

#### Townesilitus deceptor

(Wesmael, 1835)

Microctonus deceptor Wesmael, 1835

##### Distribution

England, Scotland, Ireland

##### Notes

added by Haeselbarth (1988)

#### Townesilitus fulviceps

(Ruthe, 1856)

Microctonus fulviceps Ruthe, 1856

##### Distribution

England

##### Notes

added by Haeselbarth (1988)

### Exothecinae

#### Exothecinae

Förster, 1863

PHANOMERINAE Fahringer, 1928

#### Colastes

Haliday, 1833

##### Notes

Belokobylskij (1998) treats *Shawiana* and *Xenarcha* as subgenera of *Colastes*.

#### Colastes

Haliday, 1833

EXOTHECUS Wesmael, 1838
PHANOMERIS Förster, 1863
PHAENOMERIS Dalla Torre, 1898

#### Colastes (Colastes) affinis

(Wesmael, 1838)

Exothecus affinis Wesmael, 1838

##### Distribution

England, Scotland, Wales

##### Notes

NMS, det. Shaw & van Achterberg, added here; treated as a species of Colastes (Xenarcha) by Belokobylskij (1998).

#### Colastes (Colastes) braconius

Haliday, 1833

debilis (Wesmael, 1838, *Exothecus*)
gracilis Papp, 1975

##### Distribution

England, Scotland, Wales, Ireland

##### Notes

some distribution data from Shaw and Askew (1976)

#### Colastes (Colastes) fragilis

(Haliday, 1836)

Rogas fragilis Haliday, 1836
semeyticus Jakimavičius, 1969

##### Distribution

England, Scotland

#### Colastes (Colastes) incertus

(Wesmael, 1838)

Exothecus incertus Wesmael, 1838

##### Distribution

England, Scotland

#### Colastes (Colastes) magdalenae

Sterzynski, 1983

##### Distribution

England

##### Notes

NMS, det. Shaw & van Achterberg, added on Fauna Europaea

#### Colastes (Colastes) pubicornis

(Thomson, 1892)

Exothecus pubicornis Thomson, 1892

##### Distribution

England, Scotland

##### Notes

added by Godfray and McGavin (1985)

#### Colastes (Colastes) vividus

Papp, 1975

##### Distribution

England

##### Notes

NMS, det. Shaw, added here

#### Fungivenator

van Achterberg & Shaw, 2008

#### Colastes (Fungivenator) sandei

van Achterberg & Shaw, 2008

##### Distribution

England

##### Notes

added by van Achterberg and Shaw (2008)

#### Shawiana

van Achterberg, 1983

PHANOMERIS misident.

#### Shawiana catenator

(Haliday, 1836)

Rogas catenator Haliday, 1836

##### Distribution

England, Scotland, Wales

##### Notes

some distribution data from Shaw and Askew (1976)

#### Shawiana laevis

(Thomson, 1892)

Exothecus laevis Thomson, 1892
rugulosus (Hellén, 1959, *Colastes*)

##### Distribution

England

##### Notes

NMS, det. Shaw, added on Fauna Europaea

#### Xenarcha

Förster, 1863

ZAMEGASPILUS Ashmead, 1900

#### Xenarcha abnormis

(Wesmael, 1838)

Exothecus abnormis Wesmael, 1838
glabricollis (Thomson, 1892, *Exothecus*)

##### Distribution

England, Scotland, Wales

##### Notes

NMS, det. Shaw, added on Fauna Europaea

#### Xenarcha lustrator

(Haliday, 1836)

Rogas lustrator Haliday, 1836
dimidiatus (Nees, 1834, *Bracon*) preocc.
lustratrix Schulz, 1906
thomsoni (Szépligeti, 1906, *Phanomeris*)

##### Distribution

England, Wales, Ireland

### Gnamptodontinae

#### Gnamptodontinae

Fischer, 1970

GNAPTODONTINAE misspelling
GNAPTOGASTRINAE Tobias, 1976

#### Gnamptodon

Haliday, 1836

GNAPTODON Haliday, 1837 suppressed
DIRAPHUS Wesmael, 1838
MESOTAGES Förster, 1863

#### Gnamptodon decoris

(Förster, 1863)

Mesotages decoris Förster, 1863
klemensiewiczii Niezabitowski, 1910
bachmaieri Fischer, 1957

##### Distribution

England, Wales

##### Notes

NMS, det. Shaw, added on Fauna Europaea

#### Gnamptodon pumilio

(Nees, 1834)

Bracon pumilio Nees, 1834
pygmaeus (Wesmael, 1838, *Diraphus*)

##### Distribution

England, Scotland, Ireland

##### Notes

some distribution data from Shaw and Askew (1976)

### Helconinae

#### Helconinae

Förster, 1863

##### Notes

The Diospilini were removed to the Brachistinae by Sharanowski et al. (2011).

#### Helconini

Förster, 1863

##### Notes

Much distribution and taxonomic data from Achterberg (1987).

#### Helcon

Nees, 1814

GYMNOSCELUS Förster, 1863
EDYIA Cameron, 1905
COELOSTEPHANUS Kieffer, 1911

#### Helcon claviventris

Wesmael, 1835

##### Distribution

England

##### Notes

added by Achterberg (1987)

#### Helcon tardator

Nees, 1812

Helcon tardator ?*adulterator* (Villers, 1789, *Ichneumon*)

##### Distribution

England

#### Helconidea

Viereck, 1914

#### Helconidea dentator

(Fabricius, 1804)

Pimpla dentator Fabricius, 1804
aequator (Nees, 1812, *Helcon*)
tentator (Thunberg, 1824, *Ichneumon*) preocc.
rugator (Ratzeburg, 1848, *Helcon*)
armator (Marshall, 1898, *Helcon*)
dentatrix (Schulz, 1906, *Pimpla*)

##### Distribution

England

##### Notes

*Helconidea armator* (Marshall, 1898, *Helcon*) removed from synonymy by Achterberg (2014).

#### Helconidea ruspator

(Linnaeus, 1758)

Ichneumon ruspator Linnaeus, 1758
dentator (Nees, 1812, *Helcon*) preocc.

##### Distribution

England

#### Wroughtonia

Cameron, 1899

DUPORTIA Kieffer, 1921

#### Wroughtonia spinator

(Lepeletier, 1825)

Helcon spinator Lepeletier, 1825
annulicornis (Nees, 1834, *Helcon*)

##### Distribution

England

### Homolobinae

#### Homolobinae

van Achterberg, 1979

#### Homolobus

Förster, 1863

ZELE misident.

##### Notes

Shaw (2010) summarises the taxonomy and biology of British and Irish species, including some distribution data.

#### Apatia

Enderlein, 1920

#### Homolobus (Apatia) truncator

(Say, 1828)

Bracon truncator Say, 1828
calcarator (Wesmael, 1835, *Phylax*)
melleus (Cresson, 1872, *Phylax*)
crassicalcaratus (Viereck, 1905, *Zele*)
calcaratrix (Schulz, 1906, *Zele*)
fuscitarsis (Bengtsson, 1918, *Phylacter*)
simillimus (Enderlein, 1920, *Apatia*)
unicolor (Enderlein, 1920, *Zele*)
chlorophthalmus (Nixon, 1938, *Zele*)

##### Distribution

England

##### Notes

Nixon’s description of *Zele chlorophthalmus* validated the use of the name for this taxon; *chlorophthalmus* of authors is a misidentification (*Bracon chlorophthalmus* Spinola, 1808 is actually a species of the true *Zele*).

#### Chartolobus

van Achterberg, 1979

#### Homolobus (Chartolobus) infumator

(Lyle, 1914)

Zele infumator Lyle, 1914
wesmaeli (Bengtsson, 1918, *Phylacter*)
japonicus (Watanabe, 1932, *Zele*)

##### Distribution

England, Scotland, Wales, Ireland

#### Homolobus

Förster, 1863

#### Homolobus (Homolobus) discolor

(Wesmael, 1835)

Phylax discolor Wesmael, 1835
pectoralis (Herrich-Schäffer, 1838, *Rogas*); synonymy by Achterberg (1992b)

##### Distribution

England, Wales, Ireland

#### Oulophus

van Achterberg, 1979

#### Homolobus (Oulophus) flagitator

(Curtis, 1837)

Zele flagitator Curtis, 1837
geminator (Lyle, 1914, *Zele*)

##### Distribution

England, Scotland, Wales, Ireland

#### Phylacter

Reinhard, 1863

#### Homolobus (Phylacter) annulicornis

(Nees, 1834)

Rogas annulicornis Nees, 1834
testaceator misident.
simplex (Herrich-Schäffer, 1838, *Rogas*); synonymy by Achterberg (1992b)

##### Distribution

England, Scotland, Ireland

### Hormiinae

#### Hormiinae

Förster, 1863

#### Hormius

Nees, 1818

CHLIDONIA Herrich-Schäffer, 1838
HORMIELLUS Enderlein, 1912
MEDIELLA Hedqvist, 1963
ANHORMIUS Belokobylskij, 1989

#### Hormius maderae

Graham, 1986

##### Distribution

England

##### Notes

NMS, det. Shaw & van Achterberg, added on Fauna Europaea

#### Hormius moniliatus

(Nees, 1811)

Bracon moniliatus Nees, 1811
brevipennis Hellén, 1957
dusmeti (Docavo Alberti, 1960, *Hormiopterus*)
insularis Hedqvist, 1965
coniceps Hellén, 1957

##### Distribution

England, Ireland, Isle of Man

#### Hormius piciventris

Wesmael, 1838

##### Distribution

England, Scotland, Wales, Ireland, Isle of Man

##### Notes

NMS, det. Shaw & van Achterberg, added on Fauna Europaea. Considered here to be a valid species; van Achterberg (in Belokobylskij et al. 2003) considered that *piciventris* may be a distinct species but it is listed as a synonym of *moniliatus* in Taxapad (Yu et al. 2012).

### Ichneutinae

#### Ichneutinae

Förster, 1863

#### Ichneutini

Förster, 1863

#### Ichneutes

Nees, 1814

#### Ichneutes brevis

Wesmael, 1835

##### Distribution

England, Ireland

#### Ichneutes reunitor

Nees, 1816

costatus (Zetterstedt, 1838, *Microgaster*)
laeviventris Hellén, 1958
leptostigma Hellén, 1958

##### Distribution

England, Ireland

#### Proteropini

van Achterberg, 1976

#### Proterops

Wesmael, 1835

ICHNEUTIDEA Ashmead, 1900
PROTEROPOIDES Viereck, 1909

#### Proterops nigripennis

Wesmael, 1835

##### Distribution

England, Scotland, Ireland

### Macrocentrinae

#### Macrocentrinae

Förster, 1863

#### Austrozele

Roman, 1910

PANISCOZELE Enderlein, 1920
PALINZELE Brues, 1922
LAEVIS Sharma, 1982

#### Austrozele longipalpis

van Achterberg, 1993

##### Distribution

England

##### Notes

added by Achterberg (1993a)

#### Macrocentrus

Curtis, 1833

AMICROPLUS Förster, 1863
AMICROPLITES Dalla Torre, 1898
FHOGRA Cameron, 1901
LEPTOZELE Cameron, 1910
METAPLEURODON Enderlein, 1920
PSEUDOPHYLACTER Fahringer, 1929

##### Notes

Distribution data from Eady and Clark (1964) and Achterberg and Haeselbarth (1983).

#### Macrocentrus bicolor

Curtis, 1833

limbator (Ratzeburg, 1848, *Rogas*)
gracilipes Telenga, 1935

##### Distribution

England, Scotland, Wales, Ireland, Isle of Man

##### Notes

Achterberg and Haeselbarth (1983) and Achterberg (1993a) treat *gracilipes* as a synonym of *bicolor* but Belokobylskij et al. (2003) list it as a synonym of *thoracicus*.

#### Macrocentrus blandus

Eady and Clark, 1964

##### Distribution

England, Scotland, Ireland

#### Macrocentrus cingulum

Brischke, 1882

grandii Goidanich, 1937
gifuensis misident.

##### Distribution

England

#### Macrocentrus collaris

(Spinola, 1808)

Bracon collaris Spinola, 1808
ebeninus (Nees, 1834, *Bracon*)
dubius (Wesmael, 1835, *Eubadizon*)
picipes (Haliday, 1835, *Helcon*)
dispar (Kollar, 1852, *Bracon*) preocc.
kollari (Rondani, 1877, *Bracon*); synonymy by Papp (1996b)
affinis Hedwig, 1961
affiniqades Shenefelt, 1969

##### Distribution

England, Ireland

#### Macrocentrus equalis

Lyle, 1914

##### Distribution

England

#### Macrocentrus infirmus

(Nees, 1834)

Rogas infirmus Nees, 1834

##### Distribution

England, Scotland, Ireland, Isle of Man

#### Macrocentrus linearis

(Nees, 1812)

Bracon linearis Nees, 1812
abdominalis (Fabricius, 1793, *Ichneumon*) preocc.
abdominator (Thunberg, 1824, *Ichneumon*)
fissura (Thunberg, 1824, *Ichneumon*)
pallidator (Zetterstedt, 1838, *Bracon*) preocc.
tenuis (Ratzeburg, 1848, *Rogas*)
iridescens French, 1880
gifuensis Ashmead, 1906
amicroploides Viereck, 1912
pallidatorius (Fahringer, 1928, *Bracon*)

##### Distribution

England, Scotland, Ireland, Isle of Man

#### Macrocentrus marginator

(Nees, 1811)

Bracon marginator Nees, 1811
rugator (Ratzeburg, 1848, *Rogas*)

##### Distribution

England, Scotland, Ireland

##### Notes

The North American *Macrocentrus aegeriae* Rohwer, 1915 was removed from synonymy by Achterberg and Haeselbarth (1983).

#### Macrocentrus nidulator

(Nees, 1834)

Rogas nidulator Nees, 1834
longicaudis (Herrich-Schäffer, 1838, *Rogas*)
procerus Costa, 1884
curticaudis Telenga, 1950

##### Distribution

England, Scotland, Wales, Ireland

#### Macrocentrus nitidus

(Wesmael, 1835)

Rogas nitidus Wesmael, 1835

##### Distribution

England, Scotland

#### Macrocentrus pallipes

(Nees, 1811)

Bracon pallipes Nees, 1811
pallidipes Dalla Torre, 1898

##### Distribution

England

#### Macrocentrus resinellae

(Linnaeus, 1758)

Ichneumon resinellae Linnaeus, 1758
resinator (Thunberg, 1824, *Ichneumon*) unavailable
flavipes (Ratzeburg, 1844, *Rogas*)
interstitialis (Ratzeburg, 1844, *Rogas*)
obscurator (Ratzeburg, 1848, *Rogas*)
intricator (Ratzeburg, 1852, *Helcon*)
punctifrons Thomson, 1895
sublaevis Thomson, 1895

##### Distribution

England, Scotland

#### Macrocentrus sylvestrellae

van Achterberg, 2001

##### Distribution

England

##### Notes

NMS, det. Shaw, added here

#### Macrocentrus thoracicus

(Nees, 1811)

Bracon thoracicus Nees, 1811
longicornis (Wesmael, 1835, *Rogas*)

##### Distribution

England, Wales

#### Macrocentrus townesi

van Achterberg & Haeselbarth, 1983

##### Distribution

England, Scotland, Ireland

##### Notes

added by Achterberg and Haeselbarth (1983)

### Meteorinae

#### Meteorinae

Cresson, 1887

ZELINAE Ashmead, 1900
ZEMIOTINAE van Achterberg, 1976

##### Notes

Stigenberg et al. (2015) included the meteorines as a basal tribe of Euphorinae, rather than as a separate subfamily, as was the preference of many authors, for example, Shaw and Huddleston (1991). In light of their very different developmental biology (parasitoids of larval Lepidoptera and Coleoptera as opposed to parasitising adult insects) and sister-group position to the euphorines, we retain the subfamily rank for Meteorinae.

#### Meteorus

Haliday, 1835

SAPROTICHUS Holmgren, 1868
PACHYTHECUS Cameron, 1912 preocc.

##### Notes

Distribution and synonymic data from Huddleston (1980), Stigenberg and Ronquist (2011) and Stigenberg & Shaw (2013), except where noted. Taxonomy follows Stigenberg and Ronquist (2011).

Species of *Meteorus* excluded from the British and Irish list:

[*flaviceps* (Ratzeburg, 1844, *Perilitus*) nom. dub.]

#### Meteorus abdominator

(Nees, 1811)

Perilitus abdominator Nees, 1811
brunnipes (Ruthe, 1862, *Perilitus*)
bruneipes Dalla Torre, 1898
brevipesalis Shenefelt, 1969
Meteorus abdominator ?*delator* (Haliday, 1835, *Perilitus*); synonymy by Achterberg (1997)

##### Distribution

England, Scotland, Wales, Ireland

#### Meteorus abscissus

Thomson, 1895

##### Distribution

England, Scotland, Ireland

#### Meteorus affinis

(Wesmael, 1835)

Perilitus affinis Wesmael, 1835
gracilis Ruthe, 1862 preocc.
punctiventris Ruthe, 1862
ruthei Schmiedeknecht, 1897
voloscensis Fischer, 1959

##### Distribution

England, Scotland, Wales, Ireland

#### Meteorus alborossicus

Lobodenko, 2000

##### Distribution

England

##### Notes

added by Stigenberg and Ronquist (2011)

#### Meteorus brevicauda

Thomson, 1895

thuringiacus Schmiedeknecht, 1897
mongolicus Fahringer, 1935

##### Distribution

England

##### Notes

Listed by Huddleston (1978) but Huddleston (1980) could not find any British or Irish material. Shaw (1988b) subsequently recorded it as a British species.

#### Meteorus cespitator

(Thunberg, 1824)

Ichneumon cespitator Thunberg, 1824
atrator (Curtis, 1832, *Zele*)
similator (Nees, 1834, *Perilitus*)
microcerus (Wesmael, 1835, *Perilitus*)
humeralis (Zetterstedt, 1838, *Bracon*)
rufipes (Zetterstedt, 1838, *Bracon*)
ambiguus Ruthe, 1862

##### Distribution

England, Scotland, Wales, Ireland

#### Meteorus cinctellus

(Spinola, 1808)

Bracon cinctellus Spinola, 1808
fuscipes (Wesmael, 1835, *Perilitus*)

##### Distribution

England, Scotland, Wales

##### Notes

This has recently been identified as *Meteorus necator* (e.g. Belokobylskij et al. 2003) but, according to Stigenberg and Ronquist (2011), *Ichneumon necator* Fabricius, 1777, is actually a species of Microgastrinae.

#### Meteorus cis

(Bouché, 1834)

Bracon cis Bouché, 1834
profligator (Haliday, 1835, *Perilitus*); synonymy by van Achterberg in Belokobylskij et al. (2003)

##### Distribution

England, Scotland, Wales, Ireland

#### Meteorus colon

(Haliday, 1835)

Perilitus colon Haliday, 1835
fragilis (Wesmael, 1835, *Perilitus*)
fasciatus (Ratzeburg, 1844, *Perilitus*)
alternatus Ruthe, 1862
continuus Ruthe, 1862
luridus Ruthe, 1862
pallidus Ruthe, 1862
trivittatus Ruthe, 1862

##### Distribution

England, Scotland, Wales, Ireland

#### Meteorus consimilis

(Nees, 1834)

Perilitus consimilis Nees, 1834
brevipes (Wesmael, 1835, *Perilitus*)
albicornis Ruthe, 1862
flagellatus Alexeev, 1971

##### Distribution

England, Ireland

#### Meteorus eadyi

Huddleston, 1980

##### Distribution

England, Wales

##### Notes

added by Huddleston (1980)

#### Meteorus eklundi

Stigenberg, 2011

##### Distribution

England

##### Notes

added by Stigenberg and Ronquist (2011)

#### Meteorus filator

(Haliday, 1835)

Perilitus filator Haliday, 1835
laticeps (Wesmael, 1835, *Perilitus*)
hodisensis Fischer, 1970

##### Distribution

England, Scotland, Ireland

#### Meteorus heliophilus

Fischer, 1970

##### Distribution

England

##### Notes

added by Huddleston (1980)

#### Meteorus hirsutipes

Huddleston, 1980

##### Distribution

England, Ireland

##### Notes

added by Huddleston (1980)

#### Meteorus ictericus

(Nees, 1811)

Bracon ictericus Nees, 1811
minutor (Thunberg, 1824, *Ichneumon*)
lucidator (Trentepohl, 1829, *Bracon*)
ephippium (Curtis, 1832, *Zele*)
xanthomelas (Wesmael, 1835, *Perilitus*)
rubriceps (Ratzeburg, 1844, *Perilitus*)
confinis Ruthe, 1862
consors Ruthe, 1862
fallax Ruthe, 1862
liquis Ruthe, 1862
pleuralis Ruthe, 1862
crassicrus Thomson, 1895
lophyriphagus Fahringer, 1934
dumbletoni Muesebeck, 1939
adoxophyesi Minamikawa, 1954
makinoharanus Minamikawa, 1954

##### Distribution

England, Scotland, Wales, Ireland, Isle of Man

#### Meteorus jaculator

(Haliday, 1835)

Perilitus jaculator Haliday, 1835
obscurellus Ruthe, 1862
tenuicornis Thomson, 1895
turcicus Fahringer, 1944

##### Distribution

England, Scotland, Wales, Ireland

#### Meteorus limbatus

Maeto, 1989

##### Distribution

England, Scotland

##### Notes

added by Stigenberg and Ronquist (2011)

#### Meteorus lionotus

Thomson, 1895

ruficoloratus Fischer, 1957

##### Distribution

England, Scotland

##### Notes

added by Huddleston (1980)

#### Meteorus longipilosus

Stigenberg, 2011

##### Distribution

England, Wales

##### Notes

added by Stigenberg and Shaw (2013)

#### Meteorus melanostictus

Capron, 1887

niger Lyle, 1913
monachae Tobias, 1986; synonymy by Belokobylskij (2000b)

##### Distribution

England

#### Meteorus micropterus

(Haliday, 1835)

Perilitus micropterus Haliday, 1835

##### Distribution

England, Scotland, Wales, Ireland

#### Meteorus obfuscatus

(Nees, 1811)

Bracon obfuscatus Nees, 1811
thoracicus (Curtis, 1832, *Zele*)
formosus (Wesmael, 1835, *Perilitus*)
orchesiae (Boie, 1841, *Alysia*)
fodori Papp, 1973

##### Distribution

England

#### Meteorus obsoletus

(Wesmael, 1835)

Perilitus obsoletus Wesmael, 1835
viridanae Johansson, 1964

##### Distribution

England, Scotland, Ireland

##### Notes

added by Huddleston (1980)

#### Meteorus oculatus

Ruthe, 1862

##### Distribution

Scotland

##### Notes

added by Stigenberg and Shaw (2013)

#### Meteorus pendulus

(Müller, 1776)

Ichneumon pendulus Müller, 1776
pendulator (Latreille, 1799, *Ichneumon*)
gyrator (Thunberg, 1824, *Ichneumon*); synonymy by van Achterberg in Belokobylskij et al. (2003)
ochraceator (Curtis, 1832, *Zele*) nom. nud.
scutellator (Nees, 1834, *Perilitus*)
petiolator (Zetterstedt, 1838, *Bracon*)
parvulus Thomson, 1895

##### Distribution

England, Scotland, Wales, Ireland

#### Meteorus pulchricornis

(Wesmael, 1835)

Perilitus pulchricornis Wesmael, 1835
striatus Thomson, 1895
thomsoni Marshall, 1899
japonicus Ashmead, 1906
nipponensis Viereck, 1912
baicalensis Telenga, 1950
graeffei Fischer, 1957
macedonicus Fischer, 1957
tuberculifer Fischer, 1957

##### Distribution

England, Scotland, Wales, Ireland

##### Notes

added by Huddleston (1980)

#### Meteorus rubens

(Nees, 1811)

Bracon rubens Nees, 1811
leviventris (Wesmael, 1835, *Perilitus*)
islandicus Ruthe, 1859
medianus Ruthe, 1862
vulgaris (Cresson, 1872, *Perilitus*)
dejanus (Rondani, 1877, *Perilitus*); synonymy by Papp (1996b)
scutatus Costa, 1884
coquilleti Ashmead, 1889
heteroneurus Thomson, 1895
mellinervis Viereck, 1903
mamestrae Viereck, 1913
szechuanensis Fahringer, 1935
mesopotamicus Fischer, 1957

##### Distribution

England, Scotland, Wales, Ireland, Isle of Man

#### Meteorus ruficeps

Nees, 1834

pallipes (Wesmael, 1835, *Perilitus*)
nigritarsis Ruthe, 1862
pallidipes Marshall, 1887

##### Distribution

England, Scotland, Ireland, Isle of Man

#### Meteorus rufus

(DeGeer, 1773)

Ichneumon rufus DeGeer, 1773
rufus (Retzius, 1783, *Ichneumon*) preocc.
unicolor (Wesmael, 1835, *Perilitus*); synonymy by van Achterberg in Belokobylskij et al. (2003)
chinensis (Holmgren, 1868, *Saprotichus*)

##### Distribution

England, Wales

#### Meteorus sibyllae

Stigenberg, 2011

##### Distribution

England, Scotland

##### Notes

added by Stigenberg and Shaw (2013)

#### Meteorus sulcatus

Szépligeti, 1896

insignis Muesebeck, 1939
molorchi Fischer, 1966

##### Distribution

England

#### Meteorus tabidus

(Wesmael, 1835)

Perilitus tabidus Wesmael, 1835
dubius Ruthe, 1862
facialis Ruthe, 1862
pentheri Fischer, 1970

##### Distribution

England, Scotland, Ireland

#### Meteorus tenellus

Marshall, 1887

boreus Tobias, 1986

##### Distribution

Scotland

##### Notes

Added by Stigenberg and Shaw (2013). Separated from *cinctellus* by Stigenberg and Ronquist (2011); Marshall (1887) gave no locality data so the first documented British occurrence was published by Stigenberg and Shaw (2013).

#### Meteorus versicolor

(Wesmael, 1835)

Perilitus versicolor Wesmael, 1835
bimaculatus (Wesmael, 1835, *Perilitus*)
unicolor (Hartig, 1838, *Perilitus*) preocc.
brevicornis (Ratzeburg, 1844, *Perilitus*) preocc.
rugator (Ratzeburg, 1852, *Perilitus*)
decoloratus Ruthe, 1862
ikonomovi Fischer, 1959
hartigi Shenefelt, 1969

##### Distribution

England, Scotland, Wales, Ireland

#### Meteorus vexator

(Haliday, 1835)

Perilitus vexator Haliday, 1835

##### Distribution

England, Scotland, Ireland

#### Zele

Curtis, 1832

PROTELUS Förster, 1863
SEMIOTES Förster, 1863

##### Notes

Synonymic and some distribution data from Achterberg (1979), Achterberg (1984b) and Stigenberg and Shaw (2013). *Zele longicauda* Curtis, 1832 is apparently a synonym of a species of *Eubazus* (van Achterberg, pers. comm.).

species of *Zele* excluded from the British and Irish list:

[*annulicrus* (Thomson, 1895, *Meteorus*)] Included in Huddleston’s (Huddleston 1978) checklist but we can trace no specimens or literature citations.

#### Zele albiditarsus

Curtis, 1832

testaceator Curtis, 1832
albitarsis (Nees, 1834, *Perilitus*)
dispar (Wesmael, 1835, *Perilitus*)
calcitrator (Curtis, 1837, *Meteorus*)
wesmaeli (Boie, 1850, *Perilitus*)
testaceatrix Schulz, 1906

##### Distribution

England, Scotland, Wales, Ireland

#### Zele caligatus

(Haliday, 1835)

Meteorus caligatus Haliday, 1835
neesii (Ruthe, 1862, *Meteorus*)
alaskensis (Ashmead, 1902, *Dyscoletes*)
sibiricus (Fahringer, 1930, *Meteorus*)

##### Distribution

England, Scotland, Wales, Ireland

#### Zele chlorophthalmus

(Spinola, 1808)

Bracon chlorophthalmus Spinola, 1808
chrysophthalmus (Nees, 1811, *Bracon*)
pallidus (Nees, 1811, *Bracon*); synonymy by Stigenberg and Ronquist (2011)
nudator (Thunberg, 1824, *Ichneumon*)
splendens (Costa, 1884, *Meteorus*)
nigricollis (Thomson, 1895, *Meteorus*)

##### Distribution

England, Scotland, Ireland

#### Zele deceptor

(Wesmael, 1835)

Perilitus deceptor Wesmael, 1835
pallitarsis (Cresson, 1872, *Perilitus*)
rufulus (Thomson, 1895, *Meteorus*)
palliditarsis (Dalla Torre, 1898, *Meteorus*)
maximus (Muesebeck, 1923, *Meteorus*)
reticulatus (Muesebeck, 1923, *Meteorus*)
romani (Fahringer, 1930, *Meteorus*)
separandus (Fischer, 1957, *Meteorus*)
metallicus (Jakimavičius, 1972, *Apanteles*)

##### Distribution

England, Scotland, Wales, Ireland

### Microgastrinae

#### Microgastrinae

Förster, 1863

##### Notes

The generic classification of Microgastrinae broadly follows Papp (1988), based on Mason’s (Mason 1981) phylogenetic treatment of microgastrines that split up the large genus ‘*Apanteles*’, which in turn built upon Nixon’s (Nixon 1965) assignment of ‘*Apanteles*’ into species groups. We do not follow van Achterberg’s (Achterberg 2003c) recent generic revision (reflected in Fauna Europaea), which reassigned many genera to *Apanteles* and *Protapanteles*. We consider this reclassification to be premature, being based on very little explicit character evidence and, with its concentration on western Palaearctic species, not really addressing the affinities of most of the world’s microgastrine species. However, there are undoubted merits to van Achterberg’s treatment as not all of the genera currently employed are well-defined. The following genera are recognised as valid by van Achterberg (2003c): *Apanteles* (=*Choeras*, *Dolichogenidea*, *Iconella*, *Illidops*, *Pholetesor*), *Cotesia*, *Deuterixys*, *Diolcogaster*, *Hygroplitis*, *Microgaster*, *Microplitis*, *Paroplitis* and *Protapanteles* (=*Distatrix*, *Glyptapanteles*, *Rasivalva*, *Sathon*). Some distribution data taken from Nixon (1965), Nixon (1968), Nixon (1970), Nixon (1972), Nixon (1973), Nixon (1974), Nixon (1976), remainder from Shaw (2012) and NMS.

#### Apantelini

Viereck, 1918

#### Apanteles

Förster, 1863

UROGASTER Ashmead, 1898
XESTAPANTELES Cameron, 1910
ALLAPANTELES Brèthes, 1915
AREOLATUS Rao & Chalikwar, 1976 unavailable

##### Notes

The generic placement of several species treated by Nixon (1973) in his *metacarpalis* group, here largely apportioned between *Apanteles* and *Dolichogenidea* following Papp (1988), is questionable (cf. Achterberg 2003c).

species of *Apanteles* excluded from the British and Irish list:

[*anomalon* (Curtis, 1830, *Microgaster*)] This name appeared in Huddleston (1978) but is not listed by Papp (1988) or Achterberg (2003c) and remains uninterpreted.

[*nigripes* (Ratzeburg, 1844, *Microgaster*)] This name appeared in Huddleston (1978) but is not listed by Papp (1988) or Achterberg (2003a).

[*picipes* (Bouché, 1834, *Microgaster*)] Papp (1987) intended to deal with this name, with a footnote in that paper saying that it would be dealt with under *A. xanthostigma*, but there is no mention there of *picipes*. It is presumed that the name *picipes* is a synonym or a nomen dubium; it is not listed as a valid species by Achterberg (2003c).

#### Apanteles atreus

Nixon, 1973

##### Distribution

England

#### Apanteles brunnistigma

Abdinbekova, 1969

sotades Nixon, 1976

##### Distribution

England, Scotland, Wales, Isle of Man

#### Apanteles carpatus

(Say, 1836)

Microgaster carpata Say, 1836
solitarius (Ashmead,1900)
hawaiiensis (Ashmead, 1901)
fuscicornis (Cameron, 1910)
piceoventris Muesebeck, 1921
igae Watanabe, 1932
sarcitorius Telenga, 1955
ultericus Telenga, 1955

##### Distribution

England

#### Apanteles chrysis

Nixon, 1973

##### Distribution

England

#### Apanteles contaminatus

(Haliday, 1834)

Microgaster contaminatus Haliday, 1834

##### Distribution

Scotland, Ireland

#### Apanteles corvinus

Reinhard, 1880

lucidus Szépligeti, 1896
rasteratus Fahringer, 1936
aptus Papp, 1977

##### Distribution

England, Scotland

#### Apanteles galleriae

Wilkinson, 1932

##### Distribution

England

##### Notes

added by Shaw (2012)

#### Apanteles lacteus

(Nees, 1834)

Microgaster lacteus Nees, 1834

##### Distribution

England

##### Notes

We follow Mason (1981) in placing *lacteus* in *Apanteles* rather than Papp’s (Papp 1988) placement in *Dolichogenidea*.

#### Apanteles lenea

Nixon, 1976

##### Distribution

England, Scotland, Ireland

#### Apanteles metacarpalis

(Thomson, 1895)

Microgaster metacarpalis Thomson, 1895

##### Distribution

England, Ireland

#### Apanteles miramis

Nixon, 1976

##### Distribution

England

#### Apanteles obscurus

(Nees, 1834)

Microgaster obscura Nees, 1834
arenarius (Haliday, 1834)

##### Distribution

England, Ireland

#### Apanteles sodalis

(Haliday, 1834)

Microgaster sodalis Haliday, 1834
carbonarius (Ratzeburg, 1848) preocc.
ater (Ratzeburg, 1852); synonymy by Achterberg (1997)
lugens (Ratzeburg, 1852)
lindbergi Hedqvist, 1965

##### Distribution

England

#### Apanteles xanthostigma

(Haliday, 1834)

Microgaster xanthostigma Haliday, 1834
ochrostigma (Wesmael, 1837)
xanthocarpus Szépligeti, 1901

##### Distribution

England, Scotland, Wales, Isle of Man

#### Choeras

Mason, 1981

#### Choeras arene

(Nixon, 1973)

Apanteles arene Nixon, 1973

##### Distribution

England, Scotland, Ireland

#### Choeras dorsalis

(Spinola, 1808)

Microgaster dorsalis Spinola, 1808
cruciatus (Ratzeburg, 1844)
suffolciensis (Morley, 1902)

##### Distribution

England, Wales

#### Choeras parasitellae

(Bouché, 1834)

Microgaster parasitellae Bouché, 1834
adjuncta misident.
flavilabris (Ratzeburg, 1844)
rufilabris (Ratzeburg, 1844)
lictorius (Reinhard, 1880)
polypori (Gautier & Bonnamour, 1930)

##### Distribution

England, Scotland

##### Notes

Listed under *Apanteles* and *Microgaster* in Huddleston (1978).

#### Choeras ruficornis

(Nees, 1834)

Microgaster ruficornis Nees, 1834
hedymeles (Nixon, 1973)

##### Distribution

England

#### Choeras tedellae

(Nixon, 1961)

Apanteles tedellae Nixon, 1961
epinotiae (Fischer, 1962)
epinoticida (Fischer, 1966)

##### Distribution

England

#### Choeras tiro

(Reinhard, 1880)

Microgaster tiro Reinhard, 1880

##### Distribution

England

#### Choeras validus

(Thomson, 1895)

Apanteles validus Thomson, 1895

##### Distribution

England

#### Dolichogenidea

Viereck, 1911

##### Notes

species of *Dolichogenidea* excluded from the British and Irish list:

[*anarsiae* (Faure & Alabouvette, 1924, *Apanteles*)] Listed as a British species by Huddleston (1978) in error, there are no British records.

[*cerialis* (Nixon, 1976, *Apanteles*); syn. *areolaris* (Balevski & Tobias, 1980, *Apanteles*) preocc.] Listed as a British species by Huddleston (1978) in error, only known from southern and Eastern Europe and Israel.

[*ensiformis* (Ratzeburg, 1844, *Microgaster*)] Listed as a British species by Huddleston (1978) in error (see note under *Napamus vipio*).

[*evonymellae* (Bouché, 1834, *Microgaster*); syn. *iarbas* (Nixon, 1972, *Apanteles*)] Listed as a British species by Huddleston (1978) in error, there are no British records.

[*impura* (Nees, 1834, *Microgaster*)] Notwithstanding Papp (1978), we regard this name as uncertainly interpreted, but in any case we have not seen British material that conforms to Papp's interpretation.

#### Dolichogenidea agilla

(Nixon, 1972)

Apanteles agilla Nixon, 1972
piratica (Papp, 1977)

##### Distribution

England

##### Notes

added by Shaw (2012)

#### Dolichogenidea annularis

(Haliday, 1834)

Microgaster annularis Haliday, 1834

##### Distribution

England

#### Dolichogenidea appellator

(Telenga, 1949)

Apanteles appellator Telenga, 1949
litae (Nixon, 1972)

##### Distribution

England

##### Notes

Added by Shaw (2012). Papp (1988) notes, but does not follow, the view that *appellator* may be the valid name (cf. Kotenko and Tobias 1986). Shaw (2012) has found that reared ‘*appellator*’ and ‘*litae*’ appear to be conspecific. The situation is complicated by Nixon's (Nixon 1972) treatment of some series (from a different host in Cyprus, and from Egypt) as ‘litae var operculellae’, and it is this that Papp (1988)lists as a junior synonym of *appellator*.

#### Dolichogenidea artissima

(Papp, 1971)

Apanteles artissimus Papp, 1971
abila (Nixon, 1972)

##### Distribution

England, Scotland, Wales

#### Dolichogenidea ate

(Nixon, 1973)

Apanteles ate Nixon, 1973

##### Distribution

England

#### Dolichogenidea bres

(Nixon, 1973)

Apanteles bres Nixon, 1973

##### Distribution

England

#### Dolichogenidea breviventris

(Ratzeburg, 1848)

Microgaster breviventris Ratzeburg, 1848
mesoxantha (Ruschka, 1917)
nilae (Telenga, 1961)

##### Distribution

England, Scotland, Wales, Ireland

#### Dolichogenidea britannica

(Wilkinson, 1941)

Apanteles britannicus Wilkinson, 1941

##### Distribution

England

#### Dolichogenidea candidata

(Haliday, 1834)

Microgaster candidatus Haliday, 1834
longicauda (Wesmael, 1837); synonymy by Achterberg (1997)
terebrator (Ratzeburg, 1852)

##### Distribution

England, Scotland, Wales, Isle of Man

#### Dolichogenidea coleophorae

(Wilkinson, 1938)

Apanteles coleophorae Wilkinson, 1938

##### Distribution

England

#### Dolichogenidea coniferae

(Haliday, 1834)

Microgaster coniferae Haliday, 1834

##### Distribution

England, Isle of Man

##### Notes

Revised status (Shaw 2012): inadvertently listed as a synonym of candidata by Achterberg (1997) due to a drafting error (van Achterberg, pers. comm.).

#### Dolichogenidea credne

(Nixon, 1973)

Apanteles credne Nixon, 1973

##### Distribution

England

#### Dolichogenidea cytherea

(Nixon, 1972)

Apanteles cytherea Nixon, 1972

##### Distribution

England

#### Dolichogenidea decora

(Haliday, 1834)

Microgaster decorus Haliday, 1834
lineata (Reinhard, 1880)
sibirica Fahringer, 1938

##### Distribution

Ireland

#### Dolichogenidea dilecta

(Haliday, 1834)

Microgaster dilectus Haliday, 1834
femoralis (Bouché, 1834)

##### Distribution

England, Isle of Man

#### Dolichogenidea drusilla

(Nixon, 1972)

Apanteles drusilla Nixon, 1972

##### Distribution

England

#### Dolichogenidea emarginata

(Nees, 1834)

Microgaster emarginatus Nees, 1834
scapularis (Bouché,1834)

##### Distribution

England, Scotland, Wales

#### Dolichogenidea exilis

(Haliday, 1834)

Microgaster exilis Haliday, 1834

##### Distribution

England

##### Notes

Shaw (2012) gives a diagnosis; not treated by Nixon or Papp.

#### Dolichogenidea faucula

(Nixon, 1972)

Apanteles faucula Nixon, 1972

##### Distribution

England

#### Dolichogenidea gagates

(Nees, 1834)

Microgaster gagates Nees, 1834

##### Distribution

England

#### Dolichogenidea glabra

(Papp, 1978)

Apanteles glaber Papp, 1978

##### Distribution

England, Scotland

##### Notes

added by Shaw (2012)

#### Dolichogenidea gracilariae

(Wilkinson, 1940)

Apanteles gracilariae Wilkinson, 1940

##### Distribution

England, Isle of Man

#### Dolichogenidea halidayi

(Marshall, 1872)

Apanteles halidayi Marshall, 1872
albipennis (Haliday, 1834) preocc.
halidaii misspelling

##### Distribution

England, Scotland, Isle of Man

#### Dolichogenidea hilaris

(Haliday, 1834)

Microgaster hilaris Haliday, 1834

##### Distribution

Ireland

#### Dolichogenidea imperator

(Wilkinson, 1939)

Apanteles imperator Wilkinson, 1939

##### Distribution

England, Scotland, Wales, Isle of Man

#### Dolichogenidea infima

(Haliday, 1834)

Microgaster infimus Haliday, 1834

##### Distribution

England

#### Dolichogenidea lacteicolor

(Viereck, 1911)

Apanteles lacteicolor Viereck, 1911
conspersae (Fiske, 1911)

##### Distribution

England

#### Dolichogenidea lacteipennis

(Curtis, 1830)

Microgaster lacteipennis Curtis, 1830
lissonota (Tobias, 1964)

#### Dolichogenidea laevigata

(Ratzeburg, 1848)

Microgaster laevigatus Ratzeburg, 1848
hoplites (Ratzeburg, 1848)
calcarata (Ivanov, 1899)

##### Distribution

England, Scotland, Wales

#### Dolichogenidea laevigatoides

(Nixon, 1972)

Apanteles laevigatoides Nixon, 1972

##### Distribution

England

#### Dolichogenidea laevissima

(Ratzeburg, 1848)

Microgaster laevissimus Ratzeburg, 1848
tersa (Papp, 1973)

##### Distribution

England

#### Dolichogenidea lemariei

(Nixon, 1961)

Apanteles lemariei Nixon, 1961

##### Distribution

England

#### Dolichogenidea lineipes

(Wesmael, 1837)

Microgaster lineipes Wesmael, 1837

##### Distribution

England, Scotland, Isle of Man

#### Dolichogenidea longicalcar

(Thomson, 1895)

Apanteles longicalcar Thomson, 1895

##### Distribution

England

#### Dolichogenidea longipalpis

(Reinhard, 1880)

Apanteles longipalpis Reinhard, 1880
tadzhica (Telenga, 1949)

##### Distribution

England, Scotland

##### Notes

Papp (1981) reports that the type series of *tadzhica* belongs to two species (*lacteus* and *longipalpis*) but did not select a lectotype; Belokobylskij et al. (2003) treated the name as a synonym of *longipalpis*.

#### Dolichogenidea marica

(Nixon, 1972)

Apanteles marica Nixon, 1972

##### Distribution

England

#### Dolichogenidea myron

(Nixon, 1973)

Apanteles myron Nixon, 1973

##### Distribution

England, Scotland

##### Notes

Transferred from *Apanteles* in anticipation of publication by Jose Fernandez-Triana.

#### Dolichogenidea ononidis

(Marshall, 1889)

Apanteles ononidis Marshall, 1889

##### Distribution

England

##### Notes

Shaw (2012) gives a diagnosis; not treated by Nixon or Papp.

#### Dolichogenidea petrovae

(Walley, 1937)

Apanteles petrovae Walley, 1937
dioryctriae (Wilkinson, 1938)
magna (Telenga, 1955)
murinanae (Čapek & Zwölfer, 1957)

##### Distribution

England

##### Notes

added by Shaw (2012)

#### Dolichogenidea phaloniae

(Wilkinson, 1940)

Apanteles phaloniae Wilkinson, 1940

##### Distribution

England, Scotland, Wales, Ireland

#### Dolichogenidea phaola

(Nixon, 1972)

Apanteles phaola Nixon, 1972

##### Distribution

England

#### Dolichogenidea praetor

(Marshall, 1885)

Apanteles praetor Marshall, 1885

##### Distribution

England

#### Dolichogenidea princeps

(Wilkinson, 1941)

Apanteles princeps Wilkinson, 1941

##### Distribution

England, Scotland, Wales

#### Dolichogenidea punctiger

(Wesmael, 1837)

Microgaster punctiger Wesmael, 1837
itea (Nixon, 1972)

##### Distribution

England, Scotland

#### Dolichogenidea sicaria

(Marshall, 1885)

Apanteles sicarius Marshall, 1885
chrysosticta (Marshall, 1899)
crudelis (Papp, 1971)

##### Distribution

England, Scotland

#### Dolichogenidea sisenna

(Nixon, 1972)

Apanteles sisenna Nixon, 1972

##### Distribution

England

##### Notes

Recorded as British by Nixon (1972) but omitted by Huddleston (1978).

#### Dolichogenidea soikai

(Nixon, 1972)

Apanteles soikai Nixon, 1972

##### Distribution

England

##### Notes

added by Shaw (2012)

#### Dolichogenidea trachala

(Nixon, 1965)

Apanteles trachalus Nixon, 1965
sevocata (Papp, 1975)

##### Distribution

England, Scotland, Wales, Ireland

##### Notes

#A parasitoid of synanthropic Lepidoptera species and probably introduced to Britain and Ireland (Nixon 1976).

#### Dolichogenidea ultor

(Reinhard, 1880)

Apanteles ultor Reinhard, 1880
lactipennis (Ratzeburg, 1852) preocc.

##### Distribution

England

#### Dolichogenidea victor

(Wilkinson, 1941)

Apanteles victor Wilkinson, 1941

##### Distribution

England

#### Illidops

Mason, 1981

#### Illidops butalidis

(Marshall, 1888)

Apanteles butalidis Marshall, 1888

##### Distribution

England, Scotland

#### Illidops naso

(Marshall, 1885)

Apanteles naso Marshall, 1885
contortus (Tobias, 1964)
crantor (Nixon, 1965)
evander (Nixon, 1965)
coresia (Nixon, 1973)

##### Distribution

England

#### Illidops suevus

(Reinhard, 1880)

Apanteles suevus Reinhard, 1880
minutus (Szépligeti, 1896)
polonicus (Fahringer, 1936)
brevisternis (Tobias, 1964)
suspicax (Tobias, 1964)
dion (Nixon, 1965)
sesostris (Nixon, 1976)

##### Distribution

England

#### Napamus

Papp, 1993

##### Notes

species of *Napamus* excluded from the British and Irish list:

[*vipio* (Reinhard, 1880, *Apanteles*)] Mistakenly listed as a British species by Kloet and Hincks (1945) and Huddleston (1978). Shenefelt (1973) listed it as British on the misunderstanding that Morley and Rait-Smith (1933) had produced a catalogue of British Lepidoptera-parasitoid asscociations, whereas their listings also included non-British rearings from hosts that occur in Britain. Morley and Rait-Smith (1933) cite Marshall (1896) as the source of host records for *vipio*, who does not mention Britain. *Apanteles vipio* was transferred from *Illidops* by Papp (1993).

#### Pholetesor

Mason, 1981

#### Pholetesor arisba

(Nixon, 1973)

Apanteles arisba Nixon, 1973

##### Distribution

England, Scotland

#### Pholetesor bicolor

(Nees, 1834)

Microgaster bicolor Nees, 1834
ardeaepenellae (Bouché, 1834)
pedias (Nixon, 1973)
umbellatarum (Haliday, 1834)
schillei (Niezabitowski, 1910)
longicauda (Fahringer, 1938)

##### Distribution

England

##### Notes

Some distribution data from Shaw and Askew (1976). Van Achterberg’s (Achterberg 1997) synonymy of *bicolor* under *circumscriptus* is not followed here (Shaw 2012); it is probable that his reared series represents two species, *bicolor* and *circumscriptus.*

#### Pholetesor circumscriptus

(Nees, 1834)

Microgaster circumscriptus Nees, 1834
exiguus (Haliday, 1834); synonymy by Achterberg (1997)
blancardellae (Bouché, 1834)
lividipes (Wesmael, 1837)
flavolimbatus (Ratzeburg, 1848)
lautellus (Marshall, 1895)

##### Distribution

England, Scotland, Wales, Ireland

#### Pholetesor elpis

(Nixon, 1973)

Apanteles elpis Nixon, 1973
girkanus (Tobias, 1976)

##### Distribution

England

#### Pholetesor errans

(Nixon, 1973)

Apanteles errans Nixon, 1973
arenicola (Papp, 1973)

##### Distribution

England

#### Pholetesor laetus

(Marshall, 1885)

Apanteles laetus Marshall, 1885
exiguus misident.
salalicus misident.
metallicus (Jakimavičius, 1972)

##### Distribution

England, Scotland

##### Notes

Shaw (2012) tentatively identified *exiguus* sensu Nixon (1973) as an extreme of morphological variation within *laetus*.

#### Pholetesor maritimus

(Wilkinson, 1941)

Apanteles maritimus Wilkinson, 1941

##### Distribution

England, Scotland, Wales

#### Pholetesor moldavicus

(Tobias, 1975)

Apanteles moldavicus Tobias, 1975

##### Distribution

England

##### Notes

Added by Shaw (2012). Although this generic placement (Papp 1988) was followed by Shaw (2012) is seems very likely to be inappropriate.

#### Pholetesor nanus

(Reinhard, 1880)

Apanteles nanus Reinhard, 1880
szoecsi (Papp, 1973)

##### Distribution

England, Scotland, Wales, Isle of Man

#### Pholetesor phaetusa

(Nixon, 1973)

Apanteles phaetusa Nixon, 1973

##### Distribution

England, Scotland

#### Pholetesor viminetorum

(Wesmael, 1837)

Microgaster viminetorum Wesmael, 1837
fuliginosus (Wesmael, 1837)

##### Distribution

England, Scotland, Wales, Ireland

#### Cotesiini

Mason, 1981

#### Cotesia

Cameron, 1891

CRYPTAPANTELES Viereck, 1910
STENOPLEURA Viereck, 1911

##### Notes

Some taxonomic and distribution data for species parasitizing Lycaenidae taken from Shaw (2007).

species of *Cotesia* excluded from the British and Irish list:

[*acuminata* (Reinhard, 1880, *Apanteles*); *cultrator* (Marshall, 1885, *Apanteles*)] Not a British or Irish species. Marshall’s description was based on unprovenanced material reared from a host that, on inspection by MRS, is almost certainly not British. The current synonymy is also in doubt.

[*ordinaria* (Ratzeburg, 1844, *Microgaster*); syn. *dendrolimi* (Matsumura, 1926, *Apanteles*); *dendrolimusi* (Matsumura, 1926, *Apanteles*)] Although listed as a British species by various authors we can find no evidence that it has occurred here.

[*saltator* (Thunberg, 1824, *Ichneumon*) preocc.] Appeared in Huddleston (1978) as Roman (1912) incorrectly synonymised *tenebrosa* under *saltator*.

[*scabricula* (Reinhard, 1880, *Apanteles*); syn. *eguchii* (Watanabe, 1935, *Apanteles*)] No evidence that this is a British or Irish species.

#### Cotesia abjecta

(Marshall, 1885)

Apanteles abjectus Marshall, 1885
complanata (Lyle, 1916, *Apanteles*)

##### Distribution

England, Scotland

#### Cotesia affinis

(Nees, 1834)

Microgaster affinis Nees, 1834
euphorbiae (Bouché, 1834, *Microgaster*)
vinulae (Bouché, 1834, *Microgaster*)
harpyiae (Niezabitowski, 1910, *Apanteles*)
okamotoi (Watanabe, 1932, *Apanteles*)
planus (Watanabe, 1932, *Apanteles*)

##### Distribution

England

#### Cotesia analis

(Nees, 1834)

Microgaster analis Nees, 1834
praetextata (Haliday, 1834, *Microgaster*)
mediana (Ratzeburg, 1852, *Microgaster*)
leucaniae (Wikinson, 1937, *Apanteles*)

##### Distribution

England

#### Cotesia astrarches

(Marshall, 1889)

Apanteles astrarches Marshall, 1889
Cotesia astrarches ?*arctica* (Thomson, 1895, *Apanteles*)
genalis (Tobias, 1964, *Apanteles*)

##### Distribution

England, Scotland, Wales

##### Notes

Nixon’s (Nixon 1974) *arctica* encompassed two species that occur in Britain, *astrarches* and *tenebrosa*. It is not clear which, if either, the name *arctica* is associated with. The name *astrarches* was placed, erroneosly, in synonymy with *arctica* by Nixon (1974), which was followed by Huddleston (1978). Shaw (2007) clarified the status of *astrarches*.

#### Cotesia bignellii

(Marshall, 1885)

Apanteles bignellii Marshall, 1885

##### Distribution

England, Scotland, Wales, Ireland

#### Cotesia brevicornis

(Wesmael, 1837)

Microgaster brevicornis Wesmael, 1837
cleoceridis (Marshall, 1889, *Apanteles*)

##### Distribution

England, Scotland

#### Cotesia cajae

(Bouché, 1834)

Microgaster cajae Bouché, 1834
difficilis (Nees, 1834, *Microgaster*)

##### Distribution

England

#### Cotesia callimone

(Nixon, 1974)

Apanteles callimone Nixon, 1974
scelerata (Tobias, 1986, *Apanteles*)

##### Distribution

Scotland, Wales, Ireland

#### Cotesia chares

(Nixon, 1965)

Apanteles chares Nixon, 1965

##### Distribution

England

#### Cotesia cleora

(Nixon, 1974)

Apanteles cleora Nixon, 1974

##### Distribution

England

#### Cotesia coryphe

(Nixon, 1974)

Apanteles coryphe Nixon, 1974

##### Distribution

England

##### Notes

Papp (1987) synonymised *coryphe* under *rubripes* but this seems unwarranted, given the distinctly different hosts and other aspects of biology.

#### Cotesia cuprea

(Lyle, 1925)

Apanteles cupreus Lyle, 1925

##### Distribution

England, Scotland

#### Cotesia errator

(Nixon, 1974)

Apanteles errator Nixon, 1974

##### Distribution

England, Wales

#### Cotesia eulipis

(Nixon, 1974)

Apanteles eulipis Nixon, 1974
eulipsis misspelling

##### Distribution

England, Scotland

#### Cotesia euryale

(Nixon, 1974)

Apanteles euryale Nixon, 1974

##### Distribution

England

##### Notes

It is not clear from Nixon (1974) that this species occurs in Britain, despite its being ‘bred in captivity at Slough’.

#### Cotesia ferruginea

(Marshall, 1885)

Apanteles ferrugineus Marshall, 1885

##### Distribution

England

#### Cotesia gades

(Nixon, 1974)

Apanteles gades Nixon, 1974

##### Distribution

England

##### Notes

added by Allen (1978)

#### Cotesia gastropachae

(Bouché, 1834)

Microgaster gastropachae Bouché, 1834

##### Distribution

England, Scotland, Isle of Man

#### Cotesia geryonis

(Marshall, 1885)

Apanteles geryonis Marshall, 1885

##### Distribution

England

#### Cotesia glomerata

(Linnaeus, 1758)

Ichneumon glomeratus Linnaeus, 1758
glomerator (Thunberg, 1824, *Ichneumon*)
nigriventris (Nees, 1834, *Microgaster*)
recondita (Nees, 1834, *Microgaster*)
stellatarum (Bouché, 1834, *Microgaster*)
crataegi (Ratzeburg, 1844, *Microgaster*)
oleracea (Taylor, 1860, *Microgaster*)
pieridis (Packard, 1881, *Microgaster*) preocc.
pieridivora (Riley, 1882, *Microgaster*)
aporiae (Ivanov, 1899, *Apanteles*)
nawaii (Ashmead, 1906, *Glyptapanteles*)
aporiae (Matsumura, 1908, *Apanteles*) preocc.
heterotergis (Fahringer, 1936, *Apanteles*)

##### Distribution

England, Scotland, Isle of Man

#### Cotesia gonopterygis

(Marshall, 1885)

Apanteles gonopterygis Marshall, 1885

##### Distribution

England

#### Cotesia hyphantriae

(Riley, 1887)

Apanteles hyphantriae Riley, 1887

##### Distribution

England, Wales

#### Cotesia inducta

(Papp, 1973)

Apanteles inductus Papp, 1973
tenuivalvis (Tobias, 1986, *Apanteles*)

##### Distribution

England, Ireland

##### Notes

added by Revels (2006); Shaw (2007)

#### Cotesia isolde

(Nixon, 1974)

Apanteles isolde Nixon, 1974

##### Distribution

England, Scotland

#### Cotesia jucunda

(Marshall, 1885)

Apanteles jucundus Marshall, 1885
nigrinervis (Thomson, 1895, *Microgaster*)

##### Distribution

England, Scotland

#### Cotesia juniperatae

(Bouché, 1834)

Microgaster juniperatae Bouché, 1834

##### Distribution

England, Scotland

#### Cotesia kurdjumovi

(Telenga, 1955)

Apanteles kurdjumovi Telenga, 1955
laverna (Nixon, 1974, *Apanteles*)

##### Distribution

England, Scotland

#### Cotesia limbata

(Marshall, 1885)

Apanteles limbatus Marshall, 1885
kawadai (Watanabe, 1934, *Apanteles*)

##### Distribution

England, Scotland

#### Cotesia lineola

(Curtis, 1830)

Microgaster lineola Curtis, 1830
gabrielis (Gautier & Riel, 1919, *Apanteles*)

##### Distribution

England

#### Cotesia melanoscela

(Ratzeburg, 1844)

Microgaster melanoscelus Ratzeburg, 1844
solitaria (Ratzeburg, 1844, *Microgaster*)
creata (Balevski, 1980, *Apanteles*)

##### Distribution

England

#### Cotesia melitaearum

(Wilkinson, 1937)

Apanteles melitaearum Wilkinson, 1937
melittaearum misspelling
ukrainica (Tobias, 1986, *Apanteles*)

##### Distribution

England, Scotland, Wales

#### Cotesia notha

(Marshall, 1885)

Apanteles nothus Marshall, 1885

##### Distribution

England, Scotland

#### Cotesia numen

(Nixon, 1974)

Apanteles numen Nixon, 1974

##### Distribution

England, Scotland

#### Cotesia ofella

(Nixon, 1974)

Apanteles ofella Nixon, 1974
Cotesia ofella ?*perspicua* (Nees, 1834, *Microgaster*)

##### Distribution

England

##### Notes

*Microgaster perspicua* is listed as a senior synonym of *cajae* in Taxapad (Yu et al. 2012), following Marshall (1885), who listed *cajae* as the senior synonym. Papp (2005a) lists *perspicua* as a tentative synonym (which would have priority) of *ofella*.

#### Cotesia onaspis

(Nixon, 1974)

Apanteles onaspis Nixon, 1974
avetyanae (Tobias, 1976, *Apanteles*)

##### Distribution

England

#### Cotesia orestes

(Nixon, 1974)

Apanteles orestes Nixon, 1974

##### Distribution

England

#### Cotesia pilicornis

(Thomson, 1890)

Microgaster pilicornis Thomson, 1890
piliflagellaris (Tobias, 1986, *Apanteles*)

##### Distribution

England, Scotland, Wales, Ireland

##### Notes

In NMS numerous series reared solitarily from various species of Pterophoridae are probably conspecific but show a great variation in extent of pilosity of the antenna.

#### Cotesia praepotens

(Haliday, 1834)

Microgaster praepotens Haliday, 1834
placida (Haliday, 1834, *Microgaster*)
memnon (Nixon, 1974, *Apanteles*)
acutivalvis (Balevski, 1980, *Apanteles*)
beshtaui (Tobias, 1986, *Apanteles*)

##### Distribution

England, Ireland

##### Notes

Nixon’s (Nixon 1974) *praepotens* is apparently correctly called *sericea* (Belokobylskij et al. 2003).

#### Cotesia risilis

(Nixon, 1974)

Apanteles risilis Nixon, 1974

##### Distribution

England

#### Cotesia rubecula

(Marshall, 1885)

Apanteles rubecula Marshall, 1885

##### Distribution

England

#### Cotesia rubripes

(Haliday, 1834)

Microgaster rubripes Haliday, 1834

##### Distribution

England, Scotland

#### Cotesia ruficrus

(Haliday, 1834)

Microgaster ruficrus Haliday, 1834
antipoda (Ashmead, 1900, *Apanteles*)
manilae (Ashmead, 1904, *Apanteles*)
sydneyensis (Cameron, 1911, *Apanteles*)
narangae (Viereck, 1913, *Apanteles*)
sesamiae (Risbec, 1956, *Apanteles*) nom. nud.

##### Distribution

England, Wales, Isle of Man

#### Cotesia salebrosa

(Marshall, 1885)

Apanteles salebrosus Marshall, 1885
callunae Nixon, 1974

##### Distribution

England, Scotland

#### Cotesia saltatoria

(Balevski, 1980)

Apanteles saltatorius Balevski, 1980

##### Distribution

England, Scotland

##### Notes

added by Shaw (2007)

#### Cotesia sericea

(Nees, 1834)

Microgaster sericeus Nees, 1834
praepotens misident.
brachycera (Thomson, 1895, *Apanteles*)

##### Distribution

England, Scotland

##### Notes

Nixon’s (Nixon 1974) *praepotens* is apparently correctly called *sericea* (Belokobylskij et al. 2003).

#### Cotesia sibyllarum

(Wilkinson, 1936)

Apanteles sibyllarum Wilkinson, 1936

##### Distribution

England

#### Cotesia spuria

(Wesmael, 1837)

Microgaster spurius Wesmael, 1837
insidens (Ratzeburg, 1844, *Microgaster*)

##### Distribution

England, Scotland, Wales, Isle of Man

#### Cotesia subordinaria

(Tobias, 1976)

Apanteles subordinarius Tobias, 1976

##### Distribution

England

##### Notes

added by Shaw (2012)

#### Cotesia telengai

(Tobias, 1972)

Apanteles telengai Tobias, 1972
amabilis (Nixon, 1974, *Apanteles*)

##### Distribution

England

#### Cotesia tenebrosa

(Wesmael, 1837)

Microgaster tenebrosus Wesmael, 1837
Cotesia tenebrosa ?*arctica* (Thomson, 1895, *Apanteles*)

##### Distribution

England, Scotland

##### Notes

Added by Shaw (2007); Nixon’s (Nixon 1974) *arctica* encompassed two species that occur in Britain, *astrarches* and *tenebrosa*. It is not clear which, if either, the name *arctica* is associated with. The name *astrarches* was placed, erroneosly, in synonymy with *arctica* by Nixon (1974), which was followed by Huddleston (1978). Shaw (2007) clarified the status of *tenebrosa*.

#### Cotesia tetrica

(Reinhard, 1880)

Apanteles tetricus Reinhard, 1880
opacula (Thomson, 1895, *Microgaster*)

##### Distribution

England, Scotland

#### Cotesia tibialis

(Curtis, 1830)

Microgaster tibialis Curtis, 1830
atrator (Curtis, 1830, *Microgaster*)
gracilis (Curtis, 1830, *Microgaster*)
congesta (Nees, 1834, *Microgaster*)
intricata (Haliday, 1834, *Microgaster*)
gracilipes (Thomson, 1895, *Microgaster*)
similis (Szépligeti, 1901, *Apanteles*)
atratrix (Schulz, 1906, *Microgaster*)
aranearum (Goureau, 1908, *Apanteles*) nom. nud.
mamestrae (Matsumura, 1908, *Apanteles*)
simulans (Lyle, 1917, *Apanteles*)
claustrata (Gautier & Bonnamour, 1923, *Apanteles*)

##### Distribution

England, Scotland, Wales

#### Cotesia vanessae

(Reinhard, 1880)

Apanteles vanessae Reinhard, 1880

##### Distribution

England

#### Cotesia vestalis

(Haliday, 1834)

Microgaster vestalis Haliday, 1834
plutellae (Kurdjumov, 1912, *Apanteles*); synonymy by Shaw (2003b)

##### Distribution

England, Wales

#### Cotesia villana

(Reinhard, 1880)

Apanteles villanus Reinhard, 1880
fasciatae (Gautier & du Dresnay, 1926, *Apanteles*)
rubroides (Papp, 1971, *Apanteles*); synonymy by Papp (2009a)

##### Distribution

England

#### Cotesia zygaenarum

(Marshall, 1885)

Apanteles zygaenarum Marshall, 1885

##### Distribution

England, Scotland, Wales, Ireland, Isle of Man

#### Deuterixys

Mason, 1981

#### Deuterixys carbonaria

(Wesmael, 1837)

Microgaster carbonarius Wesmael, 1837
anomala (Lyle, 1925, *Apanteles*)

##### Distribution

England, Scotland

#### Deuterixys plugarui

(Tobias, 1975)

Apanteles plugarui Tobias, 1975

##### Distribution

England

##### Notes

added by Shaw (2012)

#### Deuterixys rimulosa

(Niezabitowski, 1910)

Apanteles rimulosus Niezabitowski, 1910
comes (Wilkinson, 1940, *Apanteles*)

##### Distribution

England

#### Diolcogaster

Ashmead, 1901

PROTOMICROPLITIS misident.
ZADIOLOCOGASTER Viereck, 1913

##### Notes

See Mason (1981) and Fernández-Triana (2015), who restricted *Protomicroplitis* to a few Nearctic and Neotropical species.

#### Diolcogaster abdominalis

(Nees, 1834)

Microgaster abdominalis Nees, 1834

##### Distribution

England

#### Diolcogaster alvearia

(Fabricius, 1798)

Ichneumon aleuarius Fabricius, 1798
aphidum (Panzer, 1804, *Ichneumon*)
alveator (Thunberg, 1824, *Ichneumon*)
areolata (Szépligeti, 1896, *Ichneumon*)

##### Distribution

England, Scotland

#### Diolcogaster connexa

(Nees, 1834)

Microgaster connexus Nees, 1834
consularis (Haliday, 1834, *Microgaster*)
diluta (Ratzeburg, 1852, *Microgaster*)

##### Distribution

England, Isle of Man

#### Diolcogaster flavipes

(Haliday, 1834)

Microgaster flavipes Haliday, 1834

#### Diolcogaster hinzi

(Nixon, 1965)

Protomicroplitis hinzi Nixon, 1965

##### Distribution

England, Scotland

##### Notes

added by Shaw (2012)

#### Diolcogaster minuta

(Reinhard, 1880)

Microgaster minutus Reinhard, 1880

##### Distribution

England, Scotland

#### Diolcogaster scotica

(Marshall, 1885)

Microgaster scoticus Marshall, 1885

##### Distribution

England, Scotland

#### Diolcogaster spreta

(Marshall, 1885)

Microgaster spretus Marshall, 1885

##### Distribution

England, Scotland, Wales

#### Distatrix

Mason, 1981

#### Distatrix formosa

(Wesmael, 1837)

Microgaster formosus Wesmael, 1837
marshallii (Bignell, 1901, *Apanteles*)

##### Distribution

England, Wales, Isle of Man

#### Glyptapanteles

Ashmead, 1905

##### Notes

species of *Glyptapanteles* excluded from the British and Irish list:

[*thompsoni* (Lyle, 1917, *Apanteles*)] Listed as a British species by Huddleston (1978) in error; there is no evidence that it has occurred here.

#### Glyptapanteles acasta

(Nixon, 1973)

Apanteles acasta Nixon, 1973

##### Distribution

England

#### Glyptapanteles aliphera

(Nixon, 1973)

Apanteles aliphera Nixon, 1973
aliphaera misspelling
sublateralis (Tobias, 1976, *Apanteles*)

##### Distribution

England, Scotland

#### Glyptapanteles callidus

(Haliday, 1834)

Microgaster callidus Haliday, 1834
urolus (Papp, 1983, *Apanteles*)

##### Distribution

England, Scotland, Wales

##### Notes

Achterberg (1997) reinterpreted this name and treated *majalis* as the valid name for the species called *callidus* by Nixon (1973) and Papp (1983).

#### Glyptapanteles compressiventris

(Muesebeck, 1921)

Apanteles compressiventris Muesebeck, 1921
liparidis misident.

##### Distribution

Scotland, Wales

##### Notes

Wilkinson (1945) dealt with the identity of the real *liparidis*, which does not occur in Britain or Ireland.

#### Glyptapanteles fausta

(Nixon, 1973)

Apanteles fausta Nixon, 1973

##### Distribution

England, Scotland

##### Notes

Generic placement in doubt; some authors place both *fausta* and *lateralis* in *Sathon*. Papp (1983) synonymised *fausta* under *Apanteles eugeni* Papp, 1972 but later (Papp 1988) rescinded his action. Unfortunately Papp (1972) had chosen a non-reared specimen as holotype but a paratype of *eugeni* reared from *Anthophila fabriciana* (Linnaeus) is in BMNH and appears to be conspecific with *fausta* (described from this host). It seems probable that Papp's (Papp 1983) synonymy was justified but, as this requires further investigation, the name *fausta* is retained for now.

#### Glyptapanteles fraternus

(Reinhard, 1881)

Apanteles fraternus Reinhard, 1881

##### Distribution

England

#### Glyptapanteles fulvipes

(Haliday, 1834)

Microgaster fulvipes Haliday, 1834

##### Distribution

England, Scotland, Wales, Ireland

#### Glyptapanteles inclusus

(Ratzeburg, 1844)

Microgaster inclusus Ratzeburg, 1844
curvulus (Thomson, 1895, *Microgaster*)
rectinervis (Telenga, 1955, *Apanteles*)

##### Distribution

England

#### Glyptapanteles lateralis

(Haliday, 1834)

Microgaster lateralis Haliday, 1834

##### Distribution

England, Scotland, Wales

##### Notes

Generic placement in doubt; some authors place both *fausta* and *lateralis* in *Sathon*.

#### Glyptapanteles luciana

(Nixon, 1973)

Apanteles luciana Nixon, 1973

##### Distribution

Scotland

##### Notes

Described by Nixon (1973) partly from Scottish material but omitted by Huddleston (1978).

#### Glyptapanteles majalis

(Wesmael, 1837)

Microgaster majalis Wesmael, 1837
callidus misident.

##### Distribution

England, Scotland

##### Notes

Achterberg (1997) treated *majalis* as the valid name for the species called *callidus* by Nixon (1973) and Papp (1983).

#### Glyptapanteles menander

(Nixon, 1973)

Apanteles menander Nixon, 1973

##### Distribution

Scotland

#### Glyptapanteles mygdonia

(Nixon, 1973)

Apanteles mygdonia Nixon, 1973

##### Distribution

England, Scotland, Ireland

#### Glyptapanteles octonarius

(Ratzeburg, 1852)

Microgaster octonarius Ratzeburg, 1852
stauropodis (Bridgman, 1889, *Apanteles*)
lucifugus (Lyle, 1917, *Apanteles*)

##### Distribution

England

#### Glyptapanteles pallipes

(Reinhard, 1880)

Apanteles pallipes Reinhard, 1880
pallidipes (Marshall, 1885, *Apanteles*)
longicornis (Provancher, 1886, *Microgaster*)
radiatus (Ashmead, 1898, *Apanteles*)
reinhardi (Wilkinson, 1936, *Apanteles*)

##### Distribution

England, Scotland, Wales

#### Glyptapanteles pinicola

(Lyle, 1917)

Apanteles pinicola Lyle, 1917

##### Distribution

England, Scotland

#### Glyptapanteles porthetriae

(Muesebeck, 1928)

Apanteles porthetriae Muesebeck, 1928

##### Distribution

England

##### Notes

added by Shaw and Skelton (2008)

#### Glyptapanteles salepus

(Papp, 1983)

Apanteles salepus Papp, 1983

##### Distribution

England

##### Notes

added by Shaw (2012)

#### Glyptapanteles vitripennis

(Curtis, 1830)

Microgaster vitripennis Curtis, 1830
fulcriger (Wesmael, 1837, *Microgaster*)
impavidus (Gautier & Dresnyay, 1927, *Apanteles*)

##### Distribution

England, Scotland, Isle of Man

#### Protapanteles

Ashmead, 1898

#### Protapanteles anchisiades

(Nixon, 1973)

Apanteles anchisiades Nixon, 1973

##### Distribution

England, Scotland, Wales, Isle of Man

#### Protapanteles endemus

(Nixon, 1965)

Apanteles endemus Nixon, 1965

##### Distribution

England, Scotland

#### Protapanteles enephes

(Nixon, 1965)

Apanteles enephes Nixon, 1965

##### Distribution

England

#### Protapanteles hirtariae

(Kotenko & Tobias, 1986)

Apanteles hirtariae Kotenko & Tobias, 1986

##### Distribution

Scotland

##### Notes

added by Shaw (2012)

#### Protapanteles immunis

(Haliday, 1834)

Microgaster immunis Haliday, 1834

##### Distribution

England, Scotland, Wales, Ireland

#### Protapanteles incertus

(Ruthe, 1859)

Microgaster incertus Ruthe, 1859
caberae (Marshall, 1885, *Apanteles*)
jugosus (Lyle, 1916, *Apanteles*)
mihalyii (Papp, 1973, *Apanteles*)

##### Distribution

England, Scotland, Isle of Man

#### Protapanteles parallelus

(Lyle, 1917)

Apanteles parallelus Lyle, 1917
lylei (Shenefelt, 1972, *Apanteles*)

##### Distribution

England

#### Protapanteles popularis

(Haliday, 1834)

Microgaster popularis Haliday, 1834

##### Distribution

England, Scotland

#### Protapanteles triangulator

(Wesmael, 1837)

Microgaster triangulator Wesmael, 1837

##### Distribution

England

##### Notes

Included in *Protapanteles* by Papp (1988) but Mason (1981) treated it as a species of *Glyptapanteles*. Nixon (1973) was unable to suggest close relatives.

#### Rasivalva

Mason, 1981

#### Rasivalva calceata

(Haliday, 1834)

Microgaster calceatus Haliday, 1834
pubescens (Ratzeburg, 1844, *Microgaster*)

##### Distribution

England, Scotland

#### Rasivalva circumvecta

(Lyle, 1918)

Diolcogaster circumvectus Lyle, 1918

##### Distribution

England, Scotland

#### Rasivalva marginata

(Nees, 1834)

Microgaster marginatus Nees, 1834

##### Distribution

England, Scotland

#### Microgastrini

Förster, 1863

#### Hygroplitis

Thomson, 1895

#### Hygroplitis pseudorussata

Shaw, 1992

##### Distribution

England

##### Notes

added by Shaw (1992b)

#### Hygroplitis rugulosa

(Nees, 1834)

Microgaster rugulosus Nees, 1834
infumata (Haliday, 1834, *Microgaster*)
opaca (Ruthe, 1858, *Microgaster*)

##### Distribution

England, Wales, Ireland

#### Hygroplitis russata

(Haliday, 1834)

Microgaster russatus Haliday, 1834
dimidiata (Wesmael, 1837, *Microgaster*)
basalis (Stephens, 1846, *Microgaster*)
aomoriensis (Matsumura, 1910, *Microgaster*)

##### Distribution

England, Scotland, Wales

##### Notes

Specimen from Islay in Hunterian Museum, Glasgow, det. MRS.

#### Iconella

Mason, 1981

##### Notes

species of *Iconella* excluded from the British and Irish list:

[*lacteoides* (Nixon, 1965, *Apanteles*); syn. *memorabilis* (Alexeev, 1971, *Apanteles*)]

[*merula* (Reinhard, 1880, *Apanteles*); syn. ?*etiellae* (Viereck, 1911, *Apanteles*)] Both of these species were mistakenly listed by Huddleston (1978); there is no evidence that they are British or Irish.

#### Iconella aeola

(Nixon, 1965)

Apanteles aeolus Nixon, 1965

##### Distribution

England

##### Notes

added by Shaw (2012)

#### Microgaster

Latreille, 1804

LIGANIRA Walker, 1860
LISSOGASTER Bengtsson, 1926

##### Notes

The current usage of the name *Microgaster* was restored by Opinion 1510 (ICZN 1988), after temporarily being applied to the genus here called *Microplitis* (with *Microgaster* as currently understood being referred to *Lissogaster*). This was an unfortunate name change as it coincided with Papp’s (Papp 1976, Papp 1984) revisions of the genera, as well as several other important papers. Unless noted otherwise, distribution data taken from NMS, Nixon (1968) and Shaw (2012). Papp’s (Papp 1976) subsequent revision of the genus added many species to Nixon’s (Nixon 1968) revision, and Achterberg (1997) established the precedence of several Haliday names.

species of *Microgaster* excluded from the British and Irish list:

[*auriculata* (Fabricius, 1804, *Ichneumon*)] Listed as doubtfully British by Huddleston (1978) on the basis of Papp’s (Papp 1976) listing of ‘?England’. No evidence that this is really a British or Irish species.

[*deceptor* Nixon, 1968] Listed as a British species by Huddleston (1978) in error; no evidence that this is a British or Irish species.

[*fischeri* Papp, 1960] British specimens, misidentified as *fischeri* by Nixon (1968), represented an undescribed species which was described by Shaw (2012) as *raschkiellae*.

[*nobilis* Reinhard, 1880; syn. *compressifemur* Fahringer, 1937] Listed as doubtfully British by Huddleston (1978) on the basis of Papp’s (Papp 1976) listing of ‘?England’. No evidence that this is really a British or Irish species.

[*postica* Nees, 1834; syn. *marginella* Wesmael, 1837; ?*ruficoxis* Ruthe, 1858] Recorded as British by Marshall (1885), but probably in error as his diagnosis appears not to have been a *Microgaster* species in the modern sense. Papp’s (Papp 1976) listing of England probably simply reflects Marshall’s record, as does the listing in Huddleston (1978), and there is no evidence that this is a British or Irish species.

#### Microgaster acilia

Nixon, 1968

##### Distribution

England

##### Notes

Raised from synonymy under *meridiana* (Papp 1999b) by Shaw (2012).

#### Microgaster alebion

Nixon, 1968

##### Distribution

England, Scotland

##### Notes

distribution data from Shaw (2004)

#### Microgaster areolaris

Thomson, 1895

##### Distribution

England, Scotland

#### Microgaster arctostaphylica

Shaw, 2012

##### Distribution

Scotland

##### Notes

added by Shaw (2012)

#### Microgaster consors

Nixon, 1968

##### Distribution

England

#### Microgaster crassicornis

Ruthe, 1860

##### Distribution

England

#### Microgaster ductilis

Nixon, 1968

##### Distribution

England

##### Notes

added by Shaw (2012)

#### Microgaster fulvicrus

Thomson, 1895

striatoscutellaris Kiss, 1927

##### Distribution

England, Scotland, Ireland, Isle of Man

#### Microgaster globata

(Linnaeus, 1758)

Ichneumon globatus Linnaeus, 1758
laeviscuta Thomson, 1895
gossypina (Retzius, 1783, *Ichneumon*)
globator (Thunberg, 1824, *Ichneumon*)
anthomyiarum Bouché, 1834
amentorum Ratzeburg, 1844
incurvata Papp, 1976; synonymy by Papp (2002)

##### Distribution

England, Scotland

##### Notes

Some distribution data from Papp (1976). It seems that *laeviscuta* has been included as a junior synonym of *hospes* in Fauna Europaea, with *globata* not referred to. Shaw (2012) notes that *globata* as perceived by Papp (1976) is almost certainly an aggregate.

#### Microgaster hospes

Marshall, 1885

comptanae Viereck, 1911

##### Distribution

England, Scotland, Wales

#### Microgaster luctuosa

Haliday, 1834

curvicrus Thomson, 1895; synonymy by Achterberg (1997)

##### Distribution

England, Scotland, Wales

#### Microgaster meridiana

Haliday, 1834

spinolae Haliday, 1834 preocc.; synonymy by Achterberg (1997)
alexis Haliday, 1834 nom. nud.
grandis Thomson, 1895; synonymy by Achterberg (1997)
contubernalis Marshall, 1898

##### Distribution

England, Scotland, Wales, Ireland, Isle of Man

#### Microgaster messoria

Haliday, 1834

tibialis Nees, 1834 preocc.; synonymy by Achterberg (1997)
vulgaris Ruthe, 1860
pluto Morley, 1936

##### Distribution

England, Scotland, Wales, Ireland

##### Notes

Some Ruthe names traditionally regarded as synonyms of *tibialis* probably do not belong here Shaw (2012).

#### Microgaster nigricans

Nees, 1834

##### Notes

A species of doubtful status. Curtis (1837) listed *nigricans* as a British species and Papp (1976), who stated that it was known only from the male, therefore listed it as English. Nixon (1968), however, did not deal with the name.

#### Microgaster nixalebion

Shaw, 2004

##### Distribution

England, Wales

##### Notes

added by Shaw (2004)

#### Microgaster novicia

Marshall, 1885

swammerdamiae Muesebeck, 1922

##### Distribution

England, Scotland

#### Microgaster opheltes

Nixon, 1968

##### Distribution

Ireland

#### Microgaster pantographae

Muesebeck, 1922

##### Distribution

England

#### Microgaster parvistriga

Thomson, 1895

##### Distribution

England, Scotland

#### Microgaster polita

Marshall, 1885

carinata Bengtsson, 1926 preocc.
bengtssoni Fahringer, 1937

##### Distribution

England, Scotland, Ireland

#### Microgaster procera

Ruthe, 1860

intermedia Ivanov, 1899

##### Distribution

Ireland

#### Microgaster raschkiellae

Shaw, 2012

fischeri misident.

##### Distribution

England, Scotland, Wales

##### Notes

added by Shaw (2012)

#### Microgaster stictica

Ruthe, 1858

confusa Papp, 1971

##### Distribution

England, Scotland, Wales, Isle of Man

#### Microgaster subcompleta

Nees, 1834

annulipes Curtis, 1830
carinata Packard, 1881

##### Distribution

England, Scotland, Wales, Ireland, Isle of Man

#### Paroplitis

Mason, 1981

#### Paroplitis wesmaeli

(Ruthe, 1860)

Microgaster wesmaeli Ruthe, 1860
picipes (Wesmael, 1837, *Microgaster*) preocc.

##### Distribution

England

#### Sathon

Mason, 1981

#### Sathon falcatus

(Nees, 1834)

Microgaster falcatus Nees, 1834
equestris (Haliday, 1834, *Microgaster*)
gladiator (Szépligeti, 1901, *Apanteles*)

##### Distribution

England, Scotland, Wales, Ireland

#### Microplitini

Mason, 1981

#### Microplitis

Förster, 1863

DAPSILOTOMA Cameron, 1906

##### Notes

Unless noted otherwise, distribution data taken from Nixon (1970). Papp (1984) extensively revised the synonymy (as *Microgaster* species: unfortunately at that time *Microplitis* was briefly being regarded as a synonym of *Microgaster*, but this was subsequently rescinded by Opinion 1510 (ICZN 1988), whose conclusions are largely followed here.

species of *Microplitis* excluded from the British and Irish list:

[*eremitus* Reinhard, 1880] No evidence that this species has occurred in Britain or Ireland; probably listed in Shenefelt (1973) and Huddleston (1978) because Nixon (1970) reported its distribution as north-west European.

#### Microplitis aduncus

(Ruthe, 1860)

Microgaster aduncus Ruthe, 1860
brachycerus (Thomson, 1895, *Microgaster*)

##### Distribution

Scotland

#### Microplitis decens

Tobias, 1964

##### Distribution

Scotland

##### Notes

Added by Papp (1984) but it is unclear on what basis this species was listed as British (Shaw 2012).

#### Microplitis deprimator

(Fabricius, 1798)

Ichneumon deprimator Fabricius, 1798
ingratus (Haliday, 1834, *Microgaster*); synonymy by Achterberg (1997)
sordipes (Nees, 1834, *Microgaster*)
tau (Ratzeburg, 1852, *Microgaster*)
deprimatrix (Schulz, 1906, *Microgaster*)

##### Distribution

England, Scotland

##### Notes

*Micropoltis sordipes* is listed as a separate species in Taxapad (Yu et al. 2012) but we follow van Achterberg, in Fauna Europaea. However, the status of *sordipes* remains uncertain.

#### Microplitis flavipalpis

(Brullé, 1832)

Microgaster flavipalpis Brullé, 1832
ruricola Lyle, 1918

##### Distribution

England

#### Microplitis fordi

Nixon, 1970

##### Distribution

England, Scotland

##### Notes

Papp (1984) suggests that *semicircularis* (Ratzeburg, 1844, *Microgaster*) (type destroyed) may be a senior synonym.

#### Microplitis fulvicornis

(Wesmael, 1837)

Microgaster fulvicornis Wesmael, 1837
calcarata misident.
pallidicornis Marshall, 1898

##### Distribution

England, Wales

#### Microplitis impressus

(Wesmael, 1837)

Microgaster impressus Wesmael, 1837
sispes Nixon, 1970

##### Distribution

England

##### Notes

added by Shaw (2012)

#### Microplitis lugubris

(Ruthe, 1860)

Microgaster lugubris Ruthe, 1860
borealis Marshall, 1885
coracinus (Thomson, 1895, *Microgaster*)
rutheana Fahringer, 1937

##### Distribution

Scotland

#### Microplitis malimbus

(Papp, 1984)

Microgaster malimba Papp, 1984
trochanterata misident

##### Distribution

England

##### Notes

Added by Shaw (2012). Nixon’s (Nixon 1970) interpretation of *trochanterata* (not *tuberculifer*, of which *trochanterata* is a junior synonym) is actually referable to *malimbus* (Shaw 2012).

#### Microplitis mandibularis

Thomson, 1895

##### Distribution

England, Scotland

#### Microplitis mediator

(Haliday, 1834)

Microgaster mediator Haliday, 1834
medianus (Ruthe, 1860, *Microgaster*)
halidayi Fahringer, 1937
pseudomedianus Fahringer, 1937

##### Distribution

England, Scotland, Wales, Ireland, Isle of Man

#### Microplitis moestus

(Ratzeburg, 1852)

Microgaster moestus Ratzeburg, 1852

#### Microplitis naenia

Nixon, 1970

##### Distribution

England

#### Microplitis ocellatae

(Bouché, 1834)

Microgaster ocellatae Bouché, 1834
canaliculatus (Wesmael, 1837, *Microgaster*)

##### Distribution

England

#### Microplitis scrophulariae

Szépligeti, 1898

##### Distribution

England

##### Notes

added by Shaw (2012)

#### Microplitis sofron

Nixon, 1970

Microplitis sofron ?*stigmaticus* (Ratzeburg, 1844, *Microgaster*)

##### Distribution

England, Scotland, Ireland

##### Notes

Papp (1984) suggests that *stigmaticus* may be a senior synonym.

#### Microplitis spectabilis

(Haliday, 1834)

Microgaster spectabilis Haliday, 1834
fossulatus (Bouché, 1834, *Microgaster*)
Microplitis spectabilis ?*parvulus* (Ruthe, 1860, *Microgaster*)
seuratii Marshall, 1898
testaceipes (Cameron, 1906, *Dapsilotoma*)

##### Distribution

England, Wales, Ireland

#### Microplitis spinolae

(Nees, 1834)

Microgaster spinolae Nees, 1834
sapporoensis Ashmead, 1906
radiorimatus Telenga, 1955
Microplitis spinolae ?*quadridentatus* (Provancher, 1886, *Microgaster*)

##### Distribution

England

#### Microplitis strenuus

Reinhard, 1880

gracilis (Ruthe, 1860, *Microgaster*) preocc.

##### Distribution

England

#### Microplitis tristis

(Nees, 1834)

Microgaster tristis Nees, 1834
dolens Marshall, 1885

##### Distribution

England, Scotland, Wales

#### Microplitis tuberculatus

(Bouché, 1834)

Microgaster tuberculatus Bouché, 1834
fumipennis (Ratzeburg, 1852, *Microgaster*)

##### Distribution

England, Scotland, Ireland

#### Microplitis tuberculifer

(Wesmael, 1837)

Microgaster tuberculifer Wesmael, 1837
calcaratus (Thomson, 1895, *Microgaster*)
trochanteratus (Thomson, 1895, *Microgaster*)
manevali Gautier & Bonnamour, 1939

##### Distribution

England, Scotland

#### Microplitis viduus

(Ruthe, 1860)

Microgaster viduus Ruthe, 1860

##### Distribution

England

#### Microplitis xanthopus

(Ruthe, 1860)

Microgaster xanthopus Ruthe, 1860
tenuipes (Thomson, 1895, *Microgaster*)

##### Distribution

Scotland, Wales, Ireland

### Microtypinae

#### Microtypinae

Szépligeti, 1908

##### Notes

Belokobylskij et al. (2003) include *Microtypus*, without comment, in the Orgilinae, where it had traditionally been placed prior to recent phylogenetic work. In the molecular phylogeny of Belshaw and Quicke (2002) the genus *Microtypus* is the sister group to Homolobinae, as also found by Sharanowski et al. (2011) (albeit with this clade the sister group to Orgilinae). This relationship was originally suggested by Achterberg (1984b).

#### Microtypus

Ratzeburg, 1848

SIMILEARINUS Glowacki & Karpiński, 1967

##### Notes

Nomenclature follows Capek and Achterberg (1992).

#### Microtypus wesmaelii

Ratzeburg, 1848

dioryctriae Rohwer, 1920

##### Distribution

England

##### Notes

added by Shaw (1992a)

### Miracinae

#### Miracinae

Viereck, 1918

#### Mirax

Haliday, 1833

CENTISTIDEA Rohwer, 1914

##### Notes

There are also several unrecognised species in Britain.

#### Mirax rufilabris

Haliday, 1833

spartii Haliday, 1835
dryochares Marshall, 1898
nanivorae Fischer, 1957

##### Distribution

England, Scotland

##### Notes

some distribution data from Shaw and Askew (1976) and NMS

### Opiinae

#### Opiinae

Blanchard, 1845

##### Notes

The generic and tribal classification of opiines has been largely chaotic (Wharton 1988b). In Fauna Europaea, van Achterberg has enacted many taxonomic changes resulting from his work on Western Palaearctic Opiinae (in prep.), which are followed here. Some changes to the generic classification have been published by Wharton (1988b), van Achterberg (Achterberg 2004a, Achterberg 2004b, and in Belokobylskij et al. 2003) and by Li et al. (2013). Wharton (1988b) argued against recognition of tribes within Opiinae, except perhaps Ademonini (for the genus *Ademon*), with all the other genera in Opiini; van Achterberg (in prep.) does not recognise Ademonini. Some distribution data from Fischer (1958), Fischer (1967), Fischer (1997).

#### Ademon

Haliday, 1833

GIARDINAIA de Stefani-Perez, 1902
ANALOSTANIA Viereck, 1916

#### Ademon decrescens

(Nees, 1811)

Bracon decrescens Nees, 1811
mutuator (Nees, 1811, *Bracon*)

##### Distribution

England, Scotland, Wales, Ireland

#### Apodesmia

Förster, 1863

ALLOTYPUS Förster, 1863
LEMNAPHILOPIUS Fischer, 1972
AGNOPIUS Fischer, 1982
CRYPTOGNATHOPIUS Fischer, 1984

##### Notes

generic synonymy follows Li et al. (2013)

#### Apodesmia aemula

(Haliday, 1836)

Opius aemulus Haliday, 1836
melba (Papp, 1978, *Opius*)

##### Distribution

England, Ireland

#### Apodesmia curvata

(Fischer, 1957)

Opius curvatus Fischer, 1957

##### Distribution

England, Scotland

##### Notes

added by Godfray (1986)

#### Apodesmia irregularis

(Wesmael, 1835)

Opius irregularis Wesmael, 1835
bipustulata (Fischer, 1958, *Opius*)

##### Distribution

England, Ireland

#### Apodesmia ocellata

(Wesmael, 1835)

Opius ocellatus Wesmael, 1835
areolaris (Thomson, 1895, *Opius*)
hungarica (Szépligeti, 1896, *Opius*)
bruta (Papp, 1978, *Opius*)

##### Distribution

England, Scotland

#### Apodesmia posticatae

(Fischer, 1957)

Opius posticatae Fischer, 1957
seebensteinensis (Fischer, 1959, *Opius*)
hilaris (Fischer, 1963, *Opius*)
hostium (Fischer, 1964, *Opius*)

##### Distribution

England, Scotland

#### Apodesmia rufipes

(Wesmael, 1835)

Opius rufipes Wesmael, 1835
taeniata Förster, 1863
taeniata (Fischer, 1957, *Opius*) preocc.

##### Distribution

England, Ireland

#### Apodesmia saeva

(Haliday, 1837)

Opius saevus Haliday, 1837

##### Distribution

England, Scotland

#### Apodesmia saevula

(Fischer, 1958)

Opius saevulus Fischer, 1958

##### Distribution

England

#### Apodesmia similis

(Szépligeti, 1898)

Opius similis Szépligeti, 1898
xylostei (Marshall, 1898, *Opius*)
similiformis (Fischer, 1957, *Opius*)
basirufa (Fischer, 1958, *Opius*)
nodata (Fischer, 1958, *Opius*)
periclymenii (Fischer, 1964, *Opius*)
altimontana (Fischer, 1969, *Opius*)
differens (Fischer, 1958, *Opius*)
parvipunctum (Fischer, 1958, *Opius*)
selkirkensis (Fischer, 2006, *Opius*); synonymy by van Achterberg (in prep.)

##### Distribution

England, Scotland, Ireland

#### Atormus

van Achterberg, 1998

#### Atormus victus

(Haliday, 1837)

Opius victus Haliday, 1837
tarni (Papp, 1982, *Opius*)

##### Distribution

England, Scotland, Ireland

##### Notes

Listed as a synonym of *Opius singularis* in Huddleston (1978).

#### Bathystomus

Förster, 1863

COMPRESSARIA Königsmann, 1959

##### Notes

Although Wharton (1993) noted that the type (and only included) species of *Compressaria* is the same species as the type of *Bathystomus*, he did not formally synonymise these names and the synonymy has not been picked up on by, e.g. Belokobylskij et al. (2003) and Taxapad (Yu et al. 2012). Wharton (1988b) demonstrated that *Bathystomus* is a valid genus but Belokobylskij et al. (2003) treated it as a synonym of *Diachasma*. Van Achterberg (2014) formally synonymised *Compressaria* and *pugnatrix*. Huddleston (1978) included *Compressaria pugnatrix* as a species of Rogadinae.

#### Bathystomus xanthopus

Förster, 1863

pugnatrix (Marshall, 1895, *Mesocrina*)
compressiventris (Fischer, 1964, *Opius*); synonymy by van Achterberg (in prep.)

##### Distribution

England

#### Biophthora

Förster, 1863

##### Notes

Treated as a valid genus following Wharton (2006).

#### Biophthora bajula

(Haliday, 1837)

Opius bajulus Haliday, 1837
beieri (Fischer, 1968, *Sternaulopius*); synonymy by Wharton (2006)

##### Distribution

England, Ireland

#### Biosteres

Förster, 1863

RHABDOSPILUS Förster, 1863
RHINOPLUS Förster, 1863
STENOSPILUS Förster, 1863
ZETETES Förster, 1863 preocc.
OPIELLUS Ashmead, 1900
CELIESTIELLA Cameron, 1903

#### Biosteres analis

(Wesmael, 1835)

Opius analis Wesmael, 1835
colorativentris (Fischer, 1957, *Opius*)

##### Distribution

England, Scotland

#### Biosteres arenarius

(Stelfox, 1959)

Opius arenarius Stelfox, 1959

##### Distribution

Ireland

#### Biosteres bicolor

Wesmael, 1835

vagator (Förster, 1863, *Stenospilus*)

##### Distribution

Ireland

#### Biosteres carbonarius

(Nees, 1834)

Bracon carbonarius Nees, 1834
impressus (Wesmael, 1835, *Opius*)
procerus (Wesmael, 1835, *Opius*)
onzi (Fischer, 1959, *Opius*); synonymy by Achterberg (2014)

##### Distribution

England, Scotland, Wales, Ireland

##### Notes

Synonymised under *carbonarius* by Achterberg (1975), *impressus* was removed from synonymy by Fischer (1997), a move which is not accepted by van Achterberg (in prep.). Supporting van Achterberg’s synonymy, Godfray (unpublished) has repeatedly reared specimens resembling both ‘species’ from collections of hosts made at the same place and time.

#### Biosteres haemorrhoeus

(Haliday, 1837)

Opius haemorrhoeus Haliday, 1837
castaneiventris (Thomson, 1895, *Opius*)
palaearcticus Szépligeti, 1901

##### Distribution

England, Ireland

#### Biosteres magnicornis

(Wesmael, 1835)

Opius magnicornis Wesmael, 1835

##### Distribution

England, Ireland

#### Biosteres micans

(Stelfox, 1957)

Opius micans Stelfox, 1957
nitidus (Stelfox, 1949, *Opius*) preocc.

##### Distribution

Scotland, Ireland

#### Biosteres placidus

(Haliday, 1837)

Opius placidus Haliday, 1837
melanocerus (Wesmael, 1838, *Opius*)
tarsator (Thomson, 1895, *Opius*)
indotatus Viereck,1905

##### Distribution

England, Ireland

#### Biosteres rusticus

(Haliday, 1837)

Opius rusticus Haliday, 1837

##### Distribution

England, Scotland, Ireland

#### Biosteres scabriculus

(Wesmael, 1835)

Opius scabriculus Wesmael, 1835

##### Distribution

Ireland

#### Biosteres spinaciae

(Thomson, 1895)

Opius spinaciae Thomson, 1895
pegomyiae (Gahan, 1917, *Opius*)
hyoscyamiellus (Viereck, 1925, *Opius*)

##### Distribution

England

##### Notes

added by Godfray (1988)

#### Biosteres sylvaticus

(Haliday, 1837)

Opius sylvaticus Haliday, 1837
clypealis (Thomson, 1895, *Opius*)
nitidus Szépligeti, 1896

##### Distribution

England, Scotland, Ireland

#### Biosteres wesmaelii

(Haliday, 1837)

Opius wesmaelii Haliday, 1837
carbonarius (Wesmael, 1835, *Opius*) preocc.
ultor (Förster, 1863, *Zetetes*)
ultor (Fischer, 1957, *Opius*) preocc.
jonaitisi (Jakimavi?ius, 1977, *Opius*)

##### Distribution

England, Scotland

#### Bitomoides

van Achterberg, 2004

#### Bitomoides rugosus

(Wesmael, 1838)

Opius rugosus Wesmael, 1838
rugiventris (Thomson, 1895, *Opius*); synonymy by Achterberg (2014)

##### Distribution

England

##### Notes

Listed as British by Kloet and Hincks (1945) but omitted by Huddleston (1978). Material in NMS has been identified by van Achterberg.

#### Chilotrichia

Förster, 1863

TRICHOPIUS Thomson, 1895

#### Chilotrichia blanda

(Haliday, 1837)

Opius blandus Haliday, 1837

##### Distribution

England, Ireland

#### Desmiostoma

Förster, 1863

##### Notes

Regarded by Wharton (Wharton 1983, Wharton 1988b) as a junior synonym of *Opius*.

#### Desmiostoma parvulum

(Wesmael, 1835)

Opius parvulus Wesmael, 1835
nudiscutum (Fischer, 1964, *Opius*)
ziratus (Papp, 1982, *Opius*)
temporale (Fischer, 1958, *Opius*)

##### Distribution

England, Scotland

#### Diachasma

Förster, 1863

Atoreuteus Förster, 1863
Lytacra Förster, 1863
Alysopius Tobias, 1976

#### Diachasma caffer

(Wesmael, 1835)

Opius caffer Wesmael, 1835
stygium (Förster, 1863, *Lytacra*)

#### Diachasma cephalotes

(Wesmael, 1835)

Opius cephalotes Wesmael, 1835

##### Distribution

England

#### Diachasma fulgidum

(Haliday, 1837)

Opius fulgidus Haliday, 1837

##### Distribution

England, Ireland

#### Eurytenes

Förster, 1863

#### Eurytenes

Förster, 1863

#### Eurytenes (Eurytenes) abnormis

(Wesmael, 1835)

Opius abnormis Wesmael, 1835

##### Distribution

England, Ireland

##### Notes

According to Walker and Wharton (2011), *Opius paradoxus* Ratzeburg, 1848, sometimes treated as a valid name (synonymous with *abnormis*) should be regarded as invalid as it was first proposed as a synonym of *abnormis*; Walker and Wharton (2011) also provide some locality data.

#### Eurytenes (Eurytenes) britannicola

Fischer, 2006

##### Distribution

England

##### Notes

Added by Fischer (2006): mis-placed in *Eurytenes* (van Achterberg, pers. comm.).

#### Stigmatopoea

Fischer, 1986

##### Notes

The generic name *Stigmatopoea* has been treated as a synonym of *Xynobius* by Achterberg (2004a) but the type species, *macrocerus*, has been regarded as a species of *Eurytenes*, a genus which was ignored by Achterberg (2004a). Both *Xynobius* and *Stigmatopoea* are treated as subgenera of *Eurytenes*, following Wharton (2006), although Li et al. (2013) again synonymised *Stigmatopoea* with *Xynobius*.

#### Eurytenes (Stigmatopoea) macrocerus

(Thomson, 1895)

Opius macrocerus Thomson, 1895
hians (Stelfox, 1949, *Opius*)

##### Distribution

England, Scotland, Ireland

#### Xynobius

Förster, 1863

ACLISIS Förster, 1863
HOLCONOTUS Förster, 1863
AULONOTUS Ashmead, 1900
ERISTERNAULAX Viereck, 1914
XYNOBIOTENES Fischer, 1998

##### Notes

Generic synonymy from Achterberg (2004a) and Li et al. (2013) but treated as a subgenus of *Eurytenes* by Wharton (2006).

#### Eurytenes (Xynobius) aciculatus

(Thomson, 1895)

Opius aciculatus Thomson, 1895
tenuicornis (Thomson, 1895, *Opius*); synonymy by van Achterberg (in prep.)

##### Distribution

Ireland

#### Eurytenes (Xynobius) aemuloides

(Fischer, 1958)

Opius aemuloides Fischer, 1958

##### Distribution

England

#### Eurytenes (Xynobius) caelatus

(Haliday, 1837)

Opius caelatus Haliday, 1837
isomera (Förster, 1863, *Aclisis*)
pallipes Förster, 1863
pallidipes Dalla Torre, 1898

##### Distribution

England, Scotland, Ireland

#### Eurytenes (Xynobius) comatus

(Wesmael, 1835)

Opius comatus Wesmael, 1835
sulcifer (Papp, 1967, *Dapsilarthra*)

##### Distribution

England, Ireland

#### Eurytenes (Xynobius) geniculatus

(Thomson, 1895)

Opius geniculatus Thomson, 1895
albicoxis (Marshall, 1898, *Opius*)

##### Distribution

England, Scotland

#### Eurytenes (Xynobius) holconotus

(Fischer, 1958)

Opius holconotus Fischer, 1958

##### Distribution

England

#### Eurytenes (Xynobius) maculipes

Wesmael, 1835

addendus Fischer, 1959
turcmenicus Fischer, 1959

##### Distribution

England, Ireland

#### Eurytenes (Xynobius) polyzonius

(Wesmael, 1835)

Opius polyzonius Wesmael, 1835

##### Distribution

England, Scotland

#### Eurytenes (Xynobius) silenis

(Fischer, 1967)

Diachasma silenis Fischer, 1967

##### Distribution

Wales

##### Notes

Described from Welsh material (Fischer 1967) but omitted by Huddleston (1978).

#### Eurytenes (Xynobius) thomsoni

(Fischer, 1971)

Opius thomsoni Fischer, 1971
annulicornis (Thomson, 1895, *Opius*) preocc.

##### Distribution

England

#### Eurytenes (Xynobius) sp. A

van Achterberg, in prep.

##### Distribution

England

##### Notes

reared by Godfray, in NMS

#### Neopius

Gahan, 1917

##### Notes

Raised from synonymy with *Phaedrotoma* by Li et al. (2013).

#### Neopius rudis

(Wesmael, 1835)

Opius rudis Wesmael, 1835
carinaticeps Gahan, 1917

##### Distribution

England, Ireland

#### Opiognathus

Fischer, 1972

##### Notes

Raised from synonymy with *Phaedrotoma* by Li et al. (2013).

#### Opiognathus pactus

(Haliday, 1837)

Opius pactus Haliday, 1837

##### Distribution

England, Ireland

#### Opiostomus

Fischer, 1972

SNOFLAKOPIUS Fischer, 1972
JUCUNDOPIUS Fischer, 1984
OETZALOTENES Fischer, 1998
OPIOTENES Fischer, 1998

##### Notes

Raised from synonymy with *Opius* by Li et al. (2013), who report the generic synonymy.

#### Opiostomus aureliae

(Fischer, 1957)

Opius aureliae Fischer, 1957

##### Distribution

England, Scotland

##### Notes

added by Godfray and Achterberg (2015)

#### Opiostomus campanariae

(Fischer, 1959)

Opius campanariae Fischer, 1959

##### Distribution

Scotland

##### Notes

added by Godfray and Achterberg (2015)

#### Opiostomus griffithsi

(Fischer, 1962)

Opius griffithsi Fischer, 1962

##### Distribution

England

#### Opiostomus leptostigma

(Wesmael, 1835)

Opius leptostigma Wesmael, 1835
percontator (Fischer, 1964, *Opius*)

##### Distribution

England, Scotland

##### Notes

Recorded as a British species by various authors, latterly by Fischer (1967), and reared recently by Godfray (unpublished), but omitted by Huddleston (1978).

#### Opius

Wesmael, 1835

CRYPTONASTES Förster, 1863
HYPOCYNODUS Förster, 1863
HYPOLABIS Förster, 1863
MISOPHTHORA Förster, 1863
DESMATOPHORUS Thomson, 1895
ALLOPHLEBUS Fischer,1972
NOSOPAEOPIUS Fischer, 1972
OPIOTHORAX Fischer, 1972
PENDOPIUS Fischer, 1972
STOMOSEMA Fischer, 1972
ODONTOPOEA Fischer, 1987

##### Notes

Restricted by Achterberg and Salvo (1997) to a group of species with distinctive mandibles.

#### Opius agromyzicola

Fischer, 1967

##### Distribution

England

#### Opius ambiguus

Wesmael, 1835

celsus Haliday, 1837; synonymy by Achterberg (2014)
longipes Fischer, 1957
phytomyzae Fischer, 1957

##### Distribution

England, Scotland, Ireland

#### Opius brevipalpis

Thomson, 1895

mutus Fischer, 1964
gyoerfii Fischer, 1958; synonymy by Achterberg (2014)

##### Distribution

England

#### Opius caudifer

Fischer, 1958

longicornis misident.

##### Distribution

England

##### Notes

Listed as a synonym of longicornis Thomson, 1895, in Taxapad (Yu et al. 2012), regarded as a separate, valid species by van Achterberg (in prep.). Recorded as British by Fischer (1967) as *longicornis* but omitted by Huddleston (1978).

#### Opius cingulatus

Wesmael, 1835

dentifer Thomson, 1895
stramineipes Thomson, 1895

#### Opius compar

Marshall, 1894

pulchrithorax Fischer, 1958

#### Opius crassipes

Wesmael, 1835

##### Distribution

England

#### Opius flammeus

Fischer, 1959

##### Distribution

England

#### Opius funebris

Wesmael, 1835

latipes misident.

##### Distribution

England, Ireland

##### Notes

Listed as a synonym of *pygmaeator* in Taxapad (Yu et al. 2012), regarded as a separate, valid species (=*latipes* sensu Fischer) by van Achterberg (in prep.).

#### Opius fuscipennis

Wesmael, 1835

##### Distribution

England

##### Notes

Fischer (1960) mistakenly listed this species as *fuscipennis* (Szépligeti, 1914, *Rhinoplus*) (van Achterberg, pers. comm.), a separate species now placed in the genus *Pseudorhinoplus* Fischer, 1972.

#### Opius gracilis

Fischer, 1957

csikii Fischer, 1957
minor Fischer, 1957
nigrithorax Fischer, 1958 preocc.

##### Distribution

England

#### Opius instabilis

Wesmael, 1835

#### Opius levis

Wesmael, 1835

apiculator (Nees, 1834, *Bracon*)
filicornis Thomson, 1895
varipes Szépligeti, 1898

##### Distribution

England, Wales, Ireland

##### Notes

*Opius filicornis* was recorded as new to Britain by Godfray (1986) but synonymised with *levis* by van Achterberg (in Belokobylskij et al. 2003).

#### Opius lucidus

Szépligeti, 1896

##### Distribution

England, Scotland

#### Opius lugens

Haliday, 1837

abscissus Thomson, 1895
obscurus Szépligeti, 1901
adveniens Fischer, 1960

##### Distribution

England, Ireland

#### Opius nigricoloratus

Fischer, 1958

dureseaui Fischer, 1975; synonymy by Achterberg (2014)

##### Distribution

Scotland, Wales

##### Notes

added by Godfray and Achterberg (2015)

#### Opius ochrogaster

Wesmael, 1835

nigriceps Szépligeti, 1898
neopusillus Fischer, 1957

##### Distribution

England, Scotland, Ireland

#### Opius orbiculator

(Nees, 1811)

Bracon orbiculator Nees, 1811
breviscapus Thomson, 1895

##### Distribution

Ireland

#### Opius pallipes

Wesmael, 1835

exilis Haliday, 1837; synonymy by Achterberg (1997)
pallidipes (Marshall, 1872, *Hypolabis*)
liopleuris Thomson, 1895
piceus Thomson, 1895
adaequator (Fischer, 1964, *Hypolabis*) nom. nud.
lividipes (Fischer, 1964, *Hypolabis*) nom. nud.
subsulcatus (Fischer, 1964, *Hypolabis*) nom. nud.
extusus Papp, 1981
cisromensis Papp, 1982

##### Distribution

England, Scotland, Wales, Ireland

#### Opius pendulus

Haliday, 1837

latipes Fischer, 1958

##### Distribution

England, Ireland

#### Opius phytobiae

Fischer, 1959

##### Distribution

England

##### Notes

added by Godfray (1986)

#### Opius propodealis

Fischer, 1958

##### Distribution

England

#### Opius pygmaeator

(Nees, 1811)

Bracon pygmaeator Nees, 1811
ruminans Fischer, 1957
dilatatus Fischer, 1960
meracus Fischer, 1960

##### Distribution

England, Ireland

#### Opius pygmaeus

Fischer, 1962

##### Distribution

England

#### Opius singularis

Wesmael, 1835

clarus Haliday, 1836; synonymy by Fischer (1997)
spretus Haliday, 1836; synonymy by Fischer (1997)
vindex Haliday, 1837; synonymy by van Achterberg (in prep.)
arenosus Szépligeti, 1898

##### Distribution

England, Ireland

##### Notes

Both *clarus* and *spretus* were also synonymised by Achterberg (1997) but Fischer’s publication pre-dated this.

#### Opius soenderupianus

Fischer, 1967

##### Distribution

England

##### Notes

added by Godfray and Achterberg (2015)

#### Opius tenellae

Fischer, 1969

##### Distribution

Scotland, Ireland

#### Phaedrotoma

Förster, 1863

EUTRICHOPSIS Förster, 1863
NOSOPOEA Förster, 1863
TOLBIA Cameron, 1907
BRACHYCENTRUS Szépligeti, 1907
COELOREUTEUS Roman, 1910
HEXAULAX Cameron, 1910
BAEOCENTRUM Schulz, 1911
NEODIOSPILUS Szépligeti, 1911
NEOPIUS Fischer, 1965
EUOPIUS Fischer, 1967
GASTROSEMA Fischer, 1972
GERIUS Fischer, 1972
GRIMNIRUS Fischer, 1972
HOENIRUS Fischer, 1972
MEROTRACHYS Fischer, 1972
MIMIRUS Fischer, 1972
PHLEBOSEMA Fischer, 1972
NEOEPHEDRUS Samanta, Tamili, Saha & Raychaudhuri, 1983
ADONTOPIUS Fischer, 1984
KAINOPAEOPIUS Fischer, 1987
MILLENIOPIUS Fischer, 1996
NEOTROPOPIUS Fischer, 1999

##### Notes

*Phaedrotoma* was raised from synonymy with *Opius* by Achterberg and Salvo (1997) to accommodate many species previously placed in *Opius*. Generic synonymy follows Li et al. (2013).

species of *Phaedrotoma* excluded from the British and Irish list:

[*viennensis* (Fischer, 1959, *Opius*)] Listed by Huddleston (1978) but we cannot trace any published records or specimens.

#### Phaedrotoma aethiops

(Haliday, 1837)

Opius aethiops Haliday, 1837

##### Distribution

England, Ireland

#### Phaedrotoma caesa

(Haliday, 1837)

Opius caesus Haliday, 1837
punctiventris (Thomson, 1895, *Opius*)
subtilis (Szépligeti, 1898, *Opius*)
hydrelliae (Rimsky-Korsakov, 1925, *Opius*)
hydrelliae (Muesebeck, 1933, *Opius*) preocc.
aquatica (Muesebeck, 1967, *Opius*)
hydrelliana (Fischer, 1971, *Opius*)

##### Distribution

England, Ireland

#### Phaedrotoma curvata

(Fischer, 1957)

Opius curvatus Fischer, 1957

##### Distribution

Scotland

##### Notes

added by Godfray (1986)

#### Phaedrotoma decorata

(Stelfox, 1949)

Opius decoratus Stelfox, 1949

##### Distribution

Ireland

#### Phaedrotoma depeculator

(Förster, 1863)

Opius depeculator Förster, 1863
semiaciculata (Stelfox, 1949, *Opius*)

##### Distribution

England, Ireland

#### Phaedrotoma diversa

(Szépligeti, 1898)

Opius diversus Szépligeti, 1898

##### Distribution

England

##### Notes

Recorded as British by Fischer (1960) but omitted by Huddleston (1978).

#### Phaedrotoma exigua

(Wesmael, 1835)

Opius exiguus Wesmael, 1835

##### Distribution

England, Ireland

#### Phaedrotoma fallax

(Szépligeti, 1896)

Opius fallax Szépligeti, 1896

##### Distribution

England

##### Notes

Listed as a synonym of *Opius instabilis* in Taxapad (Yu et al. 2012) but regarded as a valid species, in *Phaedrotoma*, by van Achterberg (in prep.).

#### Phaedrotoma fasciata

(Thomson, 1895)

Opius fasciatus Thomson, 1895
comparanda (Fischer, 1958, *Opius*)

##### Distribution

England

#### Phaedrotoma heringi

(Fischer, 1962)

Opius heringi Fischer, 1962

##### Distribution

Ireland

#### Phaedrotoma instabiloides

(Fischer, 1959)

Opius instabiloides Fischer, 1959

##### Distribution

Ireland

#### Phaedrotoma minusculae

(Fischer, 1967)

Opius minusculae Fischer, 1967

##### Distribution

England

#### Phaedrotoma monticola

(Szépligeti, 1898)

Opius monticola Szépligeti, 1898

##### Distribution

England

##### Notes

added by Fischer (1997)

#### Phaedrotoma munda

(Förster, 1863)

Eutrichopsis munda Förster, 1863
munda (Fischer, 1957, *Opius*) preocc.

##### Distribution

England

#### Phaedrotoma nitidulator

(Nees, 1834)

Opius nitidulator Nees, 1834
vittata (Ruschka, 1915, *Opius*) preocc.

#### Phaedrotoma novojariae

(Fischer, 2006)

Opius novojariae Fischer, 2006
stigmatocauda (Fischer, 2006, *Eurytenes*); synonymy by van Achterberg (in prep.)

##### Distribution

England

##### Notes

added by Fischer (2006)

#### Phaedrotoma paraphytomyzae

(Fischer, 2005)

Opius paraphytomyzae Fischer, 2005

##### Distribution

England

##### Notes

added by Fischer (2005)

#### Phaedrotoma pulchriceps

(Szépligeti, 1898)

Opius pulchriceps Szépligeti, 1898
ilicis (Nixon, 1939, *Opius*)
pulcherrimus (Fischer, 1958, *Opius*)
pulchriventris (Fischer, 1958, *Opius*); synonymy by Achterberg (2014)
vexator (Fischer, 1964, *Opius*)
affectus (Papp, 1981, *Opius*)

##### Distribution

England, Ireland

#### Phaedrotoma recondes

van Achterberg, 2004

reconditor misident.

##### Notes

Added by van Achterberg in Fauna Europaea; described by van Achterberg because the material named Opius reconditor (now classified in *Rhogadopsis*) by authors (e.g. Fischer 1972, Fischer and Koponen 1999) is not conspecific with the type. Both species apparently occur in Britain.

#### Phaedrotoma reptantis

(Fischer, 1957)

Opius reptantis Fischer, 1957

##### Distribution

England, Scotland

##### Notes

Listed as a synonym of *Opius ambiguus* in Taxapad (Yu et al. 2012) but regarded as a valid species, in *Phaedrotoma*, by van Achterberg (in prep.).

#### Phaedrotoma rex

(Fischer, 1958)

Opius rex Fischer, 1958

##### Distribution

England

#### Phaedrotoma rudiformis

(Fischer, 1958)

Opius rudiformis Fischer, 1958
uligiloci (Fischer, 2006, *Opius*); synonymy by Achterberg (2014)

##### Distribution

England

#### Phaedrotoma staryi

Fischer, 1958

sitagrus Papp, 1982

##### Distribution

England, Scotland

#### Phaedrotoma tacita

(Haliday, 1837)

Opius tacitus Haliday, 1837

##### Distribution

England

##### Notes

omitted by Huddleston (1978)

#### Phaedrotoma variegata

(Szepligeti, 1896)

Opius variegatus Szepligeti, 1896

##### Distribution

England, Scotland, Ireland

#### Rhogadopsis

Brèthes, 1913

LISSOSEMA Fischer, 1972

##### Notes

Raised from synonymy with *Phaedrotoma* by Li et al. (2013).

#### Rhogadopsis reconditor

(Wesmael, 1835)

Opius reconditor Wesmael, 1835
docilis (Haliday, 1837, *Opius*)
parvungula Thomson, 1895

##### Distribution

England, Ireland

##### Notes

*Opius docilis* was synonymised with *reconditor* by Achterberg (1997) but listed as a separate species in Taxapad (Yu et al. 2012); re-synonymised by Achterberg (2014).

#### Utetes

Förster, 1863

THEROBOLUS Förster, 1863
FREKIUS Fischer, 1971; synonymy by Wharton (2006)

##### Notes

species of *Utetes* excluded from the British and Irish list:

[*christenseni* (Papp, 1982, *Opius*)] Listed in Fauna Europaea as occurring in Britain, but presumably mistakenly as no literature or specimen records can be located. Known only from Georgia, Greece and Ukraine (data from Yu et al. 2012).

#### Utetes caudatus

(Wesmael, 1835)

Opius caudatus Wesmael, 1835
exsertus (Thomson, 1895, *Opius*)

##### Distribution

England

##### Notes

Listed as English by Fischer and Koponen (1999), presumably following Fischer (1958), but not listed in Huddleston (1978).

#### Utetes coracinus

(Thomson, 1895)

Opius coracinus Thomson, 1895

##### Distribution

England

##### Notes

BMNH, det. Fischer, added in Fauna Europaea; there may be a literature citation for its occurrence in Britain (van Achterberg, pers. comm.) but we have been unable to trace it.

#### Utetes fulvicollis

(Thomson, 1895)

Opius fulvicollis Thomson, 1895
cupidus (Gahan, 1919, *Opius*)

##### Distribution

England

##### Notes

added by Godfray (1986)

#### Utetes rotundiventris

(Thomson, 1895)

Opius rotundiventris Thomson, 1895

##### Distribution

England

#### Utetes ruficeps

(Wesmael, 1835)

Opius ruficeps Wesmael, 1835

##### Distribution

England

#### Utetes testaceus

(Wesmael, 1838)

Opius testaceus Wesmael, 1838

##### Distribution

England

#### Utetes truncatus

(Wesmael, 1838)

Opius truncatus Wesmael, 1838

##### Distribution

England

#### Utetes zelotes

(Marshall, 1891)

Opius zelotes Marshall, 1891
insertus (Fischer, 1971, *Opius*)
incertus misspelling

##### Distribution

England, Scotland

### Orgilinae

#### Orgilinae

Ashmead, 1900

#### Orgilus

Haliday, 1833

ISCHIUS Wesmael, 1837
MACROPALPUS Ratzeburg, 1844
ORESIMUS Ashmead, 1900
ORGILOMORPHA Ashmead, 1900
ISCHIOLUS Hellén, 1958

##### Notes

Taxonomic and much distribution data from Taeger (1989).

#### Orgilus achterbergi

Taeger, 1989

##### Distribution

England

##### Notes

added by Taeger (1989)

#### Orgilus dovnari

Tobias, 1986

ukrainicus Tobias, 1986

##### Distribution

England

##### Notes

NMS, det. Taeger, added here

#### Orgilus interjectus

Taeger, 1989

##### Distribution

England

##### Notes

added by Taeger (1989)

#### Orgilus ischnus

Marshall, 1898

subtilirugosus Papp, 1971

##### Distribution

England, Wales

#### Orgilus leptocephalus

Hartig, 1838

obscurator misident.
rugulosus Fahringer, 1937
hyperboreus Hellén, 1958

##### Distribution

Ireland

##### Notes

Although Taeger (1989) clarified the identity of *obscurator* auctt. as *leptocephalus*, this species was recorded as *obscurator* by O'Connor et al. (1999).

#### Orgilus minor

Taeger, 1989

##### Distribution

England

##### Notes

NMS, det. Taeger, added here

#### Orgilus parvipennis

Thomson, 1895

micropterus Morley, 1907
macroptera (Rudow, 1917, *Aptesis*)
decoratus Hellén, 1946
curtipennis Fischer, 1958
discolor Hellén, 1958

##### Distribution

England, Scotland

#### Orgilus pimpinellae

Niezabitowski, 1910

laevigator misident.

##### Distribution

England, Scotland, Ireland

##### Notes

Added by Taeger (1989); apparently recorded as Irish under the name *Microdus laevigator* Nees, 1812 (O'Connor et al. 1999), which is listed as a species inquirendae by Taeger (1989).

#### Orgilus punctulator

(Nees, 1811)

Microdus punctulator Nees, 1811
rufiventris Fahringer, 1937

##### Distribution

England

##### Notes

added by Taeger (1989)

#### Orgilus rugosus

(Nees, 1834)

Microgaster rugosus Nees, 1834

##### Distribution

England

##### Notes

NMS, det. Taeger, added here

#### Orgilus tobiasi

Taeger, 1989

##### Distribution

Wales, Ireland

##### Notes

added by Taeger (1989)

### Pambolinae

#### Pambolinae

Marshall, 1885

#### Chremylini

Hellén, 1957

#### Chremylus

Haliday, 1833

PENECERUS Wesmael, 1838
PARAMESOCRINA Nagamori, 1925

#### Chremylus elaphus

Haliday, 1833

rubiginosus (Nees, 1834, *Hormius*)
transversus (Say, 1836, *Bracon*)
nigriceps Ashmead, 1893
terminalis Ashmead, 1893
japonicus Ashmead, 1896
tineavorus (Nagamori, 1925, *Paramesocrina*)

##### Distribution

England, Ireland

#### Pambolini

Marshall, 1885

#### Dimeris

Ruthe, 1854

PARAPTESIS Magretti, 1884

#### Dimeris mira

Ruthe, 1854

melanocephala (Marshall, 1870, *Pambolus*)
flavipes (Magretti, 1884, *Paraptesis*)
aptera Marshall, 1885
inermis Fitch, 1885

##### Distribution

England

#### Pambolus

Haliday, 1836

#### Phaenodus

Förster, 1863

#### Pambolus (Phaenodus) pallipes

(Förster, 1863)

Phaenodus pallipes Förster, 1863
flavipes (Förster, 1863, *Araphis*)
pallidipes (Marshall, 1897, *Phaenodus*)
chalveri (Docavo, 1960, *Phaenodus*)

##### Distribution

England, Scotland

##### Notes

added by Belokobylskij (1986)

#### Pambolus

Haliday, 1836

ARAPHIS Ruthe, 1854
ARRHAPHIS misspelling
ARHAPHIS misspelling
FOLCHINIA Kieffer, 1906
PARAMBOLUS Dahl, 1912

##### Notes

species of Pambolus (Pambolus) excluded from the British and Irish list:

[*biglumis* (Haliday, 1836, *Rogas*); syn. *rosenhaueri* (Ratzeburg, 1852, *Pezomachus*); *dubius* (Fitch, 1885, *Araphis*); *imminens* (Fitch, 1885, *Araphis*)] Included as British in Fauna Europaea and Taxapad (Yu et al. 2012) (but not by Huddleston 1978); this seems to be on the basis of Fitch’s descriptions of the synonymous names *dubius* and *imminens* in Marshall’s (Marshall 1885) monograph of British Braconidae. However, the types of Fitch’s species are from the Ruthe collection of German material and we have seen no British or Irish specimens of *biglumis*.

### Rhysipolinae

#### Rhysipolinae

Belokobylskij, 1984

#### Pseudavga

Tobias, 1964

#### Pseudavga flavicoxa

Tobias, 1964

rustus (Papp, 1991, *Rhysipolis*)

##### Distribution

England

##### Notes

added by Shaw and Sims (2015)

#### Rhysipolis

Förster, 1863

##### Notes

Most synonymy has been omitted; published taxonomy has been confused and there are several more species present than have been recorded in the literature, with the application of names not yet settled.

#### Rhysipolis decorator

(Haliday, 1836)

Rogas decorator Haliday, 1836
ruficeps (Wesmael, 1838, *Exothecus*)
ruficornis (Szépligeti, 1896, *Xenarcha*)

##### Distribution

England, Scotland, Isle of Man

##### Notes

some distribution data from Shaw and Askew (1976)

#### Rhysipolis hariolator

(Haliday, 1836)

Rogas hariolator Haliday, 1836
barbatus (Wesmael, 1838, *Exothecus*)

##### Distribution

England, Scotland

##### Notes

some distribution data from Shaw and Askew (1976)

#### Rhysipolis meditator

(Haliday, 1836)

Rogas meditator Haliday, 1836

##### Distribution

England, Scotland

#### Rhysipolis variabilis

(Szépligeti, 1896)

Xenarcha variabilis Szépligeti, 1896

##### Distribution

England

##### Notes

NMS, det. Shaw & van Achterberg, added here; treated as a synonym of *meditator* by Belokobylskij and Tobias (1986) and Belokobylskij et al. (2003) but here treated as a valid species.

#### Rhysipolis varicoxa

(Thomson, 1892)

Exothecus varicoxa Thomson, 1892

##### Distribution

England

##### Notes

NMS, det. Shaw & van Achterberg, added here; treated as a synonym of *meditator* by Belokobylskij and Tobias (1986) and Belokobylskij et al. (2003)​ but here treated as a valid species.

### Rhyssalinae

#### Rhyssalinae

Förster, 1863

HISTEROMERINAE Fahringer, 1930

#### Acrisidini

Hellén, 1957

#### Acrisis

Förster, 1863

EUCHASMUS Marshall, 1888
EPISIGALPHUS Ashmead, 1900

#### Acrisis exiguus

(Marshall, 1888)

Euchasmus exiguus Marshall, 1888

##### Distribution

England

#### Proacrisis

Tobias, 1983

##### Notes

Treated as a synonym of *Acrisis* in Fauna Europaea, as a separate genus by Belokobylskij et al. (2003).

#### Proacrisis acutus

Tobias, 1983

##### Distribution

England

##### Notes

NMS, det. Shaw & van Achterberg, added here

#### Proacrisis rarus

Tobias, 1983

##### Distribution

England

##### Notes

NMS, det. Shaw & van Achterberg, added on Fauna Europaea

#### Histeromerini

Fahringer, 1930

##### Notes

Zaldívar-Riverón et al. (2006) and Sharanowski et al. (2011) both found, on the basis of molecular phylogenetic results, that the morphologically and biologically aberrant genus *Histeromerus* belongs in Rhyssalinae; however, there is as yet no indication as to how *Histeromerus* can be accommodated within the existing tribal classification of Rhyssalinae, so we simply use Histeromerini for now.

#### Histeromerus

Wesmael, 1838

MITHOTYNIA Hedqvist, 1976

#### Histeromerus mystacinus

Wesmael, 1838

apterus (Hedqvist, 1976, *Mithotynia*)

##### Distribution

England, Wales, Ireland

##### Notes

distribution data from Shaw (1995) and the Horniman Museum

#### Rhyssalini

Förster, 1863

#### Dolopsidea

Hincks, 1944

DOLOPS Marshall, 1889 preocc.
EXONTSIRA Belokobylskij, 1982

#### Dolopsidea indagator

(Haliday, 1836)

Rogas indagator Haliday, 1836
tuberculata (Wesmael, 1838, *Exothecus*)
aculeator (Marshall, 1889, *Dolops*)
hastifer (Marshall, 1889, *Dolops*)
caucasica (Tobias, 1976, *Doryctodes*)
rhodopea (Zaykov, 1980, *Rhyssalus*)

##### Distribution

England, Scotland, Ireland

##### Notes

some distribution data from Shaw (1993)

#### Oncophanes

Förster, 1863

EPIRHYSSALUS Ashmead, 1900

#### Oncophanes minutus

(Wesmael, 1838)

Exothecus minutus Wesmael, 1838
lanceolator (Nees, 1834, *Bracon*) preocc.
laevigatus (Ratzeburg, 1852, *Bracon*)

##### Distribution

England, Wales, Scotland

##### Notes

Traditionally treated as a species separate from *minutus* (e.g. Huddleston 1978; Fauna Europaea), *laevigatus* was synonymised by Belokobylskij (1998); we follow this, although it seems likely that there are two species involved.

#### Pseudobathystomus

Belokobylskij, 1986

#### Pseudobathystomus funestus

(Haliday, 1836)

Rogas funestus Haliday, 1836
schmiedeknechti (Fahringer, 1930, *Bathystomus*)

##### Distribution

England, Scotland

##### Notes

Listed as a species of *Rhysipolis* in Huddleston (1978).

#### Pseudobathystomus tobiasi

(Zaykov, 1980)

Oncophanes tobiasi Zaykov, 1980

##### Distribution

England, Scotland

##### Notes

NMS, det. Shaw & van Achterberg, added on Fauna Europaea

#### Pseudobathystomus vernalis

Belokobylskij, 1994

##### Distribution

England, Scotland

##### Notes

NMS, det. Shaw & van Achterberg, added on Fauna Europaea

#### Rhyssalus

Haliday, 1833

EURHOPTROCENTRUS Tobias,1977

#### Rhyssalus clavator

Haliday, 1833

##### Distribution

England, Scotland, Ireland, Isle of Man

#### Rhyssalus longicaudis

(Tobias & Belokobylskij, 1991)

Eurhoptrocentrus longicaudis Tobias & Belokobylskij, 1991

##### Distribution

Wales

##### Notes

NMS, det. Shaw & van Achterberg, added on Fauna Europaea

### Rogadinae

#### Rogadinae

Förster, 1863

##### Notes

Distribution data mostly from NMS and BMNH.

#### Aleiodini

Muesebeck, 1928

##### Notes

Resurrected by Zaldívar-Riverón et al. (2008b).

#### Aleiodes

Wesmael, 1838

ROGAS misident.
RHOGAS misident.
PETALODES Wesmael, 1838
NEORHOGAS Szépligeti, 1906
CHELONORHOGAS Enderlein, 1912

##### Notes

*Aleiodes* species are further subdivided in Taxapad (Yu et al. 2012) into the subgenera *Aleiodes*, *Chelonorhogas*, *Neorhogas* and *Heterogamus* (for those occurring in Britain); the latter is considered here to be a distinct genus (see note under *Heterogamus*).

species of *Aleiodes* excluded from the British and Irish list:

[*arcticus* (Thomson, 1892, *Rogas*)] MRS has seen the specimens that this record is based upon (in Ipswich Museum) and they are not *arcticus*.

#### Aleiodes albitibia

(Herrich-Schäffer, 1838)

Rogas albitibia Herrich-Schäffer, 1838
heterogaster Wesmael, 1838

##### Distribution

England, Scotland, Wales, Ireland

#### Aleiodes alternator

(Nees, 1834)

Rogas alternator Nees, 1834
geniculator misident.
balteatus (Curtis, 1834, *Rogas*)

##### Distribution

England, Scotland, Wales, Ireland

#### Aleiodes apicalis

(Brullé, 1832)

Bracon apicalis Brullé, 1832
ductor misident., in part
reticulator (Nees, 1834, *Rogas*)

##### Distribution

England

##### Notes

to be added by van Achterberg & Shaw (in prep.); just one British specimen of this Mediterranean species, considered to be an erratic. Synonymy anticipates van Achterberg & Shaw (in prep.).

#### Aleiodes apiculatus

(Fahringer, 1932)

Rhogas apiculatus Fahringer, 1932

##### Distribution

England

##### Notes

to be added by van Achterberg & Shaw (in prep.)

#### Aleiodes assimilis

(Nees, 1812)

Bracon assimilis Nees, 1812
bicolor misident., in part
zygaenae (Nees, 1834, *Rogas*)

##### Distribution

England, Scotland, Ireland

##### Notes

added by Schwarz and Shaw (2000)

#### Aleiodes aterrimus

(Ratzeburg, 1852)

Bracon aterrimus Ratzeburg, 1852
grandis Giraud, 1857

##### Distribution

England

#### Aleiodes bicolor

(Spinola, 1808)

Bracon bicolor Spinola, 1808

##### Distribution

England, Scotland, Wales, Ireland

#### Aleiodes cantherius

(Lyle, 1919)

Rhogas cantherius Lyle, 1919

##### Distribution

England

#### Aleiodes circumscriptus

(Nees, 1834)

Rogas circumscriptus Nees, 1834

##### Distribution

England, Scotland, Wales, Ireland

##### Notes

Sensu neotype (to be published by van Achterberg & Shaw, in prep.).

#### Aleiodes compressor

(Herrich-Schäffer, 1838)

Rogas compressor Herrich-Schäffer, 1838
unicolor (Wesmael, 1838, *Petalodes*) preocc.

##### Distribution

England, Scotland, Wales

#### Aleiodes coxalis

(Spinola, 1808)

Rogas coxalis Spinola, 1808
ater (Curtis, 1834, *Rogas*)
tristis Wesmael, 1838

##### Distribution

England, Scotland, Isle of Man

##### Notes

Anticipates synonymy to be published by van Achterberg & Shaw (in prep.).

#### Aleiodes crassipes

(Thomson, 1892)

Rogas crassipes Thomson, 1892

##### Distribution

Wales

##### Notes

to be added by van Achterberg & Shaw (in prep.)

#### Aleiodes cruentus

(Nees, 1834)

Rogas cruentus Nees, 1834

##### Distribution

England

#### Aleiodes dissector

(Nees, 1834)

Rogas dissector Nees, 1834
aestivalis (Vollenhoven, 1858, *Phylax*)

##### Distribution

England, Scotland

#### Aleiodes fortipes

(Reinhard, 1863)

Rogas fortipes Reinhard, 1863
freyi (Hellén, 1927, *Rhogas*)

##### Distribution

England

##### Notes

to be added by van Achterberg & Shaw (in prep.)

#### Aleiodes gastritor

(Thunberg, 1824)

Ichneumon gastritor Thunberg, 1824
circumscriptus misident., in part
testaceus misident., in part

##### Notes

Distribution data are not given as this name covers an aggregate of at least four species in Britain (van Achterberg & Shaw, in prep.). Further, it is not clear to which the name *gastritor* should be applied. *Bracon testaceus* Spinola, 1808 is actually *Rogas luteus*, and ‘*Aleiodes testaceus* (Spinola)’ is not a valid taxon, although it has been applied to several small, orangeish Aleiodes, epecially in the *gastritor* aggregate.

#### Aleiodes grassator

(Thunberg, 1824)

Ichneumon grassator Thunberg, 1824
carbonarius Giraud, 1857
flavipalpis (Thomson, 1892, *Rogas*)

##### Distribution

England, Scotland

##### Notes

Anticipates synonymy to be published by van Achterberg & Shaw (in prep.).

#### Aleiodes heterostigma

(Stelfox, 1953)

Rogas heterostigma Stelfox, 1953

##### Distribution

Wales, Ireland

#### Aleiodes hirtus

(Thomson, 1892)

Rogas hirtus Thomson, 1892

##### Distribution

England, Scotland

##### Notes

To be added by van Achterberg & Shaw (in prep.); treated as a synonym of *pallidicornis* by Belokobylskij et al. (2003), following Papp (1985).

#### Aleiodes modestus

(Reinhard, 1863)

Rogas modestus Reinhard, 1863
piceus (Fahringer, 1932, *Rhogas*)

##### Distribution

England, Scotland, Wales

#### Aleiodes nigriceps

Wesmael, 1838

##### Distribution

England, Scotland, Wales, Ireland

##### Notes

to be added by van Achterberg & Shaw (in prep.); listed as a subspecies of circumscriptus in Taxapad (Yu et al., 2012), following Papp (1999c) and other authors.

#### Aleiodes nigricornis

Wesmael, 1838

##### Distribution

England, Scotland, Wales, Ireland

#### Aleiodes nobilis

(Haliday, 1834)

Rogas nobilis Haliday, 1834
ductor misident., in part
medianus (Thomson, 1892, *Rogas*)

##### Distribution

England, Scotland, Ireland

##### Notes

Brought out of synonymy with *ductor* by Achterberg (1997).

#### Aleiodes pallidator

(Thunberg, 1824)

Ichneumon pallidator Thunberg, 1824
ochraceus (Curtis, 1834, *Rogas*)
unicolor Wesmael, 1838
Aleiodes pallidator ?*pellucens* (Telenga, 1941, *Rhogas*)

##### Distribution

England

##### Notes

some distribution data from Shaw (1977), Shaw (1981)

#### Aleiodes pallidicornis

(Herrich-Schäffer, 1838)

Rogas pallidicornis Herrich-Schäffer, 1838
ductor misident., in part

##### Distribution

Scotland

##### Notes

to be added by van Achterberg & Shaw (in prep.); specimen in BMNH (seen by MRS).

#### Aleiodes pictus

(Herrich-Schäffer, 1838)

Rogas pictus Herrich-Schäffer, 1838
borealis misident., in part
circumscriptus misident., in part
nigriceps misident., in part

##### Distribution

England, Scotland, Wales, Ireland

##### Notes

to be added by van Achterberg & Shaw (in prep.)

#### Aleiodes praetor

(Reinhard, 1863)

Rogas praetor Reinhard, 1863
luteus (Szépligeti, 1906, *Neorhogas*)

##### Distribution

England

#### Aleiodes pulchripes

Wesmael, 1838

##### Distribution

England, Ireland, Isle of Man

##### Notes

some distribution data from Shaw (1979)

#### Aleiodes punctipes

(Thomson, 1892)

Rogas punctipes Thomson, 1892

##### Distribution

Scotland, Wales, Ireland

#### Aleiodes ruficornis

(Herrich-Schäffer, 1838)

Rogas ruficornis Herrich-Schäffer, 1838
dimidiatus misident.
gasterator misident.

##### Distribution

England, Ireland

#### Aleiodes rugulosus

(Nees, 1811)

Bracon rugulosus Nees, 1811
pictus (Kokujev, 1898, *Rhogas*) preocc.

##### Distribution

England, Scotland, Wales, Ireland

#### Aleiodes seriatus

(Herrich-Schäffer, 1838)

Rogas seriatus Herrich-Schäffer, 1838
vittiger Wesmael, 1838
kuslitzkyi (Tobias, 1976, *Rogas*)

##### Distribution

England, Ireland

#### Aleiodes signatus

(Nees, 1812)

Bracon signatus Nees, 1812
geniculator (Nees, 1834, *Rogas*)
annulipes (Herrich-Schäffer, 1838, *Rogas*)
esseni Hellén, 1927

##### Distribution

England, Wales

#### Aleiodes similis

(Curtis, 1834)

Rogas similis Curtis, 1834
circumscriptus misident., in part
testaceus misident., in part
spathuliformis (Curtis, 1834, *Rogas*)
subucola (Curtis, 1834, *Rogas*)
armatus Wesmael, 1838

##### Distribution

England, Scotland, Ireland

##### Notes

The name *armatus* has been misapplied to a range of undescribed, medium-sized, orange species, at least three of which occur in Britain and Ireland and will be described by van Achterberg & Shaw (in prep.).

#### Aleiodes testaceus

(Telenga, 1941)

Heterogamus testaceus Telenga, 1941

##### Distribution

England, Wales

##### Notes

to be added by van Achterberg & Shaw (in prep.)

#### Aleiodes ungularis

(Thomson, 1892)

Rogas ungularis Thomson, 1892

##### Distribution

England, Wales, Ireland

##### Notes

To be added by van Achterberg & Shaw (in prep.); English and Welsh records from specimens seen by MRS in, respectively, the Hope Department, Oxford, and Doncaster Museum.

#### Aleiodes unipunctator

(Thunberg, 1824)

Ichneumon unipunctator Thunberg, 1824
ductor (Thunberg, 1824, *Ichneumon*)
irregularis Wesmael, 1838

##### Distribution

England, Scotland, Wales, Ireland

##### Notes

Anticipates synonymy to be published by van Achterberg & Shaw (in prep.).

#### Aleiodes sp. L

van Achterberg & Shaw, in prep.

borealis misident.
circumscriptus misident., in part
nigriceps misident., in part

##### Distribution

England, Scotland, Wales, Ireland, Isle of Man

##### Notes

To be added by van Achterberg & Shaw (in prep.); this species, whose name is not yet published (van Achterberg & Shaw, in prep.), is very common in the British Isles and M.R. Shaw det. labels (as *borealis*) have been left in several British collections (up to 2007). The true *A. borealis* (Thomson, 1892, *Rogas*) has not been found in the British Isles.

#### Heterogamus

Wesmael, 1838

##### Notes

Usually treated as a subgenus of *Aleiodes* (as in Taxapad: Yu et al. 2012) but Zaldivar-Riverón et al. (Zaldívar-Riverón et al. 2004, Zaldívar-Riverón et al. 2008b) have shown that, on the basis of venom apparatus characters and molecular sequence data, respectively, *Heterogamus* species form a clade distinct from *Aleiodes* species. Murray (1939) and Shaw (2000a) published some distribution records.

#### Heterogamus dispar

(Haliday, 1833)

Rogas dispar Haliday, 1833
dispar (Curtis, 1834, *Rogas*) preocc.
crypticornis (Wesmael, 1838, *Aleiodes*)

##### Distribution

England, Scotland, Wales, Ireland

#### Heterogamus excavatus

Telenga, 1941

farmakena Maláč, 1941

##### Distribution

England

##### Notes

Added by Shaw (2000a); Irish records (O'Connor et al. 1999) are erroneous (Shaw 2000a).

#### Clinocentrini

van Achterberg, 1991

#### Clinocentrus

Haliday, 1833

CAMPTOCENTRUS Kriechbaumer, 1894
MICRORHOGAS Cameron, 1910
NEORHYSSALUS Baker, 1917

##### Notes

Nomenclature and distribution data from Belokobylskij (1995) and NMS.

species of *Clinocentrus* excluded from the British and Irish list:

[*tenuicornis* (Thomson, 1891, *Exothecus*)] A species of uncertain status (Belokobylskij, pers. comm. to MRS), listed as a synonym of *excubitor* in Taxapad (Yu et al. 2012), following Szépligeti (1906).

#### Clinocentrus brevicalcar

(Thomson, 1891)

Exothecus brevicalcar Thomson, 1891

##### Distribution

England, Scotland

#### Clinocentrus cunctator

(Haliday, 1836)

Rogas cunctator Haliday, 1836
analis (Wesmael, 1838, *Exothecus*)
gracilipes (Thomson, 1892, *Exothecus*)

##### Distribution

England, Scotland, Wales, Ireland

#### Clinocentrus excubitor

(Haliday, 1836)

Rogas excubitor Haliday, 1836
marginellus (Wesmael, 1838, *Exothecus*)

##### Distribution

England, Scotland, Wales, Ireland

#### Clinocentrus exsertor

(Nees, 1811)

Bracon exsertor Nees, 1811
orbitator (Nees, 1834, *Bracon*)
striolatus (Thomson, 1891, *Exothecus*)
tarsalis Ashmead, 1894

##### Distribution

England, Scotland

#### Clinocentrus hungaricus

Szépligeti, 1906

##### Distribution

England, Scotland

##### Notes

NMS, BMNH, det. Shaw, added here

#### Clinocentrus umbratilis

Haliday, 1833

petiolaris (Thomson, 1891, *Exothecus*)
polonicus Fahringer, 1931

##### Distribution

England, Scotland, Ireland

#### Clinocentrus vestigator

(Haliday, 1836)

Rogas vestigator Haliday, 1836
stigmaticus Marshall, 1897
jaroshevskyi Telenga, 1941
obsoletus (Hellén, 1957, *Oncophanes*)

##### Distribution

England, Scotland, Ireland, Isle of Man

#### Rogadini

Förster, 1863

PELECYSTOMINI Viereck, 1918

#### Rogas

Nees, 1818

PELECYSTOMA Wesmael, 1838
RHOGAS Agassiz, 1846

#### Rogas luteus

Nees, 1834

testaceus (Fabricius, 1798, *Ichneumon*) preocc.
testaceator (Thunberg, 1824, *Ichneumon*)

#### Triraphis

Ruthe, 1855

##### Notes

species of *Triraphis* excluded from the British and Irish list:

[*tricolor* (Wesmael, 1838, *Pelecystoma*); syn. *solitarius* (Watanabe, 1970, *Pelecystoma*); synonymy by Papp (1995a)] Listed by Huddleston (1978), probably on the basis of the host-parasitoid catalogue of Morley and Rait-Smith (1933), but this publication included non-British rearings from Lepidoptera that occur in Britain and there is no evidence that *tricolor*, a parasitoid of *Apoda limacodes* (Hufnagel) (Lepidoptera: Limacodidae), has ever been found in Britain or Ireland.

### Sigalphinae

#### Sigalphinae

Haliday, 1833

#### Acampsini

van Achterberg & Austin, 1992

#### Acampsis

Wesmael, 1835

#### Acampsis alternipes

(Nees, 1816)

Sigalphus alternipes Nees, 1816

##### Distribution

England

#### Sigalphini

Haliday, 1833

#### Sigalphus

Latreille, 1802

SPHAEROPYX Illiger, 1807
RHITIGASTER Wesmael, 1835
RHYTIDOGASTER Agassiz, 1846

##### Notes

species of *Sigalphus* excluded from the British and Irish list:

[*irrorator* (Fabricius, 1775, *Ichneumon*); syn. *niger* (Retzius, 1783, *Ichneumon*); syn. *globulifer* (Geoffroy, 1785, *Ichneumon*); *irroratrix* (Schulz, 1906, *Sphaeropyx*)] Shaw and Huddleston (1991) were unable to trace any British specimens.

## Supplementary Material

Supplementary material 1 Checklist of British and Irish Hymenoptera Data type: formatted text file
Brief description: Word document version of the checklist
File: oo_84392.docx Broad, G.R., Shaw, M.R. & Godfray, H.C.J.

Supplementary material 2 Checklist of British and Irish Braconidae Data type: spreadsheet
Brief description: Excel spreadsheet version of the checklist
File: oo_84393.xlsx Broad, G.R., Shaw, MR. & Godfray, H.C.J.

## Figures and Tables

**Figure 1a.**
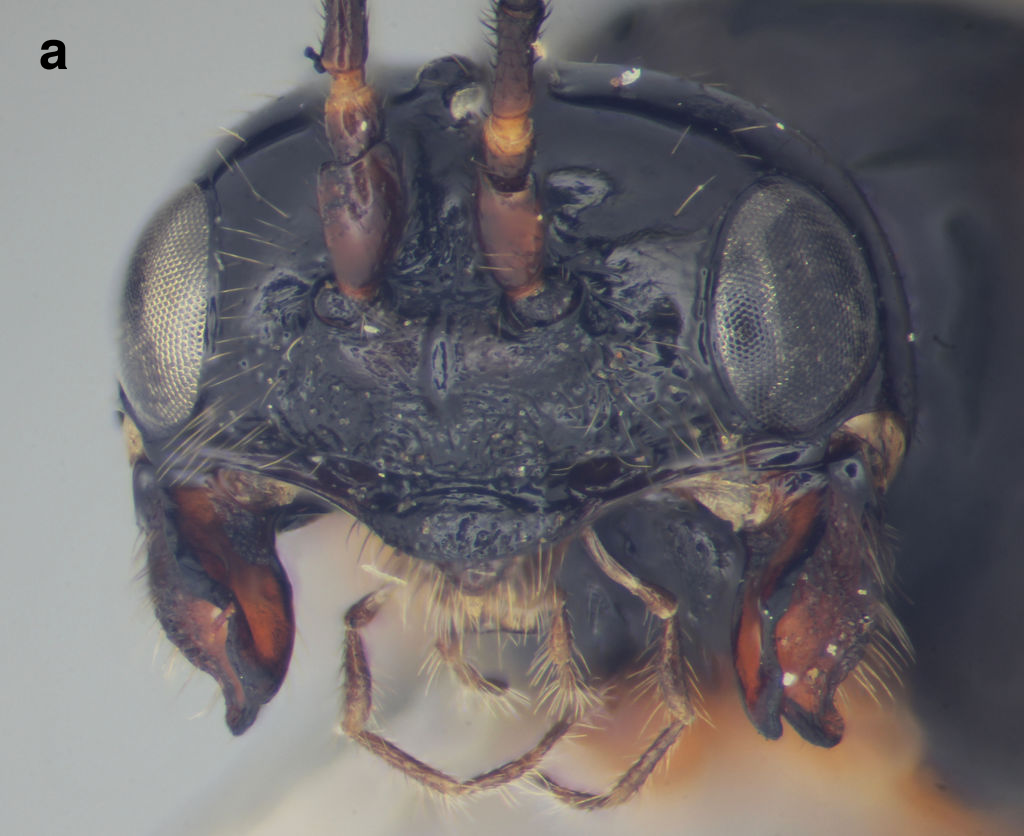
Face of *Alsysia manducator* (Panzer) (Alysiinae), with exodont mandibles for emerging from Diptera puparia. BMNH specimen.

**Figure 1b.**
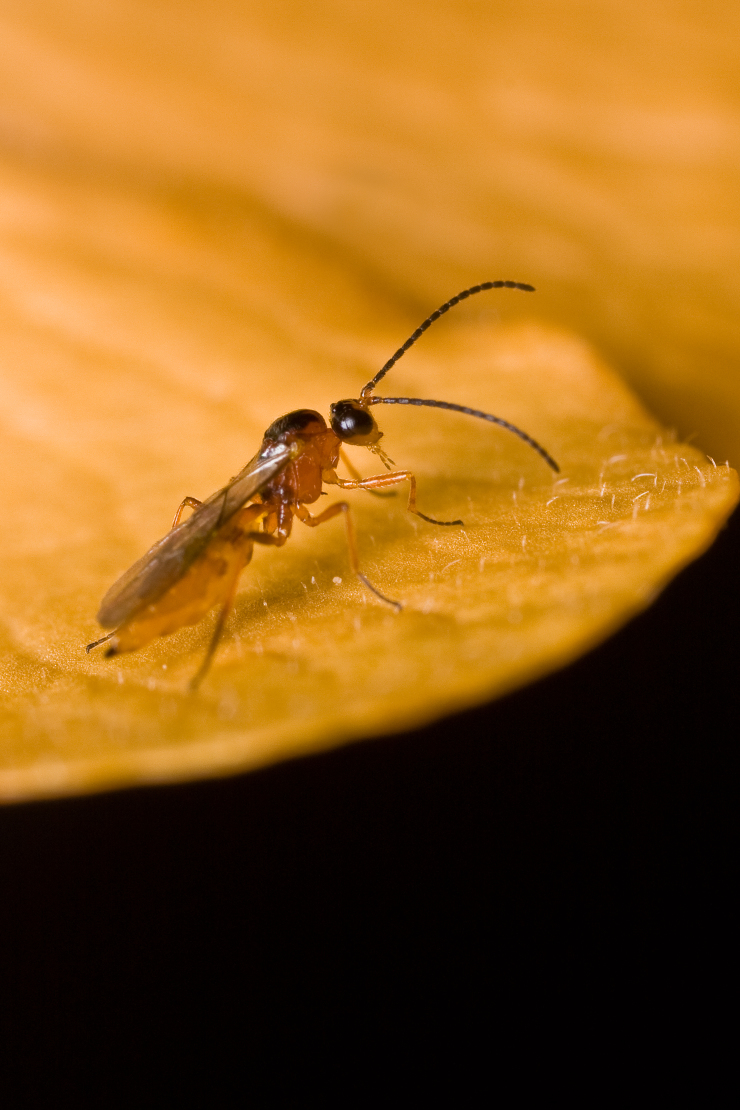
*Pauesia* sp. (Aphidiinae) (courtesy of P. Adams)

**Figure 1c.**
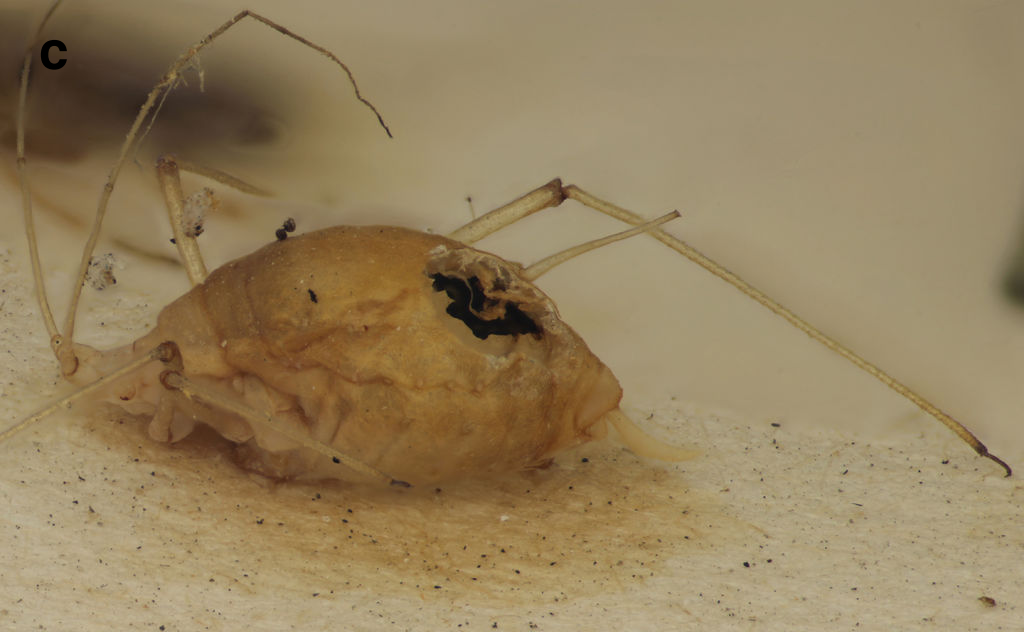
*Aphidius ervi* (Haliday) (Aphidiinae): mummified aphid host that the braconid pupates within. BMNH specimen.

**Figure 1d.**
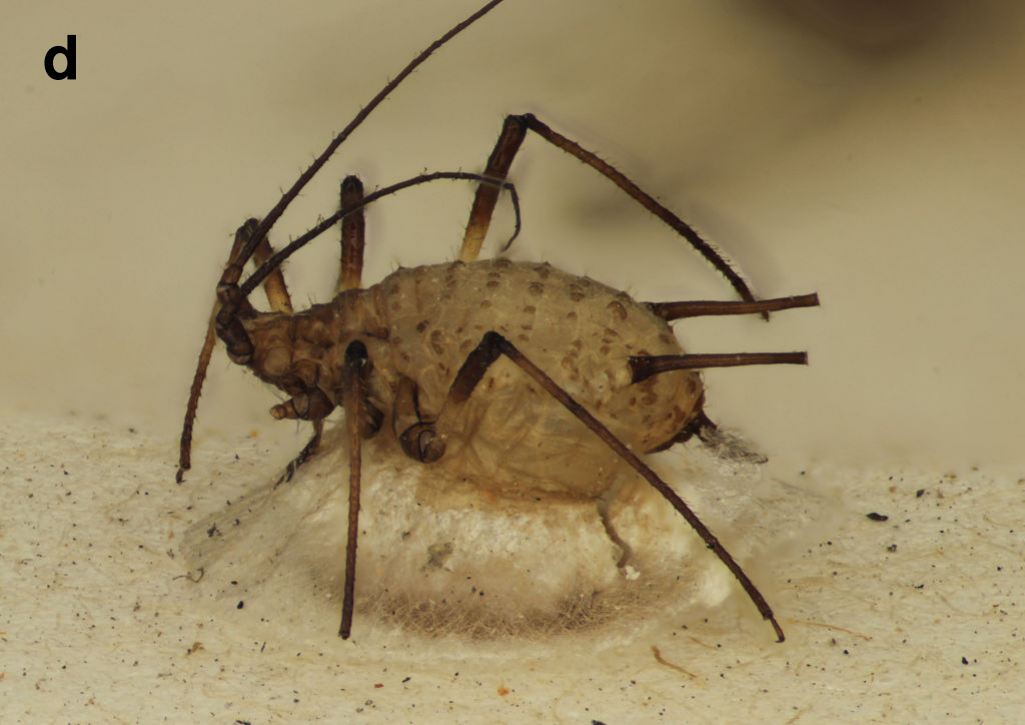
*Praon volucre* (Haliday) (Aphidiinae): mummified aphid host with the braconid pupa beneath. BMNH specimen.

**Figure 2a.**
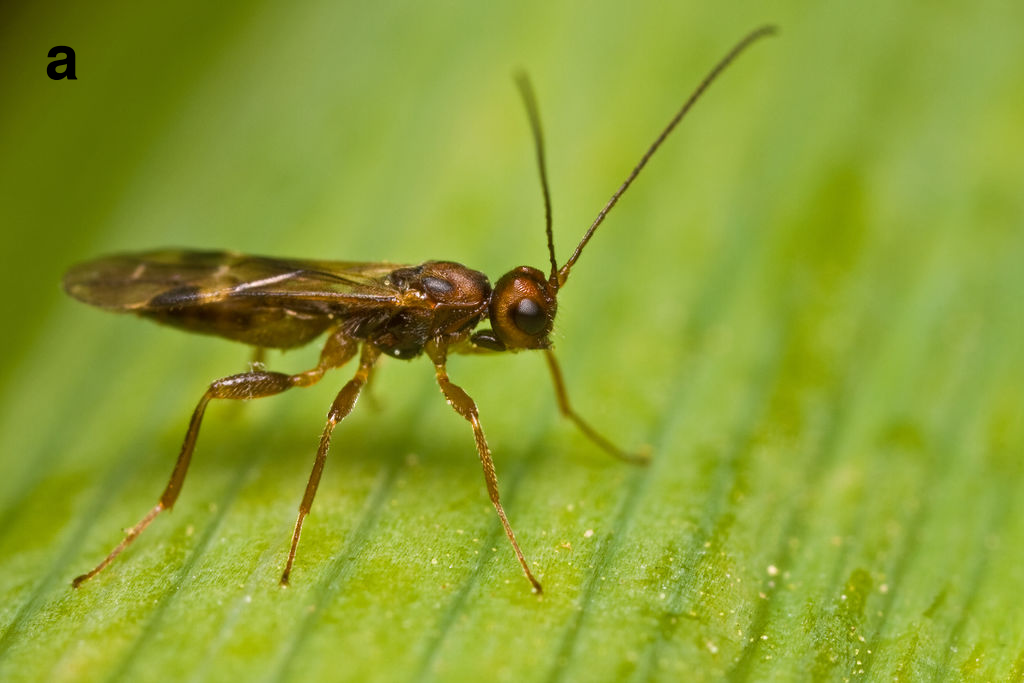
*Dendrosoter protuberans* (Nees) (Doryctinae) male (courtesy of P. Adams)

**Figure 2b.**
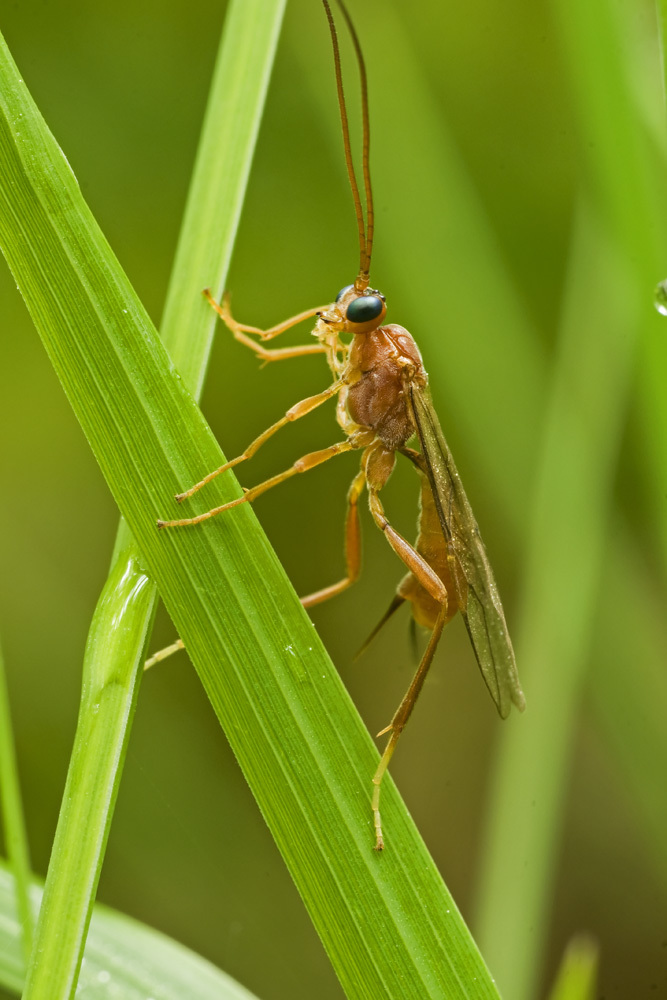
*Zele albiditarsus* Curtis (Meteorinae) female (courtesy of E. Brosens)

**Figure 2c.**
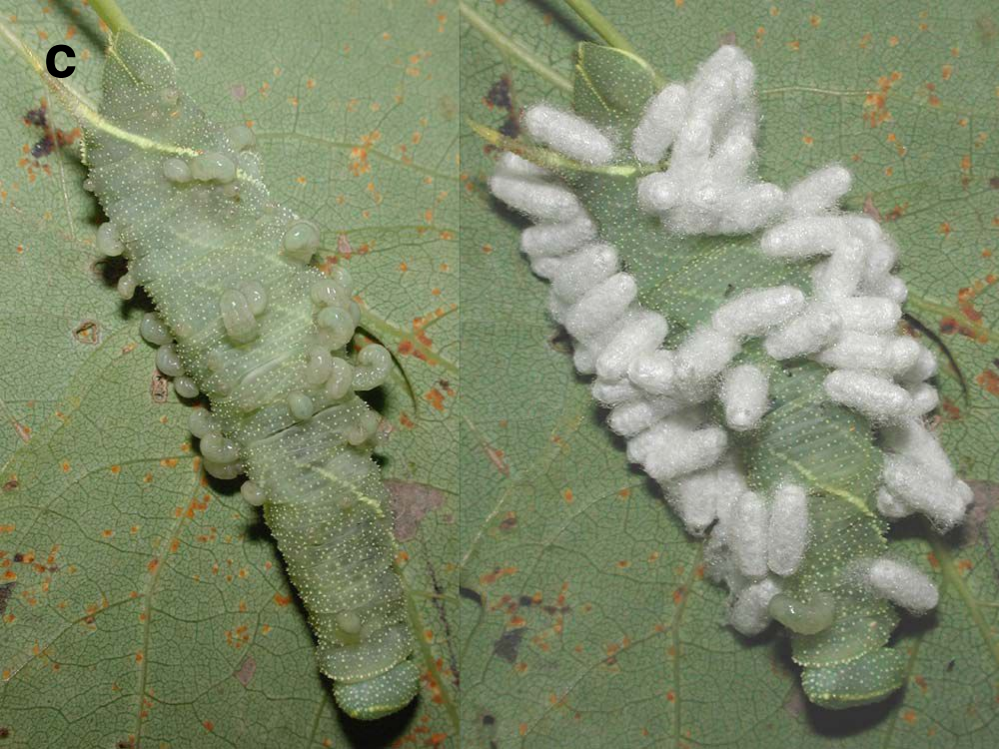
*Cotesia affinis* (Nees) (Microgastrinae) larvae emerging from their *Laothoe populi* (L.) (Lepidoptera: Sphingidae) host and then spinning cocoons (courtesy of M. Boddington)

**Figure 2d.**
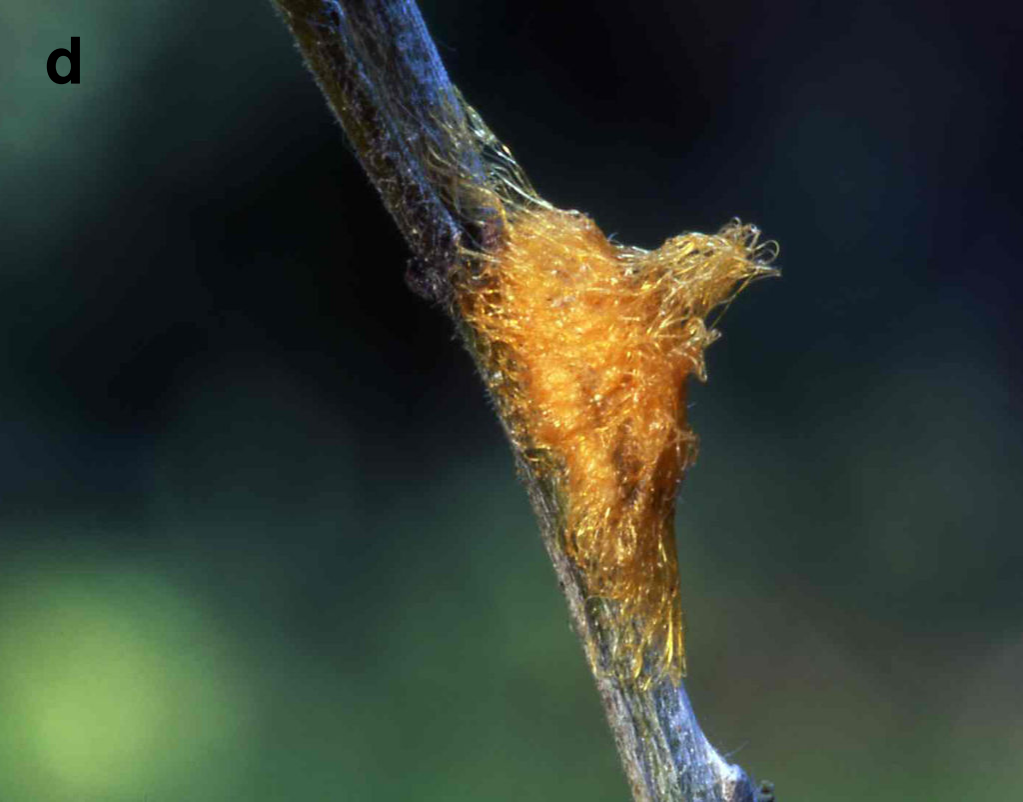
*Cotesia gonopterygis* (Marshall) (Microgastrinae) overwintering cocoon mass ex *Gonepteryx rhamni* (L.) (Lepidoptera: Pieridae) (M.R. Shaw)

**Figure 3a.**
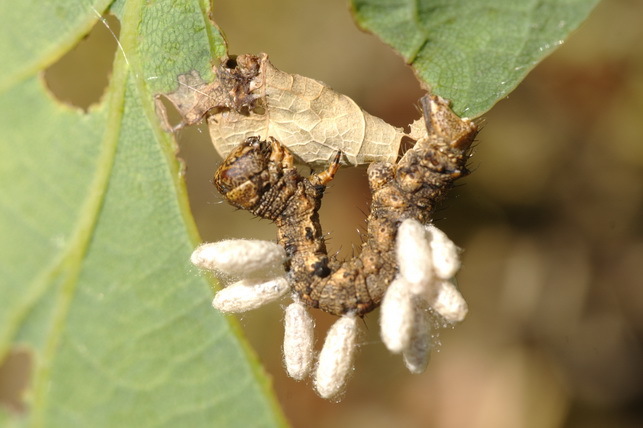
*Cotesia spuria* (Wesmael) (Microgastrinae) cocoons ex larval host *Apocheima hispidaria* (D. & S.) (Lepidoptera: Geometridae) (courtesy of J. Voogd)

**Figure 3b.**
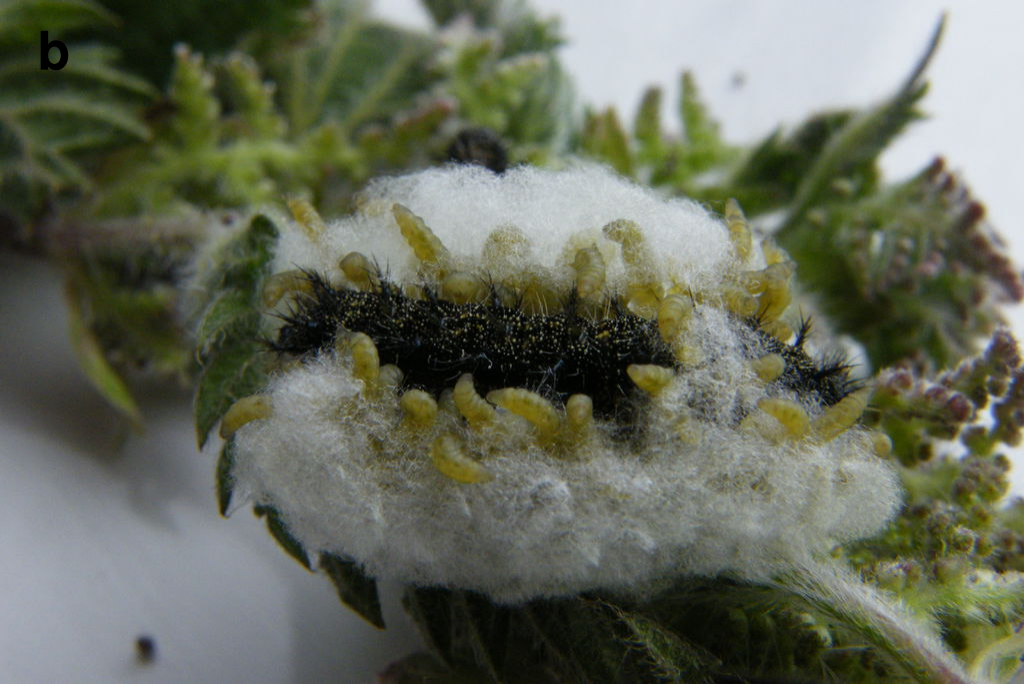
*Cotesia vanessae* (Reinhard) (Microgastrinae) emerging from larval host *Aglais urticae* (Linnaeus) (Lepidoptera: Nymphalidae) (courtesy of N. Spring)

**Figure 3c.**
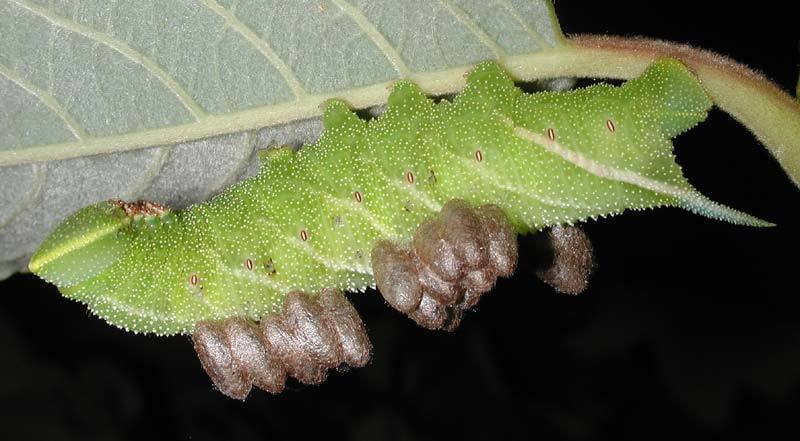
*Microplitis ocellatae* (Bouché) (Microgastrinae) cocoons ex larval host *Smerinthus ocellatus* (Linnaeus) (Lepidoptera: Sphingidae) (courtesy of M. Boddington)

**Figure 3d.**
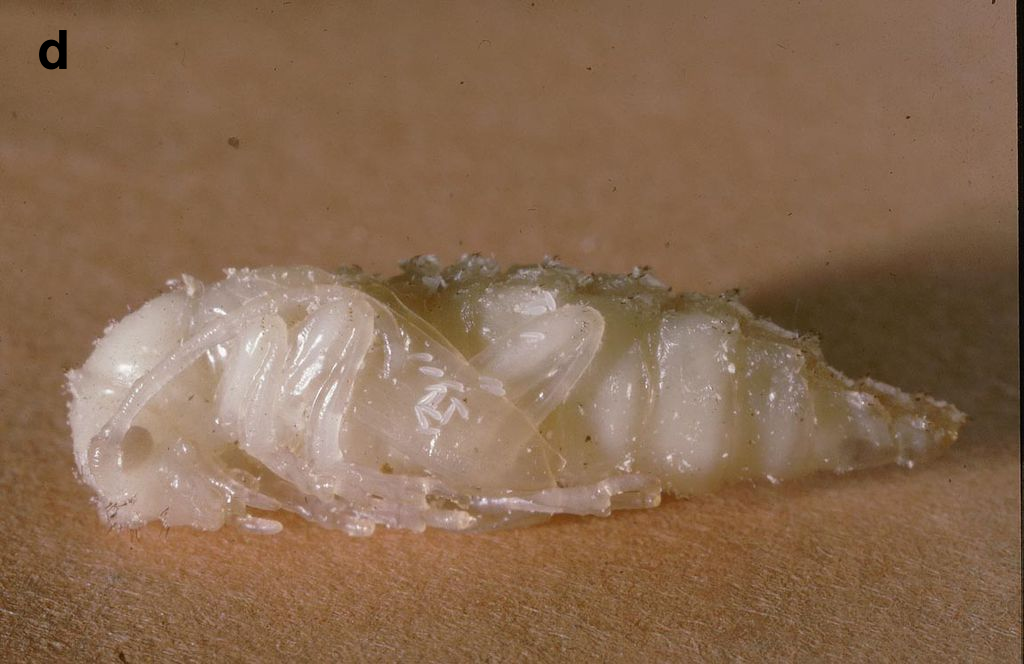
*Histeromerus mystacinus* Wesmael (Rhyssalinae) eggs on pupal host *Stictoleptura scutellata* (L.) (Coleoptera: Cerambycidae) (M.R. Shaw)

**Figure 4a.**
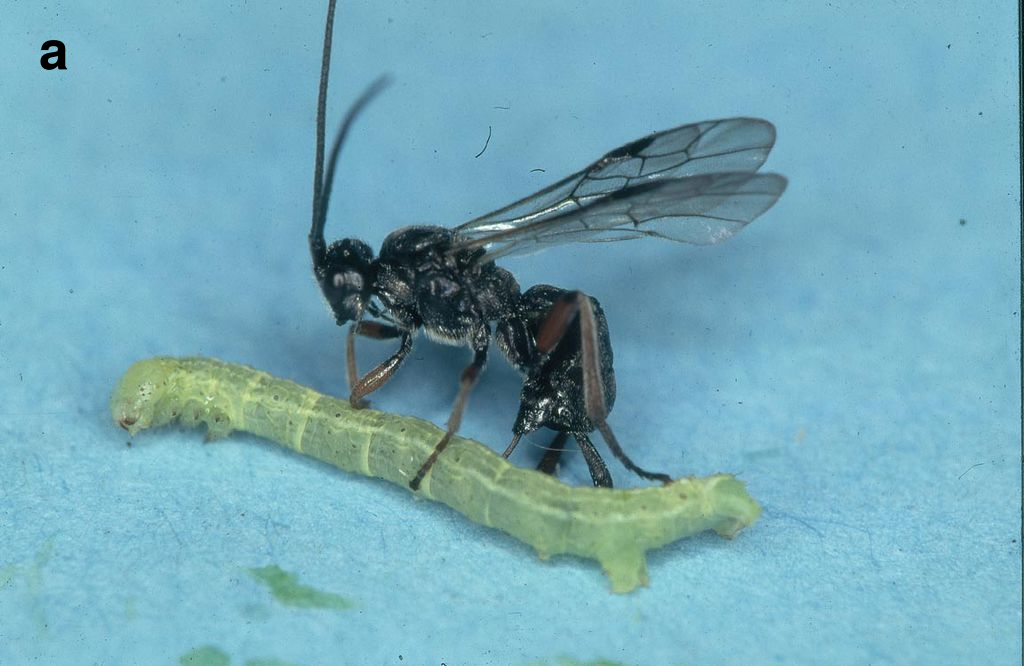
*Acampsis alternipes* (Nees) ovipositing in larval host, *Alsophila aescularia* (D. & S.) (Lepidoptera: Geometridae) (M.R. Shaw)

**Figure 4b.**
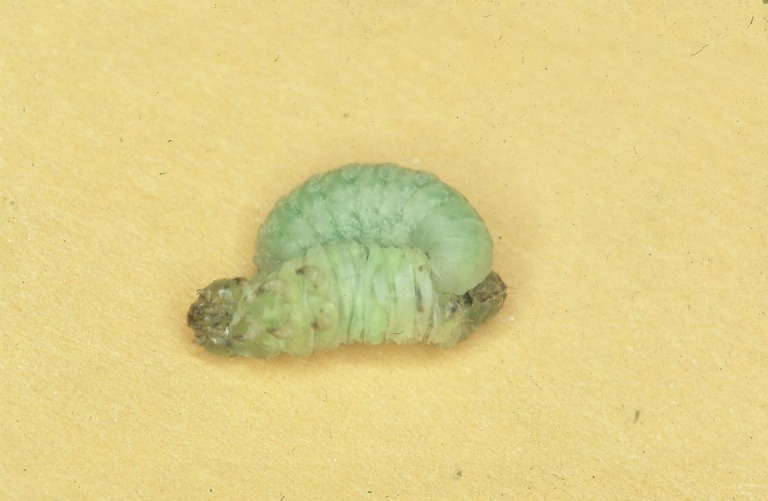
*Acampsis alternipes* (Nees) larva emerging from its larval host *Alsophila aescularia* (D. & S.) (Lepidoptera: Geometridae) to complete its feeding externally (M.R. Shaw)
