# Supplementary material for: Checklist of British and Irish Hymenoptera - Braconidae
Source: Biodivers Data J. 2016 Apr 21;(4):e8151. doi: 10.3897/BDJ.4.e8151 (PMC4867695; doi:10.3897/BDJ.4.e8151)
Supplement: Supplementary material 1 — Checklist of British and Irish Hymenoptera [file biodiversity_data_journal-4-e8151-s001.docx]

Superfamily **ICHNEUMONOIDEA**

Family **Braconidae** Nees, 1811

Gavin R. Broad, Mark R. Shaw & H. Charles J. Godfray

The bases for the Braconidae checklist are Fauna Europaea (http://www.faunaeur.org/) and Taxapad (Yu et al., 2012) (braconid data for both compiled by Kees van Achterberg). Much of the synonymy adopted here is equivalent to the German list (Belokobylskij et al., 2003), but where there are taxonomic differences of opinion, usually van Achterberg’s interpretation has been followed, largely for consistency with Fauna Europaea, but also because of van Achterberg’s experience with many groups of braconids. The braconid section of the 1978 British checklist (Huddleston, 1978) repeats many of Shenefelt’s (e.g. 1973, 1974) mistakes. Authorship: the sections on subfamilies Microgastrinae and Rogadinae are mainly the work of MRS, the section on Dacnusini (Alysiinae) is mainly the work of HCJG, who also contributed to the Alysiini and Opiinae sections. Most of the distribution data derive from specimens held in NMS and BMNH.

Subfamily AGATHIDINAE Haliday, 1833^^[[1]](#footnote-2)^^

Tribe Agathidini Nees, 1814

BASSINI Nees, 1812 invalid

EUMICRODINI Förster, 1863

BASSINI Förster, 1869 preocc.

MICRODINI Ashmead, 1900

MESOCOELINI Viereck, 1918

ANEUROBRACONINI Fahringer, 1936

EARININI Sharkey, 1992^^[[2]](#footnote-3)^^

***Agathis*** Latreille, 1804^^[[3]](#footnote-4)^^

*Aenigmostomus* Ashmead, 1900

*METRIOSOMA* Szépligeti, 1902

*BAEOGNATHA* Kokujev, 1903 van Achterberg (2011)

*LISSAGATHIS* Cameron, 1911

*RHAMPHAGATHIS* Tobias, 1962

***anglica*** Marshall, 1885 E W

*longicauda* Kokujev, 1895 preocc.

?*marshalli* Fahringer, 1937

*albanica* Fischer, 1957

*syriaca* Fischer, 1957

*caucasica* Tobias, 1963

*taiwanensis* Chou & Sharkey, 1989

***assimilis*** Kokujev, 1895 S added by Nixon (1986)

*propinqua* Kokujev, 1895

*jakowlewi* Kokujev, 1895

*sibirica* Telenga, 1933

*anchisiades* Nixon, 1986

***breviseta*** Nees, 1812 E S I

*achterbergi* Nixon, 1986^^[[4]](#footnote-5)^^

***fuscipennis*** (Zetterstedt, 1838, *Microgaster*) E I

*breviseta* misident.

*rostrata* misident.

*glabricula* Thomson, 1895

*schmiedeknechti* Kokujev, 1895

*annulata* Fahringer, 1937

*meridionellae* Fischer, 1957

*albicostellae* Fischer, 1966

*artemesiana* Fischer, 1966

***griseifrons*** Thomson, 1895 E I

*laticarpa* Telenga, 1955

***lugubris*** (Förster, 1863, *Cenostomus*) E S W I

added by Nixon (1986)

*minuta* Niezabitowski, 1910

***montana*** Shestakov, 1932 E added by Simbolotti & van Achterberg (1999)

*zaykovi* Nixon, 1986

***nigra*** Nees, 1812 E

*testaceipes* Fischer, 1957

*kasachstanica* Tobias, 1963

*nixoni* Belokobylskij & Jervis, 1998

***rufipalpis*** Nees, 1812 I

***tibialis*** Nees, 1814 E NMS, det. van Achterberg, added here

*genualis* Marshall, 1898

***varipes*** Thomson, 1895 E added by Nixon (1986)

*simulatrix* Kokujev, 1895

*rufipes* Ivanov, 1899

*dissimilis* Shestakov, 1928

*rufilabialis* Fahringer, 1937

*glabricollis* Telenga, 1955

*serratulae* Tobias, 1963

*lederi* Fischer, 1968

*ariadne* Nixon, 1986

species excluded from the British and Irish list

[***malvacearum*** Latreille, 1805^^[[5]](#footnote-6)^^

*panzeri* (Jurine, 1807, *Ichneumon*)

*metzneriae* Muesebeck, 1967]

***Bassus*** Fabricius, 1804^^[[6]](#footnote-7)^^

*Microdus* Nees, 1814

*DIPLOZON* Haliday, 1833

*Euryzona* Haliday, 1838 nom. nud.

*eumicrodus* Förster, 1863

*HEMIOGASTER* Enderlein, 1920 van Achterberg & Long (2010)

***calculator*** (Fabricius, 1798, *Ichneumon*) E

*abscissus* (Ratzeburg, 1844, *Microdus*)

***Earinus*** Wesmael, 1837

*Diatmetus* Förster, 1863

***elator*** (Fabricius, 1804, *Banchus*) E S I

*nitidulus* (Nees, 1814, *Microdus*)

*thoracicus* (Nees, 1834, *Microdus*)

*major* (Fonscolombe, 1846, *Agathis*)

*pilosus* Tobias, 1960

***gloriatorius*** (Panzer, 1809, *Bassus*) E S I M

*gloriator* (Nees, 1812, *Microdus*)

*ochropes* (Curtis, 1829, *Microdus*) nom. nud.

*affinis* (Wesmael, 1837, *Microdus*)

*delusor* (Wesmael, 1837, *Microdus*)

*tuberculatus* (Wesmael, 1837, *Microdus*)

*varicoxis* (Wesmael, 1837, *Microdus*)

*niger* (Zetterstedt, 1838, *Microgaster*)

*bicingulatus* (Thomson, 1895, *Agathis*)

*ochropes* Lyle, 1920

*ruficoxis* Fahringer, 1937

***transversus*** Lyle, 1920 E Shaw (2005)

***Lytopylus*** Förster, 1863^^[[7]](#footnote-8)^^

*AEROPHILUS* Szépligeti, 1902 Sharkey *et al.* (2006)

*Neomicrodus* Szépligeti, 1908

*Aerophilopsis* Viereck, 1913

*Aerophilina* Enderlein, 1920

*ioxia* Enderlein, 1920

*Hormagathis* Brues, 1926

*Obesomicrodus* Papp, 1971

*FACILAGATHIS* van Achterberg & Chen, 2004

***rufipes*** (Nees, 1814, *Microdus*) E

*germanicus* (Enderlein, 1904, *Braunsia*)

*diversus* Muesebeck, 1933

*amurensis* (Shestakov, 1940, *Microdus*)

***therophilus*** Wesmael, 1837^^[[8]](#footnote-9)^^

*ORGILONEURA* Ashmead, 1900

*Agathiella* Szépligeti, 1902

*AEROPHILIODES* Strand, 1911

***arcuatus*** (Reinhard, 1867, *Microdus*)^^[[9]](#footnote-10)^^

***cingulipes*** (Nees, 1812, *Microdus*) E I

*nantouensis* Chou & Sharkey, 1989

***clausthalianus*** (Ratzeburg, 1844, *Ichneumon*) E I

***conspicuus*** (Wesmael, 1837, *Microdus*) E I

*zonatus* (Marshall, 1885, *Earinus*)

*carpocapsae* Cushman, 1915

*angustatus* (Telenga, 1955, *Microdus*)

*variabilis* Chou & Sharkey, 1989

***tegularis*** (Thomson, 1895, *Agathis*) E added by Simbolotti & van Achterberg (1992)

***tumidulus*** (Nees, 1812, *Microdus*) E S I

*intermedius* (Ivanov, 1899, *Eumicrodus*)

*annae* (Enderlein, 1908, *Microdus*)

*aino* (Watanabe, 1937, *Microdus*)

*ruficoxis* (Fahringer, 1937, *Microdus*)

*rufus* (Fahringer, 1938, *Microdus*)

*bicolor* (Shestakov, 1940, *Microdus*) preocc.

*victoris* (Telenga, 1955, *Microdus*)

*shestakovi* (Shenefelt, 1970, *Agathis*)

*anuphrievi* (Tobias, 1986, *Microdus*)

species *incertae sedis* within Agathidinae^^[[10]](#footnote-11)^^

***dimidiator*** (Nees, 1834, *Microdus*) E^^[[11]](#footnote-12)^^

*cingulator* (Ratzeburg, 1852, *Microdus*)

*laticinctus* (Cresson, 1873, *Microdus*)

*ocellanae* (Richardson, 1913, *Microdus*)

***epinotiae*** (Simbolotti & van Achterberg, 1992, *Bassus*) E

added by Simbolotti & van Achterberg (1992)

***linguarius*** (Nees, 1812, *Microdus*) E

*minor* (Enderlein, 1908, *Microdus*)

*kaszabi* (Papp, 1967, *Vipio*)

***mediator*** (Nees, 1814, *Microdus*)^^[[12]](#footnote-13)^^ E

*lugubrator* (Ratzeburg, 1852, *Microdus*)

***pumilus*** (Ratzeburg, 1844, *Microdus*)^^[[13]](#footnote-14)^^ E NMS, det. Shaw, added here

***rugulosus*** (Nees, 1834, *Microdus*) E I

*compeditus* (Vollenhoven, 1878, *Microdus*)

*punctatus* (Abdinbekova, 1975, *Microdus*)

species excluded from the British and Irish list

[***brevicaudis*** (Reinhard, 1867, *Microdus*)^^[[14]](#footnote-15)^^]

[***nugax*** (Reinhard, 1867, *Microdus*)^^[[15]](#footnote-16)^^

*rufiventris* (Abdinbekova, 1975, *Microdus*)]

Subfamily ALYSIINAE Leach, 1815

Tribe ALYSIINI Leach, 1815

ALLOEINI Ashmead, 1900

***Adelurola*** Strand, 1928

*Adelura* Förster, 1863 preocc.

*Neocarpa* Fischer, 1966

***florimela*** (Haliday, 1838, *Alysia*) E S I M

*multiarticulata* (Marshall, 1898, *Phaenocarpa*)

*pentapleuroides* (Fischer, 1971, *Dapsilarthra*) unavailable

***Alloea*** Haliday, 1833

*DIASPASTA* Förster, 1863

*LAMADATHA* Cameron, 1900

***contracta*** Haliday, 1833 E S W I

*testaceipes* (Cameron, 1900, *Lamadatha*)

***lonchopterae*** Fischer, 1966 E M added by Godfray & Bland (2011)^^[[16]](#footnote-17)^^

***Alysia*** Latreille, 1804

Subgenus ***Alysia*** Latreille, 1804^^[[17]](#footnote-18)^^

*CECHENUS* Illiger, 1807

*Bassus* Nees, 1812

*GONIARCHA* Förster, 1863

*STROPHAEA* Förster, 1863

***alticola*** (Ashmead, 1890, *Pentapleura*) E S W M

*soror* Marshall, 1894

***frigida*** Haliday 1838 E added by Godfray & van Achterberg (2015)

***incongrua*** Nees, 1834 E S I

***lucia*** Haliday, 1838 S

*rudis* Tobias, 1962

*diversiceps* Fischer, 1967

***lucicola*** Haliday, 1838 E S I

***luciella*** Stelfox, 1941 I

***manducator*** (Panzer, 1799, *Ichneumon*) E S W I M

*haematopa* (Gmelin, 1790, *Ichneumon*) preocc.

*stercoraria* Latreille, 1805

*apicalis* Curtis, 1826

*similis* Curtis, 1826

*stercorator* Lamarck, 1835

*curtungula* Thomson, 1895

*bucephala* Marshall, 1898

*manducatrix* Schulz, 1906

***truncator*** (Nees, 1812, *Bassus*) E S I

species excluded from the British and Irish list

[***cingulata*** Nees, 1834 nom. dub.]

Subgenus ***ANARCHA*** Förster, 1863^^[[18]](#footnote-19)^^

***atra*** Haliday, 1838 I

***fuscipennis*** Haliday, 1838 E S I

*obscuripes* Thomson, 1895

***mandibulator*** (Nees, 1812, *Bassus*) E I

*loripes* Haliday, 1838

*mandibulatrix* Schulz, 1906

***rufidens*** Nees, 1834 E I

*puncticollis* Thomson, 1895

***sophia*** Haliday, 1835 I

***subaperta*** Thomson, 1895 E added by Wharton (1988*b*)

*similis* misident.

***thapsina*** Wharton, 1988 I added by Wharton (1988*b*)

***tipulae*** (Scopoli, 1763, *Ichneumon*) E S I

*abdominator* (Nees, 1814, *Bassus*)

*notabilis* (Förster, 1863, *Anarcha*)

***umbrata*** Stelfox, 1941 I

species excluded from the British and Irish list

[***similis*** (Nees, 1812, *Bassus*) nom. dub.]

***Anisocyrta*** Förster, 1863

***perdita*** (Haliday, 1838, *Alysia*) S

***Aphaereta*** Förster, 1863

*Trichesia* Provancher, 1880

*Trinaria* Provancher, 1886

*Aphaerete* Dalla Torre, 1898

*Atopandrium* Graham, 1952^^[[19]](#footnote-20)^^

*Trisynaldis* Fischer, 1958

***debilitata*** Morley, 1933 E W

*loripenne* (Graham, 1952, *Atopandrium*) van Achterberg (1995)

*confluctum* (Fischer, 1958, *Trisynaldis*)

***falcigera*** Graham, 1960 E I

***major*** (Thomson, 1895, *Alysia*) E S I

*major* Marshall, 1898 preocc.

***minuta*** (Nees, 1811, *Stephanus*) E S I

*cephalotes* (Haliday, 1833, *Alysia*)

*fuscipes* (Nees, 1834, *Alysia*)

*confluens* (Ratzeburg, 1844, *Alysia*)

*stigmaticalis* (Thomson, 1895, *Alysia*)

*inepta* Morley, 1933

***pallipes*** (Say, 1829, *Alysia*) E added by Shaw (1983)

*auripes* (Provancher, 1881, *Trichesia*)

*pilicornis* (Provancher, 1886, *Trinaria*)

*californica* Ashmead, 1889

*muscae* Ashmead, 1889

*oscinidis* Ashmead, 1889

*pallidipes* (Dalla Torre, 1898, *Aphaerete*)

*delosa* Viereck, 1905

*subtricarinata* Viereck, 1905

*pegomyiae* Brues, 1907

*sarcophagae* Gahan, 1914

***tenuicornis*** Nixon, 1939 E W I

***Asobara*** Förster, 1863

*SPANISTA* Förster, 1863

***tabida*** (Nees, 1834, *Alysia*) E I

*anomala* (Thomson, 1895, *Alysia*)

*crenulata* (Fahringer, 1935, *Phaenocarpa*)

***Aspilota*** Förster, 1863^^[[20]](#footnote-21)^^

*Dipiesta* Förster, 1863

*Eusynaldis* Zaykov & Fischer, 1982

*Synaldis* misident.

***acutidentata*** (Fischer, 1970, *Synaldis*)^^[[21]](#footnote-22)^^ E S NMS, BMNH, det. Munk, added here

***anaphoretica*** Fischer, 1973 E S I NMS, BMNH, det. Munk, added here

***blasii*** Fischer, 1973 E BMNH, det. Munk, added here

***compressiventris*** Stelfox & Graham, 1951 E

***curta*** Marshall, 1895 E

***daemon*** Stelfox and Graham, 1948 E S I

***delicata*** Fischer, 1973 E S I BMNH, det. Munk, added here

***efoveolata*** Thomson, 1895 E S W NMS, BMNH, det. Munk, added here

*pneumatica* Fischer, 1973

***flagellaris*** Fischer, 1973 E S I BMNH, det. Munk, added here

***fuscicornis*** (Haliday, 1838, *Alysia*) E S I

?*minuta* (Nees, 1812, *Bassus*)

*exile* (Ruthe, 1859, *Orthostigma*)

*dilatata* (Thomson, 1895, *Alysia*)

***globipes*** (Fischer, 1962, *Synaldis*) E S NMS, BMNH, det. Munk, added here

***imparidens*** Fischer, 1974 E S W NMS, BMNH, det. Munk, added here

***insolita*** (Tobias, 1962, *Orthostigma*) E I BMNH, det. Munk, added here

***intermediana*** Fischer, 1975 E added by Notton (1991*b*)

***iocosipecta*** Fischer, 1974 E S I NMS, BMNH, det. Munk, added here

***macrops*** Stelfox & Graham, 1951 E I

***nidicola*** Hedqvist, 1972 E BMNH, det. Munk, added here

***pillerensis*** Fischer, 1973 E BMNH, det. Munk, added here

***ruficornis*** (Nees, 1834, *Alysia*) E S W I

***stenogaster*** Stelfox & Graham, 1951 E

***tetragona*** Fischer, 1976 E S I NMS, BMNH, det. Munk, added here

***vernalis*** Stelfox & Graham, 1951 E S I

***Chasmodon*** Haliday, 1838

***apterus*** (Nees, 1812, *Bassus*) E S W I M

***Cratospila*** Förster, 1863

*HEDYLUS* Marshall, 1891 Papp (2009*a*)

***circe*** (Haliday, 1838, *Alysia*) E I

*habilis* (Marshall, 1891, *Hedylus*) Papp (2009*a*)

*annellata* (Thomson, 1895, *Alysia*)

***Dapsilarthra*** Förster, 1863^^[[22]](#footnote-23)^^

***apii*** (Curtis, 1826, *Alysia*) E S I

*laevipectus* (Thomson, 1895, *Alysia*)

*americana* (Brues, 1907, *Orthostigma*)

***sylvia*** (Haliday, 1839, *Alysia*) E S I

*carpathica* van Achterberg, 1983 van Achterberg (1997)

***Dinotrema*** Förster, 1863^^[[23]](#footnote-24)^^

Subgenus ***Dinotrema*** Förster, 1863

*Aspilota* misident.

*Synaldis* Förster, 1863^^[[24]](#footnote-25)^^

*Coloboma* Förster, 1863

*Spanomeris* Förster, 1863

*SCOTIONEURUS* Provancher, 1886

*PTERUSA* Fischer, 1958 van Achterberg & Vikberg (2014)

*Eudinostigma* Tobias, 1986 Wharton (2002)

***aluum*** (Stelfox & Graham, 1950, *Aspilota*) I

*alva* misspelling

***alysiae*** Munk & Peris-Felipo, 2013 E added by Munk *et al.* (2013)

***areolatum*** (Stelfox & Graham, 1950, *Aspilota*) E S

***brevicorne*** (Nees, 1814, *Bassus*) E

***brevissimicorne*** (Stelfox & Graham, 1948, *Aspilota*) S I

***compressum*** (Haliday, 1838, *Alysia*) E

***concinnum*** (Haliday, 1838, *Alysia*)^^[[25]](#footnote-26)^^ E I

*maximum* (Fischer, 1962, *Synaldis*)

*tyrrhena* (Masi, 1933, *Aspilota*)

***concolor*** (Nees, 1812, *Bassus*) E I

*distractum* (Ruthe, 1859, *Orthostigma*) unavailable

***crassicosta*** (Thomson, 1895, *Alysia*)^^[[26]](#footnote-27)^^ S NMS, det. Munk, added here

***cratocera*** (Thomson, 1895, *Alysia*) S BMNH, det. Munk, added here

***denticulatum*** (Stelfox & Graham, 1951, *Aspilota*) I

***dimidiatum*** (Thomson, 1895, *Alysia*) E BMNH, det. Munk, added here

***distractum*** (Nees, 1834, *Alysia*)

***divisum*** (Stelfox & Graham, 1950, *Aspilota*) E S I

?*aureliae* (Fischer, 1973, *Aspilota*) Papp (2004*c*)

***erythropum*** Förster, 1863 E W I

*praecipuum* (Marshall, 1895, *Aspilota*)

***falsificum*** (Stelfox & Graham, 1950, *Aspilota*) S I

***glabrum*** (Stelfox & Graham, 1951, *Aspilota*) E S I

*venustum* (Tobias, 1962, *Aspilota*)

***insidiatrix*** (Marshall, 1895, *Aspilota*) E

***insignis*** (Stelfox & Graham, 1950, *Aspilota*) E

***iuxtanaeviam*** (Fischer, 1980, *Aspilota*) E added by Fischer (1980)

***jaculans*** (Haliday, 1838, *Alysia*) E S I

***latistigma*** (Fischer, 1962, *Synaldis*)^^[[27]](#footnote-28)^^ E NMS, det. Munk, added here

***lineola*** (Thomson, 1895, *Alysia*) E

***liosoma*** (Stelfox & Graham,1951, *Aspilota*) I

*caudatum* (Thomson, 1895, *Aspilota*) preocc.

***mesocaudatum*** van Achterberg, 1988 E added by van Achterberg (1988*b*)

***microcera*** (Thomson, 1895, *Alysia*) E S W I BMNH, det. Munk, added here

***necrophilum*** (Hedqvist, 1972, *Aspilota*) E added by Disney & Munk (2005)^^[[28]](#footnote-29)^^

***nervosum*** (Haliday, 1833, *Alysia*) E S I

***pulvinatum*** (Stelfox & Graham, 1949, *Aspilota*)^^[[29]](#footnote-30)^^ E S W I

***ruficollis*** (Stelfox & Graham, 1950, *Aspilota*) E I^^[[30]](#footnote-31)^^

***semicompressum*** (Stelfox & Graham, 1949, *Aspilota*) E S I

*parapunctatum* (Fischer, 1976, *Aspilota*) Papp (2004*c*)

***sphaerimembre*** (Fischer, 1973, *Aspilota*) S NMS, det. Munk, added here

***sternaulicum*** (Fischer, 1973, *Aspilota*) E S NMS, det. Munk, added here

***tauricum*** (Telenga, 1935, *Aspilota*) E added by van Achterberg (1988*b*)

***vesparum*** (Stelfox, 1943, *Aspilota*) E S

***vituperatum*** (Fischer, 1974, *Aspilota*) E S NMS, det. Munk, added here

species of *Dinotrema* excluded from the British and Irish list

[***pusillum*** (Nees, 1812, *Bassus*)^^[[31]](#footnote-32)^^]

Subgenus ***LEPTOTREMA*** van Achterberg, 1988^^[[32]](#footnote-33)^^

Wharton (2002)

***dentifemur*** (Stelfox, 1943, *Aspilota*) E S I

Subgenus ***Prosapha*** Förster, 1863

***speculum*** (Haliday, 1838, *Alysia*) E I

*venustum* (Haliday, 1838, *Alysia*)

***grammospila*** Förster, 1863

*Paraorthostigma* Königsmann,1972

***isabella*** (Haliday, 1838, *Alysia*) E S

***rufiventris*** (Nees, 1812, *Bassus*)^^[[33]](#footnote-34)^^ E S I M

*flaviventris* (Haliday, 1838, *Alysia*)

*ochrogaster* (Szépligeti, 1898, *Phaenocarpa*)

***heterolexis*** Förster, 1863

***balteata*** (Thomson, 1895, *Alysia*) E S W I

***dictynna*** (Marshall, 1895, *Adelura*) E S I

***Idiasta*** Förster, 1863

*Euphaenocarpa* Tobias, 1975

***dichrocera*** Königsmann, 1960 E NMS, det. Shaw, added here

***maritima*** (Haliday, 1838, *Alysia*) E S I

***nephele*** (Haliday, 1838, *Alysia*) E S

***mesocrina*** Förster, 1863

*pseudomesocrina* Königsmann, 1959

***indagatrix*** Förster, 1863 E

*venatrix* Marshall, 1895

***Orthostigma*** Ratzeburg, 1844

*DELOCARPA* Förster, 1863

*ISCHNOCARPA* Förster, 1863

***cratospilum*** (Thompson, 1895, *Alysia*) E added by Godfray & van Achterberg (2015)

***longicorne*** Königsmann, 1969 I

***maculipes*** (Haliday, 1838, *Alysia*) E I

***pumilum*** (Nees, 1834, *Alysia*) E I

*flavipes* (Ratzeburg, 1844, *Aphidius*)

*fulvipes* Rondani, 1876 nom. nud.

*brunnipes* Ratzeburg, 1852

*bruneipes* Dalla Torre, 1898

***PANEREMA*** Förster, 1863^^[[34]](#footnote-35)^^

***fulvicornis*** (Haliday, 1838, *Alysia*) W I

***inops*** Förster, 1863 E W I

***Pentapleura*** Förster, 1863

*OPISENDEA* Förster, 1863

*Gnathospila* Fischer, 1966

***angustula*** (Haliday, 1838, *Alysia*) E S I

*laevipleuris* (Tobias, 1962, *Aspilota*)

***fuliginosa*** (Haliday, 1838, *Alysia*) E W I

*carinata* (Thomson, 1895, *Alysia*)

***pumilio*** (Nees, 1812, *Bassus*) E S W I

*triticaphis* (Fitch, 1861, *Toxares*)

*mesocrinoides* Goidanich, 1936

***Phaenocarpa*** Förster, 1863^^[[35]](#footnote-36)^^

Subgenus ***HOMOPHYLA*** Förster, 1863

***pullata*** (Haliday, 1838, *Alysia*) E I

species of *Phaenocarpa* (*Homophyla*) excluded from the British and Irish list

[***pegomyiae*** Marshall, 1898^[[36]](#footnote-37)^]

Subgenus ***Phaenocarpa*** Förster, 1863

*Idiolexis* Förster, 1863 Wharton (2002)

*Mesothesis* Förster, 1863

*SATHRA* Förster, 1863

*ASYNAPHES* Provancher, 1886

*Kahlia* Ashmead, 1900

*HOLCALYSIA* Cameron, 1905

*STIRALYSIA* Cameron, 1910

*Rhopaloneura* Stelfox, 1941

***canaliculata*** Stelfox, 1941 E I

***conspurcator*** (Haliday, 1838, *Alysia*) E S W I

*arctica* (Thomson, 1895, *Alysia*)

*tatrica* Niezabitowski, 1910

*remota* Papp, 1981

***eugenia*** (Haliday, 1838, *Alysia*) E S I

*pectoralis* (Zetterstedt, 1838, *Alysia*)

*orbicularis* Gurasashvilli, 1983

***eunice*** (Haliday, 1838, *Alysia*) E W I

*nimia* Stelfox, 1941

***flavipes*** (Haliday, 1838, *Alysia*) E I

***frequentator*** (Zetterstedt, 1838, *Alysia*) W added by Godfray & van Achterberg (2015)

*frequentatrix* (Schulz, 1906, *Alysia*)

***galatea*** (Haliday, 1838, *Alysia*) S I

***helophilae*** van Achterberg, 1998 E added by van Achterberg (1998)

***livida*** (Haliday, 1838, *Alysia*) E I

*debilis* (Förster, 1863, *Sathra*) van Achterberg (1998)

***luteipes*** Stelfox, 1950 I

***maria*** (Haliday, 1838, *Alysia*) E I

***nina*** (Haliday, 1838, *Alysia*) S^^[[37]](#footnote-38)^^

***notabilis*** Stelfox, 1944 S I

***picinervis*** (Haliday, 1838, *Alysia*) E S I

*americana* Ashmead, 1889

***pratellae*** (Curtis, 1826, *Alysia*) E S

*piceator* (Zetterstedt, 1838, *Alysia*)

*psalliotae* Telenga, 1935 van Achterberg (2014)

***punctigera*** (Haliday, 1838, *Alysia*)^^[[38]](#footnote-39)^^ E I

***ruficeps*** (Nees, 1812, *Bassus*) E S W I M

*testacea* (Nees, 1812, *Bassus*)

*gracilis* (Curtis, 1826, *Alysia*)

*pallida* (Curtis, 1826, *Alysia*)

*agricolator* (Zetterstedt, 1838, *Alysia*)

*oculator* (Ratzeburg, 1848, *Alysia*)

*rubriceps* (Provancher, 1883, *Alysia*) preocc.

*rubricepes* Provancher, 1888

*testaceipes* (Cameron, 1905, *Holcalysia*)

*divergens* Fischer, 1975

*fervida* Fischer, 1975

*incerta* Fischer, 1975

*meritoria* Papp, 1981

*ferga* Papp, 1982

***tacita*** Stelfox, 1941 I

*caucasica* Gurasashvili, 1983 preocc.

*caucasicola* Tobias, 1986

***trisulcata*** Stelfox, 1950 I

***Syncrasis*** Förster, 1863

*PHAENOLYTA* Förster, 1863

***fucicola*** (Haliday, 1838, *Alysia*) I

***halidayi*** (Förster, 1863, *Phaenolyta*) E I

*fuscipes* preocc. unavailable

***Tanycarpa*** Förster, 1863

*Acrobela* Förster, 1863 Wharton (2002)

*EPICLISTA* Förster, 1863

*HYPOSTROPHA* Förster, 1863

***bicolor*** (Nees, 1812, *Bassus*) E I

*ancilla* (Haliday, 1838, *Alysia*)

***gracilicornis*** (Nees, 1812, *Bassus*) I

***mitis*** Stelfox, 1941 E I

***punctata*** van Achterberg, 1976

***rufinotata*** (Haliday, 1838, *Alysia*) E I

*carinata* (Förster, 1863, *Acrobela*)

*erythrogaster* (Förster, 1863, *Epiclista*)

*foersteri* (Shenefelt, 1974, *Alysia*)

***Trachyusa*** Ruthe, 1854^^[[39]](#footnote-40)^^

*COSMIOCARPA* Förster, 1863

***aurora*** (Haliday, 1838, *Alysia*) E I

*nigriceps* Ruthe, 1854

***nigrothoracica*** van Achterberg & O’Connor, 1990 E I

added by van Achterberg & O’Connor (1990)

Tribe DACNUSINI Förster, 1863^^[[40]](#footnote-41)^^

***Amyras*** Nixon, 1943

***clandestina*** (Haliday, 1839, *Alysia*) I

*quadridentata* (Thomson, 1895, *Dacnusa*)

***Aristelix*** Nixon, 1943

***phaenicura*** (Haliday, 1839, *Alysia*) E I

*phoenicura* misspelling

***Chaenusa*** Haliday, 1839^^[[41]](#footnote-42)^^

*CHOREBIDEA* Viereck, 1914

*CHOREBIDEA* Nixon, 1943 preocc.

*CHOREBIDELLA* Riegel, 1947

***conjungens*** (Nees, 1811, *Bracon*) E S I

*conjugens* misspelling

***elongata*** Stelfox, 1957 I

***limoniadum*** (Marshall, 1896, *Chorebus*)^^[[42]](#footnote-43)^^ E

***lymphata*** (Haliday, 1839, *Alysia*)^^[[43]](#footnote-44)^^ E I

***naiadum*** (Haliday, 1839, *Alysia*) E S I

*naiadum* (Curtis, 1837, *Chorebus*) nom. nud.

***nereidum*** (Haliday, 1839, *Alysia*) E I

*nereidum* (Curtis, 1837, *Chorebus*) nom. nud.

***opaca*** Stelfox, 1957 I

***Chorebus*** Haliday, 1833

*Ametria* Förster, 1863

*Gyrocampa* Förster, 1863

*PHAENOLEXIS* Förster, 1863

*STIPHROCERA* Förster, 1863

*DIPLUSIA* Ruthe, 1882

*Etriptes* Nixon, 1943

*PARAGYROCAMPA* Tobias, 1962

***abaris*** (Nixon, 1943, *Dacnusa*) E S

***abnormiceps*** (Nixon, 1943, *Dacnusa* E

*quadriceps* (Nixon, 1941, *Dacnusa*) preocc.

***agraules*** (Nixon, 1945, *Dacnusa*) E

***albipes*** (Haliday, 1839, *Alysia*) E S I

***alecto*** (Morley, 1924, *Rhizarcha*) E S I

*turissa* (Nixon, 1937, *Dacnusa*)

***alua*** (Nixon, 1944, *Dacnusa*) I

***amasis*** (Nixon, 1945, *Dacnusa*) E

***ampliator*** (Nees, 1834, *Alysia*) E S I

*nigricornis* (Förster, 1863, *Stiphrocera*)

***anasellus*** (Stelfox, 1952, *Dacnusa*) S I

***angelicae*** (Nixon, 1945, *Dacnusa*) E

***anita*** (Nixon, 1943, *Dacnusa*) E S

***aphantus*** (Marshall, 1895, *Dacnusa*) E S W I

***apollyon*** (Morley, 1924, *Dacnusa*) E I

***ares*** (Nixon, 1944, *Dacnusa*) E

***armida*** (Nixon, 1945, *Dacnusa*) E S W

***artemisiellus*** Griffiths, 1968 E

***asramenes*** (Nixon, 1943, *Dacnusa*) E I

***avesta*** (Nixon, 1944, *Dacnusa*) E S

***bathyzonus*** (Marshall, 1895, *Dacnusa*) E I

*ornatus* (Telenga, 1935, *Dacnusa*)

***bensoni*** (Nixon, 1943, *Dacnusa*) E S

***bres*** (Nixon, 1944, *Dacnusa*) E I

***brevicornis*** (Thomson, 1895, *Dacnusa*) E S I

*chrysippe* (Nixon, 1944, *Dacnusa*)

*ea* (Nixon, 1944, *Dacnusa*)

***caelebs*** (Nixon, 1944, *Dacnusa*) E

***calthae*** Griffiths, 1967 E

***cambricus*** Griffiths, 1968 W

***cinctus*** (Haliday, 1839, *Alysia*) E I

*castaneiventris* (Thomson, 1895, *Dacnusa*)

***coxator*** (Thomson, 1895, *Dacnusa*) E S W

***crassipes*** (Stelfox, 1954, *Dacnusa*) S I

***credne*** (Nixon, 1944, *Dacnusa*) E S I

***crenulatus*** (Thomson, 1895, *Dacnusa*) E S I

*elegantulus* (Nixon, 1937, *Dacnusa*)

***crocale*** (Nixon, 1945, *Dacnusa*) E

***cubocephalus*** (Telenga, 1934, *Rhizarcha*) I

*cyclops* (Nixon, 1937, *Dacnusa*) Tobias (1986)

***cylindricus*** (Telenga, 1934, *Dacnusa*) E S

*cybele* (Nixon, 1937, *Dacnusa*) Tobias (1986)

***cytherea*** (Nixon, 1937, *Dacnusa*) E S I

*calliope* (Nixon, 1944, *Dacnusa*)

*tesmia* (Nixon, 1944, *Dacnusa*)

***dagda*** (Nixon, 1943, *Dacnusa*) E

***daimenes*** (Nixon, 1945, *Dacnusa*) E S

***deione*** (Nixon, 1944, *Dacnusa*) E S

***didas*** (Nixon, 1944, *Dacnusa*) E S

***difficilis*** Griffiths, 1968 E

***diremtus*** (Nees, 1834, *Alysia*) E I

*diremptus* (Haliday, 1839, *Alysia*)

***dirona*** (Nixon, 1945, *Dacnusa*) E I

***enephes*** (Nixon, 1945, *Dacnusa*) E

***eros*** (Nixon, 1937, *Dacnusa*) E S I

***esbelta*** (Nixon, 1937, *Dacnusa*) E S I

***euryale*** (Nixon, 1944, *Dacnusa*) E

***fallaciosae*** Griffiths, 1967 E

***fallax*** (Nixon, 1937, *Dacnusa*) E S I

***flavipes*** (Goureau, 1851, *Dacnusa*) E S I

*raissa* (Nixon, 1937, *Dacnusa*)

***fordi*** (Nixon, 1954, *Dacnusa*) E

***foveolus*** (Haliday, 1839, *Alysia*) E S W I

***fuscipennis*** (Nixon, 1937, *Dacnusa*) E

***gedanensis*** (Ratzeburg, 1852, *Alysia*) E

*anguligena* (Nixon, 1937, *Dacnusa*)

***glaber*** (Nixon, 1944, *Dacnusa*) E I

***glabriculus*** (Thomson, 1895, *Dacnusa*) E S I

*cortipalpis* (Nixon, 1937, *Dacnusa*)

***hilaris*** Griffiths, 1967 E

***hirtigena*** Stelfox, 1957 I

***humeralis*** Griffiths, 1968 I

***incertus*** (Goureau, 1851, *Dacnusa*) E

***iphias*** (Nixon, 1943, *Dacnusa*) E

***kama*** (Nixon, 1945, *Dacnusa*) E

***lanigerus*** (Stelfox, 1957, *Gyrocampa*) I

***lar*** (Morley, 1924, *Dacnusa*) E

*innanus* (Nixon, 1943, *Dacnusa*)

***larides*** (Nixon, 1944, *Dacnusa*) E I

***lateralis*** (Haliday, 1839, *Alysia*) E S W I

*fuscula* (Haliday, 1839, *Alysia*)

*albicoxa* (Thomson, 1895, *Dacnusa*)

***leptogaster*** (Haliday, 1839, *Alysia*) E I

*naenia* (Morley, 1924, *Dacnusa*)

*dinae* (Burghele, 1960, *Dacnusa*)

***longicornis*** (Nees, 1811, *Bracon*) E S I

*affinis* (Nees, 1812, *Bassus*)^^[[44]](#footnote-45)^^

***lugubris*** (Nixon, 1937, *Dacnusa*) E I

***luzulae*** Griffiths, 1966 S added by Godfray & Bland (2011)

***lychnidis*** Griffiths, 1967 E

***maculigastra*** Shenefelt, 1974 E I

*maculata* (Nixon, 1944, *Dacnusa*) preocc.

***merellus*** (Nixon, 1937, *Dacnusa*) E S I

***merion*** (Nixon, 1945, *Dacnusa*) E

***miodes*** (Nixon, 1949, *Gyrocampa*) E I

***misellus*** (Marshall, 1895, *Dacnusa*) E

***mitrus*** (Nixon, 1945, *Dacnusa*) E S I

***nanus*** (Nixon, 1943, *Dacnusa*) E

***navicularis*** (Nees, 1812, *Bassus*) E

***nerissus*** (Nixon, 1937, *Dacnusa*) E

***nigriscaposus*** (Nixon, 1949, *Gyrocampa*) I

*propodealis* (Nixon, 1949, *Gyrocampa*)

***ninella*** (Nixon, 1945, *Dacnusa*) E S I

***nobilis*** Griffiths, 1968 E I

***nomia*** (Nixon, 1937, *Dacnusa*) E I

***nydia*** (Nixon, 1937, *Dacnusa*) E S I

***orbiculatae*** Griffiths, 1967 E I

***ovalis*** (Marshall, 1896, *Dacnusa*) E I

***parvungula*** (Thomson, 1895, *Dacnusa*) E S I

*acco* (Nixon, 1943, *Dacnusa*)

***perkinsi*** (Nixon, 1944, *Dacnusa*) E

***petiolatus*** (Nees, 1834, *Alysia*) E S I

***phaedra*** (Nixon, 1937, *Dacnusa*) E S

***pimpinellae*** Griffiths, 1967 E

***pione*** (Nixon, 1944, *Dacnusa*) E

***poemyzae*** Griffiths, 1968 E S

***posticus*** (Haliday, 1839, *Alysia*) E S I

*gracilis* (Nees, 1834, *Alysia*) van Achterberg (1997)

*egregia* (Marshall, 1895, *Dacnusa*)

*dentatus* (Tobias, 1962, *Dacnusa*) Tobias (1986)

***pulverosus*** (Haliday, 1839, *Alysia*) E I

*marsyas* (Nixon, 1937, *Dacnusa*) van Achterberg (1997)

***punctum*** (Goureau, 1851, *Dacnusa*) E S I

***resa*** (Nixon, 1937, *Dacnusa*) E

***rhanis*** (Nixon, 1943, *Dacnusa*) S

***risilis*** (Nixon, 1949, *Gyrocampa*) I

***rondanii*** (Giard, 1904, *Dacnusa*) E

*galbus* (Nixon, 1944, *Dacnusa*)

***rotundiventris*** (Thomson, 1895, *Dacnusa*) I

***rousseaui*** (Schulz, 1907, *Dacnusa*)^^[[45]](#footnote-46)^^

***ruficollis*** (Stelfox, 1957, *Gyrocampa*) I

***rufimarginatus*** (Stelfox, 1954, *Dacnusa*) I

***scabiosae*** Griffiths, 1967 E

***scabrifossa*** Stelfox, 1957 I

***selene*** (Nixon, 1937, *Dacnusa*) E S

***senilis*** (Nees, 1812, *Bassus*) E S W I

*tomentosus* (Thomson, 1895, *Dacnusa*)

*nemesis* (Morley, 1924, *Dacnusa*)

***serus*** (Nixon, 1937, *Dacnusa*) E

***siniffa*** (Nixon, 1937, *Dacnusa*) E I

***solstitialis*** (Stelfox, 1952, *Dacnusa*) E

***spenceri*** Griffiths, 1964 E S

***striola*** Stelfox, 1957 I

***sylvestris*** Griffiths, 1967 E S I

***talaris*** (Haliday, 1839, *Alysia*) E S I

***tamiris*** (Nixon, 1943, *Dacnusa*) E

***tamsi*** (Nixon, 1944, *Dacnusa*) E

***tanis*** (Nixon, 1945, *Dacnusa*) E

***tenellae*** Griffiths, 1967 S I added by Griffiths (1984)

***thecla*** (Nixon, 1943, *Dacnusa*) E

***thisbe*** (Nixon, 1937, *Dacnusa*) E

***thusa*** (Nixon, 1937, *Dacnusa*) E I

***transversus*** (Nixon, 1954, *Dacnusa*) E S I

***trilobomyzae*** Griffiths, 1968 E S

***uliginosus*** (Haliday, 1839, *Alysia*) E I

*thienemanni* (Ruschka, 1913, *Gyrocampa*)

***uma*** (Nixon, 1944, *Dacnusa*) E

***varuna*** (Nixon, 1945, *Dacnusa*) E S

***vernalis*** Griffiths, 1968 E

***vitripennis*** Griffiths, 1968 S W I

***Coelinidea*** Viereck, 1913^^[[46]](#footnote-47)^^

*Lepton* Zetterstedt, 1838 preocc.

*ERIOCOELINIUS* Viereck, 1913

*FISCHERASTRIOLUS* Perepechayenko, 1999

***elegans*** (Curtis, 1829, *Chaenon*) E I

*brevicornis* (Curtis, 1829, *Chaenon*)

*cingulatus* (Curtis, 1829, *Chaenon*)

*rufinotatus* (Curtis, 1829, *Chaenon*)

*similis* (Curtis, 1829, *Chaenon*)

***fuliginosus*** (Curtis, 1829, *Chaenon*) E

***gracilis*** (Curtis, 1829, *Chaenon*) E S I

*attenuator* (Zetterstedt, 1838, *Lepton*)

***niger*** (Nees, 1811, *Stephanus*) E I

*affinis* (Curtis, 1829, *Chaenon*)

*nigricans* (Westwood, 1835, *Chaenon*)

*olivieri* (Guérin-Ménéville, 1842, *Alysia*)

***obscurus*** (Curtis, 1829, *Chaenon*) E

***podagricus*** (Haliday, 1839, *Alysia*) I

***ruficollis*** (Herrich-Schäffer, 1838, *Coelinius*) E

*procerus* (Haliday, 1839, *Alysia*)

***viduus*** (Curtis, 1829, *Chaenon*) E I

*ater* (Curtis, 1837, *Chaenon*) nom. nud.

***Coelinius*** Nees, 1818^^[[47]](#footnote-48)^^

*Chaenon* Curtis, 1829

*Copisura* Schiødte,1837

*Copidura* Förster,1862

***parvulus*** (Nees, 1811, *Chaenon*) E S I M

?*cultriformis* (Latreille, 1802, *Ichneumon*)

*circulator* (Gravenhorst, 1807, *Ichneumon*) preocc.

*anceps* (Curtis, 1829, *Chaenon*)

*rimator* (Schiødte, 1837, *Copisura*)

*bicarinatus* Herrich-Schäffer, 1838

*flexuosus* Herrich-Schäffer, 1838

*bicolor* Maréchal, 1938

***Coloneura*** Förster, 1863

*ISOMERISTA* Förster, 1863

*TRISISA* Förster, 1863

*MERITES* Nixon, 1943

*PRIAPSIS* Nixon, 1943

***dice*** (Nixon, 1943, *Priapsis*) E

***stylata*** Förster, 1863 E I

*exilis* (Förster, 1863, *Trisisa*)

*oligomera* (Förster, 1863, *Isomerista*)

*taras* (Nixon, 1943, *Merites*)

***DACNUSA*** Haliday, 1833

*AGONIA* Förster, 1863

*Aphanta* Förster, 1863

*BRACHYSTROPHA* Förster, 1863

*LIPOSCIA* Förster, 1863

*PACHYSEMA* Förster, 1863

*RHIZARCHA* Förster, 1863

*TANYSTROPHA* Förster, 1863

*RADIOLARIA* Provancher, 1886

*Coloneurella* van Achterberg, 1976

***abdita*** Haliday, 1838 E S W I

*incidens* Thomson, 1895

*lepida* Marshall, 1896

***adducta*** (Haliday, 1839, *Alysia*) E S I^^[[48]](#footnote-49)^^

*abducta* misspelling
***alticeps*** Nixon, 1937 E S

***aquilegiae*** Marshall, 1896 E S

***areolaris*** (Nees, 1811, *Bracon*) E S W I

*lysias* Goureau, 1851

***astarte*** (Nixon, 1948, *Rhizarcha*) E

***aterrima*** Thomson, 1895 E

***confinis*** Ruthe, 1859 E W I

*minuta* (Curtis, 1826, *Alysia*)^^[[49]](#footnote-50)^^

***delphinii*** Griffiths, 1967 E

***discolor*** (Förster, 1863, *Liposcia*) E S I

*cercides* (Nixon, 1954, *Pachysema*)

***dryas*** (Nixon, 1948, *Rhizarcha*) E

***ergeteles*** (Nixon, 1954, *Pachysema*) I

***euphrasiella*** Griffiths, 1984 I added by Griffiths (1984)

***evadne*** Nixon, 1937 E S I

***faeroeensis*** (Roman, 1917, *Rhizarcha*) E S W I

*lestes* Nixon, 1937

***fasciata*** Stelfox, 1954 I

***hospita*** (Förster, 1863, *Aphanta*) E S I

***laeta*** (Nixon, 1954, *Pachysema*) E S I

***laevipectus*** Thomson, 1895 E S W I

*nox* (Morley, 1924, *Rhizarcha*)

***lissos*** (Nixon, 1954, *Pachysema*) E

***longiradialis*** Nixon, 1937 E S I

***lugens*** (Haliday, 1839, *Alysia*) E I

***macrospila*** (Haliday, 1839, *Alysia*) E S I

***maculipes*** Thomson, 1895 E S W I

***maxima*** (Fischer, 1961, *Pachysema*) W

***melicerta*** (Nixon, 1954, *Pachysema*) E S I

*fumipes* Tobias, 1998

***merope*** (Nixon, 1948, *Rhizarcha*) E

***metula*** (Nixon, 1954, *Pachysema*) E S I

***monticola*** (Förster, 1863, *Brachystropha*) I

*mutia* (Nixon, 1948, *Rhizarcha*)

*coracina* Stelfox, 1957

***nigrella*** Griffiths, 1967 S added by Godfray & Bland (2011)

***nigropygmaea*** Stelfox, 1954 I

***obesa*** Stelfox, 1954 S I

***ocyroe*** Nixon, 1937 E S W I

***plantaginis*** Griffiths, 1967 E S W I

*discolor* misident.

***pubescens*** (Curtis, 1826, *Alysia*) E S I

*exserens* (Nees, 1834, *Alysia*)

***sibirica*** Telenga, 1934 E S W I

*comis* (Nixon, 1954, *Pachysema*)

***soma*** (Nixon, 1948, *Rhizarcha*) S I

***stramineipes*** (Haliday, 1839, *Alysia*) E S I

*haemorrhoa* (Förster, 1863, *Tanystropha*)

*longicauda* Thomson, 1895^^[[50]](#footnote-51)^^

***tarsalis*** Thomson, 1895 E S W I

*nitetis* (Nixon, 1948, *Rhizarcha*)

***temula*** (Haliday, 1839, *Alysia*) E S I

***veronicae*** Griffiths, 1967 E S

***Epimicta*** Förster, 1863

***marginalis*** (Haliday, 1839, *Alysia*) E I

***Exotela*** Förster, 1863^^[[51]](#footnote-52)^^

*MESORA* Förster, 1863

*TOXELEA* Nixon, 1943

*ANTRUSA* Nixon, 1943

***cyclogaster*** Förster, 1863 E S W I

*bellina* (Nixon, 1937, *Dacnusa*)

*umbellina* (Nixon, 1954, *Toxelea*)^^[[52]](#footnote-53)^^

*sonchina* Griffiths, 1967

***dives*** (Nixon, 1954, *Toxelea*) I

***flavicoxa*** (Thomson, 1895, *Dacnusa*) E S I

***gilvipes*** (Haliday, 1839, *Alysia*) E S I

*albilabris* (Thomson, 1895, *Dacnusa*)

***hera*** (Nixon, 1937, *Dacnusa*) E S M

***interstitialis*** (Thomson, 1895, *Dacnusa*)^^[[53]](#footnote-54)^^

*mamertes* (Nixon, 1943, *Dacnusa*)

***lonicerae*** Griffiths, 1967 E S

***melanocera*** (Thomson, 1895, *Dacnusa*) E I

*persimilis* (Nixon, 1954, *Antrusa*)

***phryne*** (Nixon, 1954, *Toxelea*) E S

***spinifer*** (Nixon, 1954, *Toxelea*) E S I

***sulcata*** (Tobias, 1962, *Pachysema*) E S I

***vaenia*** (Nixon, 1954, *Antrusa*) E

***viciae*** Griffiths, 1984 E added by Griffiths (1984)

***Laotris*** Nixon, 1943

***striatula*** (Haliday, 1839, *Alysia*) E S I

***Polemochartus*** Schulz, 1911

***liparae*** (Giraud, 1863, *Polemon*) E

***melas*** (Giraud, 1863, *Polemon*) E added by Shaw & Jennings (2008)

***Protodacnusa*** Griffiths, 1964

***litoralis*** Griffiths, 1964 I

***tristis*** (Nees, 1834, *Alysia*) E I

*ampliator* (Haliday, 1839, *Alysia*) preocc.

*longistigma* (Telenga, 1935, *Dacnusa*)

***Sarops*** Nixon, 1942

***rea*** Nixon, 1942 E

***Synelix*** Förster, 1863

*ECTILIS* Nixon, 1943

***semirugosa*** (Haliday, 1839, *Alysia*) E S W I

*agnata* Förster, 1863

*amaurosomae* (Telenga, 1935, *Dacnusa*)

***Tates*** Nixon, 1943

***heterocera*** (Thomson, 1895, *Dacnusa*) E S

***trachionus*** Haliday, 1833

Subgenus ***trachionus*** Haliday, 1833

*AENONE* Curtis, 1837 preocc. van Achterberg (1997)

*AENONE* Haliday, 1838 preocc., nom. nud.

van Achterberg (1997)

*OENONE* Haliday, 1839 preocc. van Achterberg (1997)

*Symphya* Förster, 1863 van Achterberg (1997)

*ANARMUS* Ruthe, 1882 van Achterberg (1997)

***mandibularis*** (Nees, 1816, *Sigalphus*) E W I

Subgenus ***Planiricus*** Perepechayenko, 2000

***hians*** (Nees, 1816, *Sigalphus*) E S I

***ringens*** (Haliday, 1839, *Alysia*) E S I

Subfamily aPHIDIINAE Haliday, 1833^^[[54]](#footnote-55)^^

Tribe Aphidiini Haliday, 1833

***ADIALYTUS*** Förster, 1863

***ambiguus*** (Haliday, 1834, *Aphidius*) S W

*diminuens* (Nees, 1834, *Aphidius*)

?*exiguus* (Haliday, 1834, *Aphidius*)^^[[55]](#footnote-56)^^

*delhiensis* (Subba Rao & Sharma, 1960, *Aphidius*)

*arvicola* (Starý, 1961, *Lysiphlebus*)

*mackaueri* (Starý, 1961, *Lysiphlebus*)

*crocinus* (Mackauer, 1962, *Lysiphlebus*)

***salicaphis*** (Fitch, 1855, *Trioxys*) E W added by Baker & Broad (2009)

*populaphis* (Fitch, 1855, *Trioxys*)

*tenuis* Förster, 1863

*salicaphidis* (Ashmead, 1889, *Lipolexis*)

*laticephalus* (Telenga, 1953, *Aphidius*)

***Aphidius*** Nees, 1818

*INCUBUS* Schrank, 1802 nom. ob.

*Theracmion* Holmgren, 1872

*Euaphidius* Mackauer, 1961

***absinthii*** Marshall, 1896^^[[56]](#footnote-57)^^ E

***aquilus*** Mackauer, 1961 E W

*sicarius* Mackauer, 1961

*callipterinellae* (Takada, 1966, *Lysaphidus*)

***asteris*** Haliday, 1834 E W

*melanocephalus* (Nees, 1811, *Bracon*)

*lutescens* Haliday, 1834

*commodus* Gahan, 1926

*artemisiae* Ivanov, 1927

***avenae*** Haliday, 1834 E W I

*picipes* (Nees, 1811, *Bracon*) suppressed

*crithmi* Marshall, 1896

*granarius* Marshall, 1896

*pascuorum* Marshall, 1896

*hungaricus* (Györfi, 1958, *Lysiphlebus*)

*caraganae* Starý, 1963

***cingulatus*** Ruthe, 1859 E W

*arcticus* (Holmgren, 1872, *Theracmion*)

*gregarius* Marshall, 1872

*lachni* Ashmead, 1889

*pterocommae* Ashmead, 1889

*pterocommae* Marshall, 1896 preocc.

*luzhetzki* Telenga, 1958

***#colemani*** Viereck, 1912^^[[57]](#footnote-58)^^

*huebrichi* Brèthes, 1913

*platensis* Brèthes, 1913

*porteri* Brèthes, 1915

*aphidiphilus* Benoit, 1955

*leroyi* Benoit, 1955

*transcaspicus* Telenga, 1958

***eadyi*** Starý, Gonzalez & Hall, 1980 E added by Müller *et al.* (1999)

*urticae* misident.

***eglanteriae*** Haliday, 1834

***ervi*** Haliday, 1834 E W I

*infirmus* (Nees, 1811, *Bracon*)

*ulmi* Marshall, 1896

*medicaginis* Marshall, 1898

*fumipennis* Györfi, 1958

*nigrescens* Mackauer, 1962

*mirotarsi* Starý, 1963

***fumatus*** Haliday, 1834

***funebris*** Mackauer, 1961 E W added by Pennacchio (1989)

*cirsii* Ivanov, 1925 preocc.

*bispinosus* Telenga, 1958

*eriophori* Mackauer, 1967

***hortensis*** Marshall, 1896 E W

*berberidis* Smith, 1944

***matricariae*** Haliday, 1834 E W I

*arundinis* Haliday, 1834

*cirsii* Haliday, 1834 preocc.

*phorodontis* Ashmead, 1889

*chrysanthemi* Marshall, 1896

*lychnidis* Marshall, 1896

*polygoni* Marshall, 1896

*affinis* Quilis, 1931

*baudysi* Quilis, 1931

*discrytus* Quilis, 1931

*merceti* Quilis, 1931

*obscuriformis* Quilis, 1931

*valentinus* Quilis, 1931

*renominatus* Hincks, 1943

*nigriteleus* Smith, 1944

***microlophii*** Pennacchio & Tremblay, 1987 E W

added by Pennacchio (1989)^^[[58]](#footnote-59)^^

***rhopalosiphi*** de Stefani-Perez, 1902 E

*equiseticola* Starý, 1963

*poacearum* Starý, 1963

***ribis*** Haliday, 1834 E

*ribaphidis* (Ashmead, 1889, *Lysiphlebus*)

*scabiosae* Marshall, 1896

*ribis* Ashmead, 1898 preocc.

***rosae*** Haliday, 1833 E W I

*aphidum* (Linnaeus, 1758, *Ichneumon*) nom. ob.

*aphidator* (Thunberg, 1824, *Ichneumon*) nom. ob.

*rosae* Curtis, 1831 nom. nud.

*protaeus* Wesmael, 1835

*rosarum* Nees, 1834

*xanthostoma* Bouché, 1834

*protaeus* Wesmael, 1835

*cancellatus* Buckton, 1876

***rubi*** Starý, 1962^^[[59]](#footnote-60)^^ E added by Müller *et al.* (1999)

***salicis*** Haliday, 1834 E W

*restrictus* Nees, 1834

*duodecimarticulatus* Ratzeburg, 1852

*dauci* Marshall, 1896

***setiger*** (Mackauer, 1961, *Euaphidius*) W added by Baker & Broad (2009)

*aceri* Ivanov, 1925

***sonchi*** Marshall, 1896 E W

***tanacetarius*** Mackauer, 1962^^[[60]](#footnote-61)^^

*tanaceti* Curtis, 1837 nom. nud.

*tanaceticola* Starý, 1963

***urticae*** Haliday, 1834 E

*euphorbiae* Marshall, 1896

*longulus* Marshall, 1896

*lonicerae* Marshall, 1896

*silenes* Marshall, 1896

*goidanichi* Quilis, 1932

*ivanovae* Telenga, 1958

*rubi* Starý, 1962

*silvaticus* Starý, 1962

*aulacorthi* Starý, 1963

***uzbekistanicus*** Luzhetski, 1960 E

*urticae* misident. Powell (1982)

?*beltrani* Quilis, 1931^^[[61]](#footnote-62)^^

?*indivisus* Quilis, 1931

?*macropterus* Quilis, 1931

?*pailloti* Quilis, 1931

*impressus* Mackauer, 1965

doubtfully placed species

[***constrictus*** (Nees, 1811, *Bracon*)^^[[62]](#footnote-63)^^]

[***dimidiatus*** Curtis, 1831]

[***pallidinotus*** Haliday, 1834 nom. nud.]

[***pseudoplatanus*** Curtis, 1837^^[[63]](#footnote-64)^^

*constrictus* misident.]

[***viminalis*** Haliday, 1834 nom. nud.]

***Betuloxys*** Mackauer, 1960

***compressicornis*** (Ruthe, 1859, *Trioxys*) E W I

*testaceus* (Stelfox, 1948, *Trioxys*)

***BINODOXYS*** Mackauer, 1960

*MISAPHIDUS* Rondani, 1848 unavailable^^[[64]](#footnote-65)^^

***acalephae*** (Marshall, 1896, *Aphidius*) E

*rietscheli* (Mackauer, 1959, *Trioxys*)

*urticae* (Mackauer, 1959, *Trioxys*)

***angelicae*** (Haliday, 1833, *Aphidius*) W

*placidus* (Gautier, 1922, *Trioxys*)

*boscai* (Quilis, 1931, *Trioxys*)

*fumariae* (Quilis, 1931, *Trioxys*)

*granatensis* (Quilis, 1931, *Trioxys*)

*obscuriformis* (Quilis, 1931, *Trioxys*)

*amoplanus* (Quilis, 1934, *Trioxys*)

*mediterraneus* (Mackauer, 1960, *Trioxys*)

*wollastonii* (Cabrera, 1962, *Trioxys*) nom. nud.

*sikkimensis* (Raychaudhuri, Samanta, Pramanik, Tamili & Sarkar, 1990, *Trioxys*)

***brevicornis*** (Haliday, 1833, *Aphidius*) W I

*minutus* (Haliday, 1833, *Aphidius*)

***centaureae*** (Haliday, 1833, *Aphidius*) E S W I

*crudelis* (Rondani, 1848, *Misaphidus*)

*orientalis* (Starý & Schlinger, 1967, *Trioxys*)

*uroleucon* Takada & Rishi, 1980

***heraclei*** (Haliday, 1833, *Aphidius*) W

*obsoletus* (Wesmael, 1835, *Aphidius*)

*variegator* (Szépligeti, 1898, *Trioxys*)

***letifer*** (Haliday, 1833, *Aphidius*) I

***Diaeretellus*** Starý, 1960

***ephippium*** (Haliday, 1834, *Aphidius*) E I

***Diaeretiella*** Starý, 1960

***rapae*** (McIntosh, 1855, *Aphidius*) E W

*vulgaris* (Bouché, 1834, *Aphidius*)

*rapae* (Curtis, 1860, *Aphidius*) preocc.

*chenopodii* (Förster, 1867, *Diaeretus*) nom. nud.

*halticae* (Rondani, 1877, *Misaphidus*)

*piceus* (Cresson, 1879, *Trioxys*)

*chenopodiaphidis* (Ashmead, 1889, *Lipolexis*)

*ferruginipes* (Ashmead, 1890, *Diaeretus*) nom. nud.

*brassicae* (Marshall, 1896, *Aphidius*)

***Diaeretus*** Förster, 1863

***leucopterus*** (Haliday, 1834, *Aphidius*) E W

*exspectatus* (Gautier & Bonnamour, 1936, *Aphidius*)

***Harkeria*** Cameron, 1900

*Paramonoctonus* Starý, 1959

***rufa*** Cameron, 1900 E^^[[65]](#footnote-66)^^

***LIPOLEXIS*** Förster, 1863

*GYNOCRYPTUS* Quilis, 1931

***gracilis*** Förster, 1863 E BMNH, det. Torrance & Broad, added here

*palpator* (Gautier & Bonnamour, 1931, *Aphidius*)

*pieltaini* (Quilis, 1931, *Gynocryptus*)

*chinensis* Chen, 1980

***Lysaphidus*** Smith, 1944

***schimitscheki*** Starý, 1960 E

***Lysiphlebus*** Förster, 1863

Subgenus ***Lysiphlebus*** Förster, 1863

*Platycyphus* Mackauer, 1960

***dissolutus*** (Nees, 1811, *Bracon*) E

*macrocornis* Mackauer, 1960

Subgenus ***phlebus*** Starý, 1975

*Aphidaria* Provancher, 1888 preocc.

***confusus*** Tremblay & Eady, 1978 E W BMNH, added here^^[[66]](#footnote-67)^^

***fabarum*** (Marshall, 1896, *Aphidius*) E W

*aphidiperda* (Rondani, 1877, *Misaphidus*)

*monilicornis* (Thomson, 1895, *Aphidius*)

*cardui* (Marshall, 1896, *Aphidius*)

*aurantii* (Pierantoni, 1907, *Aphidius*)

*polygoni* (Ivanov, 1927, *Aphidius*) preocc.

*gomezi* (Quilis, 1930, *Aphidius*)

*janinii* (Quilis, 1930, *Aphidius*)

*inermis* Quilis, 1931

*innovatus* Quilis, 1931

*moroderi* Quilis, 1931

*ivanovi* Mackauer, 1967

***MONOCTONIA*** Starý, 1962

***vesicarii*** Tremblay, 1991 E added by Rakhshani *et al.* (2015)

***Monoctonus*** Haliday, 1833

Subgenus ***Falciconus*** Mackauer, 1961

***pseudoplatani*** (Marshall, 1896, *Aphidius*) E W

Subgenus ***Monoctonus*** Haliday, 1833

***caricis*** (Haliday, 1833, *Aphidius*) E W

***cerasi*** (Marshall, 1896, *Aphidius*) E

***crepidis*** (Haliday, 1834, *Aphidius*) E W

*tuberculatus* (Wesmael, 1835, *Aphidius*)

*paludum* Marshall, 1896

***ligustri*** van Achterberg, 1989 E W added by van Achterberg (1989)

***nervosus*** (Haliday, 1833, *Aphidius*)

*paulensis* (Ashmead, 1902, *Aphidius*)

*secundus* Viereck, 1915

*biroi* Györfi, 1958

*breviantennalis* Starý, 1959

***Paralipsis*** Förster, 1863

*Myrmecobosca* Maneval, 1940

***enervis*** (Nees, 1834, *Aphidius*) E W

*mandibularis* (Maneval, 1940, *Myrmecobosca*)

*linnei* (Hincks, 1949, *Myrmecobosca*)

***Pauesia*** Quilis Pérez, 1931

Subgenus ***Paraphidius*** Starý,1958

***abietis*** (Marshall, 1896, *Aphidius*) E

***cupressobii*** (Starý, 1960, *Paraphidius*) S BMNH, det. Gärdenfors, added here

***juniperorum*** (Starý, 1960, *Paraphidius*) E S

***pini*** (Haliday, 1834, *Aphidius*) E S W I

*planistipes* (Nees,1834, *Aphidius*)

*varia* (Nees, 1834, *Aphidius*)

*panzerii* (Rondani, 1848, *Aphidius*)

*lachnivorus* (Ashmead, 1906, *Aphidius*)

[?***silvestris*** (Starý, 1960, *Paraphidius*) added by Enobakhare (2001)^^[[67]](#footnote-68)^^]

Subgenus ***Pauesia*** Quilis Pérez, 1931

***infulata*** (Haliday, 1834, *Aphidius*) S

*albiflagellaris* (Starý, 1960, *Paraphidius*)

***laricis*** (Haliday, 1834, *Aphidius*) W

***picta*** (Haliday, 1834, *Aphidius*) W

***unilachni*** (Gahan, 1926, *Aphidius*) E W added by Belshaw & Quicke (1997)

*albuferensis* Quilis, 1931

*praevisus* (Gautier & Bonnamour, 1936, *Aphidius*)

*basilewskyi* (Benoit, 1955, *Trioxys*)

***Trioxys*** Haliday, 1833

Subgenus ***Pectoxys*** Mackauer, 1960

***macroceratus*** Mackauer, 1960

Subgenus ***Trioxys*** Haliday, 1833

*Aphidileo* Rondani, 1877

*NEVROPENES* Provancher, 1886

*BIOXYS* Starý & Schlinger, 1967

***auctus*** (Haliday, 1833, *Aphidius*) I

*flaviceps* Szépligeti, 1898

***betulae*** Marshall, 1896 E W

*solani* Ivanov, 1925

*hincksi* Mackauer, 1960

***cirsii*** (Curtis, 1831, *Aphidius*) E W I

*aceris* (Haliday, 1833, *Aphidius*)

***curvicaudus*** Mackauer, 1967 E W added by Starý (1978)

***falcatus*** Mackauer, 1959 W added by Baker & Broad (2009)

***ibis*** Mackauer, 1961

***pallidus*** (Haliday, 1833, *Aphidius*) E W

*resolutus* (Nees, 1834, *Aphidius*)

*callipteri* (Marshall, 1896, *Aphidius*)

*pulcher* Gautier & Bonnamour, 1924

***tenuicaudus*** Starý, 1978 W added by Baker & Broad (2013)

Tribe EPHEDRINI Mackauer, 1961

***Ephedrus*** Haliday, 1833^^[[68]](#footnote-69)^^

Subgenus ***BREVIEPHEDRUS*** Gärdenfors,1986

***brevis*** Stelfox, 1941 E S I

*picticornis* Stelfox, 1941

*niger* Stelfox, 1941 nom. nud.

Subgenus ***Ephedrus*** Haliday, 1833

*Elassus* Wesmael, 1835

***cerasicola*** Starý, 1962 E added by Van Veen *et al.* (2008)

***helleni*** Mackauer, 1968 S added by Baker & Broad (2013)

*salicicola* Takada, 1968

***lacertosus*** (Haliday, 1833, *Aphidius*) E S I

*muesebecki* Smith, 1944

***laevicollis*** (Thomson, 1895, *Aphidius*) W I

*brevicornis* (Nees, 1834, *Aphidius*) preocc.

*minor* Stelfox, 1941

*salicicola* Takada, 1968

***niger*** Gautier, Bonnamour & Gaumont, 1929 E

added by Van Veen *et al.* (2008)

?*aphidivora* (Rondani, 1848, *Alysia*) Papp (1996*a*)

*campestris* Starý, 1962

***persicae*** Froggat, 1904 E I

*nevadensis* Baker, 1909

*nitidus* Gahan, 1917

*vidali* Quilis, 1931

*interstitialis* Watanabe, 1941

*pulchellus* Stelfox, 1941

*impressus* Granger, 1949

*holmani* Starý, 1958

*palaestinensis* Mackauer, 1959

***plagiator*** (Nees, 1811, *Bracon*) E S W I

*parcicornis* (Nees, 1834, *Aphidius*)

*japonicus* Ashmead, 1906

*homostigma* Fahringer, 1934

Subgenus ***Lysephedrus*** Starý, 1958

***validus*** (Haliday, 1833, *Aphidius*) E I

***Toxares*** Haliday, 1840

*TRIONYX* Haliday, 1833 preocc.

*TERONYX* Haldeman, 1842

***deltiger*** (Haliday, 1833, *Aphidius*) E I

*flaveolus* (Györfi, 1958, *Ephedrus*)

Tribe PRAINI Mackauer, 1961

***Areopraon*** Mackauer, 1959

*MESOPRAON* Starý, 1981 Tomanović *et al.* (2006)

***lepelleyi*** (Waterston, 1926, *Praon*) E

***silvestre*** (Starý, 1971, *Praon*)^^[[69]](#footnote-70)^^ W added by Baker & Broad (2013)

***Dyscritulus*** Hincks, 1943^^[[70]](#footnote-71)^^

*Dyscritus* Marshall, 1896 preocc.

***planiceps*** (Marshall, 1896, *Dyscritus*) E W

***pygmaeus*** Mackauer, 1961

***Praon*** Haliday, 1833

*ACHORISTUS* Ratzeburg, 1852

*APHIDARIA* Provancher, 1886

*Parapraon* Starý, 1983

***abjectum*** (Haliday, 1833, *Aphidius*) E I

*aphidiiforme* (Ratzeburg, 1852, *Bracon*)

*peregrinum* Ruthe, 1859

***absinthii*** Bignell, 1894^^[[71]](#footnote-72)^^

***barbatum*** Mackauer, 1967 E BMNH, det. Torrance & Broad, added here

***bicolor*** Mackauer, 1959 W added by Baker & Broad (2009)

***cavariellae*** Starý, 1971 S W added by Baker & Broad (2013)

***dorsale*** (Haliday, 1833, *Aphidius*) E W I

*discolor* (Nees, 1834, *Blacus*)

*collare* Förster, 1867 nom. nud.

***exsoletum*** (Nees, 1811, *Bracon*)

*palitans* Muesebeck, 1956

***flavinode*** (Haliday, 1833, *Aphidius*) E W

*emacerator* (Nees, 1834, *Blacus*)

*glabrum* Starý & Schlinger, 1967

***gallicum*** Starý, 1971 E added by Traugott *et al.* (2008)

***longicorne*** (Marshall, 1896, *Aphidius*) W

*grossum* Starý, 1971

***necans*** Mackauer, 1959 W added by Baker & Broad (2013)

*nympheae* Subba Rao, Sarup & Sharma, 1963

***spinosum*** Mackauer, 1959 W added by Baker & Broad (2009)

***volucre*** (Haliday, 1833, *Aphidius*) E W I

*angulator* (Nees, 1834, *Blacus*)

*aphidivorum* (Ratzeburg, 1844, *Aphidius*)

*pruni* Ivanov, 1925

*breve* Fahringer, 1935

*mongolicum* Watanabe, 1949

*myzophagum* Mackauer, 1959

***yomenae*** Takada, 1968 W added by Baker & Broad (2013)

species excluded from the British and Irish list

[***simulans*** (Provancher, 1886, *Aphidaria*)^^[[72]](#footnote-73)^^

*aguti* Smith, 1944]

Subfamily Brachistinae Förster, 1863^^[[73]](#footnote-74)^^

CALYPTINAE Marshall, 1872

Tribe BLACINI Förster, 1863^^[[74]](#footnote-75)^^

***BLACOMETEORUS*** Tobias, 1976

***brevicauda*** (Hellén, 1958, *Diospilus*) E added by van Achterberg (1988*a*); Shaw (1996*b*)

***pusillus*** (Hellén, 1958, *Diospilus*) E added by Shaw (1996*b*)

***Blacus*** Nees, 1818

Subgenus ***Blacus*** Nees, 1818

*GONIOCORMUS* Förster, 1863

*MIOCOLUS* Förster, 1863

***errans*** (Nees, 1811, *Bracon*) E

*vagans* Ruthe, 1861

***exilis*** (Nees, 1811, *Bracon*) E I

*lactucaphis* (Fitch, 1855, *Aphidius*)

*pallipes* (Förster, 1863, *Miocolus*)

*pallidipes* (Dalla Torre, 1898, *Miocolus*)

*nanus* Ashmead, 1905 nom. nud.

*propallipes* Shenefelt, 1969

*intermedius* Janzon, 1975

***filicornis*** Haeselbarth, 1973 E I

***forticornis*** Haeselbarth, 1973 E

***hastatus*** Haliday, 1835 E S I

*terebrator* Ruthe, 1861

***humilis*** (Nees, 1811, *Bracon*) E W I

*trivialis* Haliday, 1835

*wesmaeli* Ruthe, 1861

***instabilis*** Ruthe, 1861 E S I

*petiolatus* Haeselbarth, 1973 nom. nud.

***leptostigma*** Ruthe, 1861 I

***longipennis*** (Gravenhorst, 1809, *Ophion*) E S I

*dubius* Ruthe, 1861

***nigricornis*** Haeselbarth, 1973 E S I

***paganus*** Haliday, 1835 I

*brevicornis* Ruthe, 1861

***stelfoxi*** Haeselbarth, 1973 E^^[[75]](#footnote-76)^^

Subgenus ***GANYCHORUS*** Haliday, 1835

***ambulans*** Haliday, 1835 I

***armatulus*** Ruthe, 1861 E

***diversicornis*** Nees, 1834 E I

*compar* Ruthe, 1861

***macropterus*** Haeselbarth, 1973 E S^^[[76]](#footnote-77)^^

***maculipes*** Wesmael, 1835 E S I

***nitidus*** Haeselbarth, 1973 E

*petiolatus* Tobias, 1976

***pallipes*** Haliday, 1835^^[[77]](#footnote-78)^^ E I

*barynoti* misident.

*tuberculatus* Wesmael, 1835

*florus* Goureau, 1851

***ruficornis*** (Nees, 1811, *Bracon*) E S W I

*bisstigmatus* (Say, 1836, *Microgaster*)

*tipulator* (Zetterstedt, 1838, *Bracon*)

*cerealis* (Curtis, 1860, *Dacnusa*)

*pallidipes* (Costa, 1885, *Dinocampus*)

*dentatus* Hellén, 1958

***strictus*** Stelfox, 1941 S I

*strictus* (Curtis, 1837, *Ganychorus*) nom. nud.

***tripudians*** Haliday, 1835 E I

Subgenus ***HYSTEROBOLUS*** Viereck, 1913

***mamillanus*** Ruthe, 1861 E

*aptenodytes* Marshall, 1889

Tribe BrachistinI Förster, 1863

***Eubazus*** Nees, 1814

Subgenus ***Aliolus*** Say, 1836

***lepidus*** (Haliday, 1835, *Helcon*) E

*hofferi* (Šnoflák, 1953, *Triaspis*) van Achterberg (2003*c*)

*kusarensis* (Abdinbekova, 1969, *Allodorus*)

van Achterberg (2003*c*)

Subgenus ***Allodorus*** Förster, 1863

***convexope*** van Achterberg, 2000 S added by van Achterberg (2000)

***semirugosus*** (Nees, 1816, *Sigalphus*) E S

*rufipes* (Herrich-Schäffer, 1838, *Eubadizon*)

*tuberculator* (Zetterstedt, 1838, *Bracon*)

*curculionum* (Hartig, 1847, *Sigalphus*)

*atricornis* (Ratzeburg, 1848, *Brachistes*)

*mucronatus* (Thomson, 1892, *Calyptus*)

*truncatus* (Thomson, 1892, *Calyptus*)

*glabratus* (Fahringer, 1941, *Calyptus*)

*arete* (Fahringer, 1944, *Calyptus*)

***tricoloripes*** van Achterberg, 2000 E added by van Achterberg (2000)

Subgenus ***Brachistes*** Wesmael, 1835

***fasciatus*** (Nees, 1816, *Sigalphus*) E I

*fuscipalpis* (Wesmael, 1835, *Brachistes*)

***minutus*** (Ratzeburg, 1848, *Brachistes*) E

***ruficoxis*** (Wesmael, 1835, *Brachistes*) E

*politus* (Ratzeburg, 1852, *Brachistes*) van Achterberg in Belokobylskij *et al.* (2003)

*byctisci* (Watanabe, 1933, *Calyptus*)

*ruficornis* misspelling

***segmentatus*** (Marshall, 1889, *Calyptus*) E

***semicastaneus*** (Marshall, 1893, *Calyptus*) E

***tibialis*** (Haliday, 1835, *Helcon*) E S I

*uncigenus* (Wesmael, 1835, *Brachistes*)

Subgenus ***CALYPTUS*** Haliday, 1835

***macrocephalus*** Nees, 1812 E

*synchitae* (Hedqvist, 1956, *Eubadizon*)

*ratzeburgi* (Fischer, 1962, *Eubadizon*) van Achterberg in Belokobylskij *et al.* (2003)

*xiphydriae* Tobias, 1986 van Achterberg in Belokobylskij *et al.* (2003)

***sigalphoides*** (Marshall, 1889, *Calyptus*) E

Subgenus ***Eubazus*** Nees, 1814

*Eubadizon* Nees, 1834

*Eubadizus* Nees, 1834

***flavipes*** (Haliday, 1835, *Helcon*) E I

*laevis* (Herrich-Schäffer, 1838, *Eubadizon*)

***longicauda*** (Curtis, 1832, *Zele*)^^[[78]](#footnote-79)^^ E

***pallipes*** Nees, 1814 E

*coxalis* (Nees, 1834, *Eubadizon*)

*semistriatus* (Haliday, 1835, *Helcon*) unavailable

*americanus* (Cresson, 1872, *Eubadizon*)

*pallipede* misspelling

*pallidipes* misspelling

*pollipes* misspelling

***Foersteria*** Szépligeti, 1896

***laeviuscula*** Szépligeti, 1896 E S NMS, det. van Achterberg, added here

***puber*** (Haliday, 1835, *Helcon*) E I

*opaca* (Reinhard, 1867, *Calyptus*)

*flavipes* Szépligeti, 1896

*talitzkii* Tobias, 1961

***Schizoprymnus*** Förster, 1863

***ambiguus*** (Nees, 1816, *Sigalphus*) E I

***collaris*** (Thomson, 1874, *Sigalphus*)^^[[79]](#footnote-80)^^ E added by Notton *et al.* (2014)

***obscurus*** (Nees, 1816, *Sigalphus*) E

species excluded from the British and Irish list

[***nigripes*** (Thomson, 1892, *Sigalphus*)^^[[80]](#footnote-81)^^]

***Triaspis*** Haliday, 1838

*MUIRIELLA* Fullaway, 1919

***aciculata*** (Ratzeburg, 1848, *Sigalphus*)^^[[81]](#footnote-82)^^ E BMNH, NMS, det. van Achterberg, added here

***caledonica*** (Marshall, 1888, *Sigalphus*) S

***caudata*** (Nees, 1816, *Sigalphus*) E I

*gracilis* (Herrich-Schäffer, 1840, *Sigalphus*)

*australis* (Szépligeti, 1901, *Sigalphus*)

*arctica* Hellén, 1958

***flavipalpis*** (Wesmael, 1835, *Sigalphus*) E

***floricola*** (Wesmael, 1835, *Sigalphus*) E I

*minima* Snoflák, 1953

***luteipes*** (Thomson, 1874, *Sigalphus*) E

***obscurella*** (Nees, 1816, *Sigalphus*) E

*simulator* (Szépligeti, 1901, *Sigalphus*)

***pallipes*** (Nees, 1816, *Sigalphus*) E I M Shaw & Askew (1976)

*fulvipes* (Haliday, 1835, *Helcon*)

*fagi* (Ratzeburg, 1852, *Brachistes*)

*similis* (Szépligeti, 1901, *Sigalphus*) preocc.

*pallidipes* misspelling

***podlussanyi*** Papp, 1998 E added by Shaw & Mendel (in prep.)

***striatula*** (Nees, 1816, *Sigalphus*) E I

***thoracica*** (Curtis, 1860, *Sigalphus*) E

*gibberosa* (Szépligeti, 1901, *Sigalphus*) Papp (2004*a*)

*rugosa* (Szépligeti, 1901, *Sigalphus*) Papp (2004*a*)

species excluded from the British and Irish list

[***striola*** (Thomson, 1874, *Sigalphus*)^^[[82]](#footnote-83)^^]

Tribe DIOSPILINI Förster, 1863^^[[83]](#footnote-84)^^

***AspiCOLPUS*** Wesmael, 1838

*ASPIDOCOLPUS* Agassiz, 1846

**?*clipealis*** (Tobias, 1967, *Aspidocolpus*) E NMS, det. van Achterberg, added here^^[[84]](#footnote-85)^^

***Aspigonus*** Wesmael, 1835

*ASPIDOGONUS* Agassiz, 1846

***flavicornis*** (Nees, 1834, *Bracon*) E W

*diversicornis* Wesmael, 1835 van Achterberg in Belokobylskij *et al.* (2003)

***Diospilus*** Haliday, 1833

*Baeacis* Förster, 1878 van Achterberg (2014)

*ALLOCHROMUS* Marshall, 1902

***abietis*** (Ratzeburg, 1844, *Aspigonus*)

***capito*** (Nees, 1834, *Bracon*) E I

*filator* (Nees, 1834, *Bracon*) preocc.

*fuscipes* (Wesmael, 1835, *Taphaeus*)

***dispar*** (Nees, 1811, *Bracon*) E

*ephippium* (Nees, 1834, *Bracon*)

***inflexus*** Reinhard, 1862 E

*ovatus* Marshall, 1889

***intermedius*** (Förster, 1878, *Baeacis*) E BMNH, det. van Achterberg, added here

***morosus*** Reinhard, 1862 E

***nigricornis*** (Wesmael, 1835, *Taphaeus*)^^[[85]](#footnote-86)^^ E

*affinis* (Wesmael, 1835, *Taphaeus*)

*rufipes* Reinhard, 1862

***oleraceus*** Haliday, 1833 E I

*conformis* (Wesmael, 1835, *Taphaeus*)

*ruficornis* Szépligeti, 1896

***productus*** Marshall, 1894 E

***Taphaeus*** Wesmael, 1835^^[[86]](#footnote-87)^^

*Anostenus* Förster, 1863

*Anastenus* Dalla Torre, 1898

***hiator*** (Thunberg, 1824, *Ichneumon*) E S I

*irregularis* Wesmael, 1835

*speculator* (Haliday, 1835, *Helcon*)

*polydrusi* (Gahan, 1916, *Diospilus*)

***VADUMASONIUM*** Kammerer, 2006

*VADUM* Mason, 1987 preocc.

***vardyorum*** van Achterberg & Broad, 2013 E added by van Achterberg & Broad (2013)

Tribe DYSCOLETINI van Achterberg, 1984^^[[87]](#footnote-88)^^

***Dyscoletes*** Haliday, 1840

*DYSCOLUS* Haliday, 1836 preocc.

*DISCOLUS* misspelling

*MICROCENTRUS* Szépligeti, 1904 preocc.

*ELACHISTOCENTRUM* Schulz, 1911

***lancifer*** (Haliday, 1836, *Dyscolus*) E S

*similis* (Szépligeti, 1896, *Discoletes*)

Subfamily BRACONINAE Nees, 1812^^[[88]](#footnote-89)^^

Tribe APHRASTOBRACONINI Ashmead, 1900

***PSEUDOVIPIO*** Szépligeti, 1896

***guttiventris*** (Thomson, 1892, *Bracon*)^^[[89]](#footnote-90)^^ E

*variegatus* (Boheman, 1853, *Agathis*) preocc.

*biroi* Szépligeti, 1896

Tribe BRACONINI Nees, 1812

***Baryproctus*** Ashmead, 1900

*Barycryptus* Hoffmeyer, 1932

***barypus*** (Marshall, 1885, *Bracon*) E^^[[90]](#footnote-91)^^

*hungaricus* Szépligeti, 1901

*caucasicus* Telenga, 1936

*apti* Györfi, 1953

***Bracon*** Fabricius, 1804^^[[91]](#footnote-92)^^

Subgenus ***BRACON*** Fabricius, 1804 *BRACO* Wesmael, 1838

*Brachon* Agassiz, 1846

*Tropidobracon* Ashmead, 1900

*MICROBRACON* Ashmead, 1890

*AMICOPLIDEA* Ashmead, 1900

*MACRODYCTIUM* Ashmead, 1900

*LIOBRACON* Nason, 1905 preocc.

*Lorenzoa* de Stefani-Perez, 1909

*Seliodus* Brèthes, 1909

*Kulczynskia* Niezabitowski, 1910

*Brazon* Schulz, 1911

*STRIOBRACON* Fahringer, 1927

*Chivinia* Shestakov, 1932

*EUTROPOBRACON* Ayyar, 1928

***alutaceus*** Szépligeti, 1901 E S NMS, det. Papp, added here

*pygmaeus* Niezabitowski, 1910 Papp (2008*b*)

*polonicus* Fahringer, 1927 Papp (2008*b*)

*pallidalatus* Tobias, 1957

***flavipes*** Nees, 1834 E

***fulvipes*** Nees, 1834 E S W I M

*carinatus* Szépligeti, 1901

*marshalli* Vayssière, 1902 preocc.

*glabratus* Fahringer, 1927

*apionis* Strand, 1928

*sylvanus* Greese, 1928

*kiritshenkoi* Telenga, 1936

***intercessor*** Nees, 1834 E S

*laetus* (Wesmael, 1838, *Braco*)^^[[92]](#footnote-93)^^

*lativentris* Thomson, 1892 preocc.

*fulvus* Szépligeti, 1896

*universitatis* Dalla Torre, 1898

*adjectus* Szépligeti, 1901

*bisinuatus* Szépligeti, 1901

*dubiosus* Szépligeti, 1901

*duplicatus* Szépligeti, 1901

*fallaciosus* Szépligeti, 1901

*elegans* Szépligeti, 1901

*mixtus* Szépligeti, 1901

*mundus* Szépligeti, 1901

*nigropictus* Szépligeti, 1901

*nitidiusculus* Szépligeti, 1901

*rufiscapus* Szépligeti, 1901

*subtilis* Szépligeti, 1901

*suspectus* Szépligeti, 1901

*vigilax* Kokujev, 1912

*maidli* Fahringer, 1925

*asiaticus* Fahringer, 1927 preocc.

*concolor* Fahringer, 1927 preocc.

*major* Fahringer, 1927

*concorellus* Strand, 1928

*megasomides* Strand, 1928

*rhynchiti* Greese, 1928

*kachetinus* Telenga, 1933

*kansensis* Fahringer, 1934 preocc.

*maslovskii* Telenga, 1936

*segregatus* Telenga, 1936

***leptus*** Marshall, 1897 E^^[[93]](#footnote-94)^^

*centaureae* Szépligeti, 1901

*rufipedator* Szépligeti, 1901

*rufipalpis* Szépligeti, 1901]

***longicollis*** (Wesmael, 1838, *Braco*) E S W M Papp (1999*a*)

*fraudator* Marshall, 1885

*brevicauda* Thomson, 1892 preocc.

*crassicauda* Thomson, 1892

*pseudowesmaeli* Strand, 1928

*wesmaeli* Fahringer, 1927 preocc.

***luteator*** Spinola, 1808 E added by Fulmek (1968)^^[[94]](#footnote-95)^^

*nigripedator* Nees, 1834

*filicauda* Costa, 1888

*hypopygialis* Szépligeti, 1901

*intermedius* Szépligeti, 1901

*pilosulus* Szépligeti, 1901

***nigratus*** (Wesmael, 1838, *Braco*) E I

***pectoralis*** (Wesmael, 1838, *Braco*) E

*ochrosus* Szépligeti, 1896

*sulphurator* Szépligeti, 1896

*unicolor* Szépligeti, 1896

*fumigatus* Szépligeti, 1901 Papp (2008*b*)

***rugulosus*** Szépligeti, 1901^^[[95]](#footnote-96)^^ E S W

*depressiusculus* Szépligeti, 1901

*neglectus* Szépligeti, 1904

*spurnensis* Hincks, 1951

***scutellaris*** (Wesmael, 1838, *Braco*) E S I

***speerschneideri*** Schmiedeknecht, 1897 E added by Papp (1999*b*)

***subrugosus*** Szépligeti, 1901 E added by Papp (2008*b*)

*sulcatulus* Szépligeti, 1896^^[[96]](#footnote-97)^^

*subglaber* Szépligeti, 1901

*quinquemaculatus* Szépligeti, 1901

*trypetanus* Fahringer, 1927 Papp (2008*b*)

*tauricus* Telenga, 1936

***trucidator*** Marshall, 1888 E^^[[97]](#footnote-98)^^ BMNH, NMS, added here

*minutator* misident.

*bilineatus* Thomson, 1892

*hilaris* Marshall, 1897

*pannonicus* Szépligeti, 1901

*marshalli* Telenga, 1936 preocc.

***variegator*** Spinola, 1808 E S I

*melanosoma* Szépligeti, 1901

*micros* Szépligeti, 1901

*nanulus* Szépligeti, 1901

*lineatellae* (Fischer, 1968, *Habrobracon*) Papp (2008*a*)

Subgenus ***GLABROBRACON*** Fahringer, 1927^^[[98]](#footnote-99)^^

***abbreviator*** Nees, 1834 E

*abscissor* Nees, 1834 Papp (2008*b*)

*oestmaeli* (Wesmael, 1838, *Braco*) Papp (2008*b*)

*regularis* (Wesmael, 1838, *Braco*) Papp (2008*b*)

*eutrephes* Marshall, 1897

*rufigaster* Szépligeti, 1901 Papp (2008*b*)

*rufiventris* Telenga, 1936 preocc.

*minimula* Strand, 1928

*minimus* Fahringer, 1927 preocc.

***admotus*** Papp, 2000 E BMNH, det. Papp, added here

***albion*** Papp, 1999 E S added by Papp (1999*a*)

***arcuatus*** Thomson, 1892 E S added by Papp (2000)^^[[99]](#footnote-100)^^

***atrator*** Nees, 1834 E S I

*longicauda* Thomson, 1892

***caudatus*** Ratzeburg, 1848 E

***claripennis*** Thomson, 1892 E added by Papp (2000)

***colpophorus*** (Wesmael, 1838, *Braco*) E

*mokrzeckii* Niezabitowski, 1927 Papp (1997)

***conjugellae*** Bengtsson, 1924 E S NMS, det. Papp, added here

*bengtssoni* Fahringer, 1928 preocc.

*minor* Fahringer, 1928 preocc.

*nanana* Strand, 1928

***curticaudis*** Szépligeti, 1901^^[[100]](#footnote-101)^^ S added by Papp (2008*b*)

***delibator*** Haliday, 1833 E S I

*anthracinus* Nees, 1834 van Achterberg (1997)

*breviseta* Fahringer, 1935

***fuscicoxis*** (Wesmael, 1838, *Braco*) E

*levicarinatus* Niezabitowski, 1910

***glaphyrus*** Marshall, 1897 E^^[[101]](#footnote-102)^^

***guttator*** Panzer, 1804 E^^[[102]](#footnote-103)^^

***immutator*** Nees, 1834 E S

*efoveolatus* Thomson, 1892

*hemirugosus* Szépligeti, 1901

*flavicoxanus* Strand, 1928

*marshalli* Fahringer, 1927 preocc.

*romani* Fahringer, 1927 preocc.

*uplandiae* Strand, 1928

*austriacus* Fahringer, 1936

*nigripalpis* Telenga, 1936

***instabilis*** Marshall, 1897 E

***kopelkei*** Papp, 2000 E BMNH, det. Papp, added here

***longulus*** Thomson, 1892 E S NMS, det. Papp, added here

***marshalli*** Szépligeti, 1901 E S^^[[103]](#footnote-104)^^ added by Papp (2000)

*obscurator* misident.

***minutator*** (Fabricius, 1798, *Ichneumon*) E I^^[[104]](#footnote-105)^^

*thalassinus* Schmiedeknecht, 1897 Papp (1999*b*)

*tener* Szépligeti, 1904

*abscissoris* Strand, 1928

*flavipalpula* Strand, 1928

*flavipalpis* Fahringer, 1928 preocc.

*rufiventris* Fahringer, 1928 preocc.

*notatus* Telenga, 1936

*unicolor* Telenga, 1936 preocc.

***momphae*** Papp, 1999 E S added by Papp (1999*a*)

***nigricollis*** (Wesmael, 1838, *Braco*) E NMS, det. Papp, added here

*brunneomaculatus* Schütze & Roman, 1931

***obscurator*** Nees, 1811 E S W

*kotulai* Niezabitowski, 1910

*nigripes* Fahringer, 1928 preocc.

*rytrensis* Fahringer, 1928 preocc.

*rytronis* Strand, 1928

*zaleszczykiensis* Strand, 1928

***otiosus*** Marshall, 1885^^[[105]](#footnote-106)^^ E

*macrurus* Thomson, 1892 Papp (1999*a*)

*explorator* Szépligeti, 1904 preocc. Papp (1999*a*)

*pumilionis* Roman, 1928 Papp (1999*a*)

***pallicarpus*** Thomson, 1892 E added by Papp (2000)

***parvicornis*** Thomson, 1892 E NMS, det. Papp, added here

*carbonarius* Szépligeti, 1901 preocc.

*aterrimus* Telenga, 1936 preocc.

***parvulus*** Wesmael, 1838 E S W NMS, det. Papp, added here

*fumipennis* Thomson, 1892

*fuscipennis* Thomson, 1892 preocc.

*thomsoni* Marshall, 1897

***pineti*** Thomson, 1892 E NMS, det. Papp, added here

***praecox*** (Wesmael, 1838, *Braco*) S NMS, det. Papp, added here

*biorrhizae* Fahringer, 1928

*elongatus* Dutu-Lacatusu, 1956 preocc.

***ratzeburgii*** Dalle Torre, 1898 E

*longicaudis* Ratzeburg, 1852 preocc.

***terebella*** (Wesmael, 1838, *Braco*) E

*breviterebris* Fahringer, 1928

*miaricola* Strand, 1928

*wesmaeli* Fahringer, 1928 preocc.

*unicolor* Telenga, 1936 preocc.

***variator*** Nees, 1811 E S W I

*bipartitus* (Wesmael, 1838, *Braco*)^^[[106]](#footnote-107)^^

*maculiger* (Wesmael, 1838, *Braco*)

*collinus* Szépligeti, 1901

*breviventris* Szépligeti, 1901

*rytrensis* Niezabitowski, 1910

*sueciensis* Fahringer, 1927

*dimidiatus* Fahringer, 1928 preocc.

*nigerrimus* Fahringer, 1928

*rytrensis* Fahringer, 1928 preocc.

*rytrocola* Strand, 1928

*chinensis* Fahringer, 1929 preocc.

*caucasicus* Telenga, 1936

*collaris* Telenga, 1936 preocc.

*meridionalis* Telenga, 1936

*ornatulus* Telenga, 1936

*rytrensis* Telenga, 1936 preocc.

*turcmenus* Telenga, 1936

*asiaticus* Telenga, 1949 preocc.

***xanthogaster*** Nees, 1834 E NMS, det. Papp, added here

*breviseta* Hedwig, 1961 preocc.

species excluded from the British and Irish list

[***coniferarum*** Fahringer, 1928^^[[107]](#footnote-108)^^]

Subgenus ***HABROBRACON*** Ashmead, 1895

***concolorans*** Marshall, 1900^^[[108]](#footnote-109)^^ E S I added by Papp (2008*a*)

*concolor* Thomson, 1892 preocc.

*nigricans* (Szépligeti, 1901, *Habrobracon*) Papp (2008*a*)

*mongolicus* (Telenga, 1936, *Habrobracon*)

***crassicornis*** Thomson, 1894 E S added by Papp (2008*a*)

*flavosignatus* (Tobias, 1957, *Habrobracon*)

Papp (2008*a*)

***hebetor*** Say, 1836 E^^[[109]](#footnote-110)^^

*dorsator* Say, 1836

*brevicornis* (Wesmael, 1838, *Braco*)^^[[110]](#footnote-111)^^

*juglandis* Ashmead, 1889

*brunneus* (Szépligeti, 1901, *Habrobracon*)

*vernalis* (Szépligeti, 1901, *Habrobracon*)

*beneficientior* (Viereck, 1911, *Habrobracon*)

*plotnicovi* (Bogoljubov, 1914, *Habrobracon*)

*breviantennatus* de Stefani, 1919

*serinopae* (Cherian, 1929, *Microbracon*)

*tortricidarum* (Goidanich, 1934, *Habrobracon*)

*pectinophorae* (Watanabe, 1935, *Habrobracon*)

*asiaticus* (Telenga, 1936, *Habrobracon*)

*flavus* (Telenga, 1936, *Habrobracon*)

*turkestanicus* (Telenga, 1936, *Habrobracon*)

*lozinskii* (Bogacev, 1939, *Habrobracon*)

***stabilis*** (Wesmael, 1838, *Braco*) E I

*opacus* Stelfox, 1953 preocc.

Subgenus ***LUCOBRACON*** Fahringer, 1927

***brachycerus*** Thomson, 1892 E NMS, det. Papp, added here

*kudsiricus* Papp, 1965

***crassungula*** Thomson, 1892 E NMS, BMNH, det. Papp, added here

**erraticus** (Wesmael, 1838, Braco) E S I M

superciliosus (Wesmael, 1838, Braco)

erythrostictus Marshall, 1885

*exarator* Marshall, 1885

*praetermissus* Marshall, 1885^^[[111]](#footnote-112)^^

*vectensis* Marshall, 1885 Papp (1999*a*)

*foveola* Thomson, 1892

*aestivalis* Szépligeti, 1901 Papp (2005*a*)

*confinis* Szépligeti, 1901

*congruus* Szépligeti, 1901 preocc.

*similis* Szépligeti, 1901

*ventricosus* Szépligeti, 1901

*secernendus* Schulz, 1906

*lagodechianus* Telenga, 1936

*maculatus* Telenga, 1936

*planiceps* Telenga, 1936

*talitzkii* Telenga, 1936

*transcaspicus* Telenga, 1936

*hades* Papp, 1965

***flagellaris*** Thomson, 1892 E BMNH, det. Papp, added here

*facialis* Thomson, 1892 preocc.

*thomsonii* Dalla Torre, 1898 preocc.

*dallatorrei* Szépligeti, 1901

***grandiceps*** Thomson, 1892 S NMS, BMNH, det. Papp, added here

*gallicus* Thomson, 1892

***guttiger*** (Wesmael, 1838, *Braco*) E S

*fasciatus* Fahringer, 1927

***hylobii*** Ratzeburg, 1848 E S

*bruchorum* Fahringer, 1934

***larvicida*** (Wesmael, 1838, *Braco*) E

*crassiusculus* Szépligeti, 1901

*romani* Fahringer, 1927 preocc.

*szepligetii* Fahringer, 1927 preocc.

*fahringeriensis* Strand, 1928

*pseudoromani* Strand, 1928

***nigriventris*** Wesmael, 1838, *Braco*) E NMS, det. Papp, added here

*subornatus* Szépligeti, 1901 Papp (2008*b*)

*biroi* Fahringer, 1927

*minor* Fahringer, 1927

*albanicus* Telenga, 1936

*laticeps* Telenga, 1936

*lencoranus* Telenga, 1936

*persimilis* Telenga, 1936

*turolus* Papp, 1984

***sphaerocephalus*** Szépligeti, 1901 E S NMS, det. Papp, added here

*globiceps* Szépligeti, 1901 Papp (2005*a*)

***thuringiacus*** Schmiedeknecht, 1897 E added by Papp (1999*b*)

*schmiedeknechti* Fahringer, 1927 preocc.

*blankenburgiae* Strand, 1928

***tornator*** Marshall, 1885 E S W Papp (1997)

*aequalis* Thomson, 1892

***triangularis*** Nees, 1834 E I

species excluded from the British and Irish list

[***strobilorum*** Ratzeburg, 1848^^[[112]](#footnote-113)^^]

Subgenus ***ORTHOBRACON***  Fahringer, 1927

***discoideus*** (Wesmael, 1838, *Braco*) E S I

*opionus* Fahringer, 1928

*sculpturatus* Fahringer, 1928

*sculpturifera* Strand, 1928

***epitriptus*** Marshall, 1885 E S W I Papp (1999*a*)

*pallidipes* Szépligeti, 1896 Papp (2008*b*)

*melanogaster* Szépligeti, 1901

***exhilarator*** Nees, 1834 E S W M

*satanas* (Wesmael, 1838, *Braco*)

*tibialis* Zetterstedt, 1838

*striolatus* Thomson, 1892

*marshalli* Fahringer, 1927 preocc,

*polonicus* Fahringer, 1927 preocc.

*polonicella* Strand, 1928

*varicoloris* Strand, 1928

***filicornis*** Thomson, 1892 S NMS, det. Papp, added here

***laevigatissimus*** Dalla Torre, 1898 E^^[[113]](#footnote-114)^^

*laevigatus* Ratzeburg, 1852 preocc.

***mediator*** Nees, 1834 E S I

***ochropus*** Nees, 1834 E S NMS, det. Papp, added here

*marshalli* Fahringer, 1927 preocc.

*neesi* Fahringer, 1927

*ochropodis* Strand, 1928

***orbus*** Papp, 1981 E NMS, det. Papp, added here

***picticornis*** Wesmael, 1838 E I

*gallarum* Ratzeburg, 1852

*versicolor* Szépligeti, 1901

*ratzeburgensis* Strand, 1928

*laevigatus* Fahringer, 1927 preocc.

***procerus*** Papp, 1965 E NMS, det. Papp, added here

***pulcher*** Bengtsson, 1924 E NMS, det. Papp, added here

*bengtssoni* Fahringer, 1927

***roberti*** (Wesmael, 1838, *Braco*) E I

***romani*** Fahringer, 1927 E S W I NMS, det. Papp, added here

***subcylindricus*** (Wesmael, 1838, *Braco*) E

*niger* (Vojnovskaja-Krieger, 1929, *Baryproctus*)

***subsinuatus*** Szépligeti, 1901^^[[114]](#footnote-115)^^ E S I added by Papp (2008*b*)

***titubans*** (Wesmael, 1838, *Braco*) E NMS, BMNH, det. Papp, added here

*tarsator* Thomson, 1892

*terebrator* Szépligeti, 1901

***virgatus*** Marshall, 1897 E S Shaw & Bailey (1991)

*lineifer* van Achterberg, 1988 Papp (1999*a*)

species excluded from the British and Irish list

[***tenuicornis*** (Wesmael, 1838, *Braco*)^^[[115]](#footnote-116)^^]

Subgenus **OSCULOBRACON** Papp, 2008

**osculator** Nees, 1811 E S W I

*bisignatus* (Wesmael, 1838, *Braco*)

*degenerator* Marshall, 1885

*minutus* Szépligeti, 1901

*temporalis* Telenga, 1936

*venustus* Telenga, 1936

Subgenus ***PIGERIA*** van Achterberg, 1985^^[[116]](#footnote-117)^^

***piger*** (Wesmael, 1838, *Braco*)^^[[117]](#footnote-118)^^ E I

*rotundatus* Szépligeti, 1901 Papp (2008*b*)

*rotundulus* Szépligeti, 1904

***wolschrijni*** (van Achterberg, 1985, *Pigeria*) W

NMS, det. van Achterberg, added here

species excluded from the British and Irish list

[Subgenus ***ROSTROBRACON*** Tobias, 1957

***urinator*** (Fabricius, 1798, *Ichneumon*)^^[[118]](#footnote-119)^^

*cuspidator* (Rossi, 1792, *Ichneumon*)

*comptus* Marshall, 1897]

Tribe COELOIDINI Tobias, 1957

***Coeloides*** Wesmael, 1838^^[[119]](#footnote-120)^^

*SYNTOMOMELUS* Kokujev, 1902

*HABROBRACONIDEA* Viereck, 1912

*COELOIDINA* Viereck, 1921

*CEROBRACON* Viereck, 1926

***abdominalis*** (Zetterstedt, 1838, *Bracon*) E

***filiformis*** Ratzeburg, 1852 E added by Shaw (2000*b*)

*melanurus* Ivanov, 1896

***melanostigma*** Strand, 1918 E added by Shaw (2000*b*)

*sordidator* misident.^^[[120]](#footnote-121)^^

*stigmaticus* Hellén, 1928

***melanotus*** Wesmael, 1838 E W

*flavus* Ivanov, 1896

*maculatus* Ivanov, 1896

***scolyticida*** Wesmael, 1838 E

*initiatellus* (Ratzeburg, 1848, *Bracon*)

species excluded from the British and Irish list by Shaw & Quicke (1999)

[***Atanycolus denigrator*** (Linnaeus, 1758, *Ichneumon*)

*incertus* (Sulzer, 1776, *Ichneumon*)

*heteropus* (Thomson, 1892, *Bracon*)

*albiscutis* Telenga, 1936]

[***Cyanopterus flavator*** (Fabricius, 1793, *Ichneumon*)

*flavulator* (Ratzeburg, 1844, *Bracon*)

*longipalpis* (Thomson, 1892, *Bracon*)

*barcinonensis* (Marshall, 1897, *Coeloides*)]

[***Vipio terrefactor*** (Villers, 1789, *Ichneumon*)^^[[121]](#footnote-122)^^

*improvisus* Kokujev, 1898

*interpellator* Kokujev, 1898

*neesii* Kokujev, 1898]

[Subfamily CARDIOCHILINAE Ashmead, 1900

***Cardiochiles*** Nees, 1819

*Ditherus* Cameron, 1902

***saltator*** (Fabricius, 1781, *Ichneumon*) not British: Shaw & Huddleston (1991)

*brachialis* Rondani, 1877

*katkowi* Kokujev, 1895

*fumipennis* Szépligeti, 1901

*sibiricus* Telenga, 1955]

Subfamily Cenocoeliinae Szépligeti, 1901^^[[122]](#footnote-123)^^

***Cenocoelius*** Haliday, 1840

*Laccophrys* Förster, 1863

*Promachus* Cresson, 1887 preocc. van Achterberg (1995)

*Caenocoelius* Marshall,1894

*Postpromachus* Maes,1999

***aartseni*** (van Achterberg, 1994, *Promachus*) E

added by Shaw (1999*b*)

***analis*** (Nees, 1834, *Bracon*) E I

*flavifrons* Haliday, 1840

*cephalotes* (Ratzeburg, 1848, *Opius*) preocc.

*magdalini* (Förster, 1863, *Laccophrys*)

*hungaricus* Kiss, 1927

***LESTRICUS*** Reinhard, 1865

***secalis*** (Linnaeus, 1758, *Ichneumon*) S NMS, det. Shaw, added here^^[[123]](#footnote-124)^^

*agricolator* (Linnaeus, 1767, *Ichneumon*)

*rubriceps* (Ratzeburg, 1844, *Alysia*)

*femorator* (Tobias, 1973, *Cenocoelius*)

Subfamily CHARMONTINAE van Achterberg, 1979

***Charmon*** Haliday, 1833

*PROVANCHERIA* Ashmead, 1900

*CYCLOCORMUS* Cameron, 1911

*EUBADIZON* misident.

***cruentatus*** Haliday, 1833 E S W I M

*pectoralis* (Nees, 1834, *Eubadizon*)

*pleuralis* (Cresson, 1872, *Eubadizon*)

*luteus* (Cameron, 1911, *Cyclocormus*)

?*brevicauda* (Hellén, 1958, *Eubadizon*)^^[[124]](#footnote-125)^^

***extensor*** (Linnaeus, 1758, *Ichneumon*) E S I

*gracilis* (Provancher, 1880, *Eubadizon*)

*hungaricus* (Kiss, 1927, *Calyptus*)

*striatus* (Shestakov, 1940, *Eubadizon*)

Subfamily Cheloninae Förster, 1863

ADELIINAE Viereck, 1918^^[[125]](#footnote-126)^^

ACAELIINAE Viereck, 1918

ACOELIINAE Viereck, 1918

Tribe Chelonini Förster, 1863

***Ascogaster*** Wesmael, 1835^^[[126]](#footnote-127)^^

*CASCOGASTER* Baker, 1926

*Leptodrepana* Shaw, 1983 van Achterberg (1990)

***abdominator*** (Dahlbom, 1833, *Chelonus*) E S I

*instabilis* Wesmael, 1835

*fulviventris* Curtis, 1837

*femoralis* (Herrich-Schäffer, 1838, *Chelonus*)

*rufiventris* (Herrich-Schäffer, 1838, *Chelonus*)

*pallida* Ruthe, 1855

***albitarsus*** Reinhard, 1867 S I added by Huddleston (1984)

*similis* (Herrich-Schäffer, 1838, *Chelonus*)

*leptopus* Thomson, 1874

***annularis*** (Nees, 1816, *Sigalphus*) E

***armata*** Wesmael, 1835 E W

*pulchella* (Curtis, 1829, *Chelonus*) nom. nud.

*esenbeckii* Curtis, 1837

*luteicornis* (Herrich-Schäffer, 1838, *Chelonus*)

***bidentula*** Wesmael, 1835 E S W I added by Huddleston (1984)

*scabriuscula* (Zetterstedt, 1838, *Sigalphus*)

*multiarticulata* (Ratzeburg, 1852, *Chelonus*)

*gibbiscuta* Thomson, 1874

*fuscipennis* Thomson, 1892

*atamiensis* Ashmead, 1906

***brevicornis*** Wesmael, 1835 E I added by Huddleston (1984)

*monilicornis* (Herrich-Schäffer, 1838, *Chelonus*)

***canifrons*** Wesmael, 1835 E I

*graniger* Thomson, 1892

*zernyana* Fahringer, 1925

***consobrina*** Curtis, 1837 E S I

***dentifer*** Tobias, 1976 E added by Huddleston (1984)

***dispar*** Fahringer, 1934 E added by Huddleston (1984)

*spinifer* Tobias, 1964

*kozlovi* Tobias, 1972

***gonocephala*** Wesmael, 1835 E added by Huddleston (1984)

***grahami*** Huddleston, 1984 E added by Huddleston (1984)

***klugii*** (Nees, 1816, *Sigalphus*) E added by Huddleston (1984)

*ruficeps* Wesmael, 1835

*neesii* Reinhard, 1867

***quadridentata*** Wesmael, 1835 E

*pallidicornis* Curtis, 1837

*impressa* (Herrich-Schäffer, 1838, *Chelonus*)

*quadridens* (Herrich-Schäffer, 1838, *Chelonus*)

*cynipum* Thomson, 1892

*nigricornis* Thomson, 1892

*egregia* Kokujev, 1895

*nigrator* (Szépligeti, 1896, *Chelonus*)

*carpocapsae* (Viereck, 1909, *Chelonus*)

*epinotiae* Watanabe, 1937

***rufidens*** Wesmael, 1835 E I

*rufipes* (Herrich-Schäffer, 1838, *Chelonus*) preocc.

*laevigator* (Ratzeburg, 1852, *Chelonus*)

***rufipes*** (Latreille, 1809, *Sigalphus*) E I

*elegans* (Nees, 1816, *Sigalphus*)

*fasciata* (Dahlbom, 1833, *Chelonus*)

*pallipes* (Herrich-Schäffer, 1838, *Chelonus*)

*rubripes* (Lucas, 1849, *Chelonus*)

*rugosula* (Goureau, 1861, *Chelonus*)

*ratzeburgii* Marshall, 1885

*arisanica* Sonan, 1932

*nigribasis* Fahringer, 1934

*soror* Telenga, 1941

***varipes*** Wesmael, 1835 E S I

*atriceps* (Ratzeburg, 1844, *Chelonus*)

*tersa* Reinhard, 1867

*cavifrons* Thomson, 1874

*sternalis* Thomson, 1874

*catula* (Marshall, 1885, *Chelonus*) Papp (1996*b*)

*jaroslawensis* Kokujev, 1895

species excluded from the British and Irish list

[***bicarinata*** (Herrich-Schäffer, 1838, *Chelonus*)^^[[127]](#footnote-128)^^

*mlokossewitschi* Kokujev, 1895

*rufiventris* Telenga, 1941 preocc.]

[***similis*** (Nees, 1816, *Chelonus*)^^[[128]](#footnote-129)^^]

***Chelonus*** Jurine, 1801

Subgenus ***Chelonus*** Jurine, 1801

*Anomala* von Block, 1799 nom. ob.

*DAVISANIA* La Munyon, 1877

*ARICHELONUS* Viereck, 1913

*MEGACHELONUS* Baker, 1926

***acuminatus*** Herrich-Schäffer, 1838 E NMS, det. Huddleston, added here

***annulatus*** (Nees, 1816, *Sigalphus*) E S I

*maculatus* Szépligeti, 1896

***asiaticus*** Telenga, 1941^^[[129]](#footnote-130)^^ E I BMNH, det. Lozan, added here

***canescens*** Wesmael, 1835 E

***carbonator*** Marshall, 1885 E

*asiaticus* Fahringer, 1932

***corvulus*** Marshall, 1885 E S

*suturatus* Szépligeti, 1898

***cylindrus*** (Klug, 1816, *Sigalphus*) E

*variabilis* Herrich-Schäffer, 1838

*macrocerus* Thomson, 1874

*speculator* Marshall, 1885^^[[130]](#footnote-131)^^

*ebeninus* Fahringer, 1934

***decorus*** Marshall, 1885 E

*clavipes* Curtis, 1837 nom. nud.

*clavipes* Fahringer, 1934

*szepligetii* Fahringer, 1934

***inanitus*** (Linnaeus, 1767, *Cynips*) E I

*binarius* (Fourcroy, 1785, *Ichneumon*)

*atomos* (Rossi, 1790, *Ichneumon*)

***obscuratus*** Herrich-Schäffer, 1838 E NMS, det. Huddleston, added here

*intermedius* Thomson, 1874

***oculator*** (Fabricius, 1785, *Ichneumon*) E S

*integer* (von Block, 1799, *Anomala*)

*mutabilis* (Nees, 1816, *Sigalphus*)

*oculatus* (Nees, 1816, *Sigalphus*)

***pusio*** Marshall, 1885 E

***scabrator*** (Fabricius, 1793, *Ichneumon*) E

*scaber* (Nees, 1816, *Sigalphus*)

*buccatus* Thomson, 1874

***submuticus*** Wesmael, 1835 E

*luteipes* Thomson, 1874 Papp (1995*a*)

***wesmaelii*** Curtis, 1837 E

*zimini* Tobias, 1972

Subgenus ***Microchelonus*** Szépligeti, 1908^^[[131]](#footnote-132)^^

*CHELONELLA* Szépligeti, 1908

*NEOCHELONELLA* Hincks, 1943

***atripes*** Thomson, 1874 E NMS, BMNH, det. Huddleston and Lozan, added here

*cunctator* (Papp, 1971, *Microchelonus*)

*kamtshaticus* (Tobias, 1986, *Microchelonus*)

***basalis*** Curtis, 1837 E S

***binus*** (Tobias, 1995, *Microchelonus*) E BMNH, det. Lozan, added here

***contractus*** (Nees, 1816, *Sigalphus*) E S W I Tobias & Shaw (2005)

*compressiscapus* Szépligeti, 1898 Papp (1996*b*)

***depressus*** Thomson, 1874 E S W NMS, BMNH, det. Huddleston and Lozan, added here

***exilis*** Marshall, 1885 E

*excavatus* Tobias, 1972 Papp (1995*a*)

***fenestratus*** (Nees, 1816, *Sigalphus*)^^[[132]](#footnote-133)^^ E

*dispar* Marshall, 1885

*bimaculatus* Ivanov, 1899 preocc.

***fumipennis*** (Tobias, 1986, *Microchelonus*) BMNH, det. Lozan, added here^^[[133]](#footnote-134)^^

***latrunculus*** Marshall, 1885 E S

?*parcicornis* Herrich-Schäffer, 1838^^[[134]](#footnote-135)^^

*thomsonii* Dalla Torre, 1898

*polonicus* Fahringer, 1934

*rectus* (Papp, 1971, *Microchelonus*)

***lugubris*** Wesmael, 1835 E I NMS, BMNH, det. Huddleston and Lozan, added here

***microphtalmus*** Wesmael, 1838 E NMS, det. Huddleston, added here

*dilatus* Papp, 1971 Papp (1995*a*)

***miscellae*** (Tobias & Shaw, 2005, *Microchelonus*) E

added by Tobias & Shaw (2005)

***retusus*** (Nees, 1816, *Sigalphus*) E BMNH, det. Lozan, added here

*emarginatus* Herrich-Schäffer, 1838

*subemarginatus* Herrich-Schäffer, 1838

*caudatus* Thomson, 1874

*pamiricus* Vojnovskaja-Krieger, 1931

***risorius*** Reinhard, 1867 E

*fissus* Szépligeti, 1900 preocc.

*fissuralis* (Tobias, 1964, *Neochelonella*)

*magnifissus* (Tobias, 1986, *Microchelonus*)

***sulcatus*** Jurine, 1807 E

*rimulosus* Thomson, 1874

*curvisulcatus* Szépligeti, 1896

*rimatus* Szépligeti, 1896

Subgenus ***Parachelonus*** Tobias, 1995

***gravenhorstii*** (Nees, 1816, *Sigalphus*) E

*maculator* Dahlbom, 1833

*eurytheca* Wesmael, 1838

*adjaricus* Tobias, 1976

*tricolor* Tobias, 1976

***pellucens*** (Nees, 1816, *Sigalphus*) E added by Papp (2004*b*)

*nitens* Reinhard, 1867

*alboannulatus* Szépligeti, 1896

*pulchricornis* Szépligeti, 1898

*varimaculatus* Tobias, 1986

*austriacus* Fahringer, 1934

Subgenus ***Stylochelonus*** Hellén, 1958

***pedator*** Dahlbom, 1833 E S

*secutor* (Marshall, 1885, *Chelonus*)

***pusillus*** (Szépligeti, 1908, *Stylochelonus*) E BMNH, det. Lozan, added here

*furtivus* (Tobias, 1986, *Microchelonus*)

*tuberculiventris* (Tobias, 1986, *Microchelonus*)

Tribe PHANEROTOMINI Baker, 1926

***ADELIUS*** Haliday, 1833

*ACAELIUS* Haliday, 1834

*Acoelius* Haliday, 1835

*PLEIOMERUS* Wesmael, 1837

*ANOMOPTERUS* Rohwer, 1914

*Myriola* Shestakov, 1932

***erythronotus*** (Förster, 1851, *Acoelius*) E I

*pyrrhia* (Beirne, 1945, *Acoelius*)^^[[135]](#footnote-136)^^

*flavus* (Tobias, 1966, *Acoelius*)

***germanus*** (Haliday, 1834, *Acaelius*) E I

***subfasciatus*** Haliday, 1833 E W I Shaw & Askew (1976)

*minutissimus* (Zetterstedt, 1840, *Bracon*)

*parvulus* (Förster, 1851, *Acoelius*)

***viator*** (Förster, 1851, *Acoelius*) E

***Phanerotoma*** Wesmael, 1838

Subgenus ***Phanerotoma*** Wesmael, 1838

*Phanerogaster* Wesmael, 1838 unavailable

*Sulydus* Du Buysson, 1897

*Ichneutipterus* Vachal, 1907 van Achterberg (1990)

*Neophanerotoma* Szépligeti, 1908

*Neoacampsis* Szépligeti, 1914 van Achterberg (1990)

*Phanerotomina* Shestakov, 1930

***acuminata*** Szépligeti, 1908 E added by van Achterberg (1990)

***dentata*** (Panzer, 1805, *Chelonus*)^^[[136]](#footnote-137)^^ E

*dentator* (Nees, 1816, *Sigalphus*)

*rendilea* Fahringer, 1934

*minor* Šnoflák, 1951 van Achterberg (1990)

***leucobasis*** Kriechbaumer, 1894^^[[137]](#footnote-138)^^ E added by van Achterberg (1990)

*ocularis* Kohl, 1906

*ornatulopsis* de Saeger, 1942

*desertorum* Hedwig, 1957 van Achterberg (1990)

*flavitestacea* Fischer, 1939 van Achterberg (1990)

*caboverdensis* Hedqvist,1965

species excluded from the British and Irish list

[***planifrons*** (Nees, 1816, *Sigalphus*)^^[[138]](#footnote-139)^^

*blanda* Fahringer, 1934

*bicolor* Snoflák, 1958 preocc.

*asini* Llopis Minguez, 1968

*snoflaki* Shenefelt, 1973]

Subgenus ***Bracotritoma*** Csiki, 1909

*Tritoma* Szépligeti, 1908 preocc. van Achterberg (1990)

*Szepligetia* Schulz, 1911 van Achterberg (1990)

*Tritomios* Strand, 1921 van Achterberg (1990)

*Unica* Šnoflák, 1951

***bilinea*** Lyle, 1924 E

*gregori* Šnoflák, 1951

***tritoma*** (Marshall, 1898, *Chelonus*) E W I

*antennalis* Šnoflák, 1951 van Achterberg (1990)

Subfamily DORYCTINAE Förster, 1863^^[[139]](#footnote-140)^^

Tribe DORYCTINI Förster, 1863

***Caenopachys*** Förster, 1863^^[[140]](#footnote-141)^^

***hartigii*** (Ratzeburg, 1848, *Bracon*) E

*flaviventris* (Förster, 1878, *Dendrosoter*)

*hartigi* misspelling

***Dendrosoter*** Wesmael, 1838

*EURYBOLUS* Ratzeburg, 1848

***middendorffi*** (Ratzeburg, 1848, *Bracon*) E NMS, det. Shaw, added in Fauna Europaea

*schimitscheki* Fahringer, 1941

***protuberans*** (Nees, 1834, *Bracon*) E

*insignis* Förster, 1878

***Doryctes*** Haliday, 1836

*ISCHIOGONUS* Wesmael, 1838

*Neodoryctes* Szépligeti, 1914

*UDAMOLCUS* Enderlein, 1920

*PRISTODORYCTES* Kieffer, 1921

*Paradoryctes* Granger, 1949

*PLYCTES* Fischer, 1970

***heydenii*** Reinhard, 1865 E

**#*leucogaster*** (Nees, 1834, *Bracon*)^^[[141]](#footnote-142)^^

***obliteratus*** (Nees, 1834, *Bracon*)^^[[142]](#footnote-143)^^ E

*mutillator* misident.

*tabidus* (Haliday, 1836, *Rogas*)

*brachyurus* Marshall, 1888

*strigatus* Kokujev, 1900

*petrovskii* Kokujev, 1902

***pomarius*** Reinhard, 1865 E

*schimitscheki* Fahringer, 1931

***rossicus*** Telenga, 1941 E NMS, det. Shaw, added in Fauna Europaea

***striatellus*** (Nees, 1834, *Bracon*) E

*maculipes* Curtis, 1837 nom. nud.

*disparator* (Ratzeburg, 1844, *Bracon*)

*rex* Marshall, 1897

*striatelloides* Strand, 1918

*yogoi* Watanabe, 1954

*ambigua* Kokujev, 1900

*notatus* Kokujev, 1900

***undulatus*** (Ratzeburg, 1852, *Bracon*) E

***GILDORIA*** Hedqvist, 1974^^[[143]](#footnote-144)^^

***similis*** (Bouček, 1955, *Dendrosotinus*) E added by Shaw (1998*a*)

***Ontsira*** Cameron, 1900^^[[144]](#footnote-145)^^

*DORYCTODES* Hellén, 1927

***antica*** (Wollaston, 1858, *Clinocentrus*) S

*gallica* (Reinhard, 1865, *Doryctes*)

*truncorum* (Goureau, 1866, *Bracon*)

*incerta* (Ashmead, 1888, *Doryctes*)

*caudalis* (Hellén, 1957, *Oncophanes*)

***ignea*** (Ratzeburg, 1852, *Bracon*) E NMS, det. Shaw and van Achterberg, added here

***imperator*** (Haliday, 1836, *Rogas*) E I

*zonata* (Wesmael, 1838, *Ischiogonus*)

*praecisa* (Ratzeburg, 1852, *Bracon*)

*cingulata* (Provancher, 1880, *Syngaster*)

*dubia* (Kokujev, 1900, *Doryctes*)

*iranica* (Telenga, 1941, *Doryctodes*)

*niger* (Hedwig, 1957, *Coeloides*)

***RHACONOTUS*** Ruthe, 1854

*HEDYSOMUS* Förster, 1863

*HORMIOPTERUS* Giraud, 1869

*RHADINOGASTER* Szépligeti, 1908

*EURYPHRYMNUS* Cameron, 1910

*RHACONOTINUS* Hedqvist, 1965

***aciculatus*** Ruthe, 1854 E added by Shaw (1998*a*)

*cerdai* Docavo Alberti, 1960

*major* Tobias, 1964

***Wachsmannia*** Szépligeti, 1900^^[[145]](#footnote-146)^^

***spathiiformis*** (Ratzeburg, 1848, *Bracon*) E

*maculipennis* Szépligeti, 1900 van Achterberg (1995)

*obliteratus* misident.

Tribe ECPHYLINI Hellén, 1957

***Ecphylus*** Förster, 1863^^[[146]](#footnote-147)^^

*TERENUSA* Marshall, 1885

*PARAECPHYLUS* Ashmead, 1900

*SACTOPUS* Ashmead, 1900

*SYCOSOTER* Picard & Lichtenstein, 1917

***eccoptogastri*** (Ratzeburg, 1848, *Bracon*)

***hylesini*** (Ratzeburg, 1852, *Bracon*) E NMS, det. Shaw and van Achterberg, added in Fauna Europaea

***pinicola*** Hedqvist, 1967 E S NMS, det. Shaw and van Achterberg, added in Fauna Europaea

***silesiacus*** (Ratzeburg, 1848, *Bracon*) E^^[[147]](#footnote-148)^^ NMS, det. Shaw and van Achterberg, added in Fauna Europaea

*minutissimus* (Ratzeburg, 1848, *Bracon*) preocc.

Tribe HECABOLINI Förster, 1863

***Hecabolus*** Curtis, 1834

*ANISOPELMA* Wesmael, 1838

***sulcatus*** Curtis, 1834 E W

*belgicus* (Wesmael, 1838, *Anisopelma*)

Species excluded from the British and Irish list

[***Monolexis*** Förster, 1863

***fuscicornis*** Förster, 1863^^[[148]](#footnote-149)^^

*lycti* (Cresson, 1880, *Anisopelma*)

*minimus* (Cresson, 1880, *Anisopelma*)

*utilis* (Cresson, 1880, *Anisopelma*)

*doderoi* (Mantero, 1910, *Hecabolus*)

*lavagnei* Picard, 1913

*atis* Nixon, 1943]

Tribe HETEROSPILINI Fischer, 1981

***Heterospilus*** Haliday, 1836

*SYNODUS* misident.

*TELEBOLUS* Marshall, 1888

*KAREBA* Cameron, 1905

*ANOCATOSTIGMA* Enderlein, 1920

*HARPAGOLACCUS* Enderlein, 1920

*Lituania* Jakimavičius, 1968

***ater*** Fischer, 1960 E added by Shaw (1998*a*)

***fuscexilis*** Shaw, 1997 E added by Shaw (1997)

species excluded from the British and Irish list

[***caesus*** (Nees, 1834, *Bracon*) misident.^^[[149]](#footnote-150)^^]

Tribe SPATHIINI Förster, 1863

***Spathius*** Nees, 1818

*STENOPHASMUS* Smith, 1859

*EUSPATHIUS* Förster, 1863

*PSEUDOSPATHIUS* Szépligeti, 1902

*RHACOSPATHIUS* Cameron, 1905

***brevicaudis*** Ratzeburg, 1844 E W NMS, det. Shaw, added in Fauna Europaea

***exarator*** (Linnaeus, 1758, *Ichneumon*) E S W I

*formicatus* (Linnaeus, 1767, *Ichneumon*)

*mutillarius* (Fabricius, 1775, *Ichneumon*)

*mystacatus* (Schrank, 1781, *Ichneumon*)

*affinis* (Fabricius, 1793, *Ichneumon*)

*immaturus* (Gravenhorst, 1807, *Ichneumon*)

*clavatus* (Panzer, 1809, *Cryptus*)

*affinator* (Thunberg, 1824, *Ichneumon*)

*attenuator* (Thunberg, 1824, *Ichneumon*)

*formicator* (Thunberg, 1824, *Ichneumon*) preocc.

*mutillator* (Thunberg, 1824, *Ichneumon*)

*exannulatus* Ratzeburg, 1848

*ferrugatus* Goureau, 1866

*strandi* Fahringer, 1930

*breviterebrantus* Dutu-Lacatusu, 1956

***pedestris*** Wesmael, 1838 E^^[[150]](#footnote-151)^^

*apterus* Wollaston, 1858

*maderi* Fahringer, 1930

*hirtus* Hedqvist, 1976

***phymatodis*** Fischer, 1966 E NMS, det. Shaw, added in Fauna Europaea

***rubidus*** (Rossius, 1794, *Ichneumon*) E S I

*umbratus* (Fabricius, 1798, *Ichneumon*)

*umbrator* (Thunberg, 1824, *Ichneumon*) preocc,

*rugosus* Ratzeburg, 1848

*sculpturatus* Hellén, 1927

*depressus* Hedqvist, 1976

*bimaculatus* Telenga, 1941

***umbratus*** (Fabricius, 1798, Ichneumon) E

*erythrocephalus* Wesmael, 1838 van Achterberg (2014)

*curvicaudis* Ratzeburg, 1844^^[[151]](#footnote-152)^^

Subfamily Euphorinae Förster, 1863^^[[152]](#footnote-153)^^

Tribe CENTISTINI Čapek, 1970

***Allurus*** Förster, 1863

***lituratus*** (Haliday, 1835, *Leiophron*)^^[[153]](#footnote-154)^^ I

***muricatus*** (Haliday, 1833, *Ancylus*) S I

*armatus* (Wesmael, 1835, *Leiophron*)

*niger* (Lyle, 1926, *Leiophron*)

***Centistes*** Haliday, 1835

Subgenus ***Ancylocentrus*** Förster, 1863

***ater*** (Nees, 1834, *Leiophron*) E I

*excrucians* (Haliday, 1835, *Leiophron*)

*lativalvis* (Jakimavičius, 1972, *Allurus*)

***collaris*** (Thomson, 1895, *Leiophron*) E I BMNH, det. Mason, Broad, added here

***edentatus*** (Haliday, 1835, *Leiophron*) E I

***nasutus*** (Wesmael, 1838, *Eubadizon*) E Luff (1976*a*)

*saxo* (Reinhard, 1862, *Leiophron*)

species excluded from the British and Irish list

[***subsulcatus*** (Thomson, 1895, *Leiophron*)^^[[154]](#footnote-155)^^]

Subgenus ***Centistes*** Haliday, 1835

*aNCYLUS* Haliday, 1833

*aNCYLLUS* Haldeman, 1842

*EUPHORIDEA* Ashmead, 1900

*LIOSIGALPHUS* Ashmead, 1900

***cuspidatus*** (Haliday, 1833, *Ancylus*) E I

*lucidator* (Nees, 1834, *Bracon*)

***fuscipes*** (Nees, 1834, *Bracon*) E

*fuscipes* (Wesmael, 1835, *Leiophron*)

Subgenus ***Syrrhizus*** Förster, 1863

***delusorius*** (Förster, 1863, *Syrrhizus*) E S

Tribe COSMOPHORINI Muesebeck & Walkley, 1951

***COSMOPHORUS*** Ratzeburg, 1848

*COSMOPHORINUS* Viereck, 1925

***cembrae*** Ruschka, 1925 E added by Shaw (1989)

***Ropalophorus*** Curtis, 1837

*RHopalophorus* Blanchard, 1840 emendation, preocc.

*CORYNOPHORE* Blanchard, 1845

*EUSTALOCERUS* Förster, 1863

***clavicornis*** (Wesmael, 1835, *Microctonus*) E

*wisconsinensis* Shenefelt, 1960 Yang *et al.* (2003)

Tribe DINOCAMPINI Shaw, 1985

***dinocampus*** Förster, 1863

***coccinellae*** (Schrank, 1802, *Ichneumon*) E S W I

*terminatus* (Nees, 1812, *Bracon*)

*sculptus* (Cresson, 1872, *Euphorus*)

*americanus* (Riley, 1888, *Centistes*)

Tribe EUPHORINI Förster, 1863

***Leiophron*** Nees, 1818^^[[155]](#footnote-156)^^

Subgenus ***Euphorus*** Nees, 1834^^[[156]](#footnote-157)^^

***duploclaviventris*** Shenefelt, 1969 E

*claviventris* (Ruthe, 1856, *Microctonus*) preocc.

*ruthei* Loan, 1974

***pallidistigma*** (Curtis, 1833, *Leiophron*) E I

*pallicornis* (Nees, 1834, *Euphorus*

*claviventris* (Wesmael, 1835, *Microctonus*)

*intacta* (Haliday, 1835, *Leiophron*)

*parvula* (Ruthe, 1856, *Microctonus*)

***similis*** (Curtis, 1833, *Leiophron*)^^[[157]](#footnote-158)^^ E W I

*basalis* (Curtis, 1833, *Leiophron*)

Subgenus ***Leiophron*** Nees, 1818

*Liophron* Förster, 1863

***apicalis*** Haliday, 1833 E I M

*apicalis* Curtis, 1833 preocc.

*ornata* (Marshall, 1887, *Euphorus*)^^[[158]](#footnote-159)^^

***fascipennis*** (Ruthe, 1856, *Microctonus*) E

*fasciipennis* misspelling

*aciculata* Belokobylskij, 1993

***fulvipes*** Curtis, 1833 E

***heterocordyli*** Richards, 1967 E

***Peristenus*** Förster, 1863

***accinctus*** (Haliday, 1835, *Leiophron*)

*laeviventris* (Ruthe, 1856, *Microctonus*)

*intermedius* (Ruthe, 1856, *Microctonus*)

***antennalis*** (Hincks, 1943, *Leiophron*) preocc.^^[[159]](#footnote-160)^^ I

added by van Achterberg (1997)

*picipes* (Haliday, 1835, *Leiophron*) preocc.

***facialis*** (Thomson, 1892, *Euphorus*) E W

*fascialis* misspelling

*microcerus* (Thomson, 1892, *Euphorus*)

***grandiceps*** (Thomson, 1892, *Euphorus*)^^[[160]](#footnote-161)^^ E I

***malatus*** Loan, 1976 S Loan (1976)

***nitidus*** (Curtis, 1833, *Leiophron*) E

***orchesiae*** (Curtis, 1833, *Leiophron*) E

*rufibarbis* (Curtis, 1837, *Leiophron*)

***orthotyli*** (Richards, 1967, *Leiophron*) E

***pallipes*** (Curtis, 1833, *Leiophron*)^^[[161]](#footnote-162)^^ E S I M

*barbiger* (Wesmael, 1835, *Microctonus*)

*pallipes* (Herrich-Schäffer, 1838, *Perilitus*) preocc.

*mellipes* (Cresson, 1872, *Euphorus*)

*punctatus* (Provancher, 1883, *Microctonus*)

*tuberculifer* (Marshall, 1887, *Euphorus*)

*nocturnus* (Viereck, 1905, *Brachistes*)

***picipes*** (Curtis, 1833, *Leiophron*) E I

*coactus* (Marshall, 1887, *Euphorus*)

species excluded from the British and Irish list

[***brevicornis*** (Herrich-Schäffer, 1838, *Perilitus*)^^[[162]](#footnote-163)^^]

[***duplobrevicornis*** (Shenefelt, 1969, *Leiophron*)

*brevicornis* (Ruthe, 1856, *Microctonus*) preocc.^^[[163]](#footnote-164)^^]

[***mitis*** (Haliday, 1833, *Leiophron*)^^[[164]](#footnote-165)^^]

Tribe HELORIMORPHINI Schmiedeknecht, 1907

***CHRYSOPOPHTHORUS*** Goidanich, 1948

***hungaricus*** (Kiss, 1927, *Helorimorpha*) E added by Shaw (1996*a*)

*chrysopimaginis* Goidanich, 1948

*elegans* Tobias, 1961

***Wesmaelia*** Förster, 1863

***petiolata*** (Wollaston, 1858, *Euphorus*) E

*pendula* Förster, 1863

*cremasta* Marshall, 1872

*americana* Myers, 1917

*asiatica* Shestakov, 1932

Tribe MYIOCEPHALINI Chen & van Achterberg, 1997

LOXOCEPHALINI Shaw, 1985 invalid

***Myiocephalus*** Marshall, 1897

*LOXOCEPHALUS* Förster, 1863 preocc.

*SPILOMMA* Morley, 1909

***boops*** (Wesmael, 1835, *Microctonus*) E I

*longipes* (Förster, 1863, *Loxocephalus*)

*laticeps* (Provancher, 1886, *Gamosecus*)

*falconivibrans* (Morley, 1909, *Spilomma*)

*hedini* (Fahringer, 1930, *Aphidius*)

Tribe Neoneurini Bengtsson, 1918^^[[165]](#footnote-166)^^

Elasmosomini Viereck, 1918

***ELASMOSOMA*** Ruthe, 1858

*PARAMIRAX* Ashmead, 1895 unavailable

***berolinense*** Ruthe, 1858 E added by Shaw (2009)

***Neoneurus*** Haliday, 1838

*ECCLITES* Förster, 1863

*SIXIA* Vollenhoven, 1867 van Achterberg (1997)

***auctus*** (Thomson, 1895, *Elasmosoma*) E S

*halidaii* Marshall, 1897

*bistigmaticus* (Morley, 1909, *Euphorus*)

Tribe Perilitini Förster, 1863

Microctonini Shaw, 1985

***Microctonus*** Wesmael, 1835^^[[166]](#footnote-167)^^

*GAMOSECUS* Provancher, 1880

***aciculatus*** (Haeselbarth, 2008, *Perilitus*) I added by Haeselbarth (2008)

***aethiops*** Nees, 1834 E I

*spurius* Ruthe, 1856 Haeselbarth (2008)

?*brevispina* (Thomson, 1892, *Euphorus*) Haeselbarth (2008)

*aethiopoides* Loan, 1975 Haeselbarth (2008)

***alticae*** (Haeselbarth, 2008, *Perilitus*) E added by Haeselbarth (2008)

***aphthonae*** (Haeselbarth, 2008, *Perilitus*) E I added by Haeselbarth (2008)

***apiophaga*** Loan, 1974

***areolatus*** (Thomson, 1892, *Perilitus*) E

***belokobylskiji*** (Haeselbarth, 2008, *Perilitus*) I added by Haeselbarth (2008)

***brassicae*** (Haeselbarth, 2008, *Perilitus*) E added by Haeselbarth (2008)

***brevicollis*** (Haliday, 1835, *Perilitus*) E I

***cerealium*** (Haliday, 1835, *Perilitus*) E I

*secalis* (Haliday, 1833, *Perilitus*) unavailable

***colesi*** Drea, 1968 S I added by Haeselbarth (2008)

***consuetor*** (Nees, 1834, *Perilitus*) S added by Haeselbarth (2008)

***debilis*** (Wollaston, 1858, *Perilitus*) I added by Haeselbarth (2008)

?*gracilipes* (Thomson, 1892, *Perilitus*)

***fagi*** (Haeselbarth, 2008, *Perilitus*) E I added by Haeselbarth (2008)^^[[167]](#footnote-168)^^

***fittkaui*** (Haeselbarth, 2008, *Perilitus*) E added by Haeselbarth (2008)

***flaviventris*** (Thomson, 1892, *Perilitus*) I added by Haeselbarth (2008)

*areolatus* (Thomson, 1892, *Perilitus*)

***haszprunari*** (Haeselbarth, 2008, *Perilitus*) I added by Haeselbarth (2008)

***lipari*** Čapek & Starý, 1995 E added by Haeselbarth (2008)

***melanopus*** Ruthe, 1856 E S I added by Haeselbarth (2008)

***parcicornis*** Ruthe, 1856 E

***perforatus*** (Haeselbarth, 2008, *Perilitus*) E added by Haeselbarth (2008)

***podargae*** (Haeselbarth, 2008, *Perilitus*) E added by Haeselbarth (2008)

***retusus*** Ruthe, 1856 E I added by Luff (1976*b*)^^[[168]](#footnote-169)^^

*lancearius* Ruthe, 1856 Haeselbarth (2008)

*caudatus* (Thomson, 1892, *Perilitus*) Haeselbarth (2008)

***silvularis*** (Haeselbarth, 2008, *Perilitus*) S I added by Haeselbarth (2008)

***stenocari*** (Haeselbarth, 2008, *Perilitus*) S added by Haeselbarth (2008)

***strophosomi*** (Haeselbarth, 2008, *Perilitus*) S I

added by Haeselbarth (2008)

***thyellae*** (Haeselbarth, 2008, *Perilitus*) E S added by Haeselbarth (2008)

***Perilitus*** Nees, 1818

***areolaris*** Gerdin & Hedqvist, 1985 E added by Haeselbarth (1999)

***dubius*** (Wesmael, 1838, *Microctonus*) E

*rutilus* Herrich-Schäffer, 1838 preocc.

***foveolatus*** Reinhard, 1862 E W

*sicheli* Giard, 1895 Haeselbarth (1999)

***marci*** Haeselbarth, 1999 E added by Haeselbarth (1999)

***rutilus*** (Nees, 1811, *Bracon*) E I

*luteus* Herrich-Schäffer, 1838

*ruralis* Herrich-Schäffer, 1838

*strenuus* Marshall, 1887 Haeselbarth (1999)

*pyri* (Viereck, 1917, *Dinocampus*)

*tuberculus* Zaykov, 1981 Haeselbarth (1999)

species excluded from the British and Irish list

[***falciger*** (Ruthe, 1856, *Microctonus*)^^[[169]](#footnote-170)^^]

***Rilipertus*** Haeselbarth, 1996

***intricatus*** (Ruthe, 1859, *Microctonus*) S I

*borealis* (Thomson, 1892, *Perilitus*) Haeselbarth (1996)

***Spathicopis*** van Achterberg, 1977

***flavocephala*** van Achterberg, 1977 E NMS, det. van Achterberg, added here

Tribe PYGOSTOLINI Belokobylskij, 2000

***Pygostolus*** Haliday, 1833^^[[170]](#footnote-171)^^

***falcatus*** (Nees, 1834, *Leiophron*) E I

*testaceus* misident.

***multiarticulatus*** (Ratzeburg, 1852, *Blacus*) E

*falcatus* (Wesmael, 1838, *Blacus*) preocc.

***otiorhynchi*** (Boudier, 1834, *Bracon*)^^[[171]](#footnote-172)^^ E S

***sticticus*** (Fabricius, 1798, *Ichneumon*) E S I M

*testaceus* (Fallén, 1813, *Bassus*) preocc.

*sticticator* (Thunberg, 1824, *Ichneumon*)

*barynoti* (Boudier, 1834, *Bracon*)

*gigas* (Wesmael, 1835, *Blacus*)

Tribe syntretini Shaw, 1985

***Syntretus*** Förster, 1863^^[[172]](#footnote-173)^^

*Falcosyntretus* Tobias, 1965

*Parasyntretus* Belokobylskij, 1993

***breviradialis*** van Achterberg & Haeselbarth, 2003 E

added by Broad (2009)

***conterminus*** (Nees, 1834, *Perilitus*) E S I

***elegans*** (Ruthe, 1856, *Microctonus*) I added by van Achterberg & Haeselbarth (2003)

*transsylvanicus* (Kiss, 1927, *Perilitus*)

***flevo*** van Achterberg & Haeselbarth, 2003 E I added by van Achterberg & Haeselbarth (2003)

***fuscicoxis*** van Achterberg & Haeselbarth, 2003 E I

added by van Achterberg & Haeselbarth (2003)

***fuscivalvis*** van Achterberg & Haeselbarth, 2003 E S I

added by van Achterberg & Haeselbarth (2003)

***idalius*** (Haliday, 1833, *Perilitus*) E S I

*vernalis* (Wesmael, 1835, *Microctonus*)

*cultus* (Marshall, 1887, *Microctonus*)

***ocularis*** van Achterberg & Haeselbarth, 2003 E W S

added by van Achterberg & Haeselbarth (2003)

***politus*** (Ruthe, 1856, *Microctonus*) E I

*cynthius* (Curtis, 1837, *Microctonus*) nom. nud.

*cynthius* Lyle, 1927 van Achterberg & Haeselbarth (2003)

***pusio*** (Marshall, 1898, *Microctonus*) E I

***splendidus*** (Marshall, 1887, *Microctonus*) E S I

*testaceus* (Capron, 1887, *Microctonus*)

*suffolciensis* (Morley, 1933, *Dyscritus*) **new synonymy^^[[173]](#footnote-174)^^**

*niger* Tobias, 1976

***taegeri*** van Achterberg & Haeselbarth, 2003 E

added by van Achterberg & Haeselbarth (2003)

***xanthocephalus*** (Marshall, 1887, *Microctonus*) E S I

*tempestivus* (Curtis, 1837, *Microctonus*) nom. nud.

*lyctaea* Cole, 1959 van Achterberg & Haeselbarth (2003)

*lyctae* misspelling

***zuijleni*** van Achterberg & Haeselbarth, 2003 E S I

added by van Achterberg & Haeselbarth (2003)

species excluded from the British and Irish list

[***parvicornis*** (Ruthe, 1862, *Microctonus*)^^[[174]](#footnote-175)^^]

Tribe TOWNESILITINI Shaw, 1985

***Streblocera*** Westwood, 1833^^[[175]](#footnote-176)^^

Subgenus ***Streblocera*** Westwood, 1833

*LECYTHODELLA* Enderlein, 1912

***fulviceps*** Westwood, 1833 E

***longiscapha*** Westwood, 1882 E

Subgenus ***EUTANYCERUS*** Förster, 1863

*Villocera* Chen & van Achterberg, 1997

Belokobylskij (2000)

***macroscapa*** (Ruthe, 1856, *Microctonus*) E

*halidayana* (Förster, 1863, *Eutanycerus*)

***TOWNESILITUS*** Haeselbarth & Loan, 1983

***aemulus*** (Ruthe, 1856, *Microctonus*) E W I added by Haeselbarth (1988)

*punctifrontis* (Watanabe, 1955, *Microctonus*)

***bicolor*** (Wesmael, 1835, *Microctonus*) E W I

*breviradialis* (Tobias, 1976, *Microctonus*)

***deceptor*** (Wesmael, 1835, *Microctonus*) E S I

added by Haeselbarth (1988)

***fulviceps*** (Ruthe, 1856, *Microctonus*) E added by Haeselbarth (1988)

Subfamily EXOTHECINAE Förster, 1863

PHANOMERINAE Fahringer, 1928

***Colastes*** Haliday, 1833^^[[176]](#footnote-177)^^

Subgenus ***Colastes*** Haliday, 1833

*EXOTHECUS* Wesmael, 1838

*Phanomeris* Förster, 1863

*Phaenomeris* Dalla Torre, 1898

***affinis*** (Wesmael, 1838, *Exothecus*)^^[[177]](#footnote-178)^^ E S W

NMS, det. Shaw & van Achterberg, added here

***braconius*** Haliday, 1833 E S W I M Shaw & Askew (1976)

*debilis* (Wesmael, 1838, *Exothecus*)

*gracilis* Papp, 1975

***fragilis*** (Haliday, 1836, *Rogas*) E S

*semeyticus* Jakimavičius, 1969

***incertus*** (Wesmael, 1838, *Exothecus*) E S

***magdalenae*** Sterzynski, 1983 E NMS, det. Shaw & van Achterberg, added on Fauna Europaea

***pubicornis*** (Thomson, 1892, *Exothecus*) E S added by Godfray & McGavin (1985)

***vividus*** Papp, 1975 E NMS, det. Shaw, added here

Subgenus ***FUNGIVENATOR*** van Achterberg & Shaw, 2008

***sandei*** van Achterberg & Shaw, 2008 E added by van Achterberg & Shaw (2008)

***Shawiana*** van Achterberg, 1983

*Phanomeris* misident.

***catenator*** (Haliday, 1836, *Rogas*) E S W Shaw & Askew (1976)

***laevis*** (Thomson, 1892, *Exothecus*) E NMS, det. Shaw, added on Fauna Europaea

*rugulosus* (Hellén, 1959, *Colastes*)

***Xenarcha*** Förster, 1863

*ZAMEGASPILUS* Ashmead, 1900

***abnormis*** (Wesmael, 1838, *Exothecus*) E S W

NMS, det. Shaw , added on Fauna Europaea

*glabricollis* (Thomson, 1892, *Exothecus*)

***lustrator*** (Haliday, 1836, *Rogas*) E W I

*dimidiatus* (Nees, 1834, *Bracon*) preocc.

*lustratrix* Schulz, 1906

*thomsoni* (Szépligeti, 1906, *Phanomeris*)

Subfamily GNAMPTODONTINAE Fischer, 1970

GNAPTODONTINAE misspelling

GNAPTOGASTRINAE Tobias, 1976

***Gnamptodon*** Haliday, 1836

*Gnaptodon* Haliday, 1837 suppressed

*DIRAPHUS* Wesmael, 1838

*MESOTAGES* Förster, 1863

***decoris*** (Förster, 1863, *Mesotages*) E W NMS, det. Shaw, added on Fauna Europaea

*klemensiewiczii* Niezabitowski, 1910

*bachmaieri* Fischer, 1957

***pumilio*** (Nees, 1834, *Bracon*) E S I Shaw & Askew (1976)

*pygmaeus* (Wesmael, 1838, *Diraphus*)

Subfamily HELCONINAE Förster, 1863^^[[178]](#footnote-179)^^

Tribe HELCONINI Förster, 1863^^[[179]](#footnote-180)^^

***Helcon*** Nees, 1814

*GYMNOSCELUS* Förster, 1863

*EDYIA* Cameron, 1905

*COELOSTEPHANUS* Kieffer, 1911

***claviventris*** Wesmael, 1835 E added by van Achterberg (1987)

***tardator*** Nees, 1812 E

?*adulterator* (Villers, 1789, *Ichneumon*)

***Helconidea*** Viereck, 1914

***dentator*** (Fabricius, 1804, *Pimpla*)^^[[180]](#footnote-181)^^ E

*aequator* (Nees, 1812, *Helcon*)

*tentator* (Thunberg, 1824, *Ichneumon*) preocc.

*rugator* (Ratzeburg, 1848, *Helcon*)

*dentatrix* (Schulz, 1906, *Pimpla*)

***ruspator*** (Linnaeus, 1758, *Ichneumon*) E

*dentator* (Nees, 1812, *Helcon*) preocc.

***Wroughtonia*** Cameron, 1899

*Duportia* Kieffer, 1921

***spinator*** (Lepeletier, 1825, *Helcon*) E

*annulicornis* (Nees, 1834, *Helcon*)

Subfamily HOMOLOBINAE van Achterberg, 1979

***Homolobus*** Förster, 1863^^[[181]](#footnote-182)^^

*Zele* misident.

Subgenus ***APATIA*** Enderlein, 1920

***truncator*** (Say, 1828, *Bracon*) E

*calcarator* (Wesmael, 1835, *Phylax*)

*melleus* (Cresson, 1872, *Phylax*)

*crassicalcaratus* (Viereck, 1905, *Zele*)

*calcaratrix* (Schulz, 1906, *Zele*)

*fuscitarsis* (Bengtsson, 1918, *Phylacter*)

*simillimus* (Enderlein, 1920, *Apatia*)

*unicolor* (Enderlein, 1920 *Zele*)

*chlorophthalmus* (Nixon, 1938, *Zele*)^^[[182]](#footnote-183)^^

Subgenus ***CHARTOLOBUS*** van Achterberg, 1979

***infumator*** (Lyle, 1914, *Zele*) E S W I

*wesmaeli* (Bengtsson, 1918, *Phylacter*)

*japonicus* (Watanabe, 1932, *Zele*)

Subgenus ***Homolobus*** Förster, 1863

***discolor*** (Wesmael, 1835, *Phylax*) E W I

*pectoralis* (Herrich-Schäffer, 1838, *Rogas*)

van Achterberg (1992*b*)

Subgenus ***OULOPHUS*** van Achterberg, 1979

***flagitator*** (Curtis, 1837, *Zele*) E S W I

*geminator* (Lyle, 1914, *Zele*)

Subgenus ***PHYLACTER*** Reinhard, 1863

***annulicornis*** (Nees, 1834, *Rogas*) E S I

*testaceator* misident.

*simplex* (Herrich-Schäffer, 1838, *Rogas*) van Achterberg (1992*b*)

Subfamily Hormiinae Förster, 1863

***Hormius*** Nees, 1818

*CHLIDONIA* Herrich-Schäffer, 1838

*Hormiellus* Enderlein, 1912

*Mediella* Hedqvist, 1963

*Anhormius* Belokobylskij, 1989

***maderae*** Graham, 1986 E NMS, det. Shaw & van Achterberg, added on Fauna Europaea

***moniliatus*** (Nees, 1811, *Bracon*) E I M

*brevipennis* Hellén, 1957

*dusmeti* (Docavo Alberti, 1960, *Hormiopterus*)

*insularis* Hedqvist, 1965

*coniceps* Hellén, 1957

***piciventris*** Wesmael, 1838^^[[183]](#footnote-184)^^ E S W I M NMS, det. Shaw & van Achterberg, added on Fauna Europaea

Subfamily ICHNEUTINAE Förster, 1863

Tribe ICHNEUTINI Förster, 1863

***Ichneutes*** Nees, 1814

***brevis*** Wesmael, 1835 E I

***reunitor*** Nees, 1816 E I

*costatus* (Zetterstedt, 1838, *Microgaster*)

*laeviventris* Hellén, 1958

*leptostigma* Hellén, 1958

Tribe PROTEROPINI van Achterberg, 1976

***Proterops*** Wesmael, 1835

*Ichneutidea* Ashmead, 1900

*Proteropoides* Viereck, 1909

***nigripennis*** Wesmael, 1835 E S I

Subfamily MACROCENTRINAE Förster, 1863

***Austrozele*** Roman, 1910

*Paniscozele* Enderlein, 1920

*Palinzele* Brues, 1922

*Laevis* Sharma, 1982

***longipalpis*** van Achterberg, 1993 E added by van Achterberg (1993*b*)

***Macrocentrus*** Curtis, 1833^^[[184]](#footnote-185)^^

*Amicroplus* Förster, 1863

*Amicroplites* Dalla Torre, 1898

*Fhogra* Cameron, 1901

*Leptozele* Cameron, 1910

*Metapleurodon* Enderlein, 1920

*Pseudophylacter* Fahringer, 1929

***bicolor*** Curtis, 1833 E S W I M

*limbator* (Ratzeburg, 1848, *Rogas*)

*gracilipes* Telenga, 1935^^[[185]](#footnote-186)^^

***blandus*** Eady and Clark, 1964 E S I

***cingulum*** Brischke, 1882 E

*grandii* Goidanich, 1937

*gifuensis* misident.

***collaris*** (Spinola, 1808, *Bracon*) E I

*ebeninus* (Nees, 1834, *Bracon*)

*dubius* (Wesmael, 1835, *Eubadizon*)

*picipes* (Haliday, 1835, *Helcon*)

*dispar* (Kollar, 1852, *Bracon*) preocc.

*kollari* (Rondani, 1877, *Bracon*) Papp (1996*a*)

*affinis* Hedwig, 1961

*affiniqades* Shenefelt, 1969

***equalis*** Lyle, 1914 E

***infirmus*** (Nees, 1834, *Rogas*) E S I M

***linearis*** (Nees, 1812, *Bracon*) E S I M

*abdominalis* (Fabricius, 1793, *Ichneumon*) preocc.

*abdominator* (Thunberg, 1824, *Ichneumon*)

*fissura* (Thunberg, 1824, *Ichneumon*)

*pallidator* (Zetterstedt, 1838, *Bracon*) preocc.

*tenuis* (Ratzeburg, 1848, *Rogas*)

*iridescens* French, 1880

*gifuensis* Ashmead, 1906

*amicroploides* Viereck, 1912

*pallidatorius* (Fahringer, 1928, *Bracon*)

***marginator*** (Nees, 1811, *Bracon*)^^[[186]](#footnote-187)^^ E S I

*rugator* (Ratzeburg, 1848, *Rogas*)

***nidulator*** (Nees, 1834, *Rogas*) E S W I

*longicaudis* (Herrich-Schäffer, 1838, *Rogas*)

*procerus* Costa, 1884

*curticaudis* Telenga, 1950

***nitidus*** (Wesmael, 1835, *Rogas*) E S

***pallipes*** (Nees, 1811, *Bracon*) E

*pallidipes* Dalla Torre, 1898

***resinellae*** (Linnaeus, 1758, *Ichneumon*) E S

*resinator* (Thunberg, 1824, *Ichneumon*) unavailable

*flavipes* (Ratzeburg, 1844, *Rogas*)

*interstitialis* (Ratzeburg, 1844, *Rogas*)

*obscurator* (Ratzeburg, 1848, *Rogas*)

*intricator* (Ratzeburg, 1852, *Helcon*)

*punctifrons* Thomson, 1895

*sublaevis* Thomson, 1895

***sylvestrellae*** van Achterberg, 2001 E NMS, det. Shaw, added here

***thoracicus*** (Nees, 1811, *Bracon*) E W

*longicornis* (Wesmael, 1835, *Rogas*)

***townesi*** van Achterberg & Haeselbarth, 1983 E S I

added by van Achterberg & Haeselbarth (1983)

Subfamily Meteorinae Cresson, 1887^^[[187]](#footnote-188)^^

ZelinAE Ashmead, 1900

ZemiotinAE van Achterberg, 1976

***Meteorus*** Haliday, 1835^^[[188]](#footnote-189)^^

*Saprotichus* Holmgren, 1868

*PACHYTHECUS* Cameron, 1912 preocc.

***abdominator*** (Nees, 1811, *Perilitus*) E S W I

*brunnipes* (Ruthe, 1862, *Perilitus*)

*bruneipes* Dalla Torre, 1898

*brevipesalis* Shenefelt, 1969

?*delator* (Haliday, 1835, *Perilitus*) van Achterberg (1997)

***abscissus*** Thomson, 1895 E S I

***affinis*** (Wesmael, 1835, *Perilitus*) E S W I

*gracilis* Ruthe, 1862 preocc.

*punctiventris* Ruthe, 1862

*ruthei* Schmiedeknecht, 1897

*voloscensis* Fischer, 1959

***alborossicus*** Lobodenko, 2000 E added by Stigenberg & Ronquist (2011)

***brevicauda*** Thomson, 1895^^[[189]](#footnote-190)^^ E

*thuringiacus* Schmiedeknecht, 1897

*mongolicus* Fahringer, 1935

***cespitator*** (Thunberg, 1824, *Ichneumon*) E S W I

*atrator* (Curtis, 1832, *Zele*)

*similator* (Nees, 1834, *Perilitus*)

*microcerus* (Wesmael, 1835, *Perilitus*)

*humeralis* (Zetterstedt, 1838, *Bracon*)

*rufipes* (Zetterstedt, 1838, *Bracon*)

*ambiguus* Ruthe, 1862

***cinctellus*** (Spinola, 1808, *Bracon*)^^[[190]](#footnote-191)^^ E S W

*fuscipes* (Wesmael, 1835, *Perilitus*)

***cis*** (Bouché, 1834, *Bracon*) E S W I

*profligator* (Haliday, 1835, *Perilitus*) van Achterberg in Belokobylskij *et al.* (2003)

***colon*** (Haliday, 1835, *Perilitus*) E S W I

*fragilis* (Wesmael, 1835, *Perilitus*)

*fasciatus* (Ratzeburg, 1844, *Perilitus*)

*alternatus* Ruthe, 1862

*continuus* Ruthe, 1862

*luridus* Ruthe, 1862

*pallidus* Ruthe, 1862

*trivittatus* Ruthe, 1862

***consimilis*** (Nees, 1834, *Perilitus*) E I

*brevipes* (Wesmael, 1835, *Perilitus*)

*albicornis* Ruthe, 1862

*flagellatus* Alexeev, 1971

***eadyi*** Huddleston, 1980 E W added by Huddleston (1980)

***eklundi*** Stigenberg, 2011 E added by Stigenberg & Ronquist (2011)

***filator*** (Haliday, 1835, *Perilitus*) E S I

*laticeps* (Wesmael, 1835, *Perilitus*)

*hodisensis* Fischer, 1970

***heliophilus*** Fischer, 1970 E added by Huddleston (1980)

***hirsutipes*** Huddleston, 1980 E I added by Huddleston (1980)

***ictericus*** (Nees, 1811, *Bracon*) E S W I M

*minutor* (Thunberg, 1824, *Ichneumon*)

*lucidator* (Trentepohl, 1829, *Bracon*)

*ephippium* (Curtis, 1832, *Zele*)

*xanthomelas* (Wesmael, 1835, *Perilitus*)

*rubriceps* (Ratzeburg, 1844, *Perilitus*)

*confinis* Ruthe, 1862

*consors* Ruthe, 1862

*fallax* Ruthe, 1862

*liquis* Ruthe, 1862

*pleuralis* Ruthe, 1862

*crassicrus* Thomson, 1895

*lophyriphagus* Fahringer, 1934

*dumbletoni* Muesebeck, 1939

*adoxophyesi* Minamikawa, 1954

*makinoharanus* Minamikawa, 1954

***jaculator*** (Haliday, 1835, *Perilitus*) E S W I

*obscurellus* Ruthe, 1862

*tenuicornis* Thomson, 1895

*turcicus* Fahringer, 1944

***limbatus*** Maeto, 1989 E S added by Stigenberg & Ronquist (2011)

***lionotus*** Thomson, 1895 E S added by Huddleston (1980)

*ruficoloratus* Fischer, 1957

***longipilosus*** Stigenberg, 2011 E W added by Stigenberg & Shaw (2013)

***melanostictus*** Capron, 1887 E

*niger* Lyle, 1913

*monachae* Tobias, 1986 Belokobylskij (2000*a*)

***micropterus*** (Haliday, 1835, *Perilitus*) E S W I

***obfuscatus*** (Nees, 1811, *Bracon*) E

*thoracicus* (Curtis, 1832, *Zele*)

*formosus* (Wesmael, 1835, *Perilitus*)

*orchesiae* (Boie, 1841, *Alysia*)

*fodori* Papp, 1973

***obsoletus*** (Wesmael, 1835, *Perilitus*) E S I added by Huddleston (1980)

*viridanae* Johansson, 1964

***oculatus*** Ruthe, 1862 S added by Stigenberg & Shaw (2013)

***pendulus*** (Müller, 1776, *Ichneumon*) E S W I

*pendulator* (Latreille, 1799, *Ichneumon*)

*gyrator* (Thunberg, 1824, *Ichneumon*) van Achterberg in Belokobylskij *et al.* (2003)

*ochraceator* (Curtis, 1832, *Zele*) nom. nud.

*scutellator* (Nees, 1834, *Perilitus*)

*petiolator* (Zetterstedt, 1838, *Bracon*)

*parvulus* Thomson, 1895

***pulchricornis*** (Wesmael, 1835, *Perilitus*) E S W I

added by Huddleston (1980)

*striatus* Thomson, 1895

*thomsoni* Marshall, 1899

*japonicus* Ashmead, 1906

*nipponensis* Viereck, 1912

*baicalensis* Telenga, 1950

*graeffei* Fischer, 1957

*macedonicus* Fischer, 1957

*tuberculifer* Fischer, 1957

***rubens*** (Nees, 1811, *Bracon*) E S W I M

*leviventris* (Wesmael, 1835, *Perilitus*)

*islandicus* Ruthe, 1859

*medianus* Ruthe, 1862

*vulgaris* (Cresson, 1872, *Perilitus*)

*dejanus* (Rondani, 1877, *Perilitus*) Papp (1996*a*)

*scutatus* Costa, 1884

*coquilleti* Ashmead, 1889

*heteroneurus* Thomson, 1895

*mellinervis* Viereck, 1903

*mamestrae* Viereck, 1913

*szechuanensis* Fahringer, 1935

*mesopotamicus* Fischer, 1957

***ruficeps*** Nees, 1834 E S I M

*pallipes* (Wesmael, 1835, *Perilitus*)

*nigritarsis* Ruthe, 1862

*pallidipes* Marshall, 1887

***rufus*** (DeGeer, 1773, *Ichneumon*) E W

*rufus* (Retzius, 1783, *Ichneumon*) preocc.

*unicolor* (Wesmael, 1835, *Perilitus*) van Achterberg in Belokobylskij *et al.* (2003)

*chinensis* (Holmgren, 1868, *Saprotichus*)

***sibyllae*** Stigenberg, 2011 E S added by Stigenberg & Shaw (2013)

***sulcatus*** Szépligeti, 1896 E

*insignis* Muesebeck, 1939

*molorchi* Fischer, 1966

***tabidus*** (Wesmael, 1835, *Perilitus*) E S I

*dubius* Ruthe, 1862

*facialis* Ruthe, 1862

*pentheri* Fischer, 1970

***tenellus*** Marshall, 1887^^[[191]](#footnote-192)^^ S added by Stigenberg & Shaw (2013)

*boreus* Tobias, 1986 Stigenberg & Ronquist (2011)

***versicolor*** (Wesmael, 1835, *Perilitus*) E S W I

*bimaculatus* (Wesmael, 1835, *Perilitus*)

*unicolor* (Hartig, 1838, *Perilitus*) preocc.

*brevicornis* (Ratzeburg, 1844, *Perilitus*) preocc.

*rugator* (Ratzeburg, 1852, *Perilitus*)

*decoloratus* Ruthe, 1862

*ikonomovi* Fischer, 1959

*hartigi* Shenefelt, 1969

***vexator*** (Haliday, 1835, *Perilitus*) E S I

species excluded from the British and Irish list

[***flaviceps*** (Ratzeburg, 1844, *Perilitus*) nom. dub.]

***Zele*** Curtis, 1832^^[[192]](#footnote-193)^^

*Protelus* Förster, 1863

*Zemiotes* Förster, 1863

***albiditarsus*** Curtis, 1832 E S W I

*testaceator* Curtis, 1832

*albitarsis* (Nees, 1834, *Perilitus*)

*dispar* (Wesmael, 1835, *Perilitus*)

*calcitrator* (Curtis, 1837, *Meteorus*)

*wesmaeli* (Boie, 1850, *Perilitus*)

*testaceatrix* Schulz, 1906

***caligatus*** (Haliday, 1835, *Meteorus*) E S W I

*neesii* (Ruthe, 1862, *Meteorus*)

*alaskensis* (Ashmead, 1902, *Dyscoletes*)

*sibiricus* (Fahringer, 1930, *Meteorus*)

***chlorophthalmus*** (Spinola, 1808, *Bracon*) E S I

*chrysophthalmus* (Nees, 1811, *Bracon*)

*pallidus* (Nees, 1811, *Bracon*) Stigenberg & Ronquist (2011)

*nudator* (Thunberg, 1824, *Ichneumon*)

*splendens* (Costa, 1884, *Meteorus*)

*nigricollis* (Thomson, 1895, *Meteorus*)

***deceptor*** (Wesmael, 1835, *Perilitus*) E S W I

*pallitarsis* (Cresson, 1872, *Perilitus*)

*rufulus* (Thomson, 1895, *Meteorus*)

*palliditarsis* (Dalla Torre, 1898, *Meteorus*)

*maximus* (Muesebeck, 1923, *Meteorus*)

*reticulatus* (Muesebeck, 1923, *Meteorus*)

*romani* (Fahringer, 1930, *Meteorus*)

*separandus* (Fischer, 1957, *Meteorus*)

species excluded from the British and Irish list

[***annulicrus*** (Thomson, 1895, *Meteorus*)^^[[193]](#footnote-194)^^]

Subfamily MICROGASTRINAE Förster, 1863^^[[194]](#footnote-195)^^

Tribe Apantelini Viereck, 1918

***Apanteles*** Förster, 1863^^[[195]](#footnote-196)^^

*UROGASTER* Ashmead, 1898

*XESTAPANTELES* Cameron, 1910

*ALLAPANTELES* Brèthes, 1915

*Areolatus* Rao & Chalikwar, 1976 unavailable

***atreus*** Nixon, 1973 E

***brunnistigma*** Abdinbekova, 1969 E S W M

*sotades* Nixon, 1976

***carpatus*** (Say, 1836, *Microgaster*) E

*solitarius* (Ashmead, 1900, *Urogaster*)

*hawaiiensis* (Ashmead, 1901, *Protapanteles*)

*fuscicornis* (Cameron, 1910, *Urogaster*)

*piceoventris* Muesebeck, 1921

*igae* Watanabe, 1932

*sarcitorius* Telenga, 1955

*ultericus* Telenga, 1955

***chrysis*** Nixon, 1973 E

***contaminatus*** (Haliday, 1834, *Microgaster*) S I

***corvinus*** Reinhard, 1880 E S

*lucidus* Szépligeti, 1896

*rasteratus* Fahringer, 1936

*aptus* Papp, 1977

***galleriae*** Wilkinson, 1932 E added by Shaw (2012)

***lacteus*** (Nees, 1834, *Microgaster*)^^[[196]](#footnote-197)^^ E

***lenea*** Nixon, 1976 E S I

***metacarpalis*** (Thomson, 1895, *Microgaster*) E I

***miramis*** Nixon, 1976 E

***obscurus*** (Nees, 1834, *Microgaster*) E I

*arenarius* (Haliday, 1834, *Microgaster*)

***sodalis*** (Haliday, 1834, *Microgaster*) E

*carbonarius* (Ratzeburg, 1848, *Microgaster*) preocc.

*ater* (Ratzeburg, 1852, *Microgaster*) van Achterberg (1997)

*lugens* (Ratzeburg, 1852, *Microgaster*)

*lindbergi* Hedqvist, 1965

***xanthostigma*** (Haliday, 1834, *Microgaster*) E S W M

*ochrostigma* (Wesmael, 1837, *Microgaster*)

*xanthocarpus* Szépligeti, 1901

species of *Apanteles* excluded from the British and Irish list^^[[197]](#footnote-198)^^

[***anomalon*** (Curtis, 1830, *Microgaster*)^^[[198]](#footnote-199)^^]

[***nigripes*** (Ratzeburg, 1844, *Microgaster*)^^[[199]](#footnote-200)^^]

[***picipes*** (Bouché, 1834, *Microgaster*)^^[[200]](#footnote-201)^^]

***Choeras*** Mason, 1981

***arene*** (Nixon, 1973, *Apanteles*) E S I

***dorsalis*** (Spinola, 1808, *Microgaster*) E W

*cruciatus* (Ratzeburg, 1844, *Microgaster*)

*suffolciensis* (Morley, 1902, *Microgaster*)

***parasitellae*** (Bouché, 1834, *Microgaster*)^^[[201]](#footnote-202)^^ E S

*adjuncta* misident.

*flavilabris* (Ratzeburg, 1844, *Microgaster*)

*rufilabris* (Ratzeburg, 1844, *Microgaster*)

*lictorius* (Reinhard, 1880, *Apanteles*)

*polypori* (Gautier & Bonnamour, 1930, *Apanteles*)

***ruficornis*** (Nees, 1834, *Microgaster*) E

*hedymeles* (Nixon, 1973, *Apanteles*)

***tedellae*** (Nixon, 1961, *Apanteles*) E

*epinotiae* (Fischer, 1962, *Apanteles*)

*epinoticida* (Fischer, 1966, *Apanteles*)

***tiro*** (Reinhard, 1880, *Microgaster*) E

***validus*** (Thomson, 1895, *Apanteles*) E

***DOLICHOGENIDEA*** Viereck, 1911

***agilla*** (Nixon, 1972, *Apanteles*) E added by Shaw (2012)

*piratica* (Papp, 1977, *Apanteles*)

***annularis*** (Haliday, 1834, *Microgaster*) E

***appellator*** (Telenga, 1949, *Apanteles*) E added by Shaw (2012)^^[[202]](#footnote-203)^^

*litae* (Nixon, 1972, *Apanteles*)

***artissima*** (Papp, 1971, *Apanteles*) E S W

*abila* (Nixon, 1972, *Apanteles*)

***ate*** (Nixon, 1973, *Apanteles*) E

***bres*** (Nixon, 1973, *Apanteles*) E

***breviventris*** (Ratzeburg, 1848, *Microgaster*) E S W I

*mesoxantha* (Ruschka, 1917, *Apanteles*)

*nilae* (Telenga, 1961, *Apanteles*)

***britannica*** (Wilkinson, 1941, *Apanteles*) E

***candidata*** (Haliday, 1834, *Microgaster*) E S W M

*longicauda* (Wesmael, 1837, *Microgaster*) van Achterberg (1997)

*terebrator* (Ratzeburg, 1852, *Microgaster*)

***coleophorae*** (Wilkinson, 1938, *Apanteles*) E

***coniferae*** (Haliday, 1834, *Microgaster*)^^[[203]](#footnote-204)^^ E M

***credne*** (Nixon, 1973, *Apanteles*) E

***cytherea*** (Nixon, 1972, *Apanteles*) E

***decora*** (Haliday, 1834, *Microgaster*) I

*lineata* (Reinhard, 1880, *Apanteles*)

*sibirica* Fahringer, 1938

***dilecta*** (Haliday, 1834, *Microgaster*) E M

*femoralis* (Bouché, 1834, *Microgaster*)

***drusilla*** (Nixon, 1972, *Apanteles*) E

***emarginata*** (Nees, 1834, *Microgaster*) E S W

*scapularis* (Bouché,1834, *Microgaster*)

***exilis*** (Haliday, 1834, *Microgaster*)^^[[204]](#footnote-205)^^ E

***faucula*** (Nixon, 1972, *Apanteles*) E

***gagates*** (Nees, 1834, *Microgaster*) E

***glabra*** (Papp, 1978, *Apanteles*) E S added by Shaw (2012)

***gracilariae*** (Wilkinson, 1940, *Apanteles*) E M

***halidayi*** (Marshall, 1872, *Apanteles*) E S M

*albipennis* (Haliday, 1834, *Microgaster*) preocc.

*halidaii* misspelling

***hilaris*** (Haliday, 1834, *Microgaster*) I

***imperator*** (Wilkinson, 1939, *Apanteles*) E S W M

***infima*** (Haliday, 1834, *Microgaster*) E

***lacteicolor*** (Viereck, 1911, *Apanteles*) E

*conspersae* (Fiske, 1911, *Apanteles*)

***lacteipennis*** (Curtis, 1830, *Microgaster*)

*lissonota* (Tobias, 1964, *Apanteles*)

***laevigata*** (Ratzeburg, 1848, *Microgaster*) E S W

*hoplites* (Ratzeburg, 1848, *Microgaster*)

*calcarata* (Ivanov, 1899, *Apanteles*)

***laevigatoides*** (Nixon, 1972, *Apanteles*) E

***laevissima*** (Ratzeburg, 1848, *Microgaster*) E

*tersa* (Papp, 1973, *Apanteles*)

***lemariei*** (Nixon, 1961, *Apanteles*) E

***lineipes*** (Wesmael, 1837, *Microgaster*) E S M

***longicalcar*** (Thomson, 1895, *Apanteles*) E

***longipalpis*** (Reinhard, 1880, *Apanteles*) E S

*tadzhica* (Telenga, 1949, *Apanteles*)^^[[205]](#footnote-206)^^

***marica*** (Nixon, 1972, *Apanteles*) E

***myron*** (Nixon, 1973, *Apanteles*)^^[[206]](#footnote-207)^^ E S

***ononidis*** (Marshall, 1889, *Apanteles*)^^[[207]](#footnote-208)^^ E

***petrovae*** (Walley, 1937, *Apanteles*) E added by Shaw (2012)

*dioryctriae* (Wilkinson, 1938, *Apanteles*)

*magna* (Telenga, 1955, *Apanteles*)

*murinanae* (Čapek & Zwölfer, 1957, *Apanteles*)

***phaloniae*** (Wilkinson, 1940, *Apanteles*) E S W I

***phaola*** (Nixon, 1972, *Apanteles*) E

***praetor*** (Marshall, 1885, *Apanteles*) E

***princeps*** (Wilkinson, 1941, *Apanteles*) E S W

***punctiger*** (Wesmael, 1837, *Microgaster*) E S

*itea* (Nixon, 1972, *Apanteles*)

***sicaria*** (Marshall, 1885, *Apanteles*) E S

*chrysosticta* (Marshall, 1899, *Apanteles*)

*crudelis* (Papp, 1971, *Apanteles*)

***sisenna*** (Nixon, 1972, *Apanteles*) E^^[[208]](#footnote-209)^^

***soikai*** (Nixon, 1972, *Apanteles*) E added by Shaw (2012)

#***trachala*** (Nixon, 1965, *Apanteles*) E S W I^^[[209]](#footnote-210)^^

*sevocata* (Papp, 1975, *Apanteles*)

***ultor*** (Reinhard, 1880, *Apanteles*) E

*lactipennis* (Ratzeburg, 1852, *Microgaster*) preocc.

***victor*** (Wilkinson, 1941, *Apanteles*) E

species excluded from the British and Irish list

[***anarsiae*** (Faure & Alabouvette, 1924, *Apanteles*)^^[[210]](#footnote-211)^^]

[***cerialis*** (Nixon, 1976, *Apanteles*)^^[[211]](#footnote-212)^^

*areolaris* (Balevski & Tobias, 1980, *Apanteles*) preocc.]

[***ensiformis*** (Ratzeburg, 1844, *Microgaster*)^^[[212]](#footnote-213)^^]

[***evonymellae*** (Bouché, 1834, *Microgaster*)^^[[213]](#footnote-214)^^

*iarbas* (Nixon, 1972, *Apanteles*)]

[***impura*** (Nees, 1834, *Microgaster*)^^[[214]](#footnote-215)^^]

***ILLIDOPS*** Mason, 1981

***butalidis*** (Marshall, 1888, *Apanteles*) E S

***naso*** (Marshall, 1885, *Apanteles*) E

*contortus* (Tobias, 1964, *Apanteles*)

*crantor* (Nixon, 1965, *Apanteles*)

*evander* (Nixon, 1965, *Apanteles*)

*coresia* (Nixon, 1973, *Apanteles*)

***suevus*** (Reinhard, 1880, *Apanteles*) E

*minutus* (Szépligeti, 1896, *Apanteles*)

*polonicus* (Fahringer, 1936, *Apanteles*)

*brevisternis* (Tobias, 1964, *Apanteles*)

*suspicax* (Tobias, 1964, *Apanteles*)

*dion* (Nixon, 1965, *Apanteles*)

*sesostris* (Nixon, 1976, *Apanteles*)

species excluded from the British and Irish list

[***Napamus*** Papp, 1993

***vipio*** (Reinhard, 1880, *Apanteles*)^^[[215]](#footnote-216)^^]

***PHOLETESOR*** Mason, 1981

***arisba*** (Nixon, 1973, *Apanteles*) E S

***bicolor*** (Nees, 1834, *Microgaster*)^^[[216]](#footnote-217)^^ E Shaw & Askew (1976)

*ardeaepenellae* (Bouché, 1834, *Microgaster*)

*umbellatarum* (Haliday, 1834, *Microgaster*)

*schillei* (Niezabitowski, 1910, *Apanteles*)

*longicauda* (Fahringer, 1938, *Apanteles*)

*pedias* (Nixon, 1973, *Apanteles*)

***circumscriptus*** (Nees, 1834, *Microgaster*) E S W I

*exiguus* (Haliday, 1834, *Microgaster*) van Achterberg (1997)

*blancardellae* (Bouché, 1834, *Microgaster*)

*lividipes* (Wesmael, 1837, *Microgaster*)

*flavolimbatus* (Ratzeburg, 1848, *Microgaster*)

*lautellus* (Marshall, 1895, *Apanteles*)

***elpis*** (Nixon, 1973, *Apanteles*) E

*girkanus* (Tobias, 1976, *Apanteles*)

***errans*** (Nixon, 1973, *Apanteles*) E

*arenicola* (Papp, 1973, *Apanteles*)

***laetus*** (Marshall, 1885, *Apanteles*) E S

*exiguus* misident.^^[[217]](#footnote-218)^^

*salalicus* misident.

*metallicus* (Jakimavičius, 1972, *Apanteles*)

***maritimus*** (Wilkinson, 1941, *Apanteles*) E S W

***moldavicus*** (Tobias, 1975, *Apanteles*)^^[[218]](#footnote-219)^^ E added by Shaw (2012)

***nanus*** (Reinhard, 1880, *Apanteles*) E S W M

*szoecsi* (Papp, 1973, *Apanteles*)

***phaetusa*** (Nixon, 1973, *Apanteles*) E S

***viminetorum*** (Wesmael, 1837, *Microgaster*) E S W I

*fuliginosus* (Wesmael, 1837, *Microgaster*)

Tribe COTESIINI Mason, 1981

***COTESIA*** Cameron, 1891^^[[219]](#footnote-220)^^

*CRYPTAPANTELES* Viereck, 1910

*STENOPLEURA* Viereck, 1911

***abjecta*** (Marshall, 1885, *Apanteles*) E S

*complanata* (Lyle, 1916, *Apanteles*)

***affinis*** (Nees, 1834, *Microgaster*) E

*euphorbiae* (Bouché, 1834, *Microgaster*)

*vinulae* (Bouché, 1834, *Microgaster*)

*harpyiae* (Niezabitowski, 1910, *Apanteles*)

*okamotoi* (Watanabe, 1932, *Apanteles*)

*planus* (Watanabe, 1932, *Apanteles*)

***analis*** (Nees, 1834, *Microgaster*) E

*praetextata* (Haliday, 1834, *Microgaster*)

*mediana* (Ratzeburg, 1852, *Microgaster*)

*leucaniae* (Wikinson, 1937, *Apanteles*)

***astrarches*** (Marshall, 1889, *Apanteles*) E S W

?*arctica* (Thomson, 1895, *Apanteles*)^^[[220]](#footnote-221)^^

*genalis* (Tobias, 1964, *Apanteles*)

***bignellii*** (Marshall, 1885, *Apanteles*) E S W I

***brevicornis*** (Wesmael, 1837, *Microgaster*) E S

*cleoceridis* (Marshall, 1889, *Apanteles*)

***cajae*** (Bouché, 1834, *Microgaster*) E

*difficilis* (Nees, 1834, *Microgaster*)

***callimone*** (Nixon, 1974, *Apanteles*) S W I

*scelerata* (Tobias, 1986, *Apanteles*)

***chares*** (Nixon, 1965, *Apanteles*) E

***cleora*** (Nixon, 1974, *Apanteles*) E

***coryphe*** (Nixon, 1974, *Apanteles*)^^[[221]](#footnote-222)^^ E

***cuprea*** (Lyle, 1925, *Apanteles*) E S

***errator*** (Nixon, 1974, *Apanteles*) E W

***eulipis*** (Nixon, 1974, *Apanteles*) E S

*eulipsis* misspelling

**?*euryale*** (Nixon, 1974, *Apanteles*) E^^[[222]](#footnote-223)^^

***ferruginea*** (Marshall, 1885, *Apanteles*) E

***gades*** (Nixon, 1974, *Apanteles*) E added by Allen (1978)

***gastropachae*** (Bouché, 1834, *Microgaster*) E S M

***geryonis*** (Marshall, 1885, *Apanteles*) E

***glomerata*** (Linnaeus, 1758, *Ichneumon*) E S M

*glomerator* (Thunberg, 1824, *Ichneumon*)

*nigriventris* (Nees, 1834, *Microgaster*)

*recondita* (Nees, 1834, *Microgaster*)

*stellatarum* (Bouché, 1834, *Microgaster*)

*crataegi* (Ratzeburg, 1844, *Microgaster*)

*oleracea* (Taylor, 1860, *Microgaster*)

*pieridis* (Packard, 1881, *Microgaster*) preocc.

*pieridivora* (Riley, 1882, *Microgaster*)

*aporiae* (Ivanov, 1899, *Apanteles*)

*nawaii* (Ashmead, 1906, *Glyptapanteles*)

*aporiae* (Matsumura, 1908, *Apanteles*) preocc.

*heterotergis* (Fahringer, 1936, *Apanteles*)

***gonopterygis*** (Marshall, 1885, *Apanteles*) E

***hyphantriae*** (Riley, 1887, *Apanteles*) E W

***inducta*** (Papp, 1973, *Apanteles*) E I added by Revels (2006); Shaw (2007)

*tenuivalvis* (Tobias, 1986, *Apanteles*)

***isolde*** (Nixon, 1974, *Apanteles*) E S

***jucunda*** (Marshall, 1885, *Apanteles*) E S

*nigrinervis* (Thomson, 1895, *Microgaster*)

***juniperatae*** (Bouché, 1834, *Microgaster*) E S

***kurdjumovi*** (Telenga, 1955, *Apanteles*) E S

*laverna* (Nixon, 1974, *Apanteles*)

***limbata*** (Marshall, 1885, *Apanteles*) E S

*kawadai* (Watanabe, 1934, *Apanteles*)

***lineola*** (Curtis, 1830, *Microgaster*) E

*gabrielis* (Gautier & Riel, 1919, *Apanteles*)

***melanoscela*** (Ratzeburg, 1844, *Microgaster*) E

*solitaria* (Ratzeburg, 1844, *Microgaster*)

*creata* (Balevski, 1980, *Apanteles*)

***melitaearum*** (Wilkinson, 1937, *Apanteles*) E S W

*melittaearum* misspelling

*ukrainica* (Tobias, 1986, *Apanteles*)

***notha*** (Marshall, 1885, *Apanteles*) E S

***numen*** (Nixon, 1974, *Apanteles*) E S

***ofella*** (Nixon, 1974, *Apanteles*) E

?*perspicua* (Nees, 1834, *Microgaster*)^^[[223]](#footnote-224)^^

***onaspis*** (Nixon, 1974, *Apanteles*) E

*avetyanae* (Tobias, 1976, *Apanteles*)

***orestes*** (Nixon, 1974, *Apanteles*) E

***pilicornis*** (Thomson, 1890, *Microgaster*) E S W I^^[[224]](#footnote-225)^^

*piliflagellaris* (Tobias, 1986, *Apanteles*)

***praepotens*** (Haliday, 1834, *Microgaster*)^^[[225]](#footnote-226)^^ E I

*placida* (Haliday, 1834, *Microgaster*) van Achterberg (1997)

*memnon* (Nixon, 1974, *Apanteles*) van Achterberg (1997)

*acutivalvis* (Balevski, 1980, *Apanteles*)

*beshtaui* (Tobias, 1986, *Apanteles*)

***risilis*** (Nixon, 1974, *Apanteles*) E

***rubecula*** (Marshall, 1885, *Apanteles*) E

***rubripes*** (Haliday, 1834, *Microgaster*) E S

***ruficrus*** (Haliday, 1834, *Microgaster*) E W M

*antipoda* (Ashmead, 1900, *Apanteles*)

*manilae* (Ashmead, 1904, *Apanteles*)

*sydneyensis* (Cameron, 1911, *Apanteles*)

*narangae* (Viereck, 1913, *Apanteles*)

*sesamiae* (Risbec, 1956, *Apanteles*) nom. nud.

***salebrosa*** (Marshall, 1885, *Apanteles*) E S

*callunae* Nixon, 1974

***saltatoria*** (Balevski, 1980, *Apanteles*) E S added by Shaw (2007)

***sericea*** (Nees, 1834, *Microgaster*) E S

*praepotens* misident.^^[[226]](#footnote-227)^^

*brachycera* (Thomson, 1895, *Apanteles*) van Achterberg (1997)

***sibyllarum*** (Wilkinson, 1936, *Apanteles*) E

***spuria*** (Wesmael, 1837, *Microgaster*) E S W M

*insidens* (Ratzeburg, 1844, *Microgaster*)

***subordinaria*** (Tobias, 1976, *Apanteles*) E added by Shaw (2012)

***telengai*** (Tobias, 1972, *Apanteles*) E

*amabilis* (Nixon, 1974, *Apanteles*)

***tenebrosa*** (Wesmael, 1837, *Microgaster*) E S added by Shaw (2007)

?*arctica* (Thomson, 1895, *Apanteles*)^^[[227]](#footnote-228)^^

***tetrica*** (Reinhard, 1880, *Apanteles*) E S

*opacula* (Thomson, 1895, *Microgaster*)

***tibialis*** (Curtis, 1830, *Microgaster*) E S W

*atrator* (Curtis, 1830, *Microgaster*)

*gracilis* (Curtis, 1830, *Microgaster*)

*congesta* (Nees, 1834, *Microgaster*)

*intricata* (Haliday, 1834, *Microgaster*)

*gracilipes* (Thomson, 1895, *Microgaster*)

*similis* (Szépligeti, 1901, *Apanteles*)

*atratrix* (Schulz, 1906, *Microgaster*)

*aranearum* (Goureau, 1908, *Apanteles*) nom. nud.

*mamestrae* (Matsumura, 1908, *Apanteles*)

*simulans* (Lyle, 1917, *Apanteles*)

*claustrata* (Gautier & Bonnamour, 1923, *Apanteles*)

***vanessae*** (Reinhard, 1880, *Apanteles*) E

***vestalis*** (Haliday, 1834, *Microgaster*) E W

*plutellae* (Kurdjumov, 1912, *Apanteles*) Shaw (2003*a*)

***villana*** (Reinhard, 1880, *Apanteles*) E

*fasciatae* (Gautier & du Dresnay, 1926 *Apanteles*)

*rubroides* (Papp, 1971, *Apanteles*) Papp (2009*b*)

***zygaenarum*** (Marshall, 1885, *Apanteles*) E S W I M

species excluded from the British and Irish list

[***acuminata*** (Reinhard, 1880, *Apanteles*)

*cultrator* (Marshall, 1885, *Apanteles*)^^[[228]](#footnote-229)^^]

[***ordinaria*** (Ratzeburg, 1844, *Microgaster*^^[[229]](#footnote-230)^^)

*dendrolimi* (Matsumura, 1926, *Apanteles*)

*dendrolimusi* (Matsumura, 1926, *Apanteles*)]

[***saltator*** (Thunberg, 1824, *Ichneumon*) preocc.^^[[230]](#footnote-231)^^]

[***scabricula*** (Reinhard, 1880, *Apanteles*)^^[[231]](#footnote-232)^^

*eguchii* (Watanabe, 1935, *Apanteles*)]

***DEUTERIXYS*** Mason, 1981

***carbonaria*** (Wesmael, 1837, *Microgaster*) E S

*anomala* (Lyle, 1925, *Apanteles*)

***plugarui*** (Tobias, 1975, *Apanteles*) E added by Shaw (2012)

***rimulosa*** (Niezabitowski, 1910, *Apanteles*) E

*comes* (Wilkinson, 1940, *Apanteles*)

***DIOLCOGASTER*** Ashmead, 1901

*PROTOMICROPLITIS* misident.^^[[232]](#footnote-233)^^

*ZADIOLOCOGASTER* Viereck, 1913

***abdominalis*** (Nees, 1834, *Microgaster*) E

***alvearia*** (Fabricius, 1798, *Ichneumon*) E S

*aphidum* (Panzer, 1804, *Ichneumon*)

*alveator* (Thunberg, 1824, *Ichneumon*)

*areolata* (Szépligeti, 1896, *Ichneumon*)

***connexa*** (Nees, 1834, *Microgaster*) E M

*consularis* (Haliday, 1834, *Microgaster*)

*diluta* (Ratzeburg, 1852, *Microgaster*)

***flavipes*** (Haliday, 1834, *Microgaster*)

***hinzi*** (Nixon, 1965, *Protomicroplitis*) E S added by Shaw (2012)

***minuta*** (Reinhard, 1880, *Microgaster*) E S

***scotica*** (Marshall, 1885, *Microgaster*) E S

***spreta*** (Marshall, 1885, *Microgaster*) E S W

***DISTATRIX*** Mason, 1981

***formosa*** (Wesmael, 1837, *Microgaster*) E W M

*marshallii* (Bignell, 1901, *Apanteles*)

***GLYPTAPANTELES*** Ashmead, 1905

***acasta*** (Nixon, 1973, *Apanteles*) E

***aliphera*** (Nixon, 1973, *Apanteles*) E S

*aliphaera* misspelling

*sublateralis* (Tobias, 1976, *Apanteles*)

***callidus*** (Haliday, 1834, *Microgaster*)^^[[233]](#footnote-234)^^ E S W

*urolus* (Papp, 1983, *Apanteles*) van Achterberg (1997)

***compressiventris*** (Muesebeck, 1921, *Apanteles*) S W

*liparidis* misident.^^[[234]](#footnote-235)^^

***fausta*** (Nixon, 1973, *Apanteles*)^^[[235]](#footnote-236)^^ E S

***fraternus*** (Reinhard, 1881, *Apanteles*) E

***fulvipes*** (Haliday, 1834, *Microgaster*) E S W I

***inclusus*** (Ratzeburg, 1844, *Microgaster*) E

*curvulus* (Thomson, 1895, *Microgaster*)

*rectinervis* (Telenga, 1955, *Apanteles*)

***lateralis*** (Haliday, 1834, *Microgaster*)^^[[236]](#footnote-237)^^ E S W

***luciana*** (Nixon, 1973, *Apanteles*) S^^[[237]](#footnote-238)^^

***majalis*** (Wesmael, 1837, *Microgaster*) E S

*callidus* misident.^^[[238]](#footnote-239)^^

***menander*** (Nixon, 1973, *Apanteles*) S

***mygdonia*** (Nixon, 1973, *Apanteles*) E S I

***octonarius*** (Ratzeburg, 1852, *Microgaster*) E

*stauropodis* (Bridgman, 1889, *Apanteles*)

*lucifugus* (Lyle, 1917, *Apanteles*)

***pallipes*** (Reinhard, 1880, *Apanteles*) E S W

*pallidipes* (Marshall, 1885, *Apanteles*)

*longicornis* (Provancher, 1886, *Microgaster*)

*radiatus* (Ashmead, 1898, *Apanteles*)

*reinhardi* (Wilkinson, 1936, *Apanteles*)

***pinicola*** (Lyle, 1917, *Apanteles*) E S

***porthetriae*** (Muesebeck, 1928, *Apanteles*) E added by Shaw & Skelton (2008)

***salepus*** (Papp, 1983, *Apanteles*) E added by Shaw (2012)

***vitripennis*** (Curtis, 1830, *Microgaster*) E S M

*fulcriger* (Wesmael, 1837, *Microgaster*)

*impavidus* (Gautier & Dresnyay, 1927, *Apanteles*)

species excluded from the British and Irish list

[***thompsoni*** (Lyle, 1917, *Apanteles*)^^[[239]](#footnote-240)^^]

***PROTAPANTELES*** Ashmead, 1898

***anchisiades*** (Nixon, 1973, *Apanteles*) E S W M

***endemus*** (Nixon, 1965, *Apanteles*) E S

***enephes*** (Nixon, 1965, *Apanteles*) E

***hirtariae*** (Kotenko & Tobias, 1986, *Apanteles*) S

added by Shaw (2012)

***immunis*** (Haliday, 1834, *Microgaster*) E S W I

***incertus*** (Ruthe, 1859, *Microgaster*) E S M

*caberae* (Marshall, 1885, *Apanteles*)

*jugosus* (Lyle, 1916, *Apanteles*)

*mihalyii* (Papp, 1973, *Apanteles*)

***parallelus*** (Lyle, 1917, *Apanteles*) E

*lylei* (Shenefelt, 1972, *Apanteles*)

***popularis*** (Haliday, 1834, *Microgaster*) E S

***triangulator*** (Wesmael, 1837, *Microgaster*)^^[[240]](#footnote-241)^^ E

***RASIVALVA*** Mason, 1981

***calceata*** (Haliday, 1834, *Microgaster*) E S

*pubescens* (Ratzeburg, 1844, *Microgaster*)

***circumvecta*** (Lyle, 1918, *Diolcogaster*) E S

***marginata*** (Nees, 1834, *Microgaster*) E S

Tribe MICROGASTRINI Förster, 1863

***HYGROPLITIS*** Thomson, 1895

***pseudorussata*** Shaw, 1992 E added by Shaw (1992*a*)

***rugulosa*** (Nees, 1834, *Microgaster*) E W I

*infumata* (Haliday, 1834, *Microgaster*)

*opaca* (Ruthe, 1858, *Microgaster*)

***russata*** (Haliday, 1834, *Microgaster*) E S^^[[241]](#footnote-242)^^ W

*dimidiata* (Wesmael, 1837, *Microgaster*)

*basalis* (Stephens, 1846, *Microgaster*)

*aomoriensis* (Matsumura, 1910, *Microgaster*)

***ICONELLA*** Mason, 1981

***aeola*** (Nixon, 1965, *Apanteles*) E added by Shaw (2012)

species excluded from the British and Irish list^^[[242]](#footnote-243)^^

[***lacteoides*** (Nixon, 1965, *Apanteles*)

*memorabilis* (Alexeev, 1971, *Apanteles*)

***merula*** (Reinhard, 1880, *Apanteles*)

?*etiellae* (Viereck, 1911, *Apanteles*)]

***Microgaster*** Latreille, 1804^^[[243]](#footnote-244)^^

*LIGANIRA* Walker, 1860

*LISSOGASTER* Bengtsson, 1926

***acilia*** Nixon, 1968^^[[244]](#footnote-245)^^ E

***alebion*** Nixon, 1968 E S Shaw (2004)

***areolaris*** Thomson, 1895 E S

***arctostaphylica*** Shaw, 2012 S added by Shaw (2012)

***consors*** Nixon, 1968 E

***crassicornis*** Ruthe, 1860 E

***ductilis*** Nixon, 1968 E added by Shaw (2012)

***fulvicrus*** Thomson, 1895 E S I M

*striatoscutellaris* Kiss, 1927

***globata*** (Linnaeus, 1758, *Ichneumon*)^^[[245]](#footnote-246)^^ E S Papp (1976)

*laeviscuta* Thomson, 1895

*gossypina* (Retzius, 1783, *Ichneumon*)

*globator* (Thunberg, 1824, *Ichneumon*)

*anthomyiarum* Bouché, 1834

*amentorum* Ratzeburg, 1844

*incurvata* Papp, 1976 Papp (2002)

***hospes*** Marshall, 1885 E S W

*comptanae* Viereck, 1911

***luctuosa*** Haliday, 1834 E S W

*curvicrus* Thomson, 1895 van Achterberg (1997)

***meridiana*** Haliday, 1834 E S W I M

*spinolae* Haliday, 1834 preocc. van Achterberg (1997)

*alexis* Haliday, 1834 nom. nud.

*grandis* Thomson, 1895 van Achterberg (1997)

*contubernalis* Marshall, 1898

***messoria*** Haliday, 1834^^[[246]](#footnote-247)^^ E S W I

*tibialis* Nees, 1834 preocc. van Achterberg (1997)

*vulgaris* Ruthe, 1860

*pluto* Morley, 1936

***nigricans*** Nees, 1834^^[[247]](#footnote-248)^^

***nixalebion*** Shaw, 2004 E W added by Shaw (2004)

***novicia*** Marshall, 1885 E S

*swammerdamiae* Muesebeck, 1922

***opheltes*** Nixon, 1968 I

***pantographae*** Muesebeck, 1922 E

***parvistriga*** Thomson, 1895 E S

***polita*** Marshall, 1885 E S I

*carinata* Bengtsson, 1926 preocc.

*bengtssoni* Fahringer, 1937

***procera*** Ruthe, 1860 I

*intermedia* Ivanov, 1899

***raschkiellae*** Shaw, 2012 E S W added by Shaw (2012)

*fischeri* misident.

***stictica*** Ruthe, 1858 E S W M

*confusa* Papp, 1971

***subcompleta*** Nees, 1834 E S W I M

*annulipes* Curtis, 1830

*carinata* Packard, 1881

species excluded from the British and Irish list

[***auriculata*** (Fabricius, 1804, *Ichneumon*)^^[[248]](#footnote-249)^^]

[***deceptor*** Nixon, 1968^^[[249]](#footnote-250)^^]

[***fischeri*** Papp, 1960^^[[250]](#footnote-251)^^]

[***nobilis*** Reinhard, 1880^^[[251]](#footnote-252)^^

*compressifemur* Fahringer, 1937]

[***postica*** Nees, 1834^^[[252]](#footnote-253)^^

*marginella* Wesmael, 1837

?*ruficoxis* Ruthe, 1858]

***PAROPLITIS*** Mason, 1981

***wesmaeli*** (Ruthe, 1860, *Microgaster*) E

*picipes* (Wesmael, 1837, *Microgaster*) preocc.

***SATHON*** Mason, 1981

***falcatus*** (Nees, 1834, *Microgaster*) E S W I

*equestris* (Haliday, 1834, *Microgaster*)

*gladiator* (Szépligeti, 1901, *Apanteles*)

Tribe MICROPLITINI Mason, 1981

***Microplitis*** Förster, 1863^^[[253]](#footnote-254)^^

*DAPSILOTOMA* Cameron, 1906

***aduncus*** (Ruthe, 1860, *Microgaster*) S

*brachycerus* (Thomson, 1895, *Microgaster*)

**?*decens*** Tobias, 1964 S added by Papp (1984)^^[[254]](#footnote-255)^^

***deprimator*** (Fabricius, 1798, *Ichneumon*) E S

*ingratus* (Haliday, 1834, *Microgaster*) van Achterberg (1997)

*sordipes* (Nees, 1834, *Microgaster*)^^[[255]](#footnote-256)^^

*tau* (Ratzeburg, 1852, *Microgaster*)

*deprimatrix* (Schulz, 1906, *Microgaster*)

***flavipalpis*** (Brullé, 1832, *Microgaster*) E

*ruricola* Lyle, 1918

***fordi*** Nixon, 1970^^[[256]](#footnote-257)^^ E S

***fulvicornis*** (Wesmael, 1837, *Microgaster*) E W

*calcarata* misident.

*pallidicornis* Marshall, 1898

***impressus*** (Wesmael, 1837, *Microgaster*) E added by Shaw (2012)

*sispes* Nixon, 1970

***lugubris*** (Ruthe, 1860, *Microgaster*) S

*borealis* Marshall, 1885

*coracinus* (Thomson, 1895, *Microgaster*)

*rutheana* Fahringer, 1937

***malimbus*** (Papp, 1984, *Microgaster*) E added by Shaw (2012)

*trochanterata* misident^^[[257]](#footnote-258)^^

***mandibularis*** Thomson, 1895 E S

***mediator*** (Haliday, 1834, *Microgaster*) E S W I M

*medianus* (Ruthe, 1860, *Microgaster*)

*halidayi* Fahringer, 1937

*pseudomedianus* Fahringer, 1937

***moestus*** (Ratzeburg, 1852, *Microgaster*)

***naenia*** Nixon, 1970 E

***ocellatae*** (Bouché, 1834, *Microgaster*) E

*canaliculatus* (Wesmael, 1837, *Microgaster*)

***scrophulariae*** Szépligeti, 1898 E added by Shaw (2012)

***sofron*** Nixon, 1970 E S I

?*stigmaticus* (Ratzeburg, 1844, *Microgaster*)^^[[258]](#footnote-259)^^

***spectabilis*** (Haliday, 1834, *Microgaster*) E W I

*fossulatus* (Bouché, 1834, *Microgaster*)

?*parvulus* (Ruthe, 1860, *Microgaster*)

*seuratii* Marshall, 1898

*testaceipes* (Cameron, 1906, *Dapsilotoma*)

***spinolae*** (Nees, 1834, *Microgaster*) E

*sapporoensis* Ashmead, 1906

*radiorimatus* Telenga, 1955

?*quadridentatus* (Provancher, 1886, *Microgaster*)

***strenuus*** Reinhard, 1880 E

*gracilis* (Ruthe, 1860, *Microgaster*) preocc.

***tristis*** (Nees, 1834, *Microgaster*) E S W

*dolens* Marshall, 1885

***tuberculatus*** (Bouché, 1834, *Microgaster*) E S I

*fumipennis* (Ratzeburg, 1852, *Microgaster*)

***tuberculifer*** (Wesmael, 1837, *Microgaster*) E S

*calcaratus* (Thomson, 1895, *Microgaster*)

*trochanteratus* (Thomson, 1895, *Microgaster*)

*manevali* Gautier & Bonnamour, 1939

***viduus*** (Ruthe, 1860, *Microgaster*) E

***xanthopus*** (Ruthe, 1860, *Microgaster*) S W I

*tenuipes* (Thomson, 1895, *Microgaster*)

species excluded from the British and Irish list

[***eremitus*** Reinhard, 1880^^[[259]](#footnote-260)^^]

Subfamily MICROTYPINAE Szépligeti, 1908^^[[260]](#footnote-261)^^

***MICROTYPUS*** Ratzeburg, 1848^^[[261]](#footnote-262)^^

*SIMILEARINUS* Glowacki & Karpiński, 1967

***wesmaelii*** Ratzeburg, 1848 E added by Shaw (1992*b*)

*dioryctriae* Rohwer, 1920

Subfamily Miracinae Viereck, 1918

***Mirax*** Haliday, 1833^^[[262]](#footnote-263)^^

*CENTISTIDEA* Rohwer, 1914

***rufilabris*** Haliday, 1833 E S Shaw & Askew (1976); NMS

*spartii* Haliday, 1835

*dryochares* Marshall, 1898

*nanivorae* Fischer, 1957

Subfamily OPIINAE Blanchard, 1845^^[[263]](#footnote-264)^^

***Ademon*** Haliday, 1833

*GIARDINAIA* de Stefani-Perez, 1902

*ANALOSTANIA* Viereck, 1916

***decrescens*** (Nees, 1811, *Bracon*) E S W I

*mutuator* (Nees, 1811, *Bracon*)

***APODESMIA*** Förster, 1863

*ALLOTYPUS* Förster, 1863 Li *et al.* (2013)

*LEMNAPHILOPIUS* Fischer, 1972 Li *et al.* (2013)

*AGNOPIUS* Fischer, 1982 Li *et al.* (2013)

*CRYPTOGNATHOPIUS* Fischer, 1984 Li *et al.* (2013)

***aemula*** (Haliday, 1836, *Opius*) E I

*melba* (Papp, 1978, *Opius*)

***curvata*** (Fischer, 1957, *Opius*) E S added by Godfray (1986)

***irregularis*** (Wesmael, 1835, *Opius*) E I

*bipustulata* (Fischer, 1958, *Opius*)

***ocellata*** (Wesmael, 1835, *Opius*) E S

*areolaris* (Thomson, 1895, *Opius*)

*hungarica* (Szépligeti, 1896, *Opius*)

*bruta* (Papp, 1978, *Opius*)

***posticatae*** (Fischer, 1957, *Opius*) E S

*seebensteinensis* (Fischer, 1959, *Opius*)

*hilaris* (Fischer, 1963, *Opius*)

*hostium* (Fischer, 1964, *Opius*)

***rufipes*** (Wesmael, 1835, *Opius*) E I

*taeniata* Förster, 1863

*taeniata* (Fischer, 1957, *Opius*) preocc.

***saeva*** (Haliday, 1837, *Opius*) E S

***saevula*** (Fischer, 1958, *Opius*) E

***similis*** (Szépligeti, 1898, *Opius*) E S I

*xylostei* (Marshall, 1898, *Opius*)

*similiformis* (Fischer, 1957, *Opius*)

*basirufa* (Fischer, 1958, *Opius*)

*nodata* (Fischer, 1958, *Opius*)

*periclymenii* (Fischer, 1964, *Opius*)

*altimontana* (Fischer, 1969, *Opius*)

*differens* (Fischer, 1958, *Opius*)

*parvipunctum* (Fischer, 1958, *Opius*)

*selkirkensis* (Fischer, 2006, *Opius*) van Achterberg (in prep.)

***ATORMUS*** van Achterberg, 1998

***victus*** (Haliday, 1837, *Opius*)^^[[264]](#footnote-265)^^ E S I

*tarni* (Papp, 1982, *Opius*)

***Bathystomus*** Förster, 1863

*Compressaria* Königsmann, 1959 Wharton (1993)^^[[265]](#footnote-266)^^

***xanthopus*** Förster, 1863 E

*pugnatrix* (Marshall, 1895, *Mesocrina*) van Achterberg (2014)

*compressiventris* (Fischer, 1964, *Opius*) van Achterberg (in prep.)

***BIOPHTHORA*** Förster, 1863^^[[266]](#footnote-267)^^

***bajula*** (Haliday, 1837, *Opius*) E I

*beieri* (Fischer, 1968, *Sternaulopius*) Wharton (2006)

***Biosteres*** Förster, 1863

*Rhabdospilus* Förster, 1863

*Rhinoplus* Förster, 1863

*Stenospilus* Förster, 1863

*Zetetes* Förster, 1863 preocc.

*Opiellus* Ashmead, 1900

*Celiestiella* Cameron, 1903

***analis*** (Wesmael, 1835, *Opius*) E S

*colorativentris* (Fischer, 1957, *Opius*)

***arenarius*** (Stelfox, 1959, *Opius*) I

***bicolor*** Wesmael, 1835 I

*vagator* (Förster, 1863, *Stenospilus*

***carbonarius*** (Nees, 1834, *Bracon*) E S W I

*impressus* (Wesmael, 1835, *Opius*)^^[[267]](#footnote-268)^^

*procerus* (Wesmael, 1835, *Opius*)

*onzi* (Fischer, 1959, *Opius*) van Achterberg (2014)

***haemorrhoeus*** (Haliday, 1837, *Opius*) E I

*castaneiventris* (Thomson, 1895, *Opius*)

*palaearcticus* Szépligeti, 1901

***magnicornis*** (Wesmael, 1835, *Opius*) E I

***micans*** (Stelfox, 1957, *Opius*) S I

*nitidus* (Stelfox, 1949, *Opius*) preocc.

***placidus*** (Haliday, 1837, *Opius*) E I

*melanocerus* (Wesmael, 1838, *Opius*)

*tarsator* (Thomson, 1895, *Opius*)

*indotatus* Viereck,1905

***rusticus*** (Haliday, 1837, *Opius*) E S I

***scabriculus*** (Wesmael, 1835, *Opius*) I

***spinaciae*** (Thomson, 1895, *Opius*) E added by Godfray (1988)

*pegomyiae* (Gahan, 1917, *Opius*)

*hyoscyamiellus* (Viereck, 1925, *Opius*)

***sylvaticus*** (Haliday, 1837, *Opius*) E S I

*clypealis* (Thomson, 1895, *Opius*)

*nitidus* Szépligeti, 1896

***wesmaelii*** (Haliday, 1837, *Opius*) E S

*carbonarius* (Wesmael, 1835, *Opius*) preocc.

*ultor* (Förster, 1863, *Zetetes*)

*ultor* (Fischer, 1957, *Opius*) preocc.

*jonaitisi* (Jakimavičius, 1977, *Opius*)

***BITOMOIDES*** van Achterberg, 2004

***rugosus*** (Wesmael, 1838, *Opius*) E^^[[268]](#footnote-269)^^

*rugiventris* (Thomson, 1895, *Opius*) van Achterberg (2014)

***Chilotrichia*** Förster, 1863

*Trichopius* Thomson, 1895

***blanda*** (Haliday, 1837, *Opius*) E I

***Desmiostoma*** Förster, 1863^^[[269]](#footnote-270)^^

***parvulum*** (Wesmael, 1835, *Opius*) E S

*nudiscutum* (Fischer, 1964, *Opius*)

*ziratus* (Papp, 1982, *Opius*)

*temporale* (Fischer, 1958, *Opius*)

***Diachasma*** Förster, 1863

*Atoreuteus* Förster, 1863

*Lytacra* Förster, 1863

*Alysopius* Tobias, 1976

***caffer*** (Wesmael, 1835, *Opius*)

*stygium* (Förster, 1863, *Lytacra*)

***cephalotes*** (Wesmael, 1835, *Opius*) E

***fulgidum*** (Haliday, 1837, *Opius*) E I

***Eurytenes*** Förster, 1863

Subgenus ***Eurytenes*** Förster, 1863

***abnormis*** (Wesmael, 1835, *Opius*)^^[[270]](#footnote-271)^^ E I

***britannicola*** Fischer, 2006 E added by Fischer (2006)^^[[271]](#footnote-272)^^

Subgenus ***STIGMATOPOEA*** Fischer, 1986^^[[272]](#footnote-273)^^ van Achterberg (2004*a*)

***macrocerus*** (Thomson, 1895, *Opius*) E S I

*hians* (Stelfox, 1949, *Opius*)

Subgenus ***XYNOBIUS*** Förster, 1863^^[[273]](#footnote-274)^^

*ACLISIS* Förster, 1863

*HOLCONOTUS* Förster, 1863

*AULONOTUS* Ashmead, 1900

*ERISTERNAULAX* Viereck, 1914

*XYNOBIOTENES* Fischer, 1998

***aciculatus*** (Thomson, 1895, *Opius*) I

*tenuicornis* (Thomson, 1895, *Opius*) van Achterberg (in prep.)

***aemuloides*** (Fischer, 1958, *Opius*) E

***caelatus*** (Haliday, 1837, *Opius*) E S I

*isomera* (Förster, 1863, *Aclisis*)

*pallipes* Förster, 1863

*pallidipes* Dalla Torre, 1898

***comatus*** (Wesmael, 1835, *Opius*) E I

*sulcifer* (Papp, 1967, *Dapsilarthra*)

***geniculatus*** (Thomson, 1895, *Opius*) E S

*albicoxis* (Marshall, 1898, *Opius*)

***holconotus*** (Fischer, 1958, *Opius*) E

***maculipes*** Wesmael, 1835 E I

*addendus* Fischer, 1959

*turcmenicus* Fischer, 1959

***polyzonius*** (Wesmael, 1835, *Opius*) E S

***silenis*** (Fischer, 1967, *Diachasma*) W^^[[274]](#footnote-275)^^

**sp. A** van Achterberg (in prep.) E reared by Godfray, in NMS

***thomsoni*** (Fischer, 1971, *Opius*) E

*annulicornis* (Thomson, 1895, *Opius*) preocc.

***Neopius*** Gahan, 1917^^[[275]](#footnote-276)^^

***rudis*** (Wesmael, 1835, *Opius*) E I

*carinaticeps* Gahan, 1917

***Opiognathus*** Fischer, 1972^^[[276]](#footnote-277)^^

***pactus*** (Haliday, 1837, *Opius*) E I

***OPIOSTOMUS*** Fischer, 1972^^[[277]](#footnote-278)^^

*SNOFLAKOPIUS* Fischer, 1972 Li *et al.* (2013)

*JUCUNDOPIUS* Fischer, 1984 Li *et al.* (2013)

*OETZALOTENES* Fischer, 1998 Li *et al.* (2013)

*Opiotenes* Fischer, 1998 Li *et al.* (2013)

***aureliae*** (Fischer, 1957, *Opius*) E S added by Godfray & van Achterberg (2015)

***campanariae*** (Fischer, 1959, *Opius*) S added by Godfray & van Achterberg (2015)

***griffithsi*** (Fischer, 1962, *Opius*) E

***leptostigma*** (Wesmael, 1835, *Opius*) E S^^[[278]](#footnote-279)^^

*percontator* (Fischer, 1964, *Opius*)

***Opius*** Wesmael, 1835^^[[279]](#footnote-280)^^

*Cryptonastes* Förster, 1863

*Hypocynodus* Förster, 1863

*Hypolabis* Förster, 1863

*Misophthora* Förster, 1863

*Desmatophorus* Thomson, 1895

*Allophlebus* Fischer,1972

*NOSOPAEOPIUS* Fischer, 1972

*Opiothorax* Fischer, 1972

*Pendopius* Fischer, 1972

*STOMOSEMA* Fischer, 1972

*ODONTOPOEA* Fischer, 1987

***agromyzicola*** Fischer, 1967 E

***ambiguus*** Wesmael, 1835 E S I

*celsus* Haliday, 1837 van Achterberg (2014)

*longipes* Fischer, 1957

*phytomyzae* Fischer, 1957

***brevipalpis*** Thomson, 1895 E

*mutus* Fischer, 1964

*gyoerfii* Fischer, 1958 van Achterberg (2014)

***caudifer*** Fischer, 1958^^[[280]](#footnote-281)^^ E

*longicornis* misident.

***cingulatus*** Wesmael, 1835

*dentifer* Thomson, 1895

*stramineipes* Thomson, 1895

***compar*** Marshall, 1894

*pulchrithorax* Fischer, 1958

***crassipes*** Wesmael, 1835 E

***flammeus*** Fischer, 1959 E

***funebris*** Wesmael, 1835^^[[281]](#footnote-282)^^ E I

*latipes* misident.

***fuscipennis*** Wesmael, 1835^^[[282]](#footnote-283)^^ E

***gracilis*** Fischer, 1957 E

*csikii* Fischer, 1957

*minor* Fischer, 1957

*nigrithorax* Fischer, 1958 preocc.

***instabilis*** Wesmael, 1835

***levis*** Wesmael, 1835 E W I

*apiculator* (Nees, 1834, *Bracon*)

*filicornis* Thomson, 1895^^[[283]](#footnote-284)^^

*varipes* Szépligeti, 1898

***lucidus*** Szépligeti, 1896 E S

***lugens*** Haliday, 1837 E I

*abscissus* Thomson, 1895

*obscurus* Szépligeti, 1901

*adveniens* Fischer, 1960

***nigricoloratus*** Fischer, 1958 S W added by Godfray & van Achterberg (2015)

*dureseaui*Fischer, 1975 van Achterberg (2014)

***ochrogaster*** Wesmael, 1835 E S I

*nigriceps* Szépligeti, 1898

*neopusillus* Fischer, 1957

***orbiculator*** (Nees, 1811, *Bracon*) I

*breviscapus* Thomson, 1895

***pallipes*** Wesmael, 1835 E S W I

*exilis* Haliday, 1837 van Achterberg (1997)

*pallidipes* (Marshall, 1872, *Hypolabis*)

*liopleuris* Thomson, 1895

*piceus* Thomson, 1895

*adaequator* (Fischer, 1964, *Hypolabis*) nom. nud.

*lividipes* (Fischer, 1964, *Hypolabis*) nom. nud.

*subsulcatus* (Fischer, 1964, *Hypolabis*) nom. nud.

*extusus* Papp, 1981

*cisromensis* Papp, 1982

***pendulus*** Haliday, 1837 E I

*latipes* Fischer, 1958

***phytobiae*** Fischer, 1959 E added by Godfray (1986)

***propodealis*** Fischer, 1958 E

***pygmaeator*** (Nees, 1811, *Bracon*) E I

*ruminans* Fischer, 1957

*dilatatus* Fischer, 1960

*meracus* Fischer, 1960

***pygmaeus*** Fischer, 1962 E

***singularis*** Wesmael, 1835 E I

*clarus* Haliday, 1836 Fischer (1997)^^[[284]](#footnote-285)^^

*spretus* Haliday, 1836 Fischer (1997)

*vindex* Haliday, 1837 van Achterberg (in prep.)

*arenosus* Szépligeti, 1898

***soenderupianus*** Fischer, 1967 E added by Godfray & van Achterberg (2015)

***tenellae*** Fischer, 1969 S I

***PHAEDROTOMA*** Förster, 1863^^[[285]](#footnote-286)^^

*Eutrichopsis* Förster, 1863

*Nosopoea* Förster, 1863

*Tolbia* Cameron, 1907

*bRACHYCENTRUS* Szépligeti, 1907

*Coeloreuteus* Roman, 1910

*Hexaulax* Cameron, 1910

*BAEOCENTRUM* Schulz, 1911

*Neodiospilus* Szépligeti, 1911

*nEOPIUS* Fischer, 1965

*EUOPIUS* Fischer, 1967

*Gastrosema* Fischer, 1972

*GERIUS* Fischer, 1972

*GRIMNIRUS* Fischer, 1972

*HOENIRUS* Fischer, 1972

*MEROTRACHYS* Fischer, 1972

*MIMIRUS* Fischer, 1972

*PHLEBOSEMA* Fischer, 1972

*NEOEPHEDRUs* Samanta, Tamili, Saha & Raychaudhuri, 1983

*ADONTOPIUS* Fischer, 1984

*KAINOPAEOPIUS* Fischer, 1987

*MILLENIOPIUS* Fischer, 1996

*NEOTROPOPIUS* Fischer, 1999

***aethiops*** (Haliday, 1837, *Opius*) E I

***caesa*** (Haliday, 1837, *Opius*) E I

*punctiventris* (Thomson, 1895, *Opius*)

*subtilis* (Szépligeti, 1898, *Opius*)

*hydrelliae* (Rimsky-Korsakov, 1925, *Opius*)

*hydrelliae* (Muesebeck, 1933, *Opius*) preocc.

*aquatica* (Muesebeck, 1967, *Opius*)

*hydrelliana* (Fischer, 1971, *Opius*)

***curvata*** (Fischer, 1957, *Opius*) S added by Godfray (1986)

***decorata*** (Stelfox, 1949, *Opius*) I

***depeculator*** (Förster, 1863, *Opius*) E I

*semiaciculata* (Stelfox, 1949, *Opius*)

***diversa*** (Szépligeti, 1898, *Opius*) E^^[[286]](#footnote-287)^^

***exigua*** (Wesmael, 1835, *Opius*) E I

***fallax*** (Szépligeti, 1896, *Opius*) E^^[[287]](#footnote-288)^^

***fasciata*** (Thomson, 1895, *Opius*) E

*comparanda* (Fischer, 1958, *Opius*)

***heringi*** (Fischer, 1962, *Opius*) I

***instabiloides*** (Fischer, 1959, *Opius*) I

***minusculae*** (Fischer, 1967, *Opius*) E

***monticola*** (Szépligeti, 1898, *Opius*) E added by Fischer (1997)

***munda*** (Förster, 1863, *Eutrichopsis*) E

*munda* (Fischer, 1957, *Opius*) preocc.

***nitidulator*** (Nees, 1834, *Bracon*) E

*vittata* (Ruschka, 1915, *Opius*)

***novojariae*** (Fischer, 2006, *Opius*) E added by Fischer (2006)

*stigmatocauda* (Fischer, 2006, *Eurytenes*)

van Achterberg (in prep.)

***paraphytomyzae*** (Fischer, 2005, *Opius*) E added by Fischer (2005)

***pulchriceps*** (Szépligeti, 1898, *Opius*) E I

*ilicis* (Nixon, 1939, *Opius*)

*pulcherrimus* (Fischer, 1958, *Opius*)

*pulchriventris* (Fischer, 1958, *Opius*) van Achterberg (2014)

*vexator* (Fischer, 1964, *Opius*)

*affectus* (Papp, 1981, *Opius*)

***recondes*** van Achterberg, 2004^^[[288]](#footnote-289)^^ added by van Achterberg in Fauna Europaea

*reconditor* misident.

***reptantis*** (Fischer, 1957, *Opius*) E S^^[[289]](#footnote-290)^^

***rex*** (Fischer, 1958, *Opius*) E

***rudiformis*** (Fischer, 1958, *Opius*) E

*uligiloci* (Fischer, 2006, *Opius*) van Achterberg (in prep.)

***staryi*** Fischer, 1958 E S

*sitagrus* Papp, 1982

***tacita*** (Haliday, 1837, *Opius*) E^^[[290]](#footnote-291)^^

***variegata*** (Szepligeti, 1896, *Opius*) E S I

species excluded from the British and Irish list

[***viennensis*** (Fischer, 1959, *Opius*)^^[[291]](#footnote-292)^^]

***RHOGADOPSIS*** Brèthes, 1913^^[[292]](#footnote-293)^^

*LISSOSEMA* Fischer, 1972

***reconditor*** (Wesmael, 1835, *Opius*) E I

*docilis* (Haliday, 1837, *Opius*)^^[[293]](#footnote-294)^^

*parvungula* Thomson, 1895

***UTETES*** Förster, 1863

*Therobolus* Förster, 1863

*FREKIUS* Fischer, 1971 Wharton (2006)

***caudatus*** (Wesmael, 1835, *Opius*) E^^[[294]](#footnote-295)^^

*exsertus* (Thomson, 1895, *Opius*)

***coracinus*** (Thomson, 1895, *Opius*) E BMNH, det. Fischer, added in Fauna Europaea^^[[295]](#footnote-296)^^

***fulvicollis*** (Thomson, 1895, *Opius*) E added by Godfray (1986)

*cupidus* (Gahan, 1919, *Opius*)

***rotundiventris*** (Thomson, 1895, *Opius*) E

***ruficeps*** (Wesmael, 1835, *Opius*) E

***testaceus*** (Wesmael, 1838, *Opius*) E

***truncatus*** (Wesmael, 1838, *Opius*) E

***zelotes*** (Marshall, 1891, *Opius*) E S

*insertus* (Fischer, 1971, *Opius*)

*incertus* misspelling

species excluded from the British and Irish list

[***christenseni*** (Papp, 1982, *Opius*)^^[[296]](#footnote-297)^^]

Subfamily ORGILINAE Ashmead, 1900

***Orgilus*** Haliday, 1833^^[[297]](#footnote-298)^^

*ISCHIUS* Wesmael, 1837

*MACROPALPUS* Ratzeburg, 1844

*ORESIMUS* Ashmead, 1900

*ORGILOMORPHA* Ashmead, 1900

*ISCHIOLUS* Hellén, 1958

***achterbergi*** Taeger, 1989 E added by Taeger (1989)

***dovnari*** Tobias, 1986 E NMS, det. Taeger, added here

*ukrainicus* Tobias, 1986

***interjectus*** Taeger, 1989 E added by Taeger (1989)

***ischnus*** Marshall, 1898 E W

*subtilirugosus* Papp, 1971

***leptocephalus*** Hartig, 1838 I

*obscurator* misident.^^[[298]](#footnote-299)^^

*rugulosus* Fahringer, 1937

*hyperboreus* Hellén, 1958

***minor*** Taeger, 1989 E NMS, det. Taeger, added here

***parvipennis*** Thomson, 1895 E S

*micropterus* Morley, 1907

*macroptera* (Rudow, 1917, *Aptesis*)

*decoratus* Hellén, 1946

*curtipennis* Fischer, 1958

*discolor* Hellén, 1958

***pimpinellae*** Niezabitowski, 1910 E S I added by Taeger (1989)^^[[299]](#footnote-300)^^

*laevigator* misident.

***punctulator*** (Nees, 1811, *Microdus*) E added by Taeger (1989)

*rufiventris* Fahringer, 1937

***rugosus*** (Nees, 1834, *Microgaster*) E NMS, det. Taeger, added here

***tobiasi*** Taeger, 1989 W I added by Taeger (1989)

Subfamily PAMBOLINAE Marshall, 1885

Tribe Chremylini Hellén, 1957

***Chremylus*** Haliday, 1833

*PENECERUS* Wesmael, 1838

*PARAMESOCRINA* Nagamori, 1925

***elaphus*** Haliday, 1833 E I

*rubiginosus* (Nees, 1834, *Hormius*)

*transversus* (Say, 1836, *Bracon*)

*nigriceps* Ashmead, 1893

*terminalis* Ashmead, 1893

*japonicus* Ashmead, 1896

*tineavorus* (Nagamori, 1925, *Paramesocrina*)

Tribe PAMBOLINI Marshall, 1885

***dimeris*** Ruthe, 1854

*PARAPTESIS* Magretti, 1884

***mira*** Ruthe, 1854 E

*melanocephala* (Marshall, 1870, *Pambolus*)

*flavipes* (Magretti, 1884, *Paraptesis*)

*aptera* Marshall, 1885

*inermis* Fitch, 1885

***Pambolus*** Haliday, 1836

Subgenus ***PHAENODUS*** Förster, 1863

***pallipes*** (Förster, 1863, *Phaenodus*) E S added by Belokobylskij (1986)

*flavipes* (Förster, 1863, *Araphis*)

*pallidipes* (Marshall, 1897, *Phaenodus*)

*chalveri* (Docavo, 1960, *Phaenodus*)

species excluded from the British and Irish list

[Subgenus ***Pambolus*** Haliday, 1836

*ARAPHIS* Ruthe, 1854

*ARRHAPHIS* misspelling

*ARHAPHIS* misspelling

*FOLCHINIA* Kieffer, 1906

*PARAMBOLUS* Dahl, 1912

***biglumis*** (Haliday, 1836, *Rogas*)^^[[300]](#footnote-301)^^

*rosenhaueri* (Ratzeburg, 1852, *Pezomachus*)

*dubius* (Fitch, 1885, *Araphis*)

*imminens* (Fitch, 1885, *Araphis*)]

Subfamily Rhysipolinae Belokobylskij, 1984

***PSEUDAVGA*** Tobias, 1964

***flavicoxa*** Tobias, 1964 E added by Shaw & Sims (2015)

*rustus* (Papp, 1991, *Rhysipolis*)

***Rhysipolis*** Förster, 1863^^[[301]](#footnote-302)^^

***decorator*** (Haliday, 1836, *Rogas*) E S M Shaw & Askew (1976)

*ruficeps* (Wesmael, 1838, *Exothecus*)

*ruficornis* (Szépligeti, 1896, *Xenarcha*)

***hariolator*** (Haliday, 1836, *Rogas*) E S Shaw & Askew (1976)

*barbatus* (Wesmael, 1838, *Exothecus*)

***meditator*** (Haliday, 1836, *Rogas*) E S

***variabilis*** (Szépligeti, 1896, *Xenarcha*)^^[[302]](#footnote-303)^^ E NMS, det. Shaw & van Achterberg, added here

***varicoxa*** (Thomson, 1892, *Exothecus*)^^[[303]](#footnote-304)^^ E NMS, det. Shaw & van Achterberg, added here

Subfamily RHYSSALINAE Förster, 1863

HISTEROMERINAE Fahringer, 1930

Tribe ACRISIDINI Hellén, 1957

***Acrisis*** Förster, 1863

*Euchasmus* Marshall, 1888

*EPISIGALPHUS* Ashmead, 1900

***exiguus*** (Marshall, 1888, *Euchasmus*) E

***Proacrisis*** Tobias, 1983^^[[304]](#footnote-305)^^

***acutus*** Tobias, 1983 E NMS, det. Shaw & van Achterberg, added here

***rarus*** Tobias, 1983 E NMS, det. Shaw & van Achterberg, added on Fauna Europaea

Tribe HISTEROMERINI Fahringer, 1930^^[[305]](#footnote-306)^^

***Histeromerus*** Wesmael, 1838

*Mithotynia* Hedqvist, 1976

***mystacinus*** Wesmael, 1838 E W I Shaw (1995); Horniman

*apterus* (Hedqvist, 1976, *Mithotynia*)

Tribe RHYSSALINI Förster, 1863

***Dolopsidea*** Hincks, 1944

*DOLOPS* Marshall, 1889 preocc.

*Exontsira* Belokobylskij, 1982

***indagator*** (Haliday, 1836, *Rogas*) E S I Shaw (1993)

*tuberculata* (Wesmael, 1838, *Exothecus*)

*aculeator* (Marshall, 1889, *Dolops*)

*hastifer* (Marshall, 1889, *Dolops*)

*caucasica* (Tobias, 1976, *Doryctodes*)

*rhodopea* (Zaykov, 1980, *Rhyssalus*)

***Oncophanes*** Förster, 1863

*EPIRHYSSALUS* Ashmead, 1900

***minutus*** (Wesmael, 1838, *Exothecus*) E W S

*lanceolator* (Nees, 1834, *Bracon*) preocc.

*laevigatus* (Ratzeburg, 1852, *Bracon*)^^[[306]](#footnote-307)^^

***Pseudobathystomus*** Belokobylskij, 1986

***funestus*** (Haliday, 1836, *Rogas*)^^[[307]](#footnote-308)^^ E S

*schmiedeknechti* (Fahringer, 1930, *Bathystomus*)

***tobiasi*** (Zaykov, 1980, *Oncophanes*) E S NMS, det. Shaw & van Achterberg, added on Fauna Europaea

***vernalis*** Belokobylskij, 1994 E S NMS, det. Shaw & van Achterberg, added on Fauna Europaea

***Rhyssalus*** Haliday, 1833

*Eurhoptrocentrus* Tobias,1977

***clavator*** Haliday, 1833 E S I M

***longicaudis*** (Tobias & Belokobylskij, 1991, *Eurhoptrocentrus*) W

NMS, det. Shaw & van Achterberg, added on Fauna Europaea

Subfamily ROGADINAE Förster, 1863^^[[308]](#footnote-309)^^

Tribe ALEIODINI Muesebeck, 1928^^[[309]](#footnote-310)^^

***Aleiodes*** Wesmael, 1838^^[[310]](#footnote-311)^^

*ROGAS* misident.

*RHOGAS* misident.

*Petalodes* Wesmael, 1838

*Neorhogas* Szépligeti, 1906

*Chelonorhogas* Enderlein, 1912

***albitibia*** (Herrich-Schäffer, 1838, *Rogas*) E S W I

*heterogaster* Wesmael, 1838

***alternator*** (Nees, 1834, *Rogas*) E S W I

*geniculator* misident.

*balteatus* (Curtis, 1834, *Rogas*)

***apicalis*** (Brullé, 1832, *Bracon*) E to be added by van Achterberg & Shaw (in prep.)^^[[311]](#footnote-312)^^

*ductor* misident., in part

*reticulator* (Nees, 1834, *Rogas*)^^[[312]](#footnote-313)^^

***apiculatus*** (Fahringer, 1932, *Rhogas*) E to be added by van Achterberg & Shaw (in prep.)

***assimilis*** (Nees, 1812, *Bracon*) E S I added by Schwarz & Shaw (2000)

*bicolor* misident., in part

*zygaenae* (Nees, 1834, *Rogas*)

***aterrimus*** (Ratzeburg, 1852, *Bracon*) E

*grandis* Giraud, 1857

***bicolor*** (Spinola, 1808, *Bracon*) E S W I

**sp. near *borealis*** (Thomson, 1892, *Rogas*) E S W I M

to be added by van Achterberg & Shaw (in prep.)

*borealis* misident.^^[[313]](#footnote-314)^^

*circumscriptus* misident., in part

*nigriceps* misident., in part

***cantherius*** (Lyle, 1919, *Rhogas*) E

***circumscriptus*** (Nees, 1834, *Rogas*)^^[[314]](#footnote-315)^^ E S W I

***compressor*** (Herrich-Schäffer, 1838, *Rogas*) E S W

*unicolor* (Wesmael, 1838, *Petalodes*) preocc.

***coxalis*** (Spinola, 1808, *Rogas*) E S M

*ater* (Curtis, 1834, *Rogas*)^^[[315]](#footnote-316)^^

*tristis* Wesmael, 1838

***crassipes*** (Thomson, 1892, *Rogas*) W to be added by van Achterberg & Shaw (in prep.)

***cruentus*** (Nees, 1834, *Rogas*) E

***dissector*** (Nees, 1834, *Rogas*) E S

*aestivalis* (Vollenhoven, 1858, *Phylax*)

***fortipes*** (Reinhard, 1863, *Rogas*) E to be added by van Achterberg & Shaw (in prep.)

*freyi* (Hellén, 1927, *Rhogas*)

***gastritor*** (Thunberg, 1824, *Ichneumon*)^^[[316]](#footnote-317)^^

*circumscriptus* misident., in part

*testaceus* misident., in part^^[[317]](#footnote-318)^^

***grassator*** (Thunberg, 1824, *Ichneumon*) E S

*carbonarius* Giraud, 1857^^[[318]](#footnote-319)^^

*flavipalpis* (Thomson, 1892, *Rogas*)

***heterostigma*** (Stelfox, 1953, *Rogas*) W I

***hirtus*** (Thomson, 1892, *Rogas*)^^[[319]](#footnote-320)^^ E S to be added by van Achterberg & Shaw (in prep.)

***modestus*** (Reinhard, 1863, *Rogas*) E S W

*piceus* (Fahringer, 1932, *Rhogas*)

***nigriceps*** Wesmael, 1838^^[[320]](#footnote-321)^^ E S W I to be added by van Achterberg & Shaw (in prep.)

***nigricornis*** Wesmael, 1838 E S W I

***nobilis*** (Haliday, 1834, *Rogas*)^^[[321]](#footnote-322)^^ E S I

*ductor* misident., in part

*medianus* (Thomson, 1892, *Rogas*) van Achterberg (1997)

***pallidator*** (Thunberg, 1824, *Ichneumon*) E Shaw (1977, 1981)

*ochraceus* (Curtis, 1834, *Rogas*)

*unicolor* Wesmael, 1838

?*pellucens* (Telenga, 1941, *Rhogas*)

***pallidicornis*** (Herrich-Schäffer, 1838, *Rogas*) S^^[[322]](#footnote-323)^^

to be added by van Achterberg & Shaw (in prep.)

*ductor* misident., in part

***pictus*** (Herrich-Schäffer, 1838, *Rogas*) E S W I

to be added by van Achterberg & Shaw (in prep.)

*borealis* misident., in part

*circumscriptus* misident., in part

*nigriceps* misident., in part

***praetor*** (Reinhard, 1863, *Rogas*) E

*luteus* (Szépligeti, 1906, *Neorhogas*)

***pulchripes*** Wesmael, 1838 E I M Shaw (1979*b*)

***punctipes*** (Thomson, 1892, *Rogas*) S W I

***ruficornis*** (Herrich-Schäffer, 1838, *Rogas*) E I

*dimidiatus* misident.

*gasterator* misident.

***rugulosus*** (Nees, 1811, *Bracon*) E S W I

*pictus* (Kokujev, 1898, *Rhogas*) preocc.

***seriatus*** (Herrich-Schäffer, 1838, *Rogas*) E I

*vittiger* Wesmael, 1838

*kuslitzkyi* (Tobias, 1976, *Rogas*)

***signatus*** (Nees, 1812, *Bracon*) E W

*geniculator* (Nees, 1834, *Rogas*)

*annulipes* (Herrich-Schäffer, 1838, *Rogas*)

*esseni* Hellén, 1927

***similis*** (Curtis, 1834, *Rogas*) E S I

*circumscriptus* misident., in part

*testaceus* misident., in part

*spathuliformis* (Curtis, 1834, *Rogas*)

*subucola* (Curtis, 1834, *Rogas*)

*armatus* Wesmael, 1838^^[[323]](#footnote-324)^^

***testaceus*** (Telenga, 1941, *Heterogamus*) E W

to be added by van Achterberg & Shaw (in prep.)

***ungularis*** (Thomson, 1892, *Rogas*) E W I^^[[324]](#footnote-325)^^ to be added by van Achterberg & Shaw (in prep.)

***unipunctator*** (Thunberg, 1824, *Ichneumon*) E S W I

*ductor* (Thunberg, 1824, *Ichneumon*)^^[[325]](#footnote-326)^^

*irregularis* Wesmael, 1838

species excluded from the British and Irish list

[***arcticus*** (Thomson, 1892, *Rogas*)^^[[326]](#footnote-327)^^]

***Heterogamus*** Wesmael, 1838^^[[327]](#footnote-328)^^

***dispar*** (Haliday, 1833, *Rogas*) E S W I

*dispar* (Curtis, 1834, *Rogas*) preocc.

*crypticornis* (Wesmael, 1838, *Aleiodes*)

***excavatus*** Telenga, 1941 E added by Shaw (2000)^^[[328]](#footnote-329)^^

*farmakena* Maláč, 1941

Tribe CLINOCENTRINI van Achterberg, 1991

***Clinocentrus*** Haliday, 1833^^[[329]](#footnote-330)^^

*CAMPTOCENTRUS* Kriechbaumer, 1894

*MICRORHOGAS* Cameron, 1910

*NEORHYSSALUS* Baker, 1917

***brevicalcar*** (Thomson, 1891, *Exothecus*) E S

***cunctator*** (Haliday, 1836, *Rogas*) E S W I

*analis* (Wesmael, 1838, *Exothecus*)

*gracilipes* (Thomson, 1892, *Exothecus*)

***excubitor*** (Haliday, 1836, *Rogas*) E S W I

*marginellus* (Wesmael, 1838, *Exothecus*)

***exsertor*** (Nees, 1811, *Bracon*) E S

*orbitator* (Nees, 1834, *Bracon*)

*striolatus* (Thomson, 1891, *Exothecus*)

*tarsalis* Ashmead, 1894

***hungaricus*** Szépligeti, 1906 E S NMS, BMNH, det. Shaw, added here

***umbratilis*** Haliday, 1833 E S I

*petiolaris* (Thomson, 1891, *Exothecus*)

*polonicus* Fahringer, 1931

***vestigator*** (Haliday, 1836, *Rogas*) E S I M

*stigmaticus* Marshall, 1897

*jaroshevskyi* Telenga, 1941

*obsoletus* (Hellén, 1957, *Oncophanes*)

species excluded from the British and Irish list

[***tenuicornis*** (Thomson, 1891, *Exothecus*)^^[[330]](#footnote-331)^^]

Tribe ROGADINI Förster, 1863

PELECYSTOMINI Viereck, 1918

***Rogas*** Nees, 1818

*Pelecystoma* Wesmael, 1838

*RHOGAS* Agassiz, 1846

***luteus*** Nees, 1834

*testaceus* (Fabricius, 1798, *Ichneumon*) preocc.

*testaceator* (Thunberg, 1824, *Ichneumon*)

species excluded from the British and Irish list

[***Triraphis*** Ruthe, 1855

***tricolor*** (Wesmael, 1838, *Pelecystoma*)^^[[331]](#footnote-332)^^

*solitarius* (Watanabe, 1970, *Pelecystoma*) Papp (1995*b*)]

Subfamily SIGALPHINAE Haliday, 1833

Tribe ACAMPSINI van Achterberg & Austin, 1992

***Acampsis*** Wesmael, 1835

***alternipes*** (Nees, 1816, *Sigalphus*) E

species excluded from the British and Irish list

[Tribe SIGALPHINI Haliday, 1833

***Sigalphus*** Latreille, 1802

*SPHAEROPYX* Illiger, 1807

*Rhitigaster* Wesmael, 1835

*Rhytidogaster* Agassiz, 1846

***irrorator*** (Fabricius, 1775, *Ichneumon*)^^[[332]](#footnote-333)^^

*niger* (Retzius, 1783, *Ichneumon*)

*globulifer* (Geoffroy, 1785, *Ichneumon*)

*irroratrix* (Schulz, 1906, *Sphaeropyx*)]

**References**

Achterberg, C. van. 1975. About the identity of *Biosteres* (*Biosteres*) *impressus* (Wesmael) (Braconidae, Opiinae). *Entomologische Berichten* **35**: 175-176.

Achterberg, C. van. 1979. A revision of the subfamily Zelinae *auct*. (Hymenoptera, Braconidae). *Tijdschrift voor Entomologie* **122**: 241-479.

Achterberg, C. van. 1984*a*. Addition to the revision of the genus *Zele* Curtis (Hymenoptera: Braconidae). *Entomologische Berichten* **44**: 110-112.

Achterberg, C. van. 1984*b*. Essay on the phylogeny of Braconidae (Hymenoptera: Ichneumonoidea). *Entomologisk Tidskrift* **105**: 41-58.

Achterberg, C. van. 1985. The genera and subgenera of Centistini, with description of two new taxa from the Nearctic Region (Hymenoptera: Braconidae: Euphorinae). *Zoologische Mededelingen* **59**: 348-362.

Achterberg, C. van. 1987. Revision of the European Helconini (Hymenoptera, Braconidae: Helconinae). *Zoologische Mededelingen* **61**: 263-285.

Achterberg, C. van. 1988*a*. Revision of the subfamily Blacinae Foerster (Hymenoptera, Braconidae). *Zoologische Verhandelingen* **249**: 1-324.

Achterberg, C. van. 1988*b*. The genera of the *Aspilota*-group and some descriptions of fungicolous Alysiini from the Netherlands (Hymenoptera: Braconidae: Alysiinae). *Zoologische Verhandelingen* **247**: 1-88.

Achterberg, C. van. 1989. Revision of the subtribe Monoctonina Mackauer sensu stricto (Hymenoptera: Braconidae: Aphidiinae). *Zoologische Mededelingen* **63**: 1-22.

Achterberg, C. van. 1990. Revision of the Western Palaearctic Phanerotomini (Hymenoptera: Braconidae). *Zoologische Verhandeligen* **255**: 1-106.

Achterberg, C. van. 1992*a*. Revision of the European species of the genus *Pygostolus* Haliday (Hymenoptera: Braconidae: Euphorinae), with a key to the Holarctic species. *Zoologische Mededelingen* **66**: 349-358.

Achterberg, C. van 1992*b*. Revisionary notes on the subfamily Homolobinae (Hymenoptera: Braconidae). *Zoologische Mededelingen* **66**: 359-368.

Achterberg, C. van 1993*a*. Illustrated key to the subfamilies of the Braconidae (Hymenoptera: Ichneumonoidea). *Zoologische Verhandeligen* **283**: 1-189.

Achterberg, C. van 1993*b*. Revision of the subfamily Macrocentrinae Foerster (Hymenoptera: Braconidae) from the Palaearctic region. *Zoologische Verhandelingen* **286**: 1-110.

Achterberg, C. van. 1995. New combinations of names for Palaearctic Braconidae (Hymenoptera). *Zoologische Mededelingen* **69**: 131-138.

Achterberg, C. van. 1997. Revision of the Haliday collection of Braconidae (Hymenoptera). *Zoologische Verhandelingen* **314**: 1-115.

Achterberg, C. van. 1998. *Phaenocarpa helophilae* spec. nov. (Hymenoptera: Braconidae), a gregarious parasitoid of *Helophilus* larvae (Diptera: Syrphidae), with notes on related species. *Zoologische Mededelingen* **72**: 15-27.

Achterberg, C. van. 2000. The European species of the *Eubazus aliochinoi*-group (Hymenoptera: Braconidae: Helconinae: Brachistini). *Zoologische Mededelingen* **74**: 339-357.

Achterberg, C. van. 2003*a*[2002]. Western Palaearctic genera of the subfamily Microgastrinae: a re-appraisal of the generic and tribal division (Hymenoptera: Braconidae). Pp. 19-35 in: Melika, G. & Thuróczy, C., eds. *Parasitic wasps: Evolution, Systematics, Biodiversity and Biological Control*. Agroinform, Budapest, Hungary.

Achterberg, C. van. 2003*b*. The West Palaearctic species of the genera *Gildoria* Hedqvist and *Platyspathius* Viereck, with keys to the species (Hymenoptera: Braconidae: Doryctinae). *Zoologische Mededelingen* **77**: 267-290.

Achterberg, C. van. 2003*c*. The European species of the subgenus *Aliolus* Say of the genus *Eubazus* Nees and of the genus *Dicyrtaspis* van Achterberg (Hymenoptera: Braconidae: Brachistinae). *Zoologische Mededelingen* **77**: 301-320.

Achterberg, C. van. 2004*a*. New Indo-Australian subgenera and species of the genera *Xynobius* Foerster and *Ademoneuron* Fischer (Hymenoptera: Braconidae: Opiinae). *Zoologische Mededelingen* **78**: 313-329.

Achterberg, C. van. 2004*b*. *Bitomoides* gen. nov. (Hymenoptera: Braconidae: Opiinae) from Europe. *Zoologische Mededelingen* **78**: 331-335.

Achterberg, C. van. 2011. Order Hymenoptera, family Braconidae. The subfamily Agathidinae from the United Arab Emirates, with a review of the fauna of the Arabian Peninsula. *Arthropod fauna of the UAE* **4**: 286-352.

Achterberg, C. van & Broad, G. R. 2013. Revision of the genus *Vadumasonium* Kammerer (Hymenoptera, Braconidae, Brachistinae). *Journal of Hymenoptera Research* **33**: 91-98.

Achterberg, C. van & Haeselbarth, E. 1983. Revisionary notes on the European species of *Macrocentrus* Curtis *sensu stricto* (Hymenoptera: Braconidae). *Entomofauna* **4**: 37-59.

Achterberg, C. van & Haeselbarth, E. 2003. Revision of the genus *Syntretus* Foerster (Hymenoptera: Braconidae: Euphorinae) from Europe. *Zoologische Mededelingen* **77**: 9-78.

Achterberg, C. van & Long, K. D. 2010. Revision of the Agathidinae (Hymenoptera, Braconidae) of Vietnam, with the description of forty-two new species and three new genera. *ZooKeys* **54**: 1-184.

Achterberg, C. van & O’Connor, J. P. 1990. Revision of the Palaearctic genus *Trachyusa* Ruthe (Hymenoptera: Braconidae). *Zoologische Mededelingen* **64**: 107-112.

Achterberg, C. van & Polaszek, A. 1996. The parasites of cereal stem borers (Lepidoptera: Cossidae, Crambidae, Noctuidae, Pyralidae) in Africa, belonging to the family Braconidae (Hymenoptera: Ichneumonoidae). *Zoologische Verhandeligen* **304**: 1-123.

Achterberg, C. van & Salvo, A. 1997. Reared Opiinae (Hymenoptera: Braconidae) from Argentina. *Zoologische Mededelingen* **71**: 189-214.

Achterberg, C. van & Shaw, M. R. 2008. A new subgenus of the genus *Colastes* Haliday (Hymenoptera: Braconidae: Exothecinae) for species reared from bracket fungi, with description of two new species from Europe. *Journal of Natural History* **42**: 1849-1860.

Achterberg, C. van & Vikberg, V. 2014. *Dinotrema brevis* spec. nov. (Hymenoptera: Braconidae: Alysiinae), a new brachypterous species from Finland. *Zoologische Mededelingen* **88**: 1-7.

Allen, A. A. 1978. The discovery of *Apanteles gades* Nixon (Hymenoptera: Braconidae) in Britain. *Entomologist’s Gazette* **29**: 102.

Baker, E. A. 2013. Aphid parasitoids in Wales. *British Journal of Entomology and Natural History* **26**: 219-239.

Baker, E. A. & Broad, G. R. 2009. Five aphid parasitoids (Hymenoptera: Braconidae: Aphidiinae) new to Britain. *British Journal of Entomology and Natural History* **22**: 255-263.

Belokobylskij, S. A. 1986. Obzor vidov rodov *Pambolus* Hal. i *Dimeris* Ruthe (Hymenoptera, Braconidae) Palearktiki [A review of the Palaearctic species of the genera *Pambolus* Hal. and *Dimerus* Ruthe (Hymenoptera, Braconidae)]. *Trudy Zoologicheskogo Instituta. Leningrad* **159**: 18-37.

Belokobylskij, S. A. 1992. [On the classification and phylogeny of braconid wasps subfamilies Doryctinae and Exothecinae (Hymenoptera, Braconidae). I. Classification.] *Entomologicheskoe Obozrenie* 71: 900-928 (in Russian); English translation, 1993. *Entomological Review* **72**: 109-137.

Belokobylskij, S. A. 1998. 1. Rhyssalinae, 2. Doryctinae, 3. Histeromerinae, 4. Exothecinae, 7. Gnamptodontinae, 9. Alysiinae (Alysiini), 10. Helconinae, 11. Cenocoeliinae, 12. Brachistinae, 14. Meteorideinae, 16. Xiphozelinae, 17. Homolobinae, 18. Charmontinae, 19. Orgilinae, 20. Ecnomiinae, 21. Sigalphinae, 23. Ichneutinae, 25. Cardiochilinae, 27. Dirrhopinae, 28. Miracinae, 29. Adeliinae. In: Ler, P.A. '*Key to the insects of Russian Far East. Vol. 4. Neuropteroidea, Mecoptera, Hymenoptera. Pt 3*.' Dal'nauka, Vladivostok. 706 pp. pp.41-162, 163-298, 411-520, 531-558.

Belokobylskij, S. A. 2000*a*. Braconidae. In: Ler, P. A., ed. [Key to the insects of Russian Far East. Vol. IV. Neuropteroidea, Mecoptera, Hymenoptera. Pt 4.] Opredelitel nasekomykh Dalnego Vostoka Rossii. T. IV. Setchatokryloobraznye, skorpionnitsy, pereponchatokrylye. Ch. 4. Dalnauka, Vladivostok. 651 pp.

Belokobylskij, S. A. 2000*b*. On the Asian species of the genus *Streblocera* Westwood, 1833 (Hymenoptera: Braconidae, Euphorinae) with a key to the eastern Palaearctic species (continuation). *Entomologische Zeitschrift* **110**: 290-297

Belokobylskij, S. A. & Samartsev, K. G. 2014. Palaearctic species of the *Spathius exarator* species group (Hymenoptera: Braconidae: Doryctinae) with entirely sculptured mesopleuron. *Zootaxa* **3900**: 483-504.

Belokobylskij, S. A. & Tobias, V. I. 1986. Doryctinae. Pp. 21-72 in: Medvedev, G. S., ed. '*Opredelitel Nasekomych Evrospeiskoi Tsasti SSSR 3, Peredpontdatokrylye 4. Opr. Faune SSSR*.' **145**:1-501.

Belokobylskij, S. A., Taeger, A., van Achterberg, C., Haeselbarth, E. & Riedel, M. 2003. Checklist of the Braconidae of Germany. *Beiträge zur Entomologie* **53**: 341-435.

Belshaw, R. & Quicke, D. L. J. 1997. A molecular phylogeny of the Aphidiinae (Hymenoptera: Braconidae). *Molecular Phylogenetics and Evolution* **7**: 281-293.

Belshaw, R. & Quicke, D. L. J. 2002. Robustness of ancestral state estimates: evolution of life history strategy in ichneumonoid parasitoids. *Systematic Biology* **51**: 450-477.

Belshaw, R., Dowton, M., Quicke, D. L. J. & Austin, A. D. 2000. Estimating ancestral geographical distributions: a Gondwanan origin for aphid parasitoids? *Proceedings of the Royal Society of London series B, Biology* **267**: 491-496.

Belshaw, R., Lopez-Vaamonde, C., Degerli, N. & Quicke, D. L. J. 2001. Paraphyletic taxa and taxonomic chaining: evaluating the classification of braconine wasps (Hymenoptera: Braconidae) using 28S D2-3 rDNA sequences and morphological characters. *Biological Journal of the Linnean Society* **73**: 411-424.

Broad, G. R. 2009. *Syntretus breviradialis* van Achterberg & Haeselbarth (Hymenoptera: Braconidae), new to Britain. *British Journal of Entomology and Natural History* **22**: 253.

Čapek, M. & van Achterberg, C. 1992. A revision of the genus *Microtypus* Ratzeburg (Hymenoptera: Braconidae). *Zoologische Mededelingen* **66**: 323-338.

Chen, X. & van Achterberg, C. 1997. Revision of the subfamily Euphorinae (excluding the tribe Meteorini Cresson) (Hymenoptera: Braconidae) from China. *Zoologische Verhandelingen* **313**: 1-217.

Curtis, J. 1837. *A guide to an arrangement of British insects; being a catalogue of all the named species hitherto discovered in Great Britain and Ireland. Second edition, greatly enlarged*. London.

Disney, R. H. L. & Munk, T. 2005. The scuttle fly host *Megaselia giraudii* (Egger) (Diptera: Phoridae) and the first British records of its parasitoid *Dinotrema necrophilum* (Hedqvist, 1972) (Hymenoptera: Braconidae). *Entomologist's Gazette* **56**: 105-106.

Dowton, M., Belshaw, R., Austin, A. D. & Quicke, D. L. J. 2002. Simultaneous molecular and morphological analysis of braconid relationships (Insecta: Hymenoptera: Braconidae) indicates independent mt-tRNA gene inversions within a single wasp family. *Journal of Molecular Evolution* **54**: 210-226.

Eady, R. D. & Clark, J. A. J. 1964. A revision of the genus *Macrocentrus* Curtis (Hym., Braconidae) in Europe, with descriptions of four new species. *Entomologist's Gazette* **15**: 97-127.

Elliot, E. A. & Morley, C. 1911. On the hymenopterous parasites of Coleoptera. First Supplement. *Transactions of the Entomological Societyof London* **1911**: 452-496.

Enobakhare, D. A. 2001. Description and a key to *Pauesia* spp. (Hymenoptera: Aphidiidae) attacking conifer associated lachnids (Homoptera: Aphidoidea: Lachnidae) in Britain. *Applied Tropical Agriculture* **6**: 98-106.

Faure, J. C. 1924. Observations biologiques sur *Bracon glaphyrus* Marsh. *Revue de Pathologie Vegetale et d'Entomologie Agricole de France* **11**: 70-72.

Fernandez-Triana, J. 2015. A revision of the genus *Protomicroplitis* Ashmead (Hymenoptera, Braconidae, Microgastrinae), with the description of a new species. *Zootaxa* **4039**: 529-542.

Fischer, M. 1958. Die europäischen Arten der Gattung *Opius* Wesm. Teil 1b (Hymenoptera, Braconidae). *Annali del Museo Civico di Storia Naturale di Genova* **70**: 245-304.

Fischer, M.1960. Die europäischen Arten der Gattung *Opius* Wesmael, Teil IVa. *Annales Zoologici*. *Warszawa*. **19**: 33-112.

Fischer, M. 1967. Über gezüchtete Opiinae aus Europa (Hymenoptera, Braconidae). *Zeitschrift für Angewandte Entomologie* **60**: 318-350

Fischer, M. 1972. Hymenoptera Braconidae (Opiinae I). (Paläarktische Region). *Das Tierreich* **91**(1973):1-620.

Fischer, M. 1980. Fünf neue Raupenwespen (Hymenoptera, Braconidae). *Frustula Entomologica* **1**(1978): 147-160.

Fischer, M. 1997. Die paläarktischen Opiinae (Madenwespen) der Zoologischen Staatssammlung München (Hymenoptera, Braconidae). *Entomofauna* **18**: 137-196.

Fischer, M. 2005. Some new Opiinae (Insecta: Hymenoptera: Braconidae) in the Natural History Museum Vienna. *Annalen des Naturhistorischen Museums in Wien* **106B**: 107-133.

Fischer, M. 2006. New species of Opiinae (Insecta: Hymenoptera: Braconidae) in the Collection of the National Museums of Scotland, Edinburgh. *Annalen des Naturhistorischen Museums in Wien* **107B**: 131-144.

Fischer, M. & Koponen, M. 1999. A survey of Opiinae (Hymenoptera, Braconidae) of Finland, part 1. *Entomologica Fennica* **10**: 65-93.

Fischer, M., Tormos, J., Docavo, I. & Pardo, X. 2004. A new species of *Antrusa* and three new species of *Chorebus* (Hymenoptera: Braconidae) from the Iberian Peninsula. *Florida Entomologist* **87**: 306-311.

Fitton, M. G., Graham, M. W. R. de V., Bouček, Z. R. J., Fergusson, N. D. M., Huddleston, T., Quinlan, J. & Richards, O. W. 1978. Kloet and Hincks. A check list of British insects. Part 4: Hymenoptera. *Handbooks for the Identification of British Insects* **11**: ix + 159 pp.

Fulmek, L. 1968. Parasitinsekten der Insektengallen Europas. *Beiträge zur Entomologie* **18**: 719-952.

Gärdenfors, U. 1986. Taxonomic and biological revision of Palearctic *Ephedrus* Haliday (Hymenoptera: Braconidae, Aphidiinae). *Entomologica Scandinavica* Supplement **27**: 1-95.

Godfray, H. C. J. 1984. Intraspecific variation in the leaf-miner parasite *Exotela cyclogaster* Forster (Hymenoptera: Braconidae). *Proceedings and Transactions of the British Entomological and Natural History Society* **17**: 47-50.

Godfray, H. C. J. 1986. Four species of *Opius* (Hym., Braconidae) new to Britain. *Entomologist's Monthly Magazine* **122**: 127.

Godfray, H. C. J. 1988. *Biosteres spinaciae* (Thomson) (Hym., Braconidae, Opiinae), new to Britain. *Entomologist's Monthly Magazine* **124**: 251-252.

Godfray, H. C. J. & Bland, K. P. 2011. Three species of parasitoid wasp (Braconidae, Alysiinae) new to Britain. *British Journal of Entomology and Natural History* **24**: 127-131.

Godfray, H. C. J. & McGavin, G. C. 1985. *Colastes pubicornis* (Thomson) (Hym., Braconidae, Exothecini) new to Britain, with a first host record. *Entomologist’s Monthly Magazine* **121**: 109-110.

Griffiths, G. C. D. 1964. The Alysiinae (Hym. Braconidae) parasites of the Agromyzidae (Diptera) I. General questions of taxonomy, biology and evolution. *Beiträge zur Entomologie* **14**: 823-914.

Griffiths, G. C. D. 1967*a*. The Alysiinae (Hym. Braconidae) parasites of the Agromyzidae (Diptera) II. The parasites of *Agromyza* Fallén. *Beiträge zur Entomologie* **16** (1966): 551-605.

Griffiths, G. C. D. 1967*b*. The Alysiinae (Hym. Braconidae) parasites of the Agromyzidae (Diptera) III. The parasites of *Paraphytomyza* Enderlein, *Phytagromyza* Hendel, and *Phytomyza* Fallén. *Beiträge zur Entomologie* **16** (1966): 775-951.

Griffiths, G. C. D. 1967*c*. The Alysiinae (Hym. Braconidae) parasites of the Agromyzidae (Diptera) IV. The parasites of *Hexomyza* Enderlein, *Melanagromyza* Hendel, *Ophiomyia* Braschnikov and *Napomyza* Westwood. *Beiträge zur Entomologie* **17**: 653-696.

Griffiths, G. C. D. 1968*a*. The Alysiinae (Hym. Braconidae) parasites of the Agromyzidae (Diptera) V. The parasites of *Liriomyza* Mik and certain small genera of Phytomyzinae. *Beiträge zur Entomologie* **18**: 5-62.

Griffiths, G. C. D. 1968*b*. The Alysiinae (Hym. Braconidae) parasites of the Agromyzidae (Diptera) VI. The parasites of *Cerodontha* Rondani s.l. *Beiträge zur Entomologie* **18**: 63-152.

Griffiths G. C. D. 1984. The Alysiinae (Hym. Braconidae) parasites of the Agromyzidae (Diptera). VII. Supplement. *Beiträge zur Entomologie* **34**: 343-362.

Haeselbarth, E. 1973. Die *Blacus*-Arten Europas und Zentral-Asiens. *Veröffentlichungen der Zoologischen Staatssammlung München* **16**: 69-170.

Haeselbarth, E. 1988. Zur Braconidengattung *Townesilitus* Haeselbarth & Loan, 1983. *Entomofauna* **9**: 429-460.

Haeselbarth, E. 1996. *Rilipertus* gen. nov., eine neue Gattung der Euphorinae (Hymenoptera, Braconidae). *Entomofauna* **17**: 397-412.

Haeselbarth, E. 1999. Zur Braconiden-Gattung *Perilitus* Nees, 1818 2. Beitrag: Die Arten mit ausgebildetem ersten Cubitus-Abschnitt. *Mitteilungen der Münchener Entomologischen Gesellschaft* **89**: 11-46.

Haeselbarth, E. 2008. Zur Braconiden-Gattung *Perilitus* Nees 1818 3. Beitrag: Die Arten ohne ausgebildetem ersten Cubitus-Abschnitt (Hymenoptera, Braconidae). *Linzer Biologische Beiträge* **40**: 1013-1152.

Huddleston, T. 1978. Braconidae and Aphidiidae. Pp. 46-62 *in*: Fitton, M. G., Graham, M. W. R. de V., Bouček, Z. R. J., Fergusson, N. D. M., Huddleston, T., Quinlan, J. & Richards, O. W. 1978. Kloet and Hincks. A check list of British insects. Part 4: Hymenoptera. *Handbooks for the Identification of British Insects* **11**: ix + 159 pp.

Huddleston, T. 1980. A revision of the western Palaearctic species of the genus *Meteorus* (Hymenoptera: Braconidae). *Bulletin of the British Museum (Natural History)(Entomology)* **41**: 1-58.

Huddleston, T. 1984. The Palearctic species of *Ascogaster* (Hymenoptera: Braconidae). *Bulletin of the British Museum (Natural History), Entomology* **49**: 341-392.

Hussey, N. W. 1952. A Braconid (Hym.) parasite of *Ochina ptinoides* Marsh. (Col., Anobiidae). *Entomologist's Monthly Magazine* **88**: 201.

International Commission on Zoological Nomenclature. 1988. Opinion 1510. *Microgaster* Latreille, 1804 (Insecta, Hymenoptera): *Microgaster australis* Thomson, 1895 designated as the type species. *Bulletin of Zoological Nomenclature* 45: 239-240.

Jennings, M. T. 2008. A record of *Baryproctus barypus* (Marshall) (Hym., Braconidae) as a parasitoid of *Lipara rufitarsis* Loew (Dipt., Chloropidae) from Kent, U.K. *Entomologist’s Monthly Magazine* **144**: 192.

Jennings, M. T. 2012. *Bracon leptus* Marshall, 1897 (Hymenoptera:Braconidae) reared in Britain. *Entomologist's Monthly Magazine* **148**: 54.

Kavallieratos, N. G., Tomanovic, Z., Starý, P., Athanassiou, C. G., Fasseas, C., Petrovic, O. Stanisavljevic, L. Z. & Veroniki, M. A. 2005. *Praon* Haliday (Hymenoptera: Braconidae: Aphidiinae) of southeastern Europe: key, host range and phylogenetic relationships. *Zoologischer Anzeiger* **243**: 181-209.

Kerrich, G. J. 1932. Additions to the Ichneumonoid fauna of Wicken Fen. In: *Natural History of Wicken Fen*. Cambridge. **6**: 560-566.

Kloet, G. S. & Hincks, W. D. 1945. *A check list of British insects*. Stockport.

König, R. 1972. Zur Systematik, Faunistik, Phänologie und Ökologie mitteleuropäischer Braconiden (Hymenoptera) (1). *Faunistisch-Ökologische Mitteilungen* **4**: 85-106.

Kotenko, A. G. & Tobias, V. I. 1986. Subfamily Microgastrinae. Pp. 344–459 in: Tobias, V. I., editor. *Keys to the Insects of the European part of the USSR 3. Hymenoptera. Part 4*. Leningrad [In Russian. English translation 1995, pp. 605–816. Science Publishers.]

Kula, R. R. 2008. Taxonomic status and location of type specimens for species of *Coelinidea* Viereck and *Sarops* Nixon (Hymenoptera: Braconidae: Alysiinae) described by Garland T. Riegel. *Journal of Hymenoptera Research* **17**: 138–156.



Laing, F. 1928. A note on two parasites of *Lyctus parallelopipedus* Mel. *Entomologist* **61**: 163-164.

Li, X.-Y., van Achterberg, C. & Tan, J.-C. 2013. Revision of the subfamily Opiinae (Hymenoptera, Braconidae) from Hunan (China), including thirty-six new species and two new genera. *ZooKeys* **268**: 1-186.

Loan, C. C. 1974. The European species of *Leiophron* Nees and *Peristenus* Foerster (Hymenoptera: Braconidae, Euphorinae). *Transactions of the Royal Entomological Society of London* **126**: 207-238.

Loan, C. C. 1976. *Peristenus malatus* (Hymenoptera: Braconidae, Euphorinae) a new species, parasitic on *Blepharidopterus angulatus* (Fallén) (Heteroptera: Miridae) in Britain. *Le Naturaliste Canadien* **103**: 437-440.

Luff, M. L. 1976*a*. *Centistes nasutus* (Wesmael) (Hym., Braconidae) parasitizing *Amara apricaria* Payk. (Col., Carabidae). *Entomologist's Monthly Magazine* **112**: 40.

Luff, M. L. 1976*b*. The biology of *Microctonus caudatus* (Thomson) a braconid parasite of the ground beetle *Harpalus rufipes* (Degeer). *Ecological Entomology* **1**: 111-116.

Lyle, G. T. 1933. A catalogue of the British Braconidae. *Transactions of the Royal Entomological Society of London* **81**: 67-74.

Mackauer, M. 1961. Die Typen der Unterfamilie Aphidiinae des Britischen Museums London (Hymenoptera: Braconidae). *Beiträge zur Entomologie* **11**: 96-154.

Mancini, D., Priore, R., Battaglia, D. & van Achterberg, C. 2003. *Caenopachys hartigii* (Ratzeburg) (Hymenoptera: Braconidae: Doryctinae) confirmed for Italy, with notes on the status of the genus *Caenopachys* Foerster. *Zoologische Mededelingen* **77**: 459-470.

Marshall, T. A. 1872. *A catalogue of British Hymenoptera; Chrysididae, Ichneumonidae, Braconidae, and Evanidae*. The Entomological Society of London.

Marshall, T. A. 1885. Monograph of British Braconidae. Part I. *Transactions of the Entomological Society of London* **1885**: 1-280.

Marshall, T. A. 1896. Les Braconides. In: André, E., ed. *Species des Hymenopteres d'Europe et d'Algerie* Tome 5. 635 pp. Gray 1891.

Mason, W. R. M. 1981. The polyphyletic nature of *Apanteles* Foerster (Hymenoptera, Braconidae) - a phylogeny and reclassification of Microgastrinae. *Memoirs of the Entomological Society of Canada* **115**: 1-147.

Morley, C. 1913. On Walker's Japanese Ichneumonidae. *Entomologist* **46**: 131-135.

Morley, C. & Rait-Smith, W. 1933. The Hymenopterous parasites of the British Lepidoptera. *Transactions of the Royal Entomological Society of London* **81**: 133-183.

Müller, C. B., Adriaanse, I. C. T., Belshaw, R. & Godfray, H. C. J. 1999. The structure of an aphid-parasitoid community. *Journal of Animal Ecology* **68**: 346-370.

Munk, T., Peris-Felipo, F. J. & Jiménez-Peydró, R. 2013. New western Palaearctic *Dinotrema* species with mesoscutal pit and only medially sculptured propodeum (Hymenoptera, Braconidae, Alysiinae). *ZooKeys* **260**: 61-76.

Murray, J. 1939. *Heterogamus dispar* Curtis in Dumfriesshire. *Entomologist* **72**: 170.

New, T. R. 1970. The life histories of two species of *Leiophron* Nees (Hymenoptera, Braconidae) parasitic on Psocoptera in southern England. *Entomologist's Gazette* **21**:39-48.

Nixon, G. E. J. 1937. British species of *Dacnusa* (Braconidae). *Transactions of the Society for British Entomology* **4**: 1-88.

Nixon, G. E. J. 1943. A revision of the European Dacnusini (Hym., Braconidae, Dacnusinae). *Entomologist's Monthly Magazine* **79**: 20-34, 159-168.

Nixon, G. E. J. 1944. A revision of the European Dacnusini (Hym., Braconidae, Dacnusinae). *Entomologist's Monthly Magazine* **80**: 88-108, 140-151, 193-200, 249-255.

Nixon, G. E. J. 1945. A revision of the European Dacnusini (Hym., Braconidae, Dacnusinae). *Entomologist's Monthly Magazine* **81**: 189-204, 217-229.

Nixon, G. E. J. 1946. A revision of the European Dacnusini (Hym., Braconidae, Dacnusinae). *Entomologist's Monthly Magazine* **82**: 279-300.

Nixon, G. E. J. 1948. A revision of the European Dacnusini (Hym., Braconidae, Dacnusinae). *Entomologist's Monthly Magazine* **84**: 207-224.

Nixon, G. E. J. 1949. A revision of the European Dacnusini (Hym., Braconidae, Dacnusinae). *Entomologist's Monthly Magazine* **85**: 289-298.

Nixon, G. E. J. 1954. A revision of the European Dacnusini (Hym., Braconidae, Dacnusinae). *Entomologist's Monthly Magazine* **90**: 257-290.

Nixon, G. E. J. 1965. A reclassification of the tribe Microgasterini (Hymenoptera : Braconidae). *Bulletin of the British Museum (Natural History), Entomology* **Supplement 2**: 1-284.

Nixon, G. E. J. 1968. A revision of the genus *Microgaster* Latreille (Hymenoptera : Braconidae). *Bulletin of the British Museum (Natural History), Entomology* **22**: 31-72.

Nixon, G. E. J. 1970. A revision of the N.W. European species of *Microplitis* Förster (Hymenoptera: Braconidae). *Bulletin of the British Museum (Natural History), Entomology* **25**: 1-30.

Nixon, G. E. J. 1972. A revision of the north-western European species of the *laevigatus*-group of *Apanteles* Förster (Hymenoptera, Braconidae). *Bulletin of Entomological Research* **61**: 701-743.

Nixon, G. E. J. 1973. A revision of the north-western European species of the *vitripennis, pallipes, octonarius, triangulator, fraternus, formosus, parasitellae, metacarpalis* and *circumscriptus*-groups of *Apanteles* Förster (Hymenoptera, Braconidae). *Bulletin of Entomological Research* **63**: 169-228.

Nixon, G. E. J. 1974. A revision of the north-western European species of the *glomeratus*-group of *Apanteles* Förster (Hymenoptera, Braconidae). *Bulletin of Entomological Research* **64**: 453-524.

Nixon, G. E. J. 1976. A revision of the north-western European species of the *merula*, *laeteus*, *vipio*, *ultor*, *ater*, *butalidis*, *popularis*, *carbonarius* and *validus*-groups of Apanteles Förster (Hym.: Braconidae). *Bulletin of Entomological Research* **65**: 687-732.

Nixon, G. E. J. 1986. A revision of the European Agathidinae (Hymenoptera: Braconidae). *Bulletin of the British Museum (Natural History), Entomology* **52**: 183-242.

Notton, D. G. 1991. *Aspilota intermediana* Fischer a fungicolous braconid (Hym.) new to Britain. *Entomologist's Monthly Magazine* **127**: 159-160.

Notton, D. G., Popovici, O. A., Achterberg, C. van, De Rond, J. & Burn, J. T. 2014. Parasitoid wasps new to Britain (Hymenoptera: Platygastridae, Eurytomidae, Braconidae & Bethylidae). *European Journal of Taxonomy* **99**: 1-20.

O'Connor, J. P., Nash, R. & van Achterberg, C. 1999. A catalogue of the Irish Braconidae (Hymenoptera: Ichneumonoidea). *Occasional Publications of the Irish Biogeographical Society* **4**: 1-90.

Papp, J. 1972. New *Apanteles* Först. species from Hungary (Hymenoptera, Braconidae: Microgasterinae), I. *Annales Historico-naturales Musei Nationalis Hungarici* **64**: 335-345.

Papp, J. 1974. Zur Kenntnis der *Bracon*-Arten Österreichs (Hymenoptera, Braconidae). *Annales Historico-naturales Musei Nationalis Hungarici* **78**: 415-435.

Papp, J. 1976. Key to the European *Microgaster* Latr. Species, with a new species and taxonomical remarks (Hymenoptera: Braconidae, Microgasterinae). *Acta Zoologica Academiae Scientiarum Hungaricae* **22**: 97-117.

Papp, J. 1978. A survey of the European species of *Apanteles* Först. (Hymenoptera, Braconidae: Microgasterinae), II. The *laevigatus*-group, 1. *Annales Historico-Naturales Musei Nationalis Hungarici* **70**: 265-301.

Papp, J. 1981. A survey of the European species of *Apanteles* Först. (Hymenoptera, Braconidae: Microgastrinae), V. The *lacteus*-, *longipalpis*-, *ultor*-, *butalidis*- and *vipio*-group. *Annales Historico-Naturales Musei Nationalis Hungarici* **73**: 263-291.

Papp, J. 1983. A survey of the European species of *Apanteles* Först. (Hymenoptera, Braconidae: Microgastrinae), VII. The *carbonarius*-, *circumscriptus*-, *fraternus*-, *pallipes*-, *parasitellae*-, *vitripennis*-, *liparidis*-, *octonarius* and *thompsoni*-group. *Annales Historico-naturales Musei Nationalis Hungarici* **75**: 247-283.

Papp, J. 1984. Palaearctic species of *Microgaster* Latreille (=*Microplitis* Förster) with description of seven new species (Hymenoptera, Braconidae, Microgastrinae). *Entomologische Abhandlungen* **47**: 95-140.

Papp, J. 1985. Contributions to the Braconid fauna of Hungary, VII. Rogadinae (Hymenoptera: Braconidae). *Folia Entomologica Hungarica* **46**: 143-164.

Papp, J. 1987. A survey of the European species of *Apanteles* Förster (Hymenoptera, Braconidae: Microgastrinae), X. The *glomeratus*-group 2 and the *cultellatus*-group. *Annales Historico-Naturales Musei Nationalis Hungarici* **79**: 207-258.

Papp, J. 1988. A survey of the European species of *Apanteles* Först. (Hymenoptera, Braconidae: Microgastrinae) XI. “Homologization” of the species-groups of *Apanteles s. l.* with Mason’s generic taxa. Checklist of genera. Parasitoid / host list 1. *Annales Historico-naturales Musei Nationalis Hungarici* **80**: 145-175.

Papp, J. 1992. *Bracon strobilorum* Ratzeburg and its related species (Hymenoptera: Braconidae, Braconinae). *Folia Entomologica Hungarica* **53**: 129-134.

Papp, J. 1993. New Braconid wasps (Hymenoptera, Braconidae) in the Hungarian Natural History Museum, 4. *Annales Historico-Naturales Musei Nationalis Hungarici* **84**: 155-180.

Papp, J. 1995*a*. Revision of C. Wesmael’s *Chelonus* species (Hymenoptera Braconidae Cheloninae). *Bulletin de l’Institut Royal des Sciences Naturelles de Belgique, Entomologie* **65**: 115-134.

Papp, J. 1995*b*. Braconidae (Hymenoptera) from Korea, XVII. *Annales Historico-naturales Musei Nationalis Hungarici* **87**: 123-127.

Papp, J. 1996*a*. Taxonomic revision of C. Rondani's braconid species (Hymenoptera Braconidae). *Redia* **78** (1995): 303-309.

Papp, J. 1996*b*. Contribution to the braconid fauna of Hungary, XI. Cheloninae and Sigalphinae (Hymenoptera: Braconidae). *Folia Entomologica Hungarica* **57**: 131-156.

Papp, J. 1996*c*. The braconid wasps of (Hymenoptera, Braconidae) of the Bükk National Park (NE Hungary). *The Fauna of the Bükk National Park* **2**: 453-476.

Papp, J. 1997. Taxonomic revision of seven European species of the genus *Bracon* Fabricius (Hymenoptera: Braconidae). *Folia Entomologica Hungarica* **58**: 115-135.

Papp, J. 1998. Contributions to the braconid fauna of Cyprus (Hymenoptera, Braconidae: Braconinae). *Entomofauna* **19**: 241-251.

Papp, J. 1999*a*. Two new species of *Bracon* from Britain (Hym., Braconidae, Braconinae). *Entomologist’s Monthly Magazine* **135**: 145-152.

Papp, J. 1999*b*. A revision of the *Bracon* species described by O. Schmiedeknecht (Insecta: Hymenoptera: Braconidae: Braconinae). *Entomologische Abhandlungen* **58**: 289-308.

Papp, J. 1999*c*. The braconid wasps (Hymenoptera: Braconidae) of the Aggtelek National Park (N.E. Hungary). Pp. 547-572 in: Mahunka, S., ed. *The Fauna of the Aggtelek National Park. Vol. II*. Hungarian Natural History Museum, Budapest.

Papp, J. 2000. First synopsis of the species of *obscurator* species-group, genus *Bracon*, subgenus *Glabrobracon* (Hymenoptera: Braconidae, Braconinae). *Annales Historico-naturales Musei Nationalis Hungarici* **92**: 229-264.

Papp, J. 2002. The Braconid wasps (Hymenoptera: Braconidae) of the Ferto-Hansag National Park (NW Hungary). Pp. 557-581 in: *The Fauna of the Fertő-Hansag National Park*.

Papp, J. 2003. Type specimens of the braconid species described by T. A. Marshall and deposited in the Hungarian Natural History Museum (Hymenoptera: Braconidae). *Annales Historico-Naturales Musei Nationalis Hungarici* **95**: 135-146.

Papp, J. 2004*a*. Type specimens of the braconid species by Gy. Szépligeti deposited in the Hungarian Natural History Museum (Hymenoptera: Braconidae). *Annales Historico-Naturales Musei Nationalis Hungarici* **96**: 153-223.

Papp, J. 2004*b*. A revision of Szépligeti's *Microchelonus* species described from Hungary (Hymenoptera: Braconidae: Cheloninae). *Annales Historico-Naturales Musei Nationalis Hungarici* **96**: 225-259.

Papp, J. 2004*c*. A Bakony-hegység gyilkosfürkész faunájának alapvetése (Hymenoptera, Braconidae) V. Agathidinae, Alysiinae [A monograph of the braconid fauna of the Bakony Mountains (Hymenoptera, Braconidae) V. Agathidinae, Alysiinae]. *Folia Musei Historico-Naturalis Bakonyiensis* **21**: 111-154.

Papp, J. 2005*a*. A revision of the *Bracon* (*Lucobracon*) species described by Szépligeti from the Western Palaearctic Region (Hymenoptera: Braconidae: Braconinae). *Annales Historico-Naturales Musei Nationalis Hungarici* **97**: 197-224.

Papp, J. 2005*b*. A checklist of the Braconidae of Hungary (Hymenoptera). *Folia Entomologica Hungarica* **66**:137-194.

Papp, J. 2007. Braconidae (Hymenoptera) from Korea XXII. Subfamily Alysiinae. *Acta Zoologica Academiae Scientiarum Hungaricae* **53**: 1-38.

Papp, J. 2008*a*. Redescriptions of *Habrobracon concolorans* (Marshall) and *Habrobracon crassicornis* (Thomson) (Hymenoptera: Braconidae: Braconinae). *Entomologisk Tidskrift* **129**: 165-172.

Papp, J. 2008*b*. A revision of the *Bracon* (subgenera *Bracon* s.str., *Cyanopterobracon*, *Glabrobracon*, *Lucobracon*, *Osculobracon* subgen. n., *Pigeria*) species described by Szépligeti from the western Palaearctic Region (Hymenoptera: Braconidae, Braconinae). *Linzer Biologische Beiträge* **40**: 1741-1837.

Papp, J. 2009*a*. Contribution to the braconid fauna of the former Yugoslavia, V. Ten subfamilies. *Entomofauna* **30**: 1-36.

Papp, J. 2009*b*. Braconidae (Hymenoptera) from Mongolia, XVII. Eleven subfamilies. *Acta Zoologica Academiae Scientiarum Hungaricae* **55**: 139-173.

Papp, J. 2012. A revision of the *Bracon* Fabricius species in Wesmael’s collection deposited in Brussels (Hymenoptera: Braconidae: Braconinae). *European Journal of Taxonomy* **21**: 1-154. doi: http://dx.doi.org/10.5852/ejt.2012.21

Pennacchio, F. 1989. The Italian species of the genus *Aphidius* Nees (Hymenoptera, Braconidae, Aphidiinae). *Bollettino del Laboratorio di Entomologia Agraria Filippo Silvestri* **46**: 75-106.

Perepechayenko, V. L. 2000. [Review of genera of the tribe Dacnusini (Hymenoptera: Braconidae: Alysiinae) of Palaearctic region.] *Izvestiya Kharkovskogo Entomologitchestogo Obshchestva* **8**: 57-79. [in Russian]

Perkins, J. F. & Nixon, G. E. J. 1939. Insecta, Hymenoptera, Ichneumonoidea. Pp. 139-144 in: Salzman, F., ed. *The Victoria history of the counties of England: A history of the county of Oxford. Vol. 1.* Oxford University Press, London.

Powell, W. 1982. The identification of hymenopterous parasitoids attacking cereal aphids in Britain. *Systematic Entomology* **7**: 465-473.

Pungerl, N. B. 1986. Morphometric and electrophoretic study of *Aphidius* species (Hymenoptera: Aphidiidae) reared from a variety of aphid hosts. *Systematic Entomology* **11**: 327-354.

Quicke, D. L. J. & Sharkey, M. J. 1989. A key to and notes on the genera of Braconinae (Hymenoptera: Braconidae) from America north of Mexico with descriptions of two new genera and three new species. *Canadian Entomologist* **121**: 337-361.

Rakhshani, E., Tomanovic, Z., Stary, P., Kavallieratos, N. G., Ilic, M., Stankovic, S. S. & Rajabi-Mazhar, N. 2011. Aphidiinae parasitoids (Hymenoptera: Braconidae) of *Macrosiphoniella* aphids (Hemiptera: Aphididae) in the western Palaearctic region. *Journal of Natural History* **45**: 2559-2575.

Rakhshani, E., Starý, P., Hidalgo, N. P., Čkrić, J., Moghaddam, M. G., Tomanović, S., Petrović, A. & Tomanović, Z. 2015. Revision of the world *Monoctonia* Starý, parasitoids of gall aphids: taxonomy, distribution, host range, and phylogeny (Hymenoptera, Braconidae: Aphidiinae). *Zootaxa* **3905**: 474-488.

Revels, R. 2006. More on the rise and fall of the Holly Blue. *British Wildlife* **17**: 419-424.

Richards, O. W. 1960. On some British species of *Perilitus* Nees (Hymenoptera: Braconidae). *Proceedings of the Royal Entomological Society of London (B)* **29**: 140-144.

Richards, O. W. 1967 Some British species of *Leiophron* Nees (Hymenoptera: Braconidae, Euphorinae), with the description of two new species. *Transactions of the Royal Entomological Society of London* **119**: 171-186.

Roman, A. 1912. Die Ichneumonidentypen C.P. Thunbergs. *Zoologiska Bidrag fran Uppsala* **1**.

Schwarz, M. & Shaw, M. R. 2000. Western Palaearctic Cryptinae (Hymenoptera: Ichneumonidae) in the National Museums of Scotland, with nomenclatural changes, taxonomic notes, rearing records and special reference to the British check list. Part 3. Tribe Phygadeuontini, subtribes Chiroticina, Acrolytina, Hemitelina and Gelina (excluding *Gelis*), with descriptions of new species. *Entomologist's Gazette* **51**: 147-186.

Sharanowski, B. J., Dowling, A. P. G. & Sharkey, M. J. 2011. Molecular phylogenetics of Braconidae (Hymenoptera: Ichneumonoidea), based on multiple nuclear genes, and implications for classification. *Systematic Entomology* **36**: 549-572.

Sharkey, M. J. & Stoelb, S. A. C. 2012. Revision of *Therophilus* s.s. (Hymenoptera, Braconidae, Agathidinae) from Thailand. *Journal of Hymenoptera Research* **27**: 1-36.

Sharkey, M. J., Laurenne, N. M., Sharanowski, B., Quicke, D. L. J. & Murray, D. 2006. Revision of the Agathidinae (Hymenoptera: Braconidae) with comparisons of static and dynamic alignments. *Cladistics* **22**: 546-567.

Sharkey, M. J., Yu, D. S., van Noort, S., Seltmann, K. & Penev, L. 2009. Revision of the Oriental genera of Agathidinae (Hymenoptera, Braconidae) with an emphasis on Thailand including interactive keys to genera published in three different formats. *ZooKeys* **21**: 19-54.

Shaw, M. R. 1977. *Aleiodes pallidator* (Thunberg) (=*unicolor* Wesmael) (Hym., Braconidae) new to Britain. *Entomologist's Monthly Magazine* **113**: 81.

Shaw, M. R. 1979*a*. Book review. Kloet and Hincks. A Check List of British Insects, Second Edition (completely revised). Part 4: Hymenoptera. *Entomologist's Gazette* **30**: 218-221.

Shaw, M. R. 1979*b*. *Rogas pulchripes* (Wesmael) (Hymenoptera: Braconidae) and other parasites of arboreal *Acronicta* species (Lepidoptera: Noctuidae) at Chat Moss, Manchester. *Entomologist's Gazette* **30**: 291-294.

Shaw, M. R. 1981. Undue alarm over parasitism (Hym.) of *Clostera anachoreta* (D. & S.). *Entomologist's Record and Journal of Variation* **93**: 115.

Shaw, M. R. 1983. *Aphaereta pallipes* (Say) (Hym: Braconidae) new to Britain and Palaearctic region, with remarks on other parasites of Diptera. *Entomologist's Monthly Magazine* **119**: 73-74.

Shaw, M. R. 1988*a*. *Meteorus brevicauda* Thomson (Hymenoptera: Braconidae) reared from larvae of *Zeugophora subspinosa* (Fabricius) (Coleoptera: Chrysomelidae). *Entomologist’s Gazette* **39**: 205-206.

Shaw, M. R. 1988*b*. *Spathius curvicaudis* Ratzeburg (Hym.: Braconidae) new to Britain and parasitising *Agrilus pannonicus* (Piller and Mitterpacher) (Col.: Buprestidae). *Entomologist's Record* **100**: 215-216.

Shaw, M. R. 1992*a*. A new species of *Hygroplitis* Thomson in England (Hymenoptera: Braconidae, Microgastrinae). *Entomologist's Gazette* **43**: 283-288.

Shaw, M. R. 1992*b*. *Microtypus wesmaelii* Ratzeburg (Hymenoptera: Braconidae, Microtypinae), a species and subfamily new to Britain. *Entomologist's Gazette* **43**: 289-291.

Shaw, M. R. 1993. An enigmatic rearing of *Dolopsidea indagator* (Haliday) (Hymenoptera: Braconidae). *The Entomologist's Record and Journal of Variation* **105**: 31-36.

Shaw, M. R. 1994. Some recent British specimens of *Baryproctus barypus* (Marshall) (Hym., Braconidae, Braconinae). *Entomologist's Monthly Magazine* **130**: 219-221.

Shaw, M. R. 1995. Observations on the adult behaviour and biology of *Histeromerus mystacinus* Wesmael (Hymenoptera: Braconidae). *The Entomologist* **114**: 1-13.

Shaw, M. R. 1996*a*. *Chrysopophthorus hungaricus* (Zilahi-Kiss) (Hymenoptera: Braconidae, Euphorinae) new to Britain, a parasitoid of adult Chrysopidae (Neuroptera). *Entomologist's Gazette* **47**: 185-187.

Shaw, M. R. 1996*b*. British records of two species of *Blacometeorus* Tobias (Hymenoptera: Braconidae, Blacinae). *Entomologist's Gazette* **47**: 267-268.

Shaw, M. R. 1997. The genus *Heterospilus* Haliday in Britain, with descriptions of a new species and remarks on related taxa (Hymenoptera: Braconidae: Doryctinae). *Zoologische Mededelingen* **71**: 33-41.

Shaw, M. R. 1998. Some genera and species of Doryctinae (Hymenoptera: Braconidae) new to Britain. *Entomologist's Gazette* **49**: 191-194.

Shaw, M. R. 1999. Rearing records of two species of *Cenocoelius* Haliday from Britain (Hymenoptera: Braconidae, Cenocoeliinae). *Entomologist's Gazette* **50**: 283-286.

Shaw, M. R. 2000*a*. Records of *Aleiodes excavatus* (Telenga) (Hym., Braconidae) from the British Isles. *Entomologist's Monthly Magazine* **136**: 251.

Shaw, M. R. 2000*b*. Two species of *Coeloides* (Hym., Braconidae, Braconinae) new to Britain, with notes on congeners. *Entomologist's Monthly Magazine* **136**: 137-140.

Shaw, M. R. 2003*a*. Revised synonymy in the genus *Cotesia* (Hymenoptera: Braconidae: Microgastrinae): the identity of *Microgaster vestalis* Haliday, 1834, as a senior synonym of *Apanteles plutellae* Kurdjumov, 1912. *Entomologist's Gazette* **54**: 187-189.

Shaw, M. R. 2003*b*. Adverse comment on the supposed British status of the web-spinning sawfly *Cephalcia arvensis* Panzer (Hymenoptera: Pamphiliidae). *British Journal of Entomology and Natural History* **16**: 2.

Shaw, M. R. 2004. *Microgaster alebion* Nixon and its 'var A': description of a new species and biological notes (Hymenoptera: Braconidae, Microgastrinae). *Entomologist's Gazette* **55**: 217-224.

Shaw, M. R. 2005. Rediscovery of *Earinus transversus* Lyle (Hym.: Braconidae: Agathidinae), a parasitoid of *Trichopteryx polycommata* (D.&S.) (Lep.: Geometridae: Larentiinae). *Entomologist's Record* **117**: 85-87.

Shaw, M. R. 2007. The species of *Cotesia* Cameron (Hymenoptera: Braconidae: Microgastrinae) parasitizing Lycaenidae (Lepidoptera) in Britain. *British Journal of Entomology and Natural History* **20**: 255-267.

Shaw, M. R. 2009. Occurrence of the genus *Elasmosoma* Ruthe (Hymenoptera: Braconidae, Euphorinae) in Britain. *British Journal of Entomology and Natural History* **22**: 49-51.

Shaw, M. R. 2010. Palaearctic Homolobinae (Hymenoptera: Braconidae) in the National Museums of Scotland, with host and distribution records and a key to British species. *Entomologist’s Gazetter* **61**: 43-51.

Shaw, M. R. 2012. Notes on some European Microgastrinae (Hymenoptera: Braconidae) in the National Museums of Scotland, with twenty species new to Britain, new host data, taxonomic changes and remarks, and descriptions of two new species of *Microgaster* Latreille. *Entomologist's Gazette* **63**: 173-201.

Shaw, M. R. & Askew, R. R. 1976. Ichneumonoidea (Hymenoptera) parasitic upon leaf-mining insects of the orders Lepidoptera, Hymenoptera and Coleoptera. *Ecological Entomology* **1**: 127-133.

Shaw, M. R. & Bailey, M. 1991. Parasitoids (Hymenoptera: Braconidae, Ichneumonidae, Pteromalidae) and notes on the biology of the fern-boring sawfly *Heptamelus ochroleucus* (Stephens) (Hymenoptera: Tenthredinidae) in the English Lake District. *The Entomologist* **110**: 103-109.

Shaw, M. R. & Huddleston, T. 1991. Classification and biology of braconid wasps (Hymenoptera: Braconidae). *Handbooks for the Identification of British Insects* **7**: 1-126.

Shaw, M. R. & Jennings, M. T. 2008. *Polemochartus melas* (Giraud) (Hymenoptera: Braconidae, Alysiinae) new to Britain. *Entomologist's Gazette* **59**: 114-116.

Shaw, M. R. & Quicke, D. L. J. 1999. The British genera of Braconinae (Hym., Braconidae). *Entomologist's Monthly Magazine* **135**: 95-101.

Shaw, M. R. & Sims, I. 2015. Notes on the biology, morphology, nomenclature and classification of *Pseudavga* *flavicoxa* Tobias, 1964 (Hymenoptera, Braconidae, Rhysipolinae), a genus and species new to Britain parasitizing *Bucculatrix thoracella* (Thunberg) (Lepidoptera, Bucculatricidae). *Journal of Hymenoptera Research* **42**: 21-32.

Shaw, M. R. & Skelton, M. J. 2008. Parasitism (Hymenoptera: Braconidae, Microgastrinae) in an apparently adventitious colony of *Lymantria dispar* (Linnaeus) (Lepidoptera: Lymantriidae) in southern England, with speculations on the biology of *Glyptapanteles porthetriae* (Muesebeck). *Entomologist's Gazette* **59**: 109-113.

Shenefelt, R. D. 1973. Braconidae 5. Microgasterinae & Ichneutinae. *Hymenopterorum Catalogus (nova editio)* **11**: 669-812.

Shenefelt, R. D. 1974. Braconidae 7. Alysiinae. *Hymenopterorum Catalogus (nova editio)* **11**: 937-1113.

Simbolotti, G. & van Achterberg, C. 1992. Revision of the West Palaearctic species of the genus *Bassus* Fabricius (Hymenoptera: Braconidae). *Zoologische Verhandeligen* **281**: 1-80.

Simbolotti, G. & van Achterberg, C. 1999. Revision of the West Palaearctic species of the genus *Agathis* Latreille (Hymenoptera: Braconidae: Agathidinae). *Zoologische Verhandeligen* **325**: 1-167.

Smith, F. 1853. *List of the specimens of British animals in the collection of the British Museum. Part XIII. Nomenclature of Hymenoptera*. Taylor & Francis, London.

Starý, P. 1959. A revision of the genus *Dyscritulus* Hincks. (Hymenoptera, Braconidae, Aphidiinae). *Acta Faunistica Entomologica Musei Nationalis Pragae* **5**: 69-74.

Starý, P. 1973. A review of the *Aphidius* species (Hymenoptera, Aphidiidae) of Europe. *Annotationes Zoologicae et Botanicae*, Bratislava. **84**: 1-85.

Starý, P. 1975. *Aphidius colemani* Viereck: its taxonomy, distribution and host range. *Acta Entomologica Bohemoslovaca* **72**: 156-163.

Starý, P. 1978. Parasitoid spectrum of the arboricolous callaphid aphids in Europe (Hymenoptera, Aphidiidae; Homoptera, Aphidoidea, Callaphidae). *Acta Entomologica Bohemoslovaca* **75**: 164-177

Stelfox, A. W. & Graham, M. W. R. de V. 1951*a*. Notes on the genus *Aspilota* (Hym., Braconidae, Alysiinae), with descriptions of five new species. *The Entomologist's Monthly Magazine* **87**: 3-7.

Stelfox, A. W. & Graham, M. W. R. de V. 1951*b*. Descriptions of two new species of *Aspilota* (Hym., Braconidae) from England. *Entomologist's Monthly Magazine* **87**: 222-223.

Stevens, N. B., Austin, A. D. & Jennings, J. T. 2010. Synopsis of Australian agathidine wasps (Hymenoptera: Braconidae: Agathidinae). *Zootaxa* **2480**: 1-26.

Stevens, N. B., Austin, A. D. & Jennings, J. T. 2011. Diversity, distribution and taxonomy of the Australian agathidine genera *Camptothlipsis* Enderlein, *Lytopylus* Foerster and *Therophilus* Wesmael (Hymenoptera: Braconidae: Agathidinae). *Zootaxa* **2887**: 1-49.

Stigenberg, J. & Shaw, M. R. 2013. Western Palaearctic Meteorinae (Hymenoptera: Braconidae) in the National Museums of Scotland, with rearing, phenological and distributional data including six species new to Britain, and a discussion of a potential route to speciation. *Entomologist’s Gazette* **64**: 251-268.

Szépligeti, G. 1904. Hymenoptera. Fam. Braconidae. *Genera Insectorum* **22**: 1-253.

Szépligeti, G. 1906. Braconiden aus der Sammlung des ungarischen National-Museums, 1. *Annales Historico-Naturales Musei Nationalis Hungarici* **4**: 547-618.

Taeger, A. 1989. *Die* Orgilus*-Arten der Paläarktis (Hymenoptera, Braconidae).* Akademie der Landwirtschaftswissenschaften der Deutschen Demokratischen Republik, Berlin.

Tobias, V. I., ed. 1986. [*Keys to the Insects of the European Part of the USSR. Volume III Hymenoptera. Part IV.*] Nauka Publisher, Leningrad. [English translation published in in 1995, Science Publishers, Inc., New Hampshire, USA]

Tobias, V. I. 1998. Alysiinae (Dacnusini). Pp. 299-411 in: Ler, P. A., ed. [*Key to the insects of Russian Far East. Vol. 4. Neuropteroidea, Mecoptera, Hymenoptera. Pt 3*.] Dal'nauka, Vladivostok. 706 pp. [in Russian].

Tobias, V. I. & Jakimavicius, A. B. 1973. [Supplementary data about the braconid (Hymenoptera, Braconidae) fauna of Lithuania.] (in Russian with English summary). *Acta Entomologica Lituanica* **2**: 23-28.

Tobias, V. I. & Lozan, A. 2003. Central-European species of *Microchelonus* Szépligeti (Hymenoptera, Braconidae) with very big apical metasomal aperture of males. *Linzer Biologische Beiträge* **35**: 239-261.

Tobias, V. I. & Shaw, M. R. 2005. Rearing records of two species of *Microchelonus* Szépligeti from Britain, with the description of a new species (Hym., Braconidae: Cheloninae). *Entomologist's Monthly Magazine* **141**: 15-19.

Tomanović, Z., Kavallieratos, N. G., Starý, P., Stanisavljević, L., Petrović-Obradović, S., Tomanović, M. & Milutinović, M. 2006. Phylogenetic relationships among Praini (Hymenoptera: Braconidae: Aphidiinae) aphid parasitoids, with redescription of two species. *Insect Systematics and Evolution* **37**: 213-226.

Traugott, M., Bell, J. R., Broad, G. R., Powell, W., Van Veen, F. J. F., Vollhardt, I. M. G. & Symondson, W. O. C. 2008. Endoparasitism in cereal aphids: molecular analysis of a whole parasitoid community. *Molecular Ecology* **17**: 3928-3938.

Tremblay, E. & Eady, R. D. 1978. *Lysiphlebus confusus* n.sp. per *Lysiphlebus ambiguus* sensu Auct. nec Haliday (1834) (Hymenoptera Ichneumonoidea). *Bollettino del Laboratorio di Entomologia Agraria 'Filippo Silvestri'* **35**: 180-184.

Van Veen, F. J. F., Müller, C. B., Pell, J. K. & Godfray, H. C. J. 2008. Food web structure of three guilds of natural enemies: predators, parasitoids and pathogens of aphids. *Journal of Animal Ecology* **77**: 191-200.

Walker, A. K. & Wharton, R. A. 2011. A review of New World *Eurytenes* s. str. (Hymenoptera, Braconidae, Opiinae). *Journal of Hymenoptera Research* **20**: 23-46.

Wharton, R. A. 1980. Review of the Nearctic Alysiini (Hymenoptera, Braconidae). With discussion of generic relationships within the tribe. *University of California Publications in Entomology* **88**: 1-112+xi.

Wharton, R. A. 1983. Variation in *Opius hirtus* Fischer and discussion of *Desmiostoma* Foerster (Hymenoptera: Braconidae). *Proceedings of the Entomological Society of Washington* **85**: 327-330.

Wharton, R. A. 1985. Characterization of the genus *Aspilota* (Hymenoptera: Braconidae). *Systematic Entomology* **10**: 227-237.

Wharton, R. A. 1986. The braconid genus *Alysia* (Hymenoptera): a description of the subgenera and a revision of the subgenus *Alysia*. *Systematic Entomology* **11**: 453-504.

Wharton, R. A. 1988*a*. Classification of the braconid subfamily Opiinae (Hymenoptera). *Canadian Entomologist* **120**: 333-360.

Wharton, R. A. 1988*b*. The braconid genus *Alysia* (Hym.): a revision of the subgenus *Anarcha*. *Contributions of the American Entomological Institute* **25**: 1-69.

Wharton, R. A. 1993. Review of the Hormiini (Hymenoptera: Braconidae) with a description of new taxa. *Journal of Natural History* **27**: 107-171.

Wharton, R. A. 1994. New genera, species, and records of New World Alysiinae (Hymenoptera: Braconidae). *Proceedings of the Entomological Society of Washington* **96**: 630-664.

Wharton, R. A. 1997. Alysiinae. Pp. 85-118 in: Wharton, R. A., Marsh, P. M. & Sharkey, M. J., eds. *Manual of the New World genera of the family Braconidae (Hymenoptera)*. International Society of Hymenopterists, Special Publication No. 1. 439 pp.

Wharton, R. A. 2002. Revision of the Australian Alysiini (Hymenoptera : Braconidae). *Invertebrate Systematics* **16**: 7-105.

Wharton, R. A. 2006. The species of *Sternaulopius* Fischer (Hymenoptera: Braconidae, Opiinae) and the braconid sternaulus. *Journal of Hymenoptera Research* **15**: 317-347.

Wharton, R. A. & Austin, A. D. 1991. Revision of Australian Dacnusini (Hymenoptera; Braconidae: Alysiinae): Parasitoids of cyclorrhaphous Diptera. *Journal of the Australian Entomological Society* **30**: 193-206.

Wilkinson, D. S. 1945. Description of Palaearctic species of *Apanteles* (Hymen., Braconidae). *Transactions of the Entomological Society of London* **95**: 35-226.

Yang, Z., Gu, Y. & Song, Y. 2003. A new species in the genus *Ropalophorus* Curtis (Hymenoptera: Braconidae) from China, parasitizing adults of the bark beetle *Ips subelongatus* (Coleoptera: Scolytidae), with a key to world species of the genus. *Zoologische Mededelingen* **77**: 631-636.

Yu, D. S., van Achterberg, C. & Horstmann, K. 2005. *World Ichneumonoidea 2004. Taxonomy, biology, morphology and distribution*. CD/DVD. Taxapad, Vancouver, Canada.

Yu, D. S., van Achterberg, C. & Horstmann, K. 2012. *Ichneumonoidea 2011. Taxonomy, biology, morphology and distribution*. Database on flash-drive. www.taxapad.com, Ottawa, Ontario, Canada.

Zaldivar-Riverón, A, Areekul, B., Shaw, M. R. & Quicke, D. L. J. 2004. Comparative morphology of the venom apparatus in the braconid wasp subfamily Rogadinae (Insecta, Hymenoptera, Braconidae) and related taxa. *Zoologica Scripta* **33**: 223-237.

Zaldivar-Riverón, A., Mori, M. & Quicke, D. L. J. 2006. Systematics of the cyclostome subfamilies of braconid parasitic wasps (Hymenoptera: Ichneumonoidea): A simultaneous molecular and morphological Bayesian approach. *Molecular Phylogenetics and Evolution* **38**: 130-145.

Zaldivar-Riverón, A., Shaw, M. R., Sáez, A. G., Mori, M., Belokobylskij, S. A., Shaw, S. R. & Quicke, D. L. J. 2008*a*. Evolution of the parasitic wasp subfamily Rogadinae (Braconidae): phylogeny and evolution of lepidopteran host ranges and mummy characteristics. *BMC Evolutionary Biology* **8**: 329 (20pp.)

Zaldívar-Riverón, A., Belokobylskij, S. A., León-Regagnon, V., Briceño-G., R. & Quicke, D. L. J. 2008*b*. Molecular phylogeny and historical biogeography of the cosmopolitan parasitic wasp subfamily Doryctinae (Hymenoptera:Braconidae). *Invertebrate Systematics* **22**: 345-363.

1. Except for *Agathis* and ‘*Bassus*’ species (i.e. including *Lytopylus* and *Therophilus*), distribution and synonymic data from Nixon (1986). [↑](#footnote-ref-2)
2. The phylogenetic results of Sharkey *et al*. (2006) suggest that Earinini be accorded tribal status but that the classification of the Agathidini *s.l*. needs more scrutiny. [↑](#footnote-ref-3)
3. Distribution and synonymic data from Nixon (1986) and Simbolotti & van Achterberg (1999). [↑](#footnote-ref-4)
4. Simbolotti & van Achterberg (1999) synonymised *Agathis achterbergi* Nixon, 1986 under *breviseta* but it is listed as a valid species in Taxapad. [↑](#footnote-ref-5)
5. Listed by Huddleston (1978); probably refers to *varipes*. [↑](#footnote-ref-6)
6. Following molecular phylogenetic analysis of Agathidinae (Sharkey *et al*., 2006), the genus *Bassus s.l.* was recognised as being a polyphyletic assemblage. Species are now being described in or reassigned to *Lytopylus*, *Thermophilus* and the non-British *Camptothlipsis* (Sharkey *et al.*, 2009; Stevens *et al*., 2010, 2011; van Achterberg & Long, 2010; van Achterberg, 2011) but very few European species have been formally transferred. The species on the British list have therefore been reassigned here on the bases of recent generic keys (e.g. Sharkey *et al*., 2009) in anticipation of future taxonomy. Whereas *Bassus s.s*. is now restricted to a small group of species (only one in Britain), *Therophilus* remains large and probably para- or polyphyletic. Distribution and synonymic data for *Bassus*, *Lytopylus* and *Therophilus* from Nixon (1986) and Simbolotti & van Achterberg (1992). [↑](#footnote-ref-7)
7. See comments under *Bassus*; generic synonymy from Sharkey *et al*. (2009). [↑](#footnote-ref-8)
8. See comments under *Bassus*; generic synonymy from Sharkey *et al*. (2009). [↑](#footnote-ref-9)
9. Taken out of synonymy with *conspicuus* by Simbolotti & van Achterberg (1992). [↑](#footnote-ref-10)
10. Sharkey & Stoelb (2012) defined *Therophilus* as a monophyletic genus and listed the included species, but several species previously classified in *Therophilus* are now of uncertain generic placement. [↑](#footnote-ref-11)
11. Listed by Huddleston (1978) but no mention of British specimens by Nixon (1986), who states, however, that the traditional interpretation of the species is probably correct. No British or Irish specimens were seen by Simbolotti & van Achterberg (1992) but van Achterberg has identified an English specimen in BMNH as *Therophilus dimidiator*. [↑](#footnote-ref-12)
12. Transferred from *Agathis* by Simbolotti & van Achterberg (1999). [↑](#footnote-ref-13)
13. Listed by Huddleston (1978) but no mention of British specimens by Nixon (1986) or Simbolotti & van Achterberg (1992). Transferred from *Agathis* by Simbolotti & van Achterberg (1999) but listed as a species of *Agathis* in Taxapad. [↑](#footnote-ref-14)
14. Listed as a ‘species inquirendae’ by Nixon (1986) but included as a German species by Simbolotti & van Achterberg (1992). Transferred to *Bassus* by Simbolotti & van Achterberg (1999) but not listed as a species of *Therophilus* by Sharkey & Stoelb (2012). [↑](#footnote-ref-15)
15. Listed by Huddleston (1978) but no mention of British specimens by Nixon (1986) or Simbolotti & van Achterberg (1992). [↑](#footnote-ref-16)
16. English record from specimen in BMNH. [↑](#footnote-ref-17)
17. Much of the taxonomy and distribution from Wharton (1986). [↑](#footnote-ref-18)
18. Much of the taxonomy and distribution from Wharton (1988*b*). [↑](#footnote-ref-19)
19. Synonymised under *Aphaereta* by van Achterberg (1995) but treated as a separate genus again in Taxapad. [↑](#footnote-ref-20)
20. Some distribution data from Stelfox & Graham (1951*a*). Some species treated as belonging to *Dinotrema* by other authors have been included in *Aspilota* in Taxapad and there is clearly much work to be done in allocating species to the current generic concepts (see Wharton, 1985). The late T. Munk was preparing a revision of the European species of *Aspilota s.l.* (see note under *Dinotrema*). [↑](#footnote-ref-21)
21. Identified by T. Munk as a species of *Dinotrema*. [↑](#footnote-ref-22)
22. *Dapsilarthra* has been used in a very broad sense to include *Adelurola, Mesocrina, Heterolexis* and *Grammospila,* and in a more narrow sense to include the last two genera. Van Achterberg (2014) is followed here in according each generic rank. [↑](#footnote-ref-23)
23. Some distribution data from Stelfox & Graham (1951*a*,*b*) and van Achterberg (1988*b*). The late T. Munk was revising the European species of *Aspilota s.l.* and gave much helpful advice on the generic placements of the species occurring in Britain and Ireland, some of which is unpublished and will therefore differ from the generic combinations found in, e.g. Belokobylsij *et al.* (2003) and Taxapad (Yu *et al*., 2012). Munk also advised that although *Aspilota* is a well-defined genus, *Dinotrema* is not defined by any apomorphies and will be split up. [↑](#footnote-ref-24)
24. Wharton (1980) summarises the arguments against recognition of the genus *Synaldis*; van Achterberg (1988) likewise notes that the genus is defined only by the absence of fore wing vein 2-*SR*, which is known to be intraspecifically variable (Wharton, 1980). Despite this, various authors attach great taxonomic weight to this venational character and maintain *Synaldis* as a valid genus (as reflected by the classification in Yu *et al.*, 2012). Species that would be classified in *Synaldis* (*acutidentata*, *concolor*, *distracta* and *globipes*) are listed here in *Aspilota* and *Dinotrema*, according to Fauna Europaea. [↑](#footnote-ref-25)
25. *Synaldis maximum* Fischer, 1962 has been treated as a junior synonym by König (1972) and subsequent authors but Munk (pers. comm.) regarded this as a valid species of *Dinotrema* that may be found to occur in Britain. [↑](#footnote-ref-26)
26. Regarded by most authors as a synonym of *nervosum*, but Munk (pers. comm.) treats this as a valid species. [↑](#footnote-ref-27)
27. Treated as a species of *Eudinostigma* in Fauna Europaea and Taxapad (Yu *et al*., 2012). [↑](#footnote-ref-28)
28. Transferred from *Aspilota* by Disney & Munk (2005). [↑](#footnote-ref-29)
29. Treated as a species of *Eudinostigma* in Fauna Europaea and Taxapad (Yu *et al*., 2012). [↑](#footnote-ref-30)
30. Distribution data from van Achterberg (1988*b*). [↑](#footnote-ref-31)
31. T. Munk (pers. comm.) regarded this as an unidentified species; the type has been destroyed. [↑](#footnote-ref-32)
32. Described as a separate genus by van Achterberg (1988*b*), who gives distribution data for the one included species. [↑](#footnote-ref-33)
33. *Grammospila fuscula* (Griffiths, 1968, *Dapsilarthra*) removed from synonymy by van Achterberg (2014). [↑](#footnote-ref-34)
34. Distribution data from van Achterberg (1988*b*). [↑](#footnote-ref-35)
35. Some taxonomic and distribution data from van Achterberg (1997). [↑](#footnote-ref-36)
36. Listed as British by Lyle (1933) but we have not seen any British or Irish specimens. [↑](#footnote-ref-37)
37. Not listed in Huddleston (1978), although described from Scottish material (van Achterberg, 1998). [↑](#footnote-ref-38)
38. Listed as a species of *Idiolexis* in Fauna Europaea. [↑](#footnote-ref-39)
39. Distribution data from van Achterberg & O’Connor (1990) and the collections of NMS. [↑](#footnote-ref-40)
40. The classification of the Dacnusini was revised by Griffiths (1964-68) in a pioneering treatment that was one of the first applications of explicit phylogenetic methods in taxonomy. It was also unusual in being based on extensive reared material. Griffiths’ classification is followed here although one of the consequences of his strict adherence to phylogenetic principles is that *Chorebus* and *Dacnusa* are very large genera. Also, as Griffiths realised, the definition of *Exotela* is unsatisfactory as it lacks clear apomorphic characters. This group of insects had previously been revised by Nixon (1943-54), who recognised a greater number of genera, and some authors (Tobias, 1986, 1998; Perepechayenko, 2000; Fischer *et al.*, 2004) have resurrected Nixon’s genera or used them as subgenera. This seems a retrograde step given Griffiths’ clear statements of phylogenetic hypotheses and is not used here. In both *Chorebus* and *Dacnusa*, Griffiths defined a series of species groups that with further research should form the basis of better classifications of these genera. Griffiths (1964) gives a key to genera and Wharton (1997) is also very helpful. The *Coelinius* genus group (*Coelinidia, Coelinius*, *Polemochartus, Trachionus, Epimicta, Aristelix, Laotris, Sarops* and *Synelix*) is generally agreed to be monophyletic and was not revised at species level by Nixon or Griffiths. Distribution data from Griffiths’ and Nixon’s revisions (*loc. cit.*) and NMS. [↑](#footnote-ref-41)
41. No recent revision of this genus. [↑](#footnote-ref-42)
42. Transferred from *Chorebus* by Perepechayenko (2000). [↑](#footnote-ref-43)
43. Transferred from *Chorebus* by van Achterberg (1997). [↑](#footnote-ref-44)
44. Synonymy first established in Shenefelt (1974) but overlooked until O’Connor *et al.* (1999). [↑](#footnote-ref-45)
45. The status of this taxon needs further research. [↑](#footnote-ref-46)
46. There is no recent treatment of this genus; generic synonymy follows van Achterberg (2014). [↑](#footnote-ref-47)
47. Griffiths (1964) proposed that *Polemochartus* and *Coelinidea* should be included as subgenera of *Coelinius*. Wharton & Austin (1991), Wharton (1994) and Kula (2008) supported the concept of an enlarged *Coelinius* but, pointing to intermediate taxa in the Oriental Region, argued against the retention of subgenera. Van Achterberg (2014) retains the three genera which is current European usage and is followed here pending a modern review of the group. [↑](#footnote-ref-48)
48. Based on its unusual venation, *adducta* is often placed in the monotypic genus *Agonia*; Griffiths’ (1964) argument that it is a derived species of *Dacnusa* is followed here. [↑](#footnote-ref-49)
49. Listed as a species of *Alysia* by Shenefelt (1974) and Huddleston (1978) although a junior homonym of *Alysia minuta* Nees, 1812; Wharton (1986) showed it to belong to *Dacnusa* and van Achterberg (in litt.) concluded that it is conspecific with *confinis.* [↑](#footnote-ref-50)
50. Synonymised by Nixon (1937) although this was overlooked by Huddleston (1978). [↑](#footnote-ref-51)
51. Griffiths’ (1964-68) concept of *Exotela* is followed here; the most plesiomorphic species (*flavicoxa, interstitialis, melanocera*  and *vaenia*) are often placed in *Antrusa* (e.g. van Achterberg, 2014). [↑](#footnote-ref-52)
52. Griffiths (1967*b*) divided *cyclogaster* into three subspecies, nominate *cyclogaster*, *umbellina* and *sonchina*. Tobias (1986) later treated these as separate species although Godfray (1984) argued that *umbellina* and *cyclogaster* are host race variants. Pending further research they are treated here as a single taxon. [↑](#footnote-ref-53)
53. Placed in *Chorebus* by Tobias & Jakimavicius (1973) and in *Antrusa* by Papp (2007); needs further research. [↑](#footnote-ref-54)
54. In the older literature (and some very recent literature) often treated as a separate family (i.e. Aphidiidae) within Ichneumonoidea. Some distribution data from Mackauer (1961), Starý (1978) and Baker (2013). [↑](#footnote-ref-55)
55. The type is lost (van Achterberg, 1997). [↑](#footnote-ref-56)
56. Treated as a valid species of *Aphidius* by Rakhshani *et al.* (2011) rather than a synonym of *asteris*. [↑](#footnote-ref-57)
57. Introduced into greenhouses for biocontrol (Starý, 1975). [↑](#footnote-ref-58)
58. Also recorded by Müller et al. (1999). [↑](#footnote-ref-59)
59. Belshaw (appendix in Müller et al., 1999) demonstrates why this species should be treated as separate from urticae. [↑](#footnote-ref-60)
60. Belokobylskij et al. (2003) treat tanacetarius as the valid name for tanaceti, which is a nomen nudum. This species has not been listed as British since its original description (Curtis, 1837) and Smith’s (1853) catalogue. Pungerl (1986) treated tanacetarius as a valid species but did not see any British or Irish material. [↑](#footnote-ref-61)
61. A possible senior synonym of uzbekistanicus (Starý, 1973). [↑](#footnote-ref-62)
62. Included as a British species by Lyle (1933) on the basis of Szépligeti’s (1904) Palaearctic catalogue; the type is lost and the species has not been interpreted by recent authors. [↑](#footnote-ref-63)
63. There have been no references to this species other than Smith’s (1853) and Marshall’s (1872) catalogue listings. Although proposed (Curtis, 1837) as a name for the species called Aphidius constrictus by Haliday, it has not been interpreted by recent authors. [↑](#footnote-ref-64)
64. In Belokobylskij et al. (2003), van Achterberg listed these species under the genus name Misaphidus but, according to van Achterberg (pers. comm.), Misaphidus Rondani, 1848 s.l. (type-species: Misaphidus crudelis Rondani, 1848 (= Aphidius centaureae Haliday, 1833)) is a ‘nomen oblitum’ according to Article 23.9 of ICZN. [↑](#footnote-ref-65)
65. Distribution from van Achterberg (1989), who elevated Harkeria from a subgenus of Monoctonus. [↑](#footnote-ref-66)
66. Included on the basis of specimens in BMNH, identified by R.D. Eady as ambiguus sensu Mackauer and Starý nec Haliday, later described by Tremblay & Eady (1978) as a separate species, confusus, the true ambiguus belonging in Adialytus. [↑](#footnote-ref-67)
67. We do not know where Enobokhare’s material is deposited and the identification needs to be checked. [↑](#footnote-ref-68)
68. Synonymy and distribution data from Gärdenfors (1986). [↑](#footnote-ref-69)
69. Transferred to Areopraon by Tomanović et al. (2006). [↑](#footnote-ref-70)
70. *Dyscritus suffolciensis* Morley, 1933 is listed by default as a species of *Dyscritulus*in Taxapad (Yu *et al*., 2012) as although Starý (1959) recognised it as belonging to Euphorinae, its identity had not yet been established. Here it is synonymised under *Syntretus splendidus*(Marshall) q.v. [↑](#footnote-ref-71)
71. Erroneously listed in Taxapad as a junior synonym of flavinode; see, for example, Kavallieratos *et al*. (2005). [↑](#footnote-ref-72)
72. A Nearctic species, listed by Kloet & Hincks (1945) in error. [↑](#footnote-ref-73)
73. Following the molecular phylogenetic results of Belshaw & Quicke (2002), van Achterberg (2003c) and Belokobylskij *et al*. (2003) we treat the Brachistinae as a subfamily separate from Helconinae. Sharanowski *et al*. (2011) expanded the limits of the Brachistinae by including the tribe Diospilini and the former subfamily Blacinae (as well as the extralimital Brulleiini). The tribes employed here are those that have been used in previous classifications under the former subfamilies, but the tribal classification remains essentially untested. [↑](#footnote-ref-74)
74. Generally considered to be a distinct subfamily, but Sharanowski et al. (2011) found that the blacines nested within the Brachistinae in their molecular phylogenetic analyses. We think it likely that these results will be upheld with further phylogenetic work and follow Sharanowski et al.’s (2011) recommended changes to the subfamily classification. Distribution and taxonomic data from van Achterberg (1988a), with additional references given. Note that Tobias (1986) records *B.* (*Neoblacus*) *koenigi* Fischer, 1967 from England but there is no evidence that this species occurs here. [↑](#footnote-ref-75)
75. The holotype was collected in Sandhurst, Kent (Haeselbarth, 1973) but not listed for Britain or Ireland by van Achterberg (1988a). [↑](#footnote-ref-76)
76. Described as a subspecies of ambulans by Haeselbarth (1973), but not included by Huddleston (1978), where it should have been listed as a synonym. Treated as a separate species by van Achterberg (1997). [↑](#footnote-ref-77)
77. Included, as barynoti (Boudier, 1834) (a junior synonym of Pygostolus sticticus), as a doubtfully placed species by Huddleston (1978). [↑](#footnote-ref-78)
78. Listed as a species of Zele (=Homolobus) in Huddleston (1978). Will be synonymised under another species of Eubazus (van Achterberg, pers. comm.). [↑](#footnote-ref-79)
79. Listed as a species of *Triaspis* by Yu *et al.* (2012) but transferred back to *Schizoprymnus* by Notton *et al.* (2014). [↑](#footnote-ref-80)
80. Seems to have been recorded in error by Kloet & Hincks (1945), perpetuated by Huddleston (1978). [↑](#footnote-ref-81)
81. Although synonymised with *obscurella* by van Achterberg (in Belokobylskij et al., 2003) this is listed as a separate species in Fauna Europaea. There are English specimens identified by van Achterberg as aciculata in NMS and BMNH. [↑](#footnote-ref-82)
82. Listed as a doubtfully British species in Huddleston (1978); the only record is of a tentative identification by Nixon (Hussey, 1952). [↑](#footnote-ref-83)
83. Sharanowski et al. (2011) found that the Diospilini belong in the Brachistinae rather than Helconinae. [↑](#footnote-ref-84)
84. Tentative identification; included here as a certain generic record of an Aspicolpus species in Britain. [↑](#footnote-ref-85)
85. Listed as Taphaeus affinis and T. nigricornis in Huddleston (1978). [↑](#footnote-ref-86)
86. The systematic position of Taphaeus is uncertain. Treated as a genus of Blacinae in Fauna Europaea. [↑](#footnote-ref-87)
87. This is generally considered to be a tribe of Blacinae; we list the Dyscoletini as a tribe of Brachistinae because we follow Sharanowski et al. (2011) in including the Blacinae within Brachistinae. [↑](#footnote-ref-88)
88. Tribal classification follows Belshaw et al. (2001) and Quicke (pers. comm.). For genera other than Bracon and Coeloides, Shaw (1994) and Shaw & Quicke (1999) give distribution data. [↑](#footnote-ref-89)
89. Listed as Glyptomorpha variegata in Huddleston (1978). [↑](#footnote-ref-90)
90. See Shaw (1994) and Jennings (2008) for recent records. [↑](#footnote-ref-91)
91. Comprehensive taxonomic revisions of Bracon are lacking for all but a few species groups. Published works differ substantially in their treatments of valid species, synonyms and subgeneric placements (compare, for example, the differing treatments of Tobias (1986), Papp (1974, 1996c), and Shenefelt (1979)) so the taxonomy in Taxapad (Yu *et al.*, 2012) has, for the most part, been used as a default classification. [↑](#footnote-ref-92)
92. Synonymised by Papp (2012) who treated this taxon as a ‘var’ of *intercessor*. [↑](#footnote-ref-93)
93. Listed as British in Fauna Europaea, probably because it was described by Marshall, but the first published British record was by Jennings (2012). [↑](#footnote-ref-94)
94. Omitted by Huddleston (1978). [↑](#footnote-ref-95)
95. According to Taxapad (Yu *et al.*, 2012), *rugulosus* Szépligeti is preoccupied by *Bracon rugulosus* Nees, 1811 (now classified as a species of *Aleiodes*, in Rogadinae). Omitted by Huddleston (1978). [↑](#footnote-ref-96)
96. Papp (2008b) argues that the name *sulcatulus* should be suppressed in favour of *subrugosus*, although this has not formally been carried out. [↑](#footnote-ref-97)
97. Previously confused under *minutator* (van Achterberg, pers. comm.). Both species are present in the BMNH and NMS collections, det. Papp and van Achterberg. [↑](#footnote-ref-98)
98. Some taxonomic and distribution data from Papp (1999a,b, 2000). [↑](#footnote-ref-99)
99. Omitted by Huddleston (1978), listed by Kloet & Hincks (1945), although it is not clear on what basis. [↑](#footnote-ref-100)
100. Removed from synonymy with terebella by Papp (2008b). [↑](#footnote-ref-101)
101. Faure (1924) mentions English material but *glaphyrus* was omitted by Huddleston (1978). [↑](#footnote-ref-102)
102. Omitted by Huddleston (1978); recorded as British by Fulmek (1968), although we are not sure how reliably; English material in NMS. [↑](#footnote-ref-103)
103. Omitted by Huddleston (1978), listed by Kloet & Hincks (1945), although it is not clear on what basis. [↑](#footnote-ref-104)
104. *Bracon minutator auct*. is *trucidator* Marshall (van Achterberg, pers. comm.). [↑](#footnote-ref-105)
105. Treated as a synonym of *bipartitus* (=*variator*) by Belokobylskij et al. (2003). [↑](#footnote-ref-106)
106. Listed as a subspecies of variator in Taxapad. Listed as a British species by Huddleston (1978) but this actually refers to otiosus. [↑](#footnote-ref-107)
107. Should not have been listed by Huddleston (1978) as it was recorded as British only on the basis of Carr’s Staffordshire lists (see Perkins, 1953; Fitton *et al.*, 1978; Shaw 2003*b*). [↑](#footnote-ref-108)
108. Removed from synonymy with stabilis by Papp (2008a). [↑](#footnote-ref-109)
109. Mainly an indoors species in Britain, attacking pests of stored products. [↑](#footnote-ref-110)
110. Has been variously treated as a valid species or as a synonym of hebetor, being listed under the latter in Taxapad and by Papp (2008a). Both brevicornis and hebetor have been recorded as British. [↑](#footnote-ref-111)
111. Although synonymised with Bracon erraticus by Papp (1999a), this has not been followed by Belokobylskij et al. (2003). [↑](#footnote-ref-112)
112. England and Ireland are listed under the distribution in Shenefelt (1979), and Britain in Fauna Europaea, but we have been unable to trace any British or Irish literature records or specimens. In Belokobylskij et al. (2003) and in Taxapad this is listed as a species of Coeloides but Papp (1992) had established that this species had been mis-interpreted and is in fact a species of *Bracon* (*Lucobracon*). Swiss specimens identified as *strobilorum* by Papp in NMS and BMNH belong in *Bracon*. [↑](#footnote-ref-113)
113. Omitted by Huddleston (1978); listed as British by various authors dating back to Marshall (1885). [↑](#footnote-ref-114)
114. Removed from synonymy with *epitriptus* by Papp (2008b), who treats both species as belonging to the subgenus *Glabrobracon*, contrary to their placement in Taxapad. [↑](#footnote-ref-115)
115. Probably not a valid British or Irish species now that *B*. (*Lucobracon*) *erythrostictus* has been taken out of synonymy (Papp 1999a). [↑](#footnote-ref-116)
116. Described as a separate genus but Quicke & Sharkey (1989) suggested that *Pigeria* could be treated as a subgenus of *Bracon*, which was followed by Papp (1998). [↑](#footnote-ref-117)
117. *Bracon semiluteus* Walker, 1874 is not a synonym of *piger* (contra Morley (1913) and subsequent authors) but is a junior synonym of another species of *Bracon* (Papp, in prep.). [↑](#footnote-ref-118)
118. Marshall (1900) mentions an English specimen but this seems unlikely in view of its present southern European range. [↑](#footnote-ref-119)
119. Distribution data from Shaw (2000b). [↑](#footnote-ref-120)
120. According to Haeselbarth (in Belokobylskij et al., 2003), the name sordidator (Ratzeburg, 1844, Bracon) probably does not belong in Coeloides, so the species usually referred to as sordidator takes the name melanostigma. [↑](#footnote-ref-121)
121. The only record was due to a misidentification of Pseudovipio guttiventris (Shaw & Quicke, 1999). [↑](#footnote-ref-122)
122. Distribution data from Shaw (1999*b*). [↑](#footnote-ref-123)
123. Removed from the British list by Shaw (1999*b*) but recently found in Scotland. [↑](#footnote-ref-124)
124. Although van Achterberg (1979) treated *brevicauda* as a ‘form’ of *cruentatus*, the possibility of a separate species requires investigation. [↑](#footnote-ref-125)
125. The genus *Adelius* and related extralimital genera have usually been treated as comprising a separate subfamily, Adeliinae. Recent phylogenetic studies (Belshaw et al., 2000; Belshaw & Quicke, 2002) have placed the adeliines within the Cheloninae, as sister taxon to *Phanerotoma*. [↑](#footnote-ref-126)
126. Distribution and synonymic data taken from Huddleston (1984). [↑](#footnote-ref-127)
127. Included by Huddleston (1978) but no British specimens were seen by Huddleston (1984). [↑](#footnote-ref-128)
128. The type is lost and the species is unplaceable (Huddleston, 1984). [↑](#footnote-ref-129)
129. The name is a junior homonym of Chelonus asiaticus Fahringer, 1932. [↑](#footnote-ref-130)
130. Listed as a synonym of oculator by Huddleston (1978). [↑](#footnote-ref-131)
131. Treated as a valid genus by Papp (1996b) and Belokobylskij *et al*. (2003), sometimes treated as a straight synonym of *Chelonus* (van Achterberg & Polaszek, 1996; van Achterberg, 2004b), here treated as a subgenus of *Chelonus*, following Taxapad (Yu *et al.*, 2012). [↑](#footnote-ref-132)
132. Synonymised under *contractus* by Papp (1995a) but treated as a separate species by Tobias & Lozan (2003) and listed as such in Taxapad (Yu *et al.*, 2012). [↑](#footnote-ref-133)
133. Specimen lacking locality data, Billups coll. [↑](#footnote-ref-134)
134. Although *parcicornis* would have priority, Papp (1995a) states that the type is lost and its identity unverifiable. [↑](#footnote-ref-135)
135. Listed as a valid species by O’Connor *et al*. (1999) but as a synonym of *erythronotus* by Belokobylskij *et al*. (2003). [↑](#footnote-ref-136)
136. Huddleston (1978) listed *Sigalphus rufescens* Latreille, 1809 as a junior synonym of *dentata* but this is now considered to be a separate species, not occurring in Britain or Ireland (van Achterberg, 1990), with historical records being misidentifications. [↑](#footnote-ref-137)
137. *Phanerotoma rjabovi* Vojnovskaja-Krieger, 1929 and *media* Shestakov, 1930, synonymised under *leucobasis* by van Achterberg (1990), are now considered to be junior synonyms of *fracta* Kokujev, 1903 (Belokobylskij, 2000a). [↑](#footnote-ref-138)
138. Prior to van Achterberg’s (1990) revision, *dentata* had frequently been misidentified as *planifrons*. All of the British specimens identified as *planifrons* in the BMNH are in fact *dentata*. [↑](#footnote-ref-139)
139. Tribal classification follows Belokobylskij (1992), but note that recent work suggests that none of the major doryctine tribes are actually monophyletic (Belokobylskij et al., 2004). Generic synonyms for the most part follow Belokobylskij *et al*. (2004). [↑](#footnote-ref-140)
140. Treated as a synonym of *Dendrosoter* by Belokobylskij *et al*. (2003) but Mancini *et al*. (2003) argue that *Caenopachys* should be treated as a separate genus. [↑](#footnote-ref-141)
141. Probably occurred only in imported timber. Its British status appears to depend solely on specimens reared in July 1908 from Austrian oak imported to a timber yard near Millwall docks (Elliott & Morley, 1911). [↑](#footnote-ref-142)
142. Regarded as a synonym of *striatellus* by Belokobylskij *et al*. (2003). [↑](#footnote-ref-143)
143. Removed from synonymy with *Dendrosotinus* Telenga, 1941 by van Achterberg (2003b). [↑](#footnote-ref-144)
144. Zaldivar-Riverón *et al.* (2008*b*) did not recover *Ontsira* as monophyletic and consequently some authors (e.g. van Achterberg, 2014) prefer to treat these species as belonging to *Doryctodes*. [↑](#footnote-ref-145)
145. Belokobylskij (1992) synonymised *Wachsmannia* under *Ontsira* but van Achterberg (1995) disagreed and synonymised *Wachsmannia* under *Hypodoryctes* Kokujev, 1900. Van Achterberg (pers. comm. and in Fauna Europaea) now regards *Wachsmannia* as a separate genus again. [↑](#footnote-ref-146)
146. Other species are treated as synonyms of *silesiacus* by Belokobylskij *et al*. (2003) but van Achterberg (in Belokobylskij *et al*. 2003) states that there is biological evidence for several species. [↑](#footnote-ref-147)
147. Shaw (1998*a*) recorded *silesiacus* as British but was using the name in the broader sense (i.e. =*eccoptogastri*, =*hylesini*, =*pinicola*). Listed in Huddleston (1978) as *eccoptogastri*. [↑](#footnote-ref-148)
148. Seems to have been included on the British list only on the basis of specimens emerging from Oak (*Quercus*) timber imported from the USA (e.g. Laing, 1928). [↑](#footnote-ref-149)
149. Excluded by Shaw & Huddleston (1991), see also Shaw (1997). [↑](#footnote-ref-150)
150. Recorded as new to Britain by Perkins & Nixon (1939). Huddleston (1978) listed this as a species in need of confirmation, although, being wingless, it is straightforward to identify. There are additional British specimens in BMNH, World Museum Liverpool, Manchester Museum and Leeds City Museum. [↑](#footnote-ref-151)
151. Recorded as new to Britain by Shaw (1988*b*) but synonymised under *erythrocephalus* by Belokobylskij & Samartsev (2014) and van Achterberg (2014), who also synonymyised *erythrocephalus* under *umbratus*. [↑](#footnote-ref-152)
152. The tribal and generic classification follows Stigenberg *et al.* (2015). [↑](#footnote-ref-153)
153. Synonymised under muricatus by van Achterberg (1985), later removed from synonymy by van Achterberg (1997), with a key to the species. [↑](#footnote-ref-154)
154. Not included in Huddleston (1978) but listed as British in Taxapad (Yu *et al.*, 2012) as it was recorded by Kerrich (1932), as a tentative identification (by Roman), which has not been corroborated. [↑](#footnote-ref-155)
155. Distribution and much taxonomic data from Richards (1967), New (1970) and Loan (1974). [↑](#footnote-ref-156)
156. Treated as a subgenus of *Leiophron* by Belokobylskij (2000a). [↑](#footnote-ref-157)
157. Listed twice, under *Microctonus* and *Leiophron*, by Huddleston (1978). [↑](#footnote-ref-158)
158. The type is lost but Loan (1974) suggested, on the basis of possible type material (collected in England), that *ornata* may be a synonym of *apicalis*, which has been formally proposed by Papp (2003). [↑](#footnote-ref-159)
159. Hincks’s was a replacement name for Haliday’s use of the name picipes for a species that is distinct from *picipes* (Curtis). Preoccupied by *Leiophron antennalis* Watanabe, 1937 (now classified in *Centistes* (*Ancylocentrus*)) [↑](#footnote-ref-160)
160. Not listed by Huddleston (1978), although it was taken out of synonymy with *orchesiae* by Loan (1974). [↑](#footnote-ref-161)
161. Van Achterberg has identified a Scottish specimen as *barbiger*, currently listed as a junior synonym of *pallipes*. [↑](#footnote-ref-162)
162. Listed by Loan (1974) as *incertae sedis* within *Peristenus* or *Leiophron*, by Huddleston (1978) as a doubtfully placed species of *Leiophron* and by van Achterberg (in Belokobylskij *et al*., 2003) as a species of *Peristenus*. We cannot trace any references to this species occurring in Britain or Ireland. [↑](#footnote-ref-163)
163. According to Loan (1974), the type of *brevicornis* (Ruthe) is lost and the species cannot be identified with certainty. Recorded as British by Marshall (1872). Belokobylskij *et al*. (2003) list it as a species of *Peristenus* (=*Leiophron* according to Belokobylskij). [↑](#footnote-ref-164)
164. According to van Achterberg (in Belokobylskij *et al*., 2003) the type is lost and was probably a deformed specimen anyway. [↑](#footnote-ref-165)
165. Treated as a separate subfamily in many works (e.g. Shaw & Huddleston, 1991; van Achterberg, 1993a) but recent phylogenetic studies have shown the neoneurines to be nested within the Euphorinae (e.g. Belshaw & Quicke, 2002; Dowton et al., 2002). [↑](#footnote-ref-166)
166. Haeselbarth (2008) described many new species in *Perilitus* (*Microctonus*) but as *Microctonus* was raised to generic rank by Stigenberg *et al*. (2015), these are effectively new combinations in *Microctonus.* [↑](#footnote-ref-167)
167. Specimens tentatively associated with the type material. [↑](#footnote-ref-168)
168. Overlooked by Huddleston (1978) and O’Connor *et al*. (1999). [↑](#footnote-ref-169)
169. Richards (1960) states that it is not known with certainty from Britain. [↑](#footnote-ref-170)
170. Distribution data from van Achterberg (1992a) and BMNH. [↑](#footnote-ref-171)
171. Van Achterberg (1992a) separated *otiorhynchi* from *falcatus* but Belokobylskij *et al*. (2003), without comment, treated the two names as synonymous again. This is not followed here as *otiorhynchi* and *falcatus* seem to be distinct species. [↑](#footnote-ref-172)
172. Distribution and synonymic data from van Achterberg & Haeselbarth (2003). [↑](#footnote-ref-173)
173. The female holotype in BMNH, described in the aphidiine genus *Dyscritus* (=*Dyscritulus*, replacement name), has remained uninterpreted, although Starý (1959) recognised that it is a euphorine. It is a normal specimen of *Syntretus splendidus*. [↑](#footnote-ref-174)
174. Not listed as British or Irish by van Achterberg & Haeselbarth (2003). [↑](#footnote-ref-175)
175. *Cosmophoridia* Hedqvist, 1955 and *Eutanycerus*, usually considered synonymous with *Streblocera*, were regarded as valid subgenera by Chen & van Achterberg (1997). [↑](#footnote-ref-176)
176. Belokobylskij (1998) treats *Shawiana* and *Xenarcha* as subgenera of *Colastes*. [↑](#footnote-ref-177)
177. Treated as a species of *Colastes* (*Xenarcha*) by Belokobylskij (1998). [↑](#footnote-ref-178)
178. The Diospilini were removed to the Brachistinae by Sharanowski *et al*. (2011). [↑](#footnote-ref-179)
179. Much distribution and taxonomic data from van Achterberg (1987). [↑](#footnote-ref-180)
180. *Helconidea armator* (Marshall, 1898, *Helcon*) removed from synonymy by van Achterberg (2014). [↑](#footnote-ref-181)
181. Shaw (2010) summarises the taxonomy and biology of British and Irish species, including some distribution data. [↑](#footnote-ref-182)
182. Nixon’s description of *chlorophthalmus* validated the use of the name for this taxon; *chlorophthalmus* of authors is a misidentification (*Bracon chlorophthalmus* Spinola, 1808 is actually a species of the true *Zele*). [↑](#footnote-ref-183)
183. Considered here to be a valid species; van Achterberg (in Belokobylskij et al., 2003) considered that *piciventris* may be a distinct species but it is listed as a synonym of *moniliatus* in Taxapad. [↑](#footnote-ref-184)
184. Distribution data from Eady & Clark (1964) and van Achterberg & Haeselbarth (1983). [↑](#footnote-ref-185)
185. Van Achterberg & Haeselbarth (1983) and van Achterberg (1993b) treat *gracilipes* as a synonym of *bicolor* but Belokobylskij *et al*. (2003) list it as a synonym of *thoracicus*. [↑](#footnote-ref-186)
186. The North American *Macrocentrus aegeriae* Rohwer, 1915 was removed from synonymy by van Achterberg & Haeselbarth (1983). [↑](#footnote-ref-187)
187. Stigenberg *et al.* (2015) included the meteorines as a basal tribe of Euphorinae, rather than as a separate subfamily, as was the preference of many authors, for example, Shaw & Huddleston (1991). In light of their very different developmental biology (parasitoids of larval Lepidoptera and Coleoptera as opposed to parasitising adult insects) and sister-group position to the euphorines, we retain the subfamily rank for Meteorinae. [↑](#footnote-ref-188)
188. Distribution data from Huddleston (1980), Stigenberg & Ronquist (2011) and Stigenberg & Shaw (2013), except where noted. Taxonomy follows Stigenberg & Ronquist (2011). [↑](#footnote-ref-189)
189. Listed by Huddleston (1978) but Huddleston (1980) could not find any British or Irish material. Shaw (1988a) subsequently recorded it as a British species. [↑](#footnote-ref-190)
190. This has recently been identified as *Meteorus necator* (e.g. Belokobylskij *et al.*, 2003) but, according to Stigenberg & Ronquist (2011), *Ichneumon necator* Fabricius, 1777, is actually a species of Microgastrinae. [↑](#footnote-ref-191)
191. Separated from *cinctellus* by Stigenberg & Ronquist (2011); Marshall (1887) gave no locality data so the first documented British occurrence was published by Stigenberg & Shaw (2013). [↑](#footnote-ref-192)
192. Synonymic and some distribution data from van Achterberg (1979, 1984a) and Stigenberg & Shaw (2013). *Zele longicauda* Curtis, 1832 is apparently a synonym of a species of *Eubazus* (van Achterberg, pers. comm.). [↑](#footnote-ref-193)
193. Included in Huddleston’s (1978) checklist but we can trace no specimens or literature citations. [↑](#footnote-ref-194)
194. The generic classification of Microgastrinae broadly follows Papp (1988), based on Mason’s (1981*a*) phylogenetic treatment of microgastrines that split up the large genus ‘*Apanteles’*, which in turn built upon Nixon’s (1965) assignment of ‘*Apanteles*’ into species groups. We do not follow van Achterberg’s (2003a) recent generic revision (reflected in Fauna Europaea), which reassigned many genera to *Apanteles* and *Protapanteles*. We consider this reclassification to be premature, being based on very little explicit character evidence and, with its concentration on western Palaearctic species, not really addressing the affinities of most of the world’s microgastrine species. However, there are undoubted merits to van Achterberg’s treatment as not all of the genera currently employed are well-defined. The following genera are recognised as valid by van Achterberg (2003a): *Apanteles* (=*Choeras*, *Dolichogenidea*, *Iconella*, *Illidops*, *Pholetesor*), *Cotesia*, *Deuterixys*, *Diolcogaster*, *Hygroplitis*, *Microgaster*, *Microplitis*, *Paroplitis* and *Protapanteles* (=*Distatrix*, *Glyptapanteles*, *Rasivalva*, *Sathon*). Some distribution data taken from Nixon (1965, 1968, 1970, 1972, 1973, 1974, 1976), remainder from Shaw (2012) and NMS. [↑](#footnote-ref-195)
195. The generic placement of several species treated by Nixon (1973) in his *metacarpalis* group, here largely apportioned between *Apanteles* and *Dolichogenidea* following Papp (1988), is questionable (cf. van Achterberg, 2003a). [↑](#footnote-ref-196)
196. We follow Mason’s (1981*a*) placement of lacteus in *Apanteles* rather than Papp’s (1988) placement in *Dolichogenidea*. [↑](#footnote-ref-197)
197. These names had been included in *Apanteles* in the pre-Mason (1981*a*) sense; their current generic placement is unclear. [↑](#footnote-ref-198)
198. This name appeared in Huddleston (1978) but is not listed by Papp (1988) or van Achterberg (2003a) and remains uninterpreted. [↑](#footnote-ref-199)
199. This name appeared in Huddleston (1978) but is not listed by Papp (1988) or van Achterberg (2003a). [↑](#footnote-ref-200)
200. Papp (1987) intended to deal with this name, with a footnote in that paper saying that it would be dealt with under *A. xanthostigma*, but there is no mention there of *picipes*. It is presumed that the name *picipes* is a synonym or a *nomen dubium*; it is not listed as a valid species by van Achterberg (2003a). [↑](#footnote-ref-201)
201. Listed under *Apanteles* and *Microgaster* in Huddleston (1978). [↑](#footnote-ref-202)
202. Papp (1988) notes, but does not follow, the view that *appellator* may be the valid name (cf. Kotenko and Tobias, 1986). Shaw (2012) has found that reared ‘*appellator*’ and ‘*litae*’ appear to be conspecific. The situation is complicated by Nixon’s (1972) treatment of some series (from a different host in Cyprus, and from Egypt) as ‘*litae* var *operculellae*’, and it is this that Papp (1988) lists as a junior synonym of *appellator*. [↑](#footnote-ref-203)
203. Revised status (Shaw, 2012): inadvertently listed as a synonym of candidata by van Achterberg (1997) due to a drafting error (van Achterberg, pers. comm.). [↑](#footnote-ref-204)
204. Shaw (2012) gives a diagnosis; not treated by Nixon or Papp. [↑](#footnote-ref-205)
205. Papp (1981) reports that the type series of *tadzhica* belongs to two species (*lacteus* and *longipalpis*) but did not select a lectotype; Belokobylskij *et al*. (2003) treated the name as a synonym of *longipalpis*. [↑](#footnote-ref-206)
206. Transferred from *Apanteles* in anticipation of publication by Jose Fernandez-Triana. [↑](#footnote-ref-207)
207. Shaw (2012) gives a diagnosis; not treated by Nixon or Papp. [↑](#footnote-ref-208)
208. Recorded as British by Nixon (1972) but omitted by Huddleston (1978). [↑](#footnote-ref-209)
209. A parasitoid of synanthropic Lepidoptera species and probably introduced to Britain and Ireland (Nixon, 1976). [↑](#footnote-ref-210)
210. Listed as a British species by Huddleston (1978) in error, there are no British records. [↑](#footnote-ref-211)
211. Listed as a British species by Huddleston (1978) in error, only known from southern and Eastern Europe and Israel. [↑](#footnote-ref-212)
212. Listed as a British species by Huddleston (1978) in error (see note under *Napamus vipio*). [↑](#footnote-ref-213)
213. Listed as a British species by Huddleston (1978) in error, there are no British records. [↑](#footnote-ref-214)
214. Notwithstanding Papp (1978), we regard this name as uncertainly interpreted, but in any case we have not seen British material that conforms to Papp's (1978) interpretation. [↑](#footnote-ref-215)
215. Mistakenly listed as a British species by Kloet & Hincks (1945) and Huddleston (1978). Shenefelt (1973) listed it as British on the misunderstanding that Morley & Rait-Smith (1933) had produced a catalogue of British Lepidoptera-parasitoid asscociations, whereas their listings also included non-British rearings from hosts that occur in Britain. Morley & Rait-Smith (1933) cite Marshall (1896) as the source of host records for *vipio*, who does not mention Britain. *Apanteles vipio* was transferred from *Illidops* by Papp (1993). [↑](#footnote-ref-216)
216. Van Achterberg’s (1997) synonymy of *bicolor* under *circumscriptus* is not followed here (Shaw, 2012); it is probable that his reared series represents two species, *bicolor* and *circumscriptus*. [↑](#footnote-ref-217)
217. Shaw (2012) tentatively identified *exiguus sensu* Nixon (1973) as an extreme of morphological variation within *laetus*. [↑](#footnote-ref-218)
218. Although this generic placement (Papp, 1988) was followed by Shaw (2012) is seems very likely to be inappropriate. [↑](#footnote-ref-219)
219. Some taxonomic and distribution data for species parasitizing Lycaenidae taken from Shaw (2007). [↑](#footnote-ref-220)
220. Nixon’s (1974) *arctica* encompassed two species that occur in Britain, *astrarches* and *tenebrosa*. It is not clear which, if either, the name *arctica* is associated with. The name *astrarches* was placed, erroneosly, in synonymy with *arctica* by Nixon (1974), which was followed by Huddleston (1978). Shaw (2007) clarified the status of *astrarches*. [↑](#footnote-ref-221)
221. Papp (1987) synonymised *coryphe* under *rubripes* but this seems unwarranted, given the distinctly different hosts and other aspects of biology. [↑](#footnote-ref-222)
222. It is not clear from Nixon (1974) that this species occurs in Britain, despite its being ‘bred in captivity at Slough’. [↑](#footnote-ref-223)
223. Listed as a senior synonym of *cajae* in Taxapad (Yu *et al.*, 2012), following Marshall (1885), who listed *cajae* as the senior synonym. Papp (2005b) lists *perspicua* as a tentative synonym (which would have priority) of *ofella*. [↑](#footnote-ref-224)
224. In NMS numerous series reared solitarily from various species of Pterophoridae are probably conspecific but show a great variation in extent of pilosity of the antenna. [↑](#footnote-ref-225)
225. Nixon’s (1974) *praepotens* is apparently correctly called *sericea* (Belokobylskij et al., 2003). [↑](#footnote-ref-226)
226. Nixon’s (1974) *praepotens* is apparently correctly called *sericea* (Belokobylskij et al., 2003). [↑](#footnote-ref-227)
227. Nixon’s (1974) *arctica* encompassed two species that occur in Britain, *astrarches* and *tenebrosa*. It is not clear which, if either, the name *arctica* is associated with. The name *astrarches* was placed, erroneosly, in synonymy with *arctica* by Nixon (1974), which was followed by Huddleston (1978). Shaw (2007) clarified the status of *tenebrosa*. [↑](#footnote-ref-228)
228. Not a British or Irish species. Marshall’s description was based on unprovenanced material reared from a host that, on inspection by MRS, is almost certainly not British. The current synonymy is also in doubt. [↑](#footnote-ref-229)
229. Although listed as a British species by various authors we can find no evidence that it has occurred here. [↑](#footnote-ref-230)
230. Appeared in Huddleston (1978) as Roman (1912) incorrectly synonymised *tenebrosa* under *saltator*. [↑](#footnote-ref-231)
231. No evidence that this is a British or Irish species. [↑](#footnote-ref-232)
232. See Mason (1981) and Fernandez-Triana (2015), who restricted *Protomicroplitis* to a few Nearctic and Neotropical species. [↑](#footnote-ref-233)
233. Van Achterberg (1997) reinterpreted this name and treated *majalis* as the valid name for the species called *callidus* by Nixon (1973) and Papp (1983). [↑](#footnote-ref-234)
234. Wilkinson (1945) dealt with the identity of the real *liparidis*, which does not occur in Britain or Ireland. [↑](#footnote-ref-235)
235. Generic placement in doubt; some authors place both *fausta* and *lateralis* in *Sathon*. Papp (1983) synonymised *fausta* under *Apanteles eugeni* Papp, 1972 but later (Papp, 1988) rescinded his action. Unfortunately Papp (1972) had chosen a non-reared specimen as holotype but a paratype of *eugeni* reared from *Anthophila fabriciana* (Linnaeus) is in BMNH and appears to be conspecific with *fausta* (described from this host). It seems probable that Papp's (1983) synonymy was justified but, as this requires further investigation, the name *fausta* is retained for now. [↑](#footnote-ref-236)
236. Generic placement in doubt; some authors place both *fausta* and *lateralis* in *Sathon*. [↑](#footnote-ref-237)
237. Described by Nixon (1973) partly from Scottish material but omitted by Huddleston (1978). [↑](#footnote-ref-238)
238. Van Achterberg (1997) treated *majalis* as the valid name for the species called *callidus* by Nixon (1973) and Papp (1983). [↑](#footnote-ref-239)
239. Listed as a British species by Huddleston (1978) in error; there is no evidence that it has occurred here. [↑](#footnote-ref-240)
240. Included in *Protapanteles* by Papp (1988) but Mason (1981) treated it as a species of *Glyptapanteles*. Nixon (1973) was unable to suggest close relatives. [↑](#footnote-ref-241)
241. Specimen from Islay in Hunterian Museum, Glasgow, det. MRS. [↑](#footnote-ref-242)
242. Both of these species were mistakenly listed by Huddleston (1978); there is no evidence that they are British or Irish. [↑](#footnote-ref-243)
243. The current usage of the name *Microgaster* was restored by Opinion 1510 (ICZN, 1988), after temporarily being applied to the genus here called *Microplitis* (with *Microgaster* as currently understood being referred to *Lissogaster*). This was an unfortunate name change as it coincided with Papp’s (1976, 1984) revisions of the genera, as well as several other important papers. Unless noted otherwise, distribution data taken from NMS, Nixon (1968) and Shaw (2012). Papp’s (1976) subsequent revision of the genus added many species to Nixon’s (1968) revision, and van Achterberg (1997) established the precedence of several Haliday names. [↑](#footnote-ref-244)
244. Raised from synonymy under *meridiana* (Papp, 1999c) by Shaw (2012). [↑](#footnote-ref-245)
245. It seems that *laeviscuta* has been included as a junior synonym of *hospes* in Fauna Europaea, with *globata* not referred to. Shaw (2012) notes that *globata* as perceived by Papp (1976) is almost certainly an aggregate. [↑](#footnote-ref-246)
246. Some Ruthe names traditionally regarded as synonyms of *tibialis* probably do not belong here (Shaw, 2012). [↑](#footnote-ref-247)
247. A species of doubtful status. Curtis (1837) listed *nigricans* as a British species and Papp (1976), who stated that it was known only from the male, therefore listed it as English. Nixon (1968), however, did not deal with the name. [↑](#footnote-ref-248)
248. Listed as doubtfully British by Huddleston (1978) on the basis of Papp’s (1976) listing of ‘?England’. No evidence that this is really a British or Irish species. [↑](#footnote-ref-249)
249. Listed as a British species by Huddleston (1978) in error; no evidence that this is a British or Irish species. [↑](#footnote-ref-250)
250. British specimens, misidentified as *fischeri* by Nixon (1968), represented an undescribed species which was described by Shaw (2012) as *raschkiellae*. [↑](#footnote-ref-251)
251. Listed as doubtfully British by Huddleston (1978) on the basis of Papp’s (1976) listing of ‘?England’. No evidence that this is really a British or Irish species. [↑](#footnote-ref-252)
252. Recorded as British by Marshall (1885), but probably in error as his diagnosis appears not to have been a *Microgaster* species in the modern sense. Papp’s (1976) listing of England probably simply reflects Marshall’s record, as does the listing in Huddleston (1978), and there is no evidence that this is a British or Irish species. [↑](#footnote-ref-253)
253. Unless noted otherwise, distribution data taken from Nixon (1970). Papp (1984) extensively revised the synonymy (as *Microgaster* species: unfortunately at that time *Microplitis* was briefly being regarded as a synonym of *Microgaster*, but this was subsequently rescinded by Opinion 1510 (ICZN, 1988), whose conclusions are largely followed here. [↑](#footnote-ref-254)
254. It is unclear on what basis this species was listed as British by Papp (1984) (Shaw, 2012). [↑](#footnote-ref-255)
255. Listed as a separate species in Taxapad (Yu *et al.*, 2012) but we follow van Achterberg, in Fauna Europaea. However, the status of *sordipes* remains uncertain. [↑](#footnote-ref-256)
256. Papp (1984) suggests that *semicircularis* (Ratzeburg, 1844, *Microgaster*) (type destroyed) may be a senior synonym. [↑](#footnote-ref-257)
257. Nixon’s (1970) interpretation of *trochanterata* (not *tuberculifer*, of which *trochanterata* is a junior synonym) is actually referable to *malimbus* (Shaw, 2012). [↑](#footnote-ref-258)
258. Papp (1984) suggests that *stigmaticus* may be a senior synonym. [↑](#footnote-ref-259)
259. No evidence that this species has occurred in Britain or Ireland; probably listed in Shenefelt (1973) and Huddleston (1978) because Nixon (1970) reported its distribution as north-west European. [↑](#footnote-ref-260)
260. Belokobylskij *et al*. (2003) include *Microtypus*, without comment, in the Orgilinae, where it had traditionally been placed prior to recent phylogenetic work. In the molecular phylogeny of Belshaw & Quicke (2002) the genus *Microtypus* is the sister group to Homolobinae, as also found by Sharanowski *et al*. (2011) (albeit with this clade the sister group to Orgilinae). This relationship was originally suggested by van Achterberg (1984b). [↑](#footnote-ref-261)
261. Nomenclature follows Čapek & van Achterberg (1992). [↑](#footnote-ref-262)
262. There are also several unrecognised species in Britain. [↑](#footnote-ref-263)
263. The generic and tribal classification of opiines has been largely chaotic (Wharton, 1988a). In Fauna Europaea, van Achterberg has enacted many taxonomic changes resulting from his work on Western Palaearctic Opiinae (in prep.), which are followed here. Some changes to the generic classification have been published by Wharton (1988a), van Achterberg (2004a,b, and in Belokobylskij *et al*., 2003) and by Li *et al*. (2013). Wharton (1988a) argued against recognition of tribes within Opiinae, except perhaps Ademonini (for the genus *Ademon*), with all the other genera in Opiini; van Achterberg (in prep.) does not recognise Ademonini. Some distribution data from Fischer (1958, 1967, 1997). [↑](#footnote-ref-264)
264. Listed as a synonym of *Opius singularis* in Huddleston (1978). [↑](#footnote-ref-265)
265. Although Wharton (1993) noted that the type (and only included) species of *Compressaria* is the same species as the type of *Bathystomus*, he did not formally synonymise these names and the synonymy has not been picked up on by, e.g. Belokobylskij *et al*. (2003) and Taxapad (Yu *et al.*, 2012). Wharton (1988a) demonstrated that *Bathystomus* is a valid genus but Belokobylskij *et al*. (2003) treated it as a synonym of *Diachasma*. Van Achterberg (2014) formally synonymised *Compressaria* and *pugnatrix*. Huddleston (1978) included *Compressaria pugnatrix* as a species of Rogadinae. [↑](#footnote-ref-266)
266. Treated as a valid genus following Wharton (2006). [↑](#footnote-ref-267)
267. Synonymised under *carbonarius* by van Achterberg (1975), *impressus* was removed from synonymy by Fischer (1997), a move which is not accepted by van Achterberg (in prep.). Supporting van Achterberg’s synonymy, Godfray (unpublished) has repeatedly reared specimens resembling both ‘species’ from collections of hosts made at the same place and time. [↑](#footnote-ref-268)
268. Listed as British by Kloet & Hincks (1945) but omitted by Huddleston (1978). Material in NMS has been identified by van Achterberg. [↑](#footnote-ref-269)
269. Regarded by Wharton (1983, 1988a) as a junior synonym of *Opius*. [↑](#footnote-ref-270)
270. According to Walker & Wharton (2011), *Opius paradoxus* Ratzeburg, 1848, sometimes treated as a valid name (synonymous with *abnormis*) should be regarded as invalid as it was first proposed as a synonym of *abnormis*; Walker & Wharton (2011) also provide some locality data. [↑](#footnote-ref-271)
271. Mis-placed in *Eurytenes* (van Achterberg, pers. comm.). [↑](#footnote-ref-272)
272. The generic name *Stigmatopoea* has been treated as a synonym of *Xynobius* by van Achterberg (2004a) but the type species, *macrocerus*, has been regarded as a species of *Eurytenes*, a genus which was ignored by van Achterberg (2004a). Both *Xynobius* and *Stigmatopoea* are treated as subgenera of *Eurytenes*, following Wharton (2006), although Li *et al*. (2013) again synonymised *Stigmatopoea* with *Xynobius*. [↑](#footnote-ref-273)
273. Generic synonymy from van Achterberg (2004a) and Li *et al*. (2013) but treated as a subgenus of *Eurytenes* by Wharton (2006). [↑](#footnote-ref-274)
274. Described from Welsh material (Fischer, 1967) but omitted by Huddleston (1978). [↑](#footnote-ref-275)
275. Raised from synonymy with *Phaedrotoma* by Li *et al*. (2013). [↑](#footnote-ref-276)
276. Raised from synonymy with *Phaedrotoma* by Li *et al*. (2013). [↑](#footnote-ref-277)
277. Raised from synonymy with *Opius* by Li *et al*. (2013). [↑](#footnote-ref-278)
278. Recorded as a British species by various authors, latterly by Fischer (1967), and reared recently by Godfray (unpublished), but omitted by Huddleston (1978). [↑](#footnote-ref-279)
279. Restricted by van Achterberg & Salvo (1997) to a group of species with distinctive mandibles. [↑](#footnote-ref-280)
280. Listed as a synonym of *longicornis* Thomson, 1895 in Taxapad (Yu *et al.*, 2012), regarded as a separate, valid species by van Achterberg (in prep.). Recorded as British by Fischer (1967) as *longicornis* but omitted by Huddleston (1978). [↑](#footnote-ref-281)
281. Listed as a synonym of *pygmaeator* in Taxapad, regarded as a separate, valid species (=*latipes* *sensu* Fischer) by van Achterberg (2014). [↑](#footnote-ref-282)
282. Fischer (1960) mistakenly listed this species as *fuscipennis* (Szépligeti, 1914, *Rhinoplus*) (van Achterberg, pers. comm.), a separate species now placed in the genus *Pseudorhinoplus* Fischer, 1972. [↑](#footnote-ref-283)
283. Recorded as new to Britain by Godfray (1986) but synonymised with *levis* by van Achterberg (in Belokobylskij *et al.*, 2003). [↑](#footnote-ref-284)
284. Both *clarus* and *spretus* were also synonymised by van Achterberg (1997) but Fischer’s publication pre-dated this. [↑](#footnote-ref-285)
285. *Phaedrotoma* was raised from synonymy with *Opius* by van Achterberg & Salvo (1997) to accommodate many species previously placed in *Opius*. Generic synonymy follows Li *et al*. (2013). [↑](#footnote-ref-286)
286. Recorded as British by Fischer (1960) but omitted by Huddleston (1978). [↑](#footnote-ref-287)
287. Listed as a synonym of *Opius instabilis* in Taxapad (Yu *et al.*, 2012) but regarded as a valid species, in *Phaedrotoma*, by van Achterberg (in prep.). [↑](#footnote-ref-288)
288. Described by van Achterberg because the material named *Opius* *reconditor* (now classified in *Rhogadopsis*) by authors (e.g. Fischer, 1972; Fischer & Koponen, 1999) is not conspecific with the type. Both species apparently occur in Britain. [↑](#footnote-ref-289)
289. Listed as a synonym of *Opius ambiguus* in Taxapad (Yu *et al.*, 2012) but regarded as a valid species, in *Phaedrotoma*, by van Achterberg (in prep.). [↑](#footnote-ref-290)
290. Omitted by Huddleston (1978). [↑](#footnote-ref-291)
291. Listed by Huddleston (1978) but we cannot trace any published records or specimens. [↑](#footnote-ref-292)
292. Raised from synonymy with *Phaedrotoma* by Li *et al*. (2013). [↑](#footnote-ref-293)
293. Synonymised with *reconditor* by van Achterberg (1997) but listed as a separate species in Taxapad (Yu *et al.*, 2012); re-synonymised by van Achterberg (2014). [↑](#footnote-ref-294)
294. Listed as English by Fischer & Koponen (1999), presumably following Fischer (1958), but not listed in Huddleston (1978). [↑](#footnote-ref-295)
295. There may be a literature citation for its occurrence in Britain (van Achterberg, pers. comm.) but we have been unable to trace it. [↑](#footnote-ref-296)
296. Listed in Fauna Europaea as occurring in Britain, but presumably mistakenly as no literature or specimen records can be located. Known only from Georgia, Greece and Ukraine (data from Taxapad). [↑](#footnote-ref-297)
297. Taxonomic and much distribution data from Taeger (1989). [↑](#footnote-ref-298)
298. Although Taeger (1989) clarified the identity of *obscurator auctt*. as *leptocephalus*, this species was recorded as *obscurator* by O’Connor *et al*. (1999). [↑](#footnote-ref-299)
299. Apparently recorded as Irish under the name *Microdus laevigator* Nees, 1812 (O’Connor *et al*., 1999), which is listed as a species inquirendae by Taeger (1989). [↑](#footnote-ref-300)
300. Included as British in Fauna Europaea and Taxapad (Yu *et al.*, 2012) (but not by Huddleston, 1978); this seems to be on the basis of Fitch’s descriptions of the synonymous names *dubius* and *imminens* in Marshall’s (1885) monograph of British Braconidae. However, the types of Fitch’s species are from the Ruthe collection of German material and we have seen no British or Irish specimens of *biglumis*. [↑](#footnote-ref-301)
301. Most synonymy has been omitted; published taxonomy has been confused and there are several more species present than have been recorded in the literature, with the application of names not yet settled. [↑](#footnote-ref-302)
302. Treated as a synonym of *meditator* by Belokobylskij & Tobias (1986) and Belokobylskij *et al*. (2003) but here treated as a valid species. [↑](#footnote-ref-303)
303. Treated as a synonym of *meditator* by Belokobylskij & Tobias (1986) and Belokobylskij *et al*. (2003) but here treated as a valid species. [↑](#footnote-ref-304)
304. Treated as a synonym of *Acrisis* in Fauna Europaea, as a separate genus by Belokobylskij *et al*. (2003). [↑](#footnote-ref-305)
305. Zaldivar-Riverón *et al*. (2006) and Sharanowski *et al*. (2011) both found, on the basis of molecular phylogenetic results, that the morphologically and biologically aberrant genus *Histeromerus* belongs in Rhyssalinae; however, there is as yet no indication as to how *Histeromerus* can be accommodated within the existing tribal classification of Rhyssalinae, so we simply use Histeromerini for now. [↑](#footnote-ref-306)
306. Traditionally treated as a species separate from *minutus* (e.g. Huddleston, 1978; Fauna Europaea) but synonymised by Belokobylskij (1998); we follow this, although it seems likely that there are two species involved. [↑](#footnote-ref-307)
307. Listed as a species of *Rhysipolis* in Huddleston (1978). [↑](#footnote-ref-308)
308. Distribution data mostly from NMS and BMNH. [↑](#footnote-ref-309)
309. Resurrected by Zaldívar-Riverón *et al*. (2008). [↑](#footnote-ref-310)
310. *Aleiodes* species are further subdivided in Taxapad (Yu *et al.*, 2012) into the subgenera *Aleiodes*, *Chelonorhogas*, *Neorhogas* and *Heterogamus* (for those occurring in Britain); the latter is considered here to be a distinct genus (see note under *Heterogamus*). [↑](#footnote-ref-311)
311. Just one British specimen of this Mediterranean species, considered to be an erratic. [↑](#footnote-ref-312)
312. Anticipates synonymy to be published by van Achterberg & Shaw (in prep.). [↑](#footnote-ref-313)
313. This species, whose name is not yet published (van Achterberg & Shaw, in prep.), is very common in the British Isles and M.R. Shaw det. labels (as *borealis*) have been left in several British collections (up to 2007). The true *A. borealis* (Thomson, 1892, *Rogas*) has not been found in the British Isles. [↑](#footnote-ref-314)
314. *Sensu* neotype (to be published by van Achterberg & Shaw, in prep.). [↑](#footnote-ref-315)
315. Anticipates synonymy to be published by van Achterberg & Shaw (in prep.). [↑](#footnote-ref-316)
316. Distribution data are not given as this name covers an aggregate of at least four species in Britain (van Achterberg & Shaw, in prep.). Further, it is not clear to which the name *gastritor* should be applied. [↑](#footnote-ref-317)
317. *Bracon testaceus* Spinola, 1808 is actually *Rogas luteus*, and ‘*Aleiodes testaceus* (Spinola)’ is not a valid taxon, although it has been applied to several small, orangeish *Aleiodes*, epecially in the *gastritor* aggregate. [↑](#footnote-ref-318)
318. Anticipates synonymy to be published by van Achterberg & Shaw (in prep.). [↑](#footnote-ref-319)
319. Treated as a synonym of *pallidicornis* by Belokobylskij *et al*. (2003), following Papp (1985). [↑](#footnote-ref-320)
320. Listed as a subspecies of *circumscriptus* in Taxapad (Yu *et al.*, 2012), following Papp (1999c) and other authors. [↑](#footnote-ref-321)
321. Brought out of synonymy with *ductor* by van Achterberg (1997). [↑](#footnote-ref-322)
322. Specimen in BMNH (seen by MRS). [↑](#footnote-ref-323)
323. The name *armatus* has been misapplied to a range of undescribed, medium-sized, orange species, at least three of which occur in Britain and Ireland and will be described by van Achterberg & Shaw (in prep.). [↑](#footnote-ref-324)
324. English and Welsh records from specimens seen by MRS in, respectively, the Hope Department, Oxford, and Doncaster Museum. [↑](#footnote-ref-325)
325. Anticipates synonymy to be published by van Achterberg & Shaw (in prep.). [↑](#footnote-ref-326)
326. MRS has seen the specimens that this record is based upon (in Ipswich Museum) and they are not *arcticus*. [↑](#footnote-ref-327)
327. Usually treated as a subgenus of *Aleiodes* (as in Taxapad (Yu *et al.*, 2012)) but Zaldivar-Riverón *et al*. (2004, 2008*a*) have shown that, on the basis of venom apparatus characters and molecular sequence data, respectively, *Heterogamus* species form a clade distinct from *Aleiodes* species. Murray (1939) and Shaw (2000) published some distribution records. [↑](#footnote-ref-328)
328. Irish records (O’Connor *et al*., 1999) are erroneous (Shaw, 2000). [↑](#footnote-ref-329)
329. Nomenclature and distribution data from Belokobylskij (1995) and NMS. [↑](#footnote-ref-330)
330. A species of uncertain status (Belokobylskij, pers. comm. to MRS), listed as a synonym of *excubitor* in Taxapad (Yu *et al.*, 2012), following Szépligeti (1906). [↑](#footnote-ref-331)
331. Listed by Huddleston (1978), probably on the basis of Morley & Rait-Smith’s (1933) host-parasitoid catalogue, but this publication included non-British rearings from Lepidoptera that occur in Britain and there is no evidence that *tricolor*, a parasitoid of *Apoda limacodes* (Hufnagel) (Lepidoptera: Limacodidae), has ever been found in Britain or Ireland. [↑](#footnote-ref-332)
332. Shaw & Huddleston (1991) were unable to trace any British specimens. [↑](#footnote-ref-333)
